# Mutant p53-dependent mitochondrial metabolic alterations in a mesenchymal stem cell-based model of progressive malignancy

**DOI:** 10.1038/s41418-018-0227-z

**Published:** 2018-11-09

**Authors:** Giuseppe Lonetto, Gabriela Koifman, Alon Silberman, Ayush Attery, Hilla Solomon, Smadar Levin-Zaidman, Naomi Goldfinger, Ziv Porat, Ayelet Erez, Varda Rotter

**Affiliations:** 1 Department of Molecular Cell Biology, Weizmann Institute of Science, Rehovot, 76100 Israel; 2 Department of Biological Regulation, Weizmann Institute of Science, Rehovot, 76100 Israel; 3 Department of Chemical Research Support, Weizmann Institute of Science, Rehovot, 76100 Israel; 4 Department of Life Sciences Core Facilities, Weizmann Institute of Science, Rehovot, 76100 Israel

**Keywords:** Stem-cell research, Cancer metabolism

## Abstract

It is well accepted that malignant transformation is associated with unique metabolism. Malignant transformation involves a variety of cellular pathways that are associated with initiation and progression of the malignant process that remain to be deciphered still. Here we used a mouse model of mutant p53 that presents a stepwise progressive transformation of adult Mesenchymal Stem Cells (MSCs). While the established parental p53Mut-MSCs induce tumors, the parental p53WT-MSCs that were established in parallel, did not. Furthermore, tumor lines derived from the parental p53Mut-MSCs (p53Mut-MSC-TLs), exhibited yet a more aggressive transformed phenotype, suggesting exacerbation in tumorigenesis. Metabolic tracing of these various cell types, indicated that while malignant transformation is echoed by a direct augmentation in glycolysis, the more aggressive p53Mut-MSC-TLs demonstrate increased mitochondrial oxidation that correlates with morphological changes in mitochondria mass and function. Finally, we show that these changes are p53Mut-dependent. Computational transcriptional analysis identified a mitochondrial gene signature specifically downregulated upon knock/out of p53Mut in MSC-TLs. Our results suggest that stem cells exhibiting different state of malignancy are also associated with a different quantitative and qualitative metabolic profile in a p53Mut-dependent manner. This may provide important insights for cancer prognosis and the use of specific metabolic inhibitors in a personalized designed cancer therapy.

## Introduction

A correlation between alterations in metabolism and carcinogenesis is well accepted [1, 2]. The Warburg effect, already reported last century, showed that cancer cells exhibit enhanced energy-dependent glycolysis resulting in increased glucose metabolism and enhanced production of lactate [3, 4, 5]. Metabolism is a multi-pathway cellular program that guaranties energy supply for the cells. The transition of a normal differentiated cell into highly aggressive tumor cells involves defined cellular steps that are associated with the deregulation of important cell cycle control genes. Given this, here we examined whether tumor cells with a defined oncogenic status altered their metabolic pathways. Mutant p53 play an important role in both the initiation and progression of cancer development. Depending on the specific cancer type [6, 7], expression of mutant p53 (p53Mut) manifest Oncogenic-Gain-of-Function (GOF) in cancer cells that is associated with changes in metabolism [2, 8]. To further assess a correlation between cancer development and Mutp53 expression in relation to changes in metabolism, we adapted a p53-dependent murine experimental model where we can follow the transition of early non-transformed stem cells into highly aggressive cancer stem cells-like (CSCs-like) cells. We have previously developed an adult Mesenchymal Stem Cells (MSCs) model derived from either bone marrow of parental wild type p53 (p53WT) or parental Mutp53 (p53Mut) expressing mice carrying the R172H hotspot mutation. Only the latter, developed tumors in vivo [9]. The murine R172H mutation corresponds to the R175H hotspot mutation in human and it is a well-known mutant protein with GOF activity [9, 10].

Furthermore, tumor cell lines derived from the parental p53Mut-MSCs (p53Mut-MSC-TLs) induced highly aggressive tumors [10]. This model is presenting an in vitro step-wise system, permitting the follow-up of the increased capacity of tumorigenicity [10] and thus enabling deciphering metabolic changes that accompany each step of p53Mut-dependent malignancy.

Comparison of various metabolic profiles indicated that while parental p53Mut-MSCs relied mainly on glucose uptake and lactate secretion, the more aggressive-derived tumor cell lines p53Mut-MSC-TLs increased their mitochondrial mass and oxidative metabolism. These augmented changes in metabolism were Mutp53-dependent. These data suggest that cancer cells are characterized by metabolic pathways which are unique for each different malignant status.

## Materials and methods

### Mice strains

The following mice strains were used in this study: C57BL/6 containing p53WT or p53Mut alleles (kindly provided by Professor G. Lozano) and NOD.CB17-prkdc-SCID/NCrHsd (Harlan, Rehovot, Israel). Animal protocols were approved by the Institutional Animal Care and Use Committee of the Weizmann Institute of Science.

### Cell cultures

“Cancer stem cells-enriched cultures”. p53Mut-MSC-TLs were established as previously reported [10]. Parental MSCs and p53Mut-MSC-TLs were grown in MSCs medium, containing murine MesenCult Basal Media (StemCell Technologies, Vancouver, BC, Canada) supplemented with 20% murine mesenchymal supplement (StemCell Technologies), 60 mg/ml penicillin, 100 mg/ml streptomycin and 50 mg/ml kanamycin. MEFs were prepared as previously described [11] and were maintained in DMEM (Biological Industries, Bet-Haemek, Israel) supplemented with 10% FCS and antibiotics. H1299 cells were obtained from the ATCC and maintained in RPMI supplemented with FCS 10% and antibiotics. Cells were incubated at 37 °C in a humidified atmosphere of 5% O_2_, 7.5% CO_2_.

### Glucose uptake assay

Cells were first nutrients starved for 4 h, then incubated with 2-NBDG fluorescent glucose analogue (50 µM for 30 minutes; Molecular Probes, USA. Cat. Number: N13195). Stained cells were subsequently detected by Imaging Flow Cytometry (IFC).

### Glut1 membrane localization assessment

Following fixation and permeabilization (FixPerm kit, BD), cells were incubated with primary antibodies directed against Glut1 (Abcam, [EPR3915], Alexa Fluor 647, ab915020). Cells were then gated for Glut1-positive cells (at least 10,000 cells in each population. Stained cells were subsequently detected by Imaging Flow Cytometry (IFC).

### MitoTrackerGreen (MTG), TMRE, ROS assessment

Cells were incubated with dihydroethidium (DHE) 5 µM, MitoSOX 5 µM, TMRE 1 µM or MTG 100 nm for 20 minutes. DHE (Molecular Probes; Cat. number: D1168), MitoSOX (Molecular Probes; Cat. number: M36008), TMRE (Sigma; Cat. Number 87917), MTG (Invitrogen; Cat. number: M7514). Stained cells were subsequently detected by Imaging Flow Cytometry (IFC).

#### Apoptosis

Nuclear fragmentation index was quantified according to nuclear morphology, using the area of the 50% top intensity pixels (Area_Threshold 50) vs. the contrast of the brightfield. Apoptotic cells display a lower area and a higher contrast. For Annexin^+^ cells, the ratio between the area of Annexin staining and the bright field area was calculated, and plotted against the Max Pixel value of Annexin, to distinguish between single bright spots and the typical Annexin V membrane staining.

### Imaging flow cytometry (IFC)

Stained cells were subsequently analyzed by Imaging Flow Cytometry analysis (ImageStreamX mark II - Amnis Corp., part of EMD Millipore, Seattle, WA, USA). Cells were gated for single cells using the area and aspect ratio features, and for focused cells using the Gradient RMS feature, as previously described [12]. Data were analyzed using the IDEAS 6.2 software. (Amnis Corp.). Debris and aggregates were eliminated using the area vs. intensity of the DNA staining. Cropped cells were further eliminated using the centroid X feature (the distance of the cell from the left corner of the view field).

#### Glucose uptake and intracellular ROS

Mean Pixel value (the average of the background-subtracted pixels contained in cell image) for NBDG and DHE was used to compensate for variation in cell size.

#### Glut1 membrane localization

Cells were gated for Glut1-positive cells (at least 10,000 cells in each population) using the intensity (sum of the background subtracted pixel values) and the Max Pixel (the largest value of the background subtracted pixels) features. In addition, only cells with membrane staining were further gated using the Max Contour Position (the location of the contour in the cell that has the highest intensity concentration mapped to a number between 0 and 1, with 0 being the object center and 1 being the object perimeter) and their percentage of the single, focused cells was calculated.

#### MitoGreen (MTG), MitoSOX and TMRE assessment

The Bright Detail Intensity feature was used (the intensity of localized bright spots within the image).

### Metabolomics analysis

For intracellular measurements, p53Mut-pMSCs and p53Mut-MSC-TLs were seeded at 10^6^ cells per 10 cm plate. At the following day, cells were washed and incubated in a glucose and glutamine free DMEM medium supplemented with 10% dialysed serum (dFCS; Biological Industries) and either 4mM L-Glutamine, (98% atom ^13^C5 95% (CP), Sigma 605166) or 10 mM D-Glucose, (U-^13^C6, 99% + , Cambridge Isotope Laboratories, Inc.) or the corresponding medium containing non-labelled L-glutamine and D-Glucose, under 5% O_2_ for 7 h. Subsequently, cells were washed with ice cold saline, lysed with 50% methanol in water and quickly scraped followed by three freeze thaw cycles in liquid nitrogen. The insoluble material was pelleted in a cooled centrifuge (4 °C) and the supernatant was collected for consequent GC-MS analysis. Samples were dried overnight under air flow at 42 °C using Techne Dry-Block Heater with sample concentrator (Bibby Scientific Limited, UK) and the dried samples were treated with 40 μl of a methoxyamine hydrochloride solution (20 mg/ml in pyridine) at 37 °C for 90 min while shaking followed by incubation with 70 μl N, O-Bis (trimethylsilyl) trifluoroacetamide (Sigma) at 37 °C for additional 30 min.

For extracellular labelled lactate measurements, cells were plated as described before. At the following day, cells were washed and incubated in a glucose and glutamine free DMEM medium supplemented with 10% dialysed serum, 4mM L-Glutamine and D-[1,6-^13^C]-glucose (CIL), for 2 h. 50 μl aliquots were collected at 0, 5, 15, 30, 60, 90, 120 min and briefly centrifuged to remove any debris. Next, from each aliquot 20 μl were taken into a 15 mL plastic tube, added by 17.9 nmol of Na-[^13^C_3_]-lactate (Sigma) serving as an internal standard. Next, 1 mL of methanol, chloroform (all GC/MS grade) and mQ water were added (total volume ~3 mL) shaked and centrifuged for 5 minutes at 2000 r.p.m. The upper aqueous phase were then collected and dried overnight under air flow at 42 °C as previously described. Next, samples were than incubated with 100 µL Tri-Sil (Pierce) under 42 °C for 30 min before loading onto the GC/MS. For the generation of a 3-point standard curve (0, 50, 100%), we used 1:500 dilutions of 20% w:v stock solutions of unlabeled sodium lactate and sodium L-[3-^13^C]-lactate which went through the same processes.

### Ex vivo metabolic analysis

Seven- to eight-week-old age NOD.CB17-prkdc-SCID/NCrHsd mice were injected subcutaneously with 3 × 10^6^ cells. Tumor growth was monitored weekly and mice were sacrificed when the tumors reached a diameter of 15 mm. Procedures were conducted in accordance with Institutional Animal Care and Use Committee of the Weizmann Institute of Science. After killling, tumors were removed and incubated in medium containing either 4mM L-Glutamine (98% atom ^13^C5 95% (CP), Sigma 605166), or 10 mM d-Glucose (U-^13^C6, 99%+, Cambridge Isotope Laboratories, Inc.), or in the corresponding medium containing non-labelled L-glutamine and D-Glucose under 5% O_2_ for 9 h. Next, cells were processed into single cell suspensions using a syringe plunger and a 40 µm steel strainer. Subsequently, cells were washed with ice cold saline, lysed with 50% methanol in water and quickly scraped followed by three freeze thaw cycles in liquid nitrogen. The insoluble material was pelleted in a cooled centrifuge (4 °C) and the supernatant was collected for consequent GC-MS analysis. Samples were dried under air flow at 42 °C using Techne Dry-Block Heater with sample concentrator (Bibby Scientific Limited, UK) and the dried samples were treated with 40 μl of a methoxyamine hydrochloride solution (20 mg/ml in pyridine) at 37 °C for 90 min while shaking followed by incubation with 70 μl N, O-Bis (trimethylsilyl) trifluoroacetamide (Sigma) at 37 °C for additional 30 min.

Gas chromatography/mass spectrometry (GC–MS) analysis was performed using a gas chromatograph (7820AN, Agilent Technologies, USA) interfaced with a mass spectrometer (5975 Agilent Technologies, USA). A HP-5ms capillary column 30 m × 250 µm × 0.25 µm (19091S-433, Agilent Technologies, USA) was used. Helium carrier gas was maintained at a constant flow rate of 1.0 mL min^−1^. The GC column temperature was programmed from 70 to 150 °C via a ramp of 4 °C min−1, 250–215 °C via a ramp of 9 °C min−1, 215–300 °C via a ramp of 25 °C min^−1^ and maintained at 300 °C for additional 5 min. The MS was by electron impact ionization and operated in full scan mode from *m*/*z*, 30–500. The inlet and MS transfer line temperatures were maintained at 280 °C, and the ion source temperature was 250 °C. Sample injection (1 μL) was in splitless mode.

### Total secreted lactate measurements by Nova chemical analyzer

Mut p53 pMSCs and Mut p53 MSC-TLs were seeded at 10^6^ cells per 10 cm plate and incubated in DMEM medium as indicated above under hypoxic conditions 5% O_2_ for one hour. Subsequently, 500 μL medium from the cell culture was collected, briefly centrifuged and directly injected into the analyzer. Background lactate measurements from cell free culture were subtracted.

### Total secreted lactate measurements by a colorimetric assay

Parental WT p53 and Mut p53 MSC were seeded at 2 × 10^5^ per 12-well plate. After 24 h cells were incubated for 3 h with DMEM without serum. Secreted lactate was measured analyzing growth media by colorimetric detection (Lactate Assay Kit; Cat. Number MAK064) following manufacturer’s guidelines (Sigma Aldrich). Lactate measurements were normalized on cell number.

### Transmission electron microscopy

All cell lines were fixed with 3% paraformaldehyde, 2% glutaraldehyde in 0.1 M cacodylate buffer containing 5 mM CaCl_2_ (pH 7.4), then postfixed in 1% osmium tetroxide supplemented with 0.5% potassium hexacyanoferrate tryhidrate and potasssium dichromate in 0.1 M cacodylate (1 h), stained with 2% uranyl acetate in water (1 h), dehydrated in graded ethanol solutions and embedded in Agar 100 epoxy resin (Agar scientific Ltd., Stansted, UK). Ultrathin sections (70–90 nm) were viewed and photographed with a FEI Tecnai SPIRIT (FEI, Eidhoven, Netherlands) transmission electron microscope operated at 120 kV and equipped with an EAGLE CCD Camera.

### Western blot

Cells pellets were lysed in TLB buffer (50 mM Tris-HCl, 100 mM NaCl, 1% Triton X-100, 0.5% sodium deoxycholate, 0.1% SDS) supplemented with Protease Inhibitor Cocktail (Sigma-Aldrich) for 15 min on ice and centrifuged for 15 min. Supernatants were analyzed for protein concentration using BCA reagent (Thermo-Scientific, Grand Island, NY, USA). In all, either 50 or 80 μg of protein extracts were boiled and loaded on 10–12% SDS-polyacrylamide gel. Proteins were transferred to a nitrocellulose membrane at semi-dry conditions. Membranes were blocked using 5% dry milk in PBST. The following primary antibodies were used: α-p53 (Cell Signalling, #2524), α-HIF-1α (Abcam), β-actin (Santa Cruz Biotechnology, Dallas, TX, USA, sc-47778). The ECL western blotting detection reagents were purchased from (Thermo Scientific), followed by analysis in ChemiDoc MP (Bio-Rad, Hercules, CA, USA).

### XF extracellular flux analyzer experiments

The XF96 extracellular flux analyzer (Seahorse Biosciences) was used to determine the bioenergetic profile of live cells. Cells were seeded (12,000 cells/well) in XF96 plates (Seahorse Biosciences) in the presence of CoCl_2_ (150 µM, 20 h) (Sigma Aldrich) and allowed to recover for 24 h. Cells were then incubated in bicarbonate-free DMEM (Sigma-Aldrich) supplemented with 10 mM glucose, 2 mM l-glutamine, 1 mM pyruvate (Sigma-Aldrich) in a CO_2_-free incubator for 1 h. The oxygen consumption rate (OCR) was recorded to assess the mitochondrial respiratory activity. After three measurements under basal conditions, cells were treated sequentially with 1 µM oligomycin, 2 µM carbonyl cyanide p-(trifluoromethoxy) phenylhydrazone (FCCP), and 0.5 mM rotenone plus 0.5 mM antimycin A (Sigma-Aldrich), with three consecutive determinations under each condition that were subsequently averaged. Raw data were normalized on cell number.

### Gene Set Enrichment Analysis (GSEA)

We utilized a previous RNA sequencing analysis of three p53Mut-pMSCs and ten derived p53Mut MSC TLs [10] and generated a pre-ranked list. We rank the gene list by numerical order according to a score determined by (Fold change)^4^/p-adj of genes expressed in p53Mut MSC TLs compared to their parental p53Mut-MSCs. The gene with the highest score was the first in the ranking list while the lowest score was the last gene in the ranked list. After generating the prerank list, we perform gene-set enrichment analysis on the pre-rank list and examined the enrichment of data-sets associated with the mitochondria.

### Real-time PCR

Total RNA from cells was isolated using the NucleoSpin RNA II kit (Macherey-Nagel, Duren, Germany). A concentration of 1 μg aliquot of the total RNA was reverse transcribed into cDNA using Bio-RT (Bio-Lab, Jerusalem, Israel), dNTPs and random hexamer primers. qRT-PCR was performed on Step One Plus, ABI instrument (Applied Biosystems, Grand Island, NY, USA) using SYBR Green PCR Master Mix (Quanta BioSciences, Gaithersburg, MD, USA). The values for the specific genes were normalized to HPRT housekeeping gene using the ΔΔCt method.

“qPCR analysis for mitochondrial DNA” has been published previously [13][14],[15],. Total DNA was isolated using Quick-gDNA MiniPrep (Zymo Research, Cat. No. D3007). Mitochondrial DNA (mtDNA) was quantified with SYBR green PCR Master Mix on Step One Plus, ABI instrument (Applied Biosystems, Grand Island, NY, USA). The relative mtDNA copy number was calculated from the subtraction of mtDNA copies to nuclear DNA copies. The relative fold change was then calculated based on the ΔΔCt method. Primers used in this study are shown in Table S3.

### Knockout of mutant p53 by CRISPR/Cas9 technology

Cells were transfected with Px330 backbone expressing sgRNA for targeting murine p53 and Cas9 by GeneExpresso^TM^ in vitro DNA transfection reagent (EG-1031, Excellgen). The Px330 p53 plasmid was a gift from Tyler Jacks (Addgene plasmid 59910). After 48 h from the infection, we plated 0.5 cell per well in a 96 wee plate in order to generate single cell clones. After the generation of single cell clones, we analyzed all cloned by western analysis in order to detect p53 knockout clones. 2 out of 71 clones were KO for mutant p53.

### Measurement of intracellular ATP levels

A total of 18,000 cells were seeded in 96-well plate. After 24 h, cells were incubated for 6 h with oligomycin 1 µM (Sigma Aldrich, 75351) and rotenone 1 µM (Sigma Aldrich, R8875). Intracellular ATP was measured by a luciferin/luciferase-based assay (ATP Bioluminiscence Assay Kit CLS II) following manufacturer’s guidelines (Roche).

### Side population

Cell staining was performed using the method described by Goodell et al[16]. with the following modification. Parental MSCs were incubated in the presence or absence of the ABC transporter inhibitor verapamil (100 µM) for 90 min at 37 °C in darkness with intermittent shaking. The cells were then washed with cold PBS, resuspended in PBS, and kept at 4 °C for flow cytometry analysis.

### Statistical analysis

Results are presented as mean ± SEM unless stated otherwise. Student’s *t* test was applied when statistic followed a normal distribution. For non-Gaussian distributions a Mann–Whitney test was applied. Multiple comparisons were performed with the one-way, two-sided ANOVA (Dunnet posthoc test). *p* values < 0.05 were considered statistically significant. All analysis was performed using GraphPad Prism.

## Results

### p53Mut regulates glucose and lactate metabolism of parental mouse MSCs

Our experimental model compared parental bone marrow-derived MSCs that express p53WT (p53WT-MSCs) with the corresponding adult MSCs that express R172H p53Mutation (p53Mut-MSCs) [9, 17]. Of note, adult stem cells generated by this methodology, represent a rather heterogeneous population of stem cells [18]. Parental p53Mut-MSCs exhibited a facilitated cell growth proliferation in vitro (Supplementary Fig. S1A), that correlated with the enhanced tumorigenicity measured by the capacity to develop slow-growing tumors in vivo [9, 17, 10] and showed a significantly higher presence of side population cells, indicating an increased number of cells with stemness-like characteristics within parental p53Mut-MSCs (Supplementary Fig. S1B, C).

**Fig. 1:**
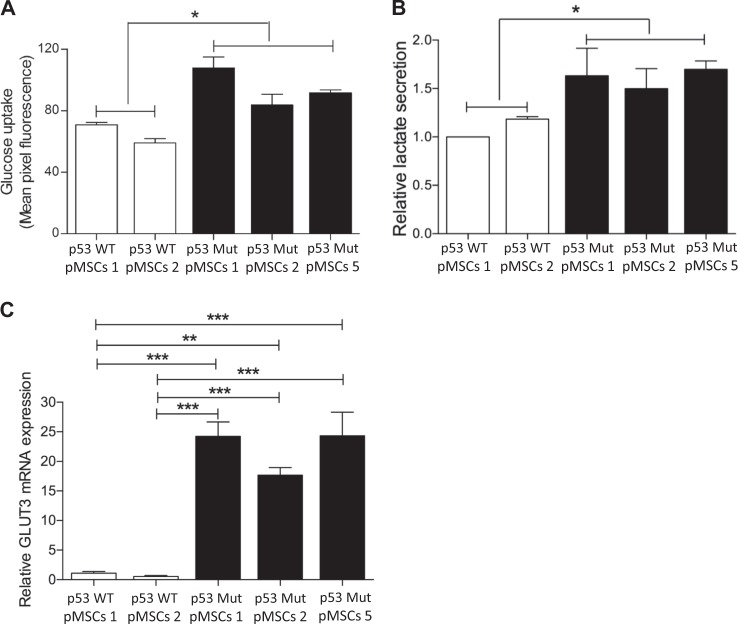
Mutant p53 regulates glucose and lactate metabolism of parental mouse MSCs Parental p53Mut MSCs were derived from mice homozygous for the murine R172H p53 mutation. Control parental p53WT MSCs were obtained from the corresponding p53WT mice. **a** Glucose uptake was evaluated by 2-NBDG incubation (50 µM, for 30 minutes) by Imaging Flow Cytometry (IFC). **b** Basal lactate secretion was evaluated assessing the lactate secreted by each isolate into the growth media. **c** Relative mRNA expression of GLUT3 in parental p53Mut MSCs compared with parental p53WT MSCs. Data are presented as mean ± SEM of at least three independent experiments. One-way, two-sided ANOVA. *p < 0.05, **p < 0.01, ***p < 0.001

To study whether increased tumorigenic potential of parental p53Mut-MSCs was accompanied by metabolic alterations, we compared the metabolic routes dominating in these cells with the corresponding parental p53WT-MSCs. Parental p53Mut-MSCs express higher levels of glucose uptake (Fig. 1a) and possess significantly increased lactate production compared to p53WT-pMSCs (Fig. 1b). In agreement, GLUT3 gene expression increased in p53Mut-pMSCs compared with parental p53WT-pMSCs (Fig. 1c). Likewise, glucose flux analysis in parental p53Mut-pMSCs showed a time-dependent augmented secretion of labeled 3-^13^C-lactate into the growth media (Supplementary Fig. S1D,E). Collectively, these results show that p53Mutant caused augmented glucose metabolism and tumorigenicity in stem cells, while p53WT is essential to preserve stem cell characteristics, including metabolism.

Consistent with previous findings [5], similar patterns were observed in p53-null H1299 cell lines and the corresponding p53Mut producers, as well as in mouse embryonic fibroblast derived from p53WT and p53Mut knock-in mice (Supplementary Fig. S1F-I).

### p53Mut-MSCs-derived tumor lines display enhanced metabolism

**Fig. 2:**
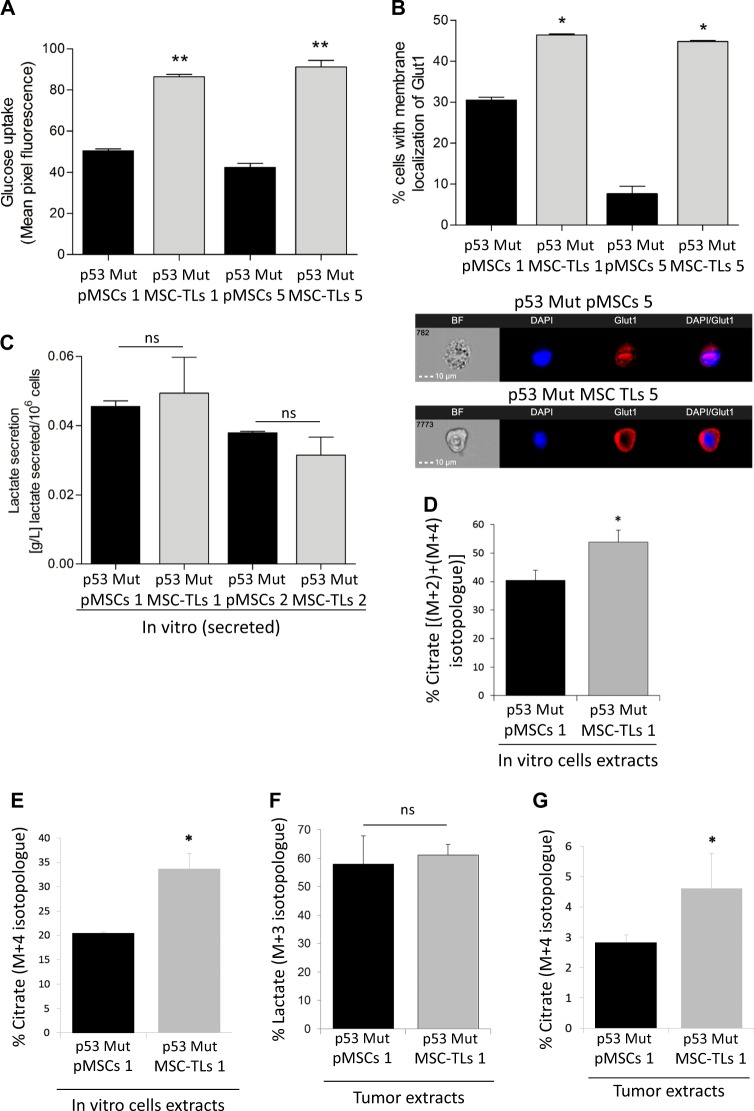
Mutant p53 MSCs-derived tumor lines display enhanced metabolism. **a** Glucose uptake was evaluated by 2-NBDG incubation (50 µM, for 30 minutes) by Imaging Flow Cytometry (IFC). **b** Localization levels of GLUT1 protein at the plasma membrane was evaluated by Imaging Flow Cytometry (IFC). **c** In vitro basal lactate secretion. **d**, **e** GC-MS measurements of cell extracts from p53Mut-pMSCs and p53Mut-MSC-TLs incubated for 7 h with RPMI containing 10 mM of uniformly labeled ^13^C-Glucose (**d**) or 4 mM of uniformly labeled ^13^C-Glutamine (**e**). Percentage of ^13^C enrichment for Citrate as % [(M + 2) + (M + 4) isotopologues] in (D) and % M + 4 isotopologue in (**e**). **f** GC-MS measurements of tumor extracts derived from p53Mut-pMSCs and p53Mut-MSC-TLs subcutaneously injected. Once established, tumors were isolated and incubated for 9 h with DMEM containing 10 mM of uniformly labeled ^13^C-Glucose. Percentage of ^13^C enrichment for lactate as % M + 3. A minimum of three mice per cohort was used. **g** GC-MS measurements of tumor extracts derived from p53Mut-pMSCs and p53Mut-MSC-TLs subcutaneously injected. Once established, tumors were isolated and incubated for 9 h with DMEM containing 4 mM of uniformly labeled ^13^C-Glutamine. Percentage of ^13^C enrichment for Citrate as % M + 4. A minimum of three mice per cohort was used. Data are presented as mean ± SD, except for mean ± SEM in (**a**, **b**) of three independent experiments. *p < 0.05, **p < 0.01. Paired Student’s *t* test

As described before, we generated highly oncogenic p53Mut-MSC-TLs derived from the p53Mut parental MSCs (pMSCs) by repeated passages in vivo and in vitro, generating well-controlled model of progressive malignancy with a specific Mutp53-dependent embryonic stem cell-like (ESC-like) gene signature [10]. The availability of these pairs of highly transformed tumor cell lines and their parental cells, prompted us to further examine whether this augmentation in oncogenesis is also reflected by different metabolic patterns. p53Mut-MSC-TLs displayed a significant enhancement in glucose uptake (Fig. 2a). Protein localization of glucose transporter Glut1 (Slc2A1) was significantly increased in p53Mut-MSC-TLs at the plasma membrane (Fig. 2b). However, no significant changes in levels of lactate secretion between p53Mut-MSC-TLs and p53Mut-pMSCs were observed (Fig. 2c). Therefore, we next examined whether these cells differ in mitochondrial metabolism. Using ^13^C-glucose tracing we measured the contribution of glucose to TCA metabolites and found an enrichment of ^13^C-citrate in p53Mut-MSC-TLs compared with p53Mut-pMSCs (Fig. 2d). Measurement of ^13^C-labelled glutamine also indicated enrichment of ^13^C-citrate synthesis in p53Mut-MSC-TLs compared to p53Mut-pMSCs (Fig. 2e). Although the total levels of citrate were not significantly different between p53Mut-MSC-TLs and p53Mut-pMSCs (Supplementary Fig. S2A, B), our data indicated specific enriched citrate mass isotopologues coming from either glucose or glutamine metabolism, that is a potential hallmark for changes in mesenchymal stemness. Though we found non-significant differences in lactate (M + 3) between p53Mut-MSC-TL1 and p53Mut-pMSC1 (Fig. 2f), we observed higher total lactate levels for the latter (Supplementary Fig. S2C). We also found the same significant trend in vivo showing higher citrate levels in p53Mut-MSC-TL1 as compared with p53Mut-pMSC1, labelled by 13C-glutamine (Supplementary Fig. S2D). These findings support different metabolism between p53Mut-pMSCs and p53Mut-MSC-TLs. To further validate our findings in vivo, NOD/SCID mice were injected subcutaneously with p53Mut-MSC-TLs and their corresponding p53Mut-pMSCs. Excised tumors were subjected to ex vivo stable ^13^C-glucose and ^13^C-glutamine labelling experiments, followed by GC/MS analysis. In agreement with the in vitro data, p53Mut-MSC-TLs primary tumors did not show any changes in the levels of intracellular lactate as compared to slow-growing p53Mut-pMSCs primary tumors (Fig. 2f), and yet displayed an enhanced glutamine-dependent synthesis of citrate (Fig. 2g). These results confirm that p53Mut-MSC-TLs exhibit augmented citrate levels both in vitro and in vivo.

These data indicates that Acetyl-CoA levels might be higher in p53Mut-MSC-TLs compared to p53Mut-pMSCs suggesting a mevalonate pathway upregulation, as previously reported [19, 20]. Nevertheless, no differences were found in the mRNA expression levels of genes involved in the mevalonate metabolism regulation (Supplementary Fig. S2E).

### p53Mut-MSC-TLs display increased mitochondrial mass

**Fig. 3:**
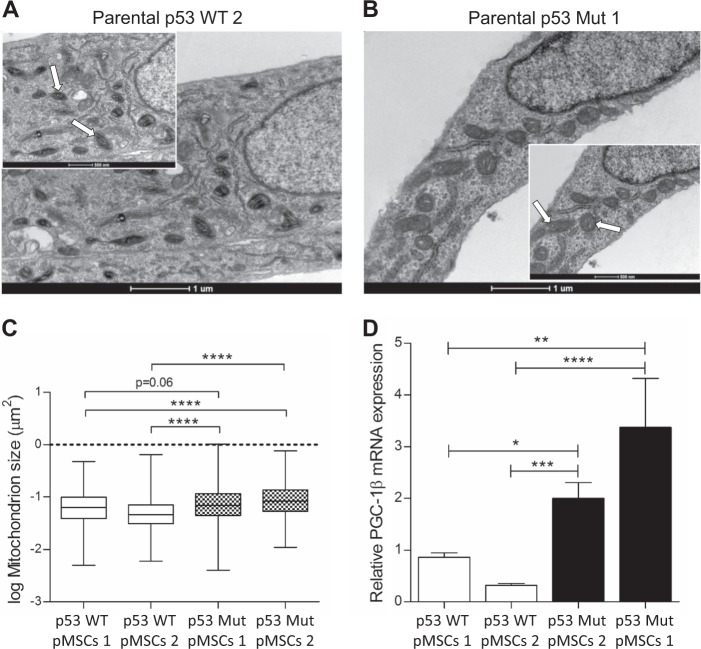
Parental Mutant p53 MSCs display increased mitochondrial mass compared to Parental WT p53 MSCs. **a**, **b** Representative EM micrographs (scale bar in big and small panels, 1 µM and 500 nm, respectively). Arrows indicates representative mitochondria. **c** Quantitation of the EM micrographs (at least 30 images per sample), show increased mitochondrial size in parental p53Mut Vs parental p53WT MSCs. One-way, two-sided ANOVA. **d** Relative mRNA expression of PGC1-β. Data are presented as mean ± SEM of at least three independent experiments. One-way, two-sided ANOVA. *p < 0.05, **p < 0.01, ***p < 0.001, ****p < 0.0001

Deregulation in mitochondrial metabolism and structure has been reported to be correlated with tumorigenesis and cancer progression [21]. To follow possible alterations in mitochondrial content and structure we performed transmission electron microscopy (TEM) analysis. First, we compared p53Mut parental MSCs with their corresponding p53WT parental MSCs to evaluate whether the expression of Mutp53 *per se*, independent of their tumorigenic phenotype, had an effect on mitochondria morphology. Mitochondria were significantly enlarged in p53Mut parental MSCs compared to p53WT parental MSCs (Fig. 3a–c). Accordingly, we found that mRNA expression of mitochondrial biogenesis master regulator PGC-1β was also increased in p53Mut parental MSCs (Fig. 3d).

**Fig. 4:**
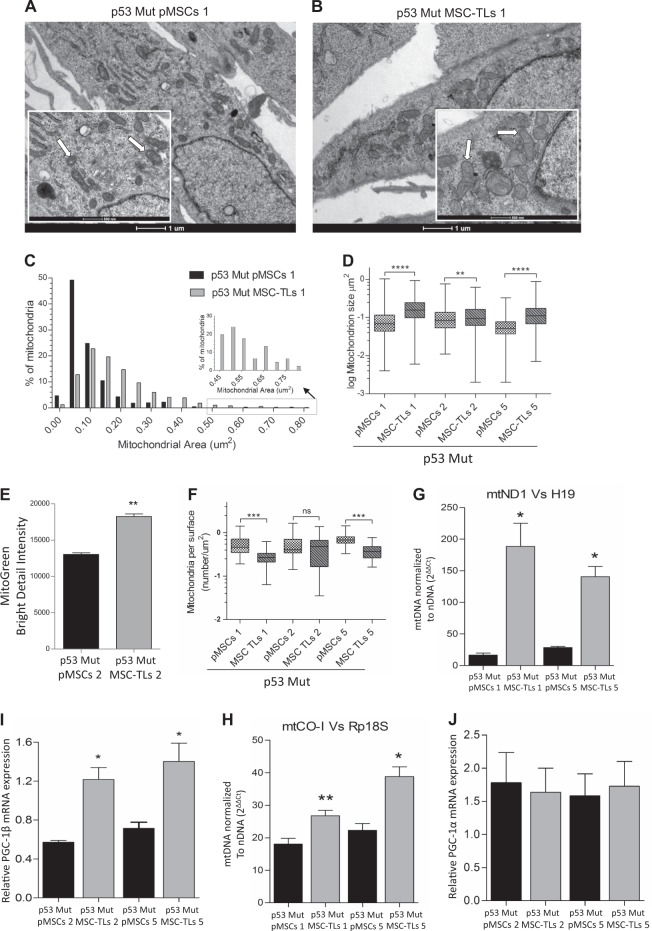
Mutant p53 MSC-TLs display increased mitochondrial mass. **a**, **b** Representative EM micrographs of p53Mut-MSC-TLs 1 and p53Mut-pMSCs1 (scale bar in big and small panels, 1 µM and 500 nm, respectively). Arrows indicates representative mitochondria. **c** Distribution of mitochondria areas in EM micrographs of p53Mut-MSC-TLs compared to p53Mut-pMSCs. **d** Quantitation of the EM micrographs (at least 30 images per sample), show increased mitochondrial size in p53Mut-MSC-TLs Vs p53Mut-pMSCs. Each p53Mut-MSC-TLs was compared to the p53Mut-pMSCs they were derived from (Mann–Whitney test). **e** MitoTracker Green (MTG) staining was evaluated by Imaging Flow Cytometry (IFC). Two-tailed paired Student’s *t* test. Three independent experiments were performed. (**f**) Mitochondrial density expressed as number of mitochondrial per area of cytoplasm (at least 30 images per sample). **g**, **h** Quantitative PCR analysis showed that mitochondrial DNA copy number normalized to nuclear DNA copy number (mtDN1 versus H19 and mtDN2 versus Mx1) was significantly increased in p53Mut-MSC-TLs Vs p53Mut-pMSCs. Two-tailed paired Student’s *t* test. Three independent experiments were performed. **i**, **j** Relative mRNA expression of PGC1-β (I) and PGC-1α (**j**) expression levels. Data are presented as mean ± SEM of at least three independent experiments. Data in (**d**, **f**) are presented as Whiskers plot (Min to Max), Mann–Whitney test. Data in (**e**, **g**–**j**) are presented as mean ± SEM of three independent experiments, Two-tailed paired Student’s *t* test, *p < 0.05, **p < 0.01, ***p < 0.001, ****p < 0.0001

Next, we investigated whether p53Mut-MSC-TLs exhibit further change in mitochondrial content when compared to p53Mut-pMSCs. Individual mitochondria of p53Mut-MSC-TLs1 were significantly enlarged compared to p53Mut-pMSCs1 (Fig. 4a, b; Supplementary Fig. S4A, B). Quantitative comparison of area distribution indicated that p53Mut-MSC-TLs mitochondria are larger than those in p53Mut-pMSCs (Fig. 4c). Statistical analysis confirmed this increment in the mitochondria size to be significant (Fig. 4d, *p* < 0.0001, *p* < 0.01 Mann–Whitney test). Increment in mitochondria mass was assessed by Mitogreen fluorescence levels (Fig. 4e). Additionally, the number of mitochondria per µm [2] of cytoplasm was reduced (Fig. 4f). p53Mut-MSC-TLs1 and p53Mut-MSC-TLs5 also showed a significant increase in mitochondrial DNA copy number as assessed by mtND1 and H19 (Fig. 4g) and mtCO-1 and Rp18S (Fig. 4h) compared to their correspondent p53Mut-pMSCs. p53Mut-pMSCs2 and p53Mut-MSC-TLs2 (Supplementary Fig. S4C-G), as well as p53Mut-pMSCs5 and p53Mut-MSC-TLs5 (Supplementary Fig. S4H-L) yielded similar patterns as observed with p53Mut-pMSCs1 and p53Mut-MSC-TLs1. mRNA expression of mitochondrial biogenesis master regulator PGC-1β was also increased in p53Mut-MSC-TLs (Fig. 4i), consistently with the differences observed in Mutp53-pMSCs vs WTp53-MSCs (Fig. 3d). We did not detect any significant change in PGC-1α expression levels (Fig. 4j). Together these data show a significant increase of mitochondrial mass in p53Mut-MSC-TLs as compared to the corresponding p53Mut-pMSCs.

### p53Mut-MSC-TLs show increased mitochondrial oxidative metabolism

**Fig. 5:**
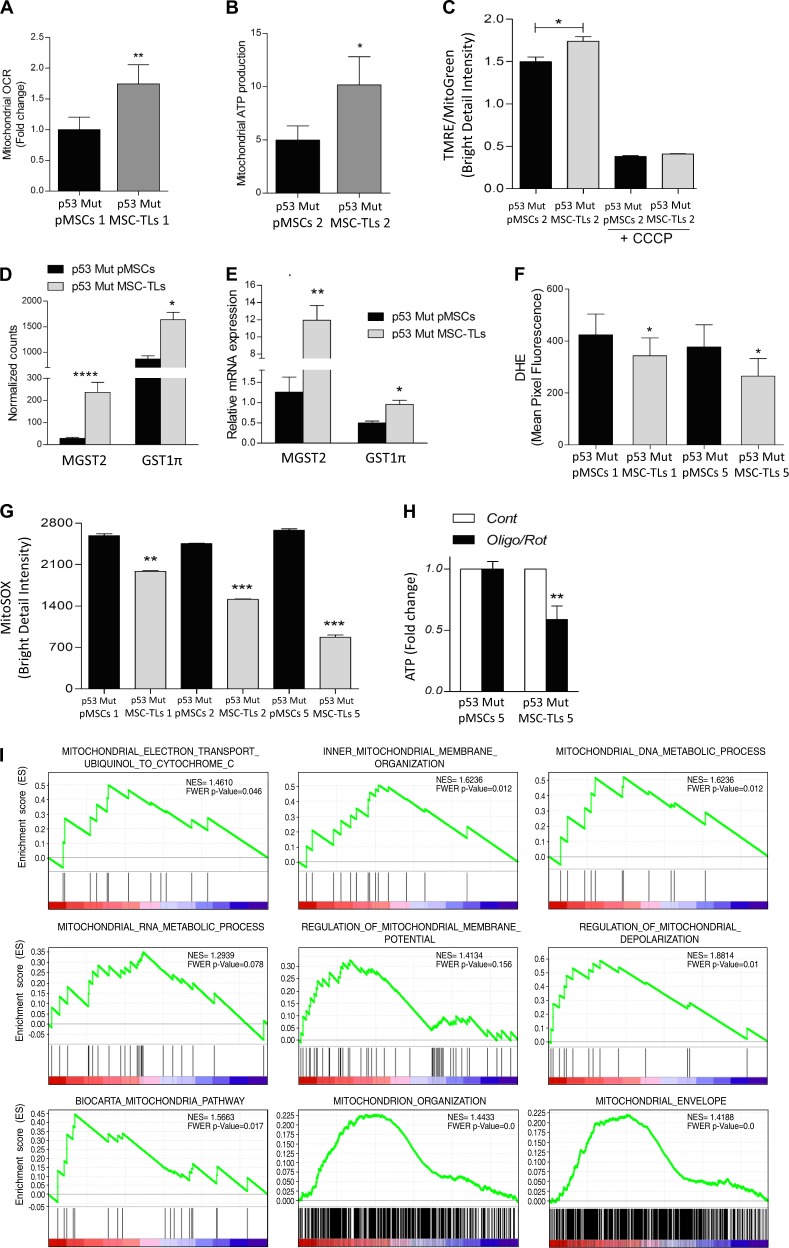
Mutant p53 MSC-TLs display increased mitochondrial oxidative metabolism **a** Basal Oxygen Consumption Rate (OCR) was determined using XFe96 Extracellular Flux Analyzer comparing p53Mut-MSC-TLs cultures vs. p53Mut-pMSCs. Results are normalized on cell number. **b** Mitochondrial ATP production (or ATP turnover) is considered as the oligomycin-sensitive respiration. **c** Increased mitochondrial membrane potential (TMRE staining) in p53Mut-MSC-TLs versus p53Mut-pMSCs assessed by Imaging Flow Cytometry (IFC). **d** Transcriptional analysis of p53Mut-pMSCs and p53Mut-MSC-TLs by RNA sequencing. Mitochondrial glutathione S-transferase 1π (GST1π) and MGST2 normalized counts. **e** Relative mRNA expression of GST1π and MGST2. **f** Ione superoxide was evaluated by DHE incubation (5 µM, for 20 minutes) by Imaging Flow Cytometry (IFC). **g** Ione superoxide was additionally evaluated by MitoSOX incubation (5 µM, for 40 minutes) by Imaging Flow Cytometry (IFC). **h** ATP content after 6 h of treatment with Oligomycin (1 µM) + Rotenone (0.5 µM). **i** Plots of gene distribution from GSEA analysis of RNA sequencing data generated in [10]. Highly, significant enriched gene-sets in p53Mut-MSC-TLs vs. p53Mut-pMSCs are shown here. In every thumbnail, the green curve represents the enrichment profile of the genes identified in the RNA-seq; red and blue colours represent positive and negative correlation, respectively. FWER *p* < 0.25 and NES are indicated in the plots. Data are presented as mean ± SEM of at least three independent experiments. Data in (**d**) are presented as mean ± SEM of normalized counts determined by RNA sequencing. ****p < 0.0001, **p < 0.01, *p < 0.05. Two-tailed Student’s *t* test

To examine whether alterations in the mitochondrial mass of p53Mut-MSC-TLs is implicated in mitochondrial function, we compared oxidative phosphorylation (OXPHOS) in p53Mut-pMSCs vs. p53Mut-MSC-TLs. To mimic the physiological oxygen tension used throughout this work, we exposed cells to the chemical hypoxia-mimicking agent, CoCl_2_ [22]. Indeed, this treatment led to HIF-1α protein stabilization (Supplementary Fig. S5A). Under such conditions, the basal OCR was significantly higher in p53Mut-MSC-TLs compared to p53Mut-pMSCs (Fig. 5a). MSC-TLs also exhibited a concomitant higher level of oligomycin-sensitive respiration that is associated to increased mitochondrial ATP production (Fig. 5b).

Accordingly, tetramethylrhodamine, ethyl ester (TMRE), which indicates increased mitochondrial membrane potential [23], showed a significant increased staining in p53Mut-MSC-TLs (Fig. 5c). TMRE bright detail intensity was normalized on Mitotracker Green. As negative control, we used the OXPHOS chemical inhibitor carbonyl cyanide m-chlorophenyl hydrazone (CCCP) which shows the presence of mitochondrial transmembrane potential.

Mitochondria are the main source for intracellular Reactive Oxygen Species (ROS) in the form of O_2_. (anione superoxide), which may affect the efficiency of mitochondrial respiration. A physiological pathway to overcome excessive ROS production is to enhance antioxidant pathways [24]. We utilized our previously described computational transcriptome analysis to compare p53Mut-pMSCs and their corresponding p53Mut-MSC-TLs [10]. This analysis indicated that p53Mut-MSC-TLs exhibited a higher expression of Microsomal Glutathione S-Transferase2 (MGST2) and glutathione S-transferase pi1 (GST1π) (Fig. 5d). These genes are associated with the antioxidant response to ROS [25]. Data were confirmed by qRT-PCR (Fig. 5e). Using the mitochondrial ROS-sensitive probe DHE, we measured a significant reduction in mitochondrial ROS production in the form of O_2_. in p53Mut-MSC-TLs (Fig. 5f). We confirmed this finding by further assessing mitochondrial ROS with MitoSOX (Fig. 5g). p53Mut-MSC-TLs displayed a significant reduced mitochondrial oxidative stress as compared to their correspondent p53Mut-MSCs. This suggests that p53Mut-MSC-TLs have a lower production of mitochondrial ROS and a decreased basal oxidative stress compared to p53Mut-pMSCs.

To evaluate the OXPHOS contribution to ATP synthesis in p53Mut-pMSCs and p53Mut-MSC-TLs, we measured total ATP levels upon treatment with mitochondrial inhibitors, oligomycin and rotenone. The treatment resulted in a significant drop in ATP levels indicating augmented energy crisis in p53Mut-MSC-TLs (Fig. 5h). On the other hand, p53Mut-pMSCs showed a negligible response. This effect was also reflected by downregulation of stem cell markers Sox2 and CD44 gene expression (Supplementary Fig. S5B), typical members of the ESC-like gene signature. Next, we tested whether gene expression related to mitochondrial metabolism in p53Mut-MSC-TLs showed any systematical change compared to their correspondent p53Mut-pMSCs. In order to do that, we adopted the Gene Set Enrichment Analysis (GSEA) computational approach [26]. By analyzing a previous RNA sequencing dataset [10] comparing the p53Mut-MSC-TLs with their corresponding p53Mut-pMSCs by GSEA, we found nine sets of genes involved in fundamental mitochondrial function significantly enriched in the aggressive p53Mut-MSC-TLs (Fig. 5i). These gene sets are involved in mitochondrial metabolic pathways that account for “mitochondrial electron transport ubiquinol to cytochrome C”, “mitochondrial inner membrane organization”, “mitochondrial DNA metabolic process”, “mitochondrial RNA metabolic process”, “regulation of mitochondrial membrane potential” and “regulation of mitochondrial depolarization”. Furthermore, we found three additional gene sets specifically enriched in the p53Mut-MSC-TLs and included general mitochondrial-related gene signatures such as “mitochondria pathway”, “mitochondrion organization” and “mitochondrial envelope”. These signatures included additional genes relevant for OXPHOS, nutrients transport across the mitochondria, mitochondrial ribosomal proteins, apoptosis, mitochondrial DNA metabolism and several others mitochondria-related genes. Finally, we performed an unsupervised GSEA on the full-curated gene sets (Table S1, http://software.broadinstitute.org/gsea/msigdb/genesets.jsp?collection=C2).

Overall, p53Mut-MSC-TLs, show increased OXPHOS and reduced mitochondrial oxidative stress. p53Mut-MSC-TLs also show dependence on OXPHOS for ATP synthesis, as inhibition of OXPHOS is leading to a significant drop in ATP content. Finally, p53Mut-MSC-TLs also show a systematic change in the expression pattern of multiple genes involved in fundamental metabolic mitochondrial pathways.

### Regulation of mitochondrial mass and activity is p53Mut-dependent

**Fig. 6:**
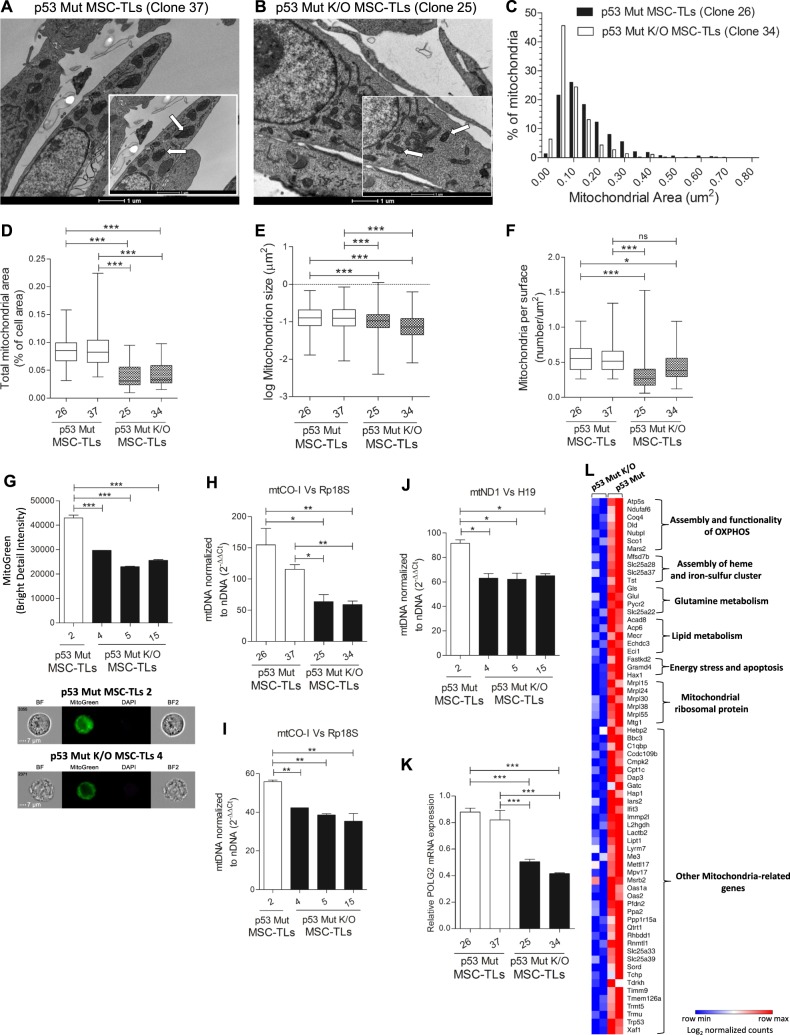
Regulation of mitochondrial mass and activity is Mutant p53-dependent. **a**, **b** Representative EM micrographs (scale bar in big and small panels, 1 µM ×9900 and 1 µM ×16,500, respectively). Arrows indicates representative mitochondria. **c** Distribution of mitochondria areas in EM micrographs of p53Mut-MSC-TLs compared to p53Mut K/O MSC-TLs. **d** Quantitation of the EM micrographs show decreased mitochondrial area (as percentage of cell area) in p53Mut K/O MSC-TLs versus p53Mut-MSC-TLs. **e** Quantitation of the EM micrographs show decreased mitochondrial size in p53Mut K/O MSC-TLs vs. p53Mut-MSC-TLs. **f** Decreased mitochondrial density (number of mitochondria per cell area) in p53Mut K/O MSC-TLs vs. p53Mut-MSC-TLs. **g** Mito Tracker Green (MTG) staining was evaluated by Imaging Flow Cytometry (IFC). **h**–**j** Quantitative PCR analysis of mitochondrial DNA copy number normalized to nuclear DNA copy number (mtCO-I versus Rp18S and mtND1 vs. H19). **k** Relative mRNA expression of POLG2. **l** Heat map presenting the mitochondrial gene signature that was downregulated following KO of p53Mut in MSC-TLs. Log2 normalized counts are shown. Data are presented as Whiskers plot (Min to Max). Data in (**g**–**k**) are presented as mean ± SEM of at least three independent experiments. One-way, two-sided ANOVA. *p < 0.05, **p < 0.01, ***p < 0.001

To further determine whether the increase in mitochondrial mass and function observed in Mut p53 MSC-TLs is Mutp53-dependent, we used p53Mut knockout (K/O) MSC-TLs generated by CRISPR/Cas9 gene editing system [10] (Supplementary Fig. S6A). We have already reported that p53Mut K/O in the MSC-TLs induces a reduction in the malignant phenotype of the p53Mut-MSC-TLs [10]. Using TEM, we observed that K/O of p53Mut in p53Mut-MSC-TLs results in a significant reduction in mitochondria size (Fig. 6a, b). Comparative area distribution of mitochondrial occupancy of p53Mut-MSC-TLs (clone 26) with p53Mut K/O MSC-TLs (clone 34) indicated a decrease in the latter (Fig. 6c). Furthermore, a decrease in total mitochondrial area (as per cell area) (Fig. 6d), size of individual mitochondria (Fig. 6e), as well as the number of mitochondria (Fig. 6f) was observed in p53Mut K/O MSC-TLs. By Mitotracker Green staining we also observed a significant decrease in mitochondrial mass of p53Mut K/O MSC-TLs compared to p53Mut-MSC-TLs (Fig. 6g). This was accompanied by a significant decrease in mitochondrial DNA copy number (Fig. 6h–j). Accordingly, RNA levels of mitochondrial DNA polymerase gamma2 (POLG2) were decreased in p53Mut K/O MSC-TLs (Fig. 6k). Furthermore, p53Mut K/O MSC-TLs exhibited a reduction in protein localization of glucose transporter GLUT1 at the plasma membrane, compared to controls (Supplementary Fig. S6B). Consistent with our previous findings [10], K/O of p53Mut led to a dramatic reduction in the expression levels of Sox2 gene (Supplementary Fig. S6C).

We utilized our previous RNA sequencing analysis [10] to compare p53Mut MSC TLs with the corresponding p53Mut K/O MSC-TLs. Functional annotation analysis (DAVID Bioinformatics Resources 6.8, NIAID/NIH) revealed a gene signature, consisting of 68 genes, associated with the Mitochondrion that was downregulated in p53Mut K/O MSC-TLs (Fig. 6l). This 68 genes signature consisted of several defined groups of genes that are involved in the regulation of key mitochondrial functions, related to OXPHOS assembly and activity of respiratory complexes such as ATP5S [27], Ndufafs6 [28], Sco1 [29] and Nubpl[30],. Additional groups of genes that were significantly downregulated upon p53Mut K/O included several factors that mediates the assembly of Heme and iron-sulfur clusters, such as protein-bound redox cofactors essential for electron transfer [31], genes encoding for multiple mitochondrial ribosomal proteins that are responsible for the translation of mitochondrial encoded genes [32], as well as enzymes implicated in glutamine and lipid metabolism, and genes implicated in the apoptotic response. For the full list of genes that were downregulated upon knocking out mutant p53 see ×Table S2 and ref. 10. We also detected an annotation cluster of genes (cluster number 6) associated with the Mitochondrion (Benjamini 3.24E-6, FDR 1.17E-4) among other annotation clusters. These results demonstrate that the oncogenic p53Mut plays a central role in coordinating both mitochondrial mass and the expression of multiple genes associated with fundamental mitochondrial metabolic processes in p53Mut-MSC-TLs.

Of note, metformin, a well-tolerated antidiabetic drug that induce anti-proliferative signals in LFS patients [33], also showed a similar effect on the aggressive p53Mut-MSC-TLs. Indeed, we performed a proliferation assay in absence or presence of Metformin using p53Mut-MSC-TLs and Mutp53 K/O MSC-TLs. As shown in Supplementary Fig. S6D, Mutp53 expressing MSC-TLs are sensitive to Metformin treatment, while Mutp53 K/O MSC-TLs display a reduced sensitivity to Metformin treatment. Similarly, mitochondrial inhibition by using oligomycin and rotenone, led to a significant induction of apoptotic cell death markers in p53Mut-MSC-TLs compared to their corresponding p53Mut-pMSCs as assessed by Annexin V and PI expression, as well as nuclear fragmentation index (× Supplementary Fig. S6E). We next examined whether K/O of p53Mut may also affect the sensitivity of p53Mut-MSC-TLs to OXPHOS inhibition mediated by oligomycin and rotenone. p53Mut K/O MSC-TLs displayed a diminished induction of apoptotic cell death upon OXPHOS inhibition as compared to p53Mut producers MSC-TLs. This was accompanied by a significant reduction of Annexin V^+^ cells (× Supplementary Fig. S6F). K/O of p53Mut partially rescues the dependence of p53Mut-MSC-TLs on OXPHOS inhibition, demonstrating that this metabolic vulnerability of p53Mut-MSC-TLs relies on the concomitant expression of p53Mut.

Our data suggest that cancer stem cells at different transformed status use different metabolic pathways. Non-transformed p53WT parental cells seem to support their metabolism by glucose uptake and lactate secretion. These cells exhibit a normal mitochondria structure and size. The more aggressive p53Mut parental cells show an augmented glucose uptake, lactate secretion and altered features in the mitochondrial structure. p53Mut-MSC-TLs further exhibited modified mitochondrial structure and metabolism. Thus, suggesting that stem cells at different state of malignancy are also associated with a different quantitative and qualitative metabolic profile in a p53Mut-dependent manner.

## Discussion

The possibility that traits of stemness in cancer cells may account for initiating a given tumor and/or to drive its evolution has been extensively studied [34, 35, 36, 37]. Both normal adult stem cells (ASCs) and somatic cells that underwent aberrant dedifferentiation, have been suggested to be potentially cells of origin in cancer [38, 39]. Nevertheless, the theory of ASCs as the origin of cancer cells is more parsimonious [40, 41, 42]. Malignant transformation is a multi-step process that is unique for each individual tumor type and involves most likely cancer initiating stem-like cells that are followed by several defined steps finally leading towards the establishment of highly transformed metastatic cells.

Metabolism is a very complex system of physiological pathways that support different cellular states. Metabolic alterations are associated with carcinogenesis [1, 2]. Cancer cells exhibit aerobic glycolysis that leads to an increased glucose metabolism and enhanced production of lactate in the tumor cells [3, 4, 5]. Given this complexity, the goal of our study was to explore whether modulation in specific metabolic routes may be associated with defined steps in the development of malignancy.

Interestingly, p53WT represses glycolysis and regulates mitochondrial respiration [43, 44, 45, 46, 47]. On the other hand, p53Mut was suggested to stimulate aerobic glycolysis through promoting GLUT1 translocation to the plasma membrane [5]. Members of the Li–Fraumeni syndrome families, carrying germline mutations in *p53* gene, were shown to have an increased mitochondrial function and enhanced OXPHOS [48, 33, 49].

p53Mut may regulate cancer stemness via alteration of the mevalonate pathway [19, 20]. Nevertheless, the role of p53Mut-induced metabolic reprogramming in cancer cells with stemness properties is still to be uncovered.

Recently, we have developed a p53-dependent stem cell system where a step-wise process of malignancy can be identified [9, 17, 10]. While p53Mut parental MSCs display enhanced proliferation, self-renewal and increased tumorigenic potential in the form of slow-growing sarcomas, the p53WT parental MSCs did not [9, 10]. Furthermore, p53Mut parental MSCs aggressive-derived tumor lines (p53Mut-MSC-TLs) form tumors by the injection of as low as 100 cells and express a signature consisting of ESCs-like genes [10].

By comparing p53WTpMSCs with p53MutpMSCs we found that the latter exhibit a significant increased glucose uptake and lactate secretion. Our data suggest that p53Mut-pMSCs perform aerobic glycolysis. Furthermore, p53Mut-MSC-TLs express yet a higher level of glucose uptake. However, no change in lactate secretion was observed when the p53Mut-MSC-TLs were compared to their corresponding p53Mut-pMSCs line of origin. This preferential increase in glucose uptake with no change in lactate secretion suggested the possibility that p53Mut-MSC-TLs have selectively preferred the usage of glucose for mitochondrial-dependent pathways as the synthesis of citrate. Citrate synthase activity was shown to strongly correlate with mitochondrial content [50]. Indeed, the increased citrate synthesis observed in p53Mut-MSC-TLs correlated with an increase in mitochondrial mass. The most pronounced changes between the various tumor cell lines of a different malignant status were manifested in the structure and function of the mitochondria. Thus, these changes can be regarded as a hallmark for the p53Mut-dependent progression of malignant transformation. These findings are supported by computational analysis, which identified multiple gene sets enriched in p53Mut-MSC-TLs related to mitochondrial functions. Therefore, we suggest a correlation between the aggressiveness of p53Mut-MSC-TLs with systematic changes in the regulation of mitochondrial metabolism.

This is greatly substantiated by the observation that cancer cells with stemness properties enhance their mitochondrial metabolism and dependence on OXPHOS [51, 52, 53, 54]. Mitochondria are indeed emerging as key regulators of cancer stemness [54, 55]. Specifically, mutant KRAS pancreatic cancer cells contain a CD133^+^/Mito^high^ subpopulation of cells resistant to KRAS ablation that also exhibit enhanced mitochondrial metabolism and augmented OXPHOS dependency [55]. Similarly, human pancreatic mutant KRAS/CD133^+^ cancer cells derived from PDX models are sensitive to mitochondrial inhibition that leads these cells with stemness properties to apoptosis [54].

A key factor that determines the efficiency of OXPHOS is the production of mitochondrial ROS. In stem cells, oxidative stress resulting from the production of ROS is associated with several cellular processes such as differentiation and impairment of mitochondrial integrity [24, 56]. We found that increased mitochondrial OCR in p53Mut-MSC-TLs correlated with a significant reduction in the production of mitochondrial ROS. This was accompanied by the increased expression of antioxidant genes, suggesting that the aggressive p53Mut-MSC-TLs may benefit from reducing oxidative stress in order to increase the efficiency of OXPHOS.

p53Mut K/O in p53Mut-MSC-TLs that correlates with a reduction in their malignant phenotype [10], also reduced the augmented unique metabolic characteristics of these cells. This confirms that metabolic changes observed in the various stem cells are indeed p53Mut-dependent. K/O of p53Mut in p53Mut-MSC-TLs led to a decrease in size and number of mitochondria, significantly affecting mitochondrial content and activity. This was also translated into the reduced localization of Glut1 at the plasma membrane and a dramatic reduction of Sox2 gene expression.

Interestingly, in a comparative computational transcriptome analysis, we identified a unique mitochondrial gene signature consisting of 68 genes involved in the various mitochondria activity and are downregulated upon p53Mut K/O in p53Mut-MSC-TLs. The fact that the abrogation of p53Mut in the aggressive MSC-TLs had a dramatic effect on mitochondrial mass and on the expression of the mitochondrial gene signature further supports our conclusion that p53Mut is a master regulator of mitochondrial metabolism in cancer stem cells.

The observation that tumor cells at a defined transformation status represent unique metabolic patterns, may offer important prognostic tools in determining personalized designed cancer therapy. Tumor expressing a highly modified mitochondrial activity should be seen as more aggressive tumors and treated accordingly. Moreover, modified metabolic pathways can be abolished by known suitable inhibitors [33] and can be used directly for personalized metabolic targeted therapy.

## Electronic supplementary material

5 supplementary figures

Legend supplementary figures

Supplementary table 1

Supplementary table 2

Supplementary table 3

Supplementary figure legends
