# Supplementary material for: Mutant p53-dependent mitochondrial metabolic alterations in a mesenchymal stem cell-based model of progressive malignancy
Source: Cell Death Differ. 2018 Nov 9;26(9):1566–81. doi: 10.1038/s41418-018-0227-z (PMC6748146; doi:10.1038/s41418-018-0227-z)
Supplement: Supplementary file 1 — 5 supplementary figures [file 41418_2018_227_MOESM1_ESM.pdf]

Figure S1

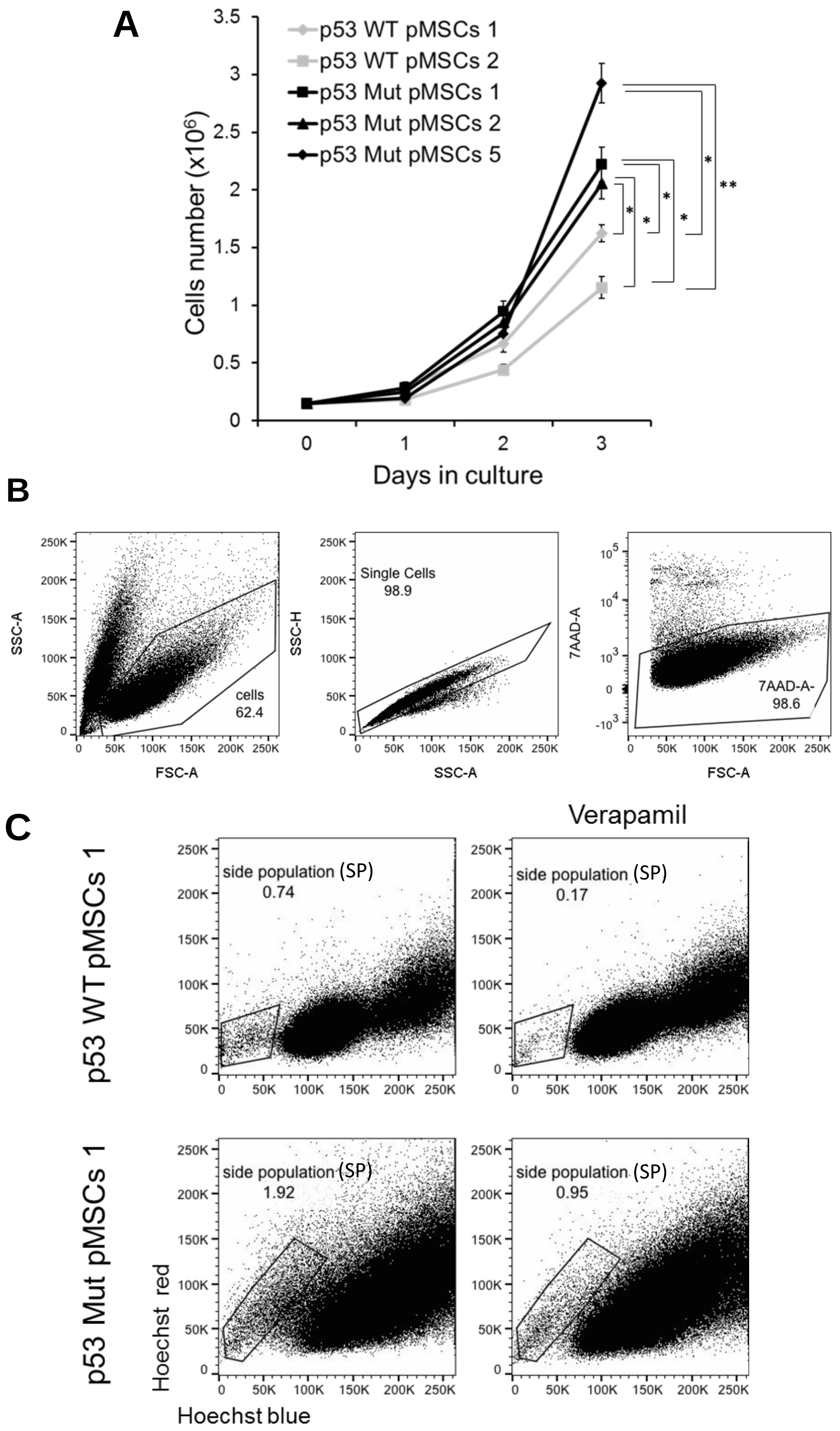

A
p53 WT pMSCs 1
p53 WT pMSCs 2
p53 Mut pMSCs 1
p53 Mut pMSCs 2
p53 Mut pMSCs 5
Cells number (x10^6)
Days in culture
B
cells 62.4
SSC-A
FSC-A
Single Cells 98.9
SSC-H
SSC-A
7AAD-A- 98.6
7AAD-A
FSC-A
C
Verapamil
p53 WT pMSCs 1
side population (SP) 0.74
side population (SP) 0.17
p53 Mut pMSCs 1
side population (SP) 1.92
side population (SP) 0.95
Hoechst red
Hoechst blue

Figure S1

D

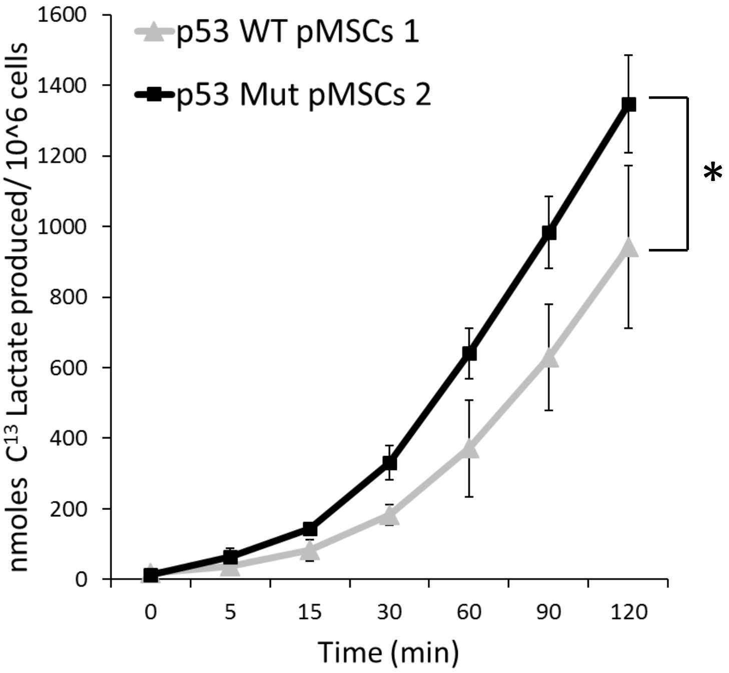

p53 WT pMSCs 1
p53 Mut pMSCs 2
nmoles C13 Lactate produced/ 10^6 cells
1600
1400
1200
1000
800
600
400
200
0
*
0
5
15
30
60
90
120
Time (min)

E

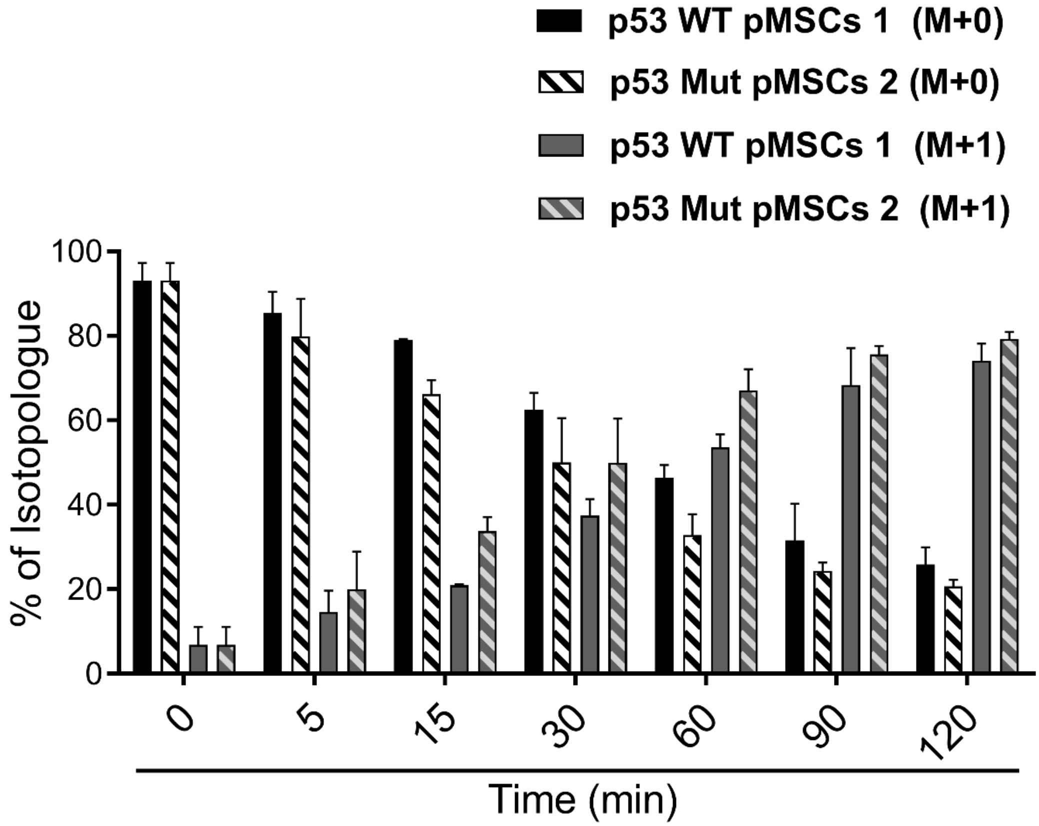

p53 WT pMSCs 1 (M+0)
p53 Mut pMSCs 2 (M+0)
p53 WT pMSCs 1 (M+1)
p53 Mut pMSCs 2 (M+1)
% of Isotopologue
100
80
60
40
20
0
0
5
15
30
60
90
120
Time (min)

Figure S1

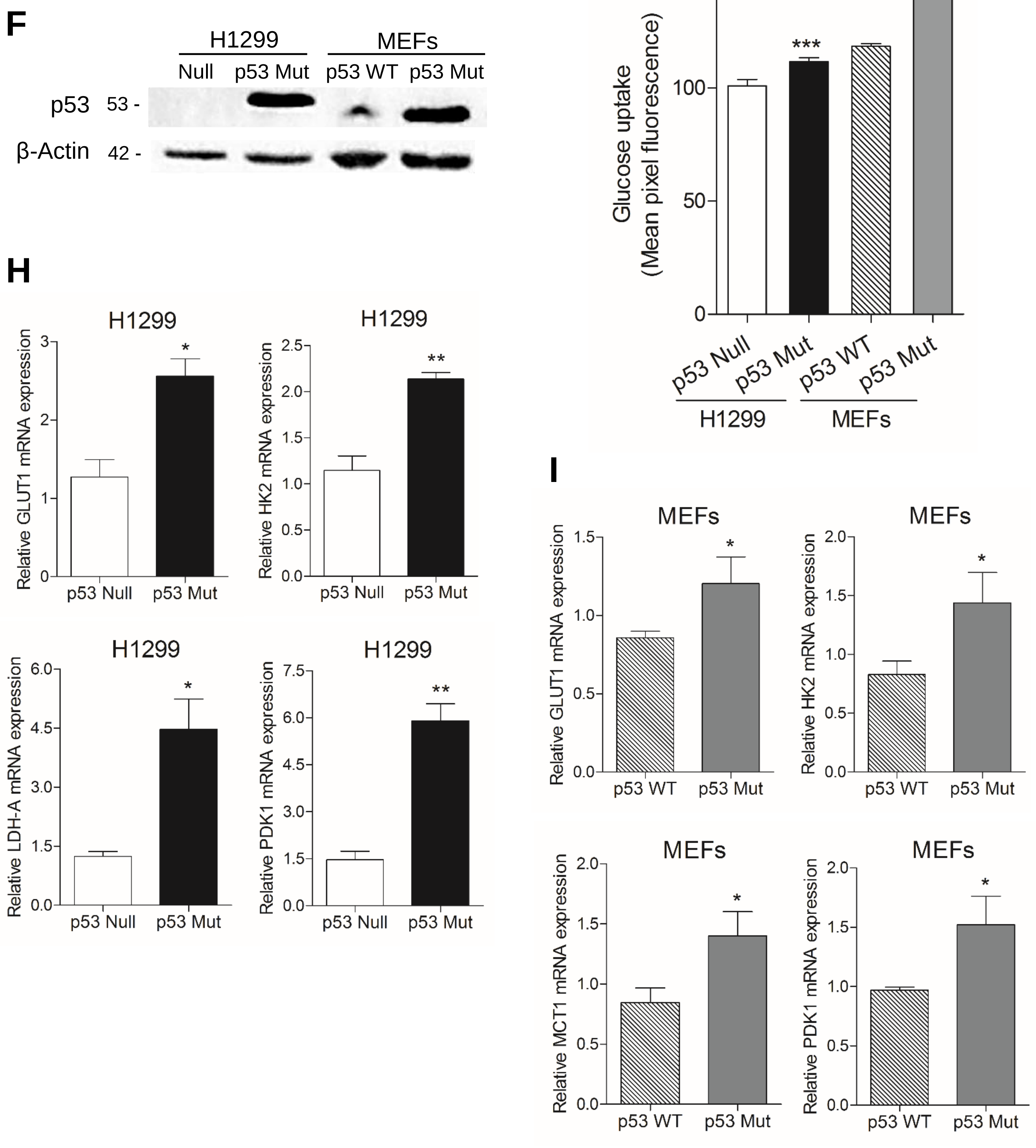
F
H1299
MEFs
Null
p53 Mut
p53 WT
p53 Mut
p53
53 -
β-Actin
42 -
G
Glucose uptake
(Mean pixel fluorescence)
150
100
50
0
***
*
p53 Null
p53 Mut
p53 WT
p53 Mut
H1299
MEFs
H
H1299
Relative GLUT1 mRNA expression
p53 Null
p53 Mut
*
H1299
Relative HK2 mRNA expression
p53 Null
p53 Mut
**
H1299
Relative LDH-A mRNA expression
p53 Null
p53 Mut
*
H1299
Relative PDK1 mRNA expression
p53 Null
p53 Mut
**
I
MEFs
Relative GLUT1 mRNA expression
p53 WT
p53 Mut
*
MEFs
Relative HK2 mRNA expression
p53 WT
p53 Mut
*
MEFs
Relative MCT1 mRNA expression
p53 WT
p53 Mut
*
MEFs
Relative PDK1 mRNA expression
p53 WT
p53 Mut
*

Figure S2

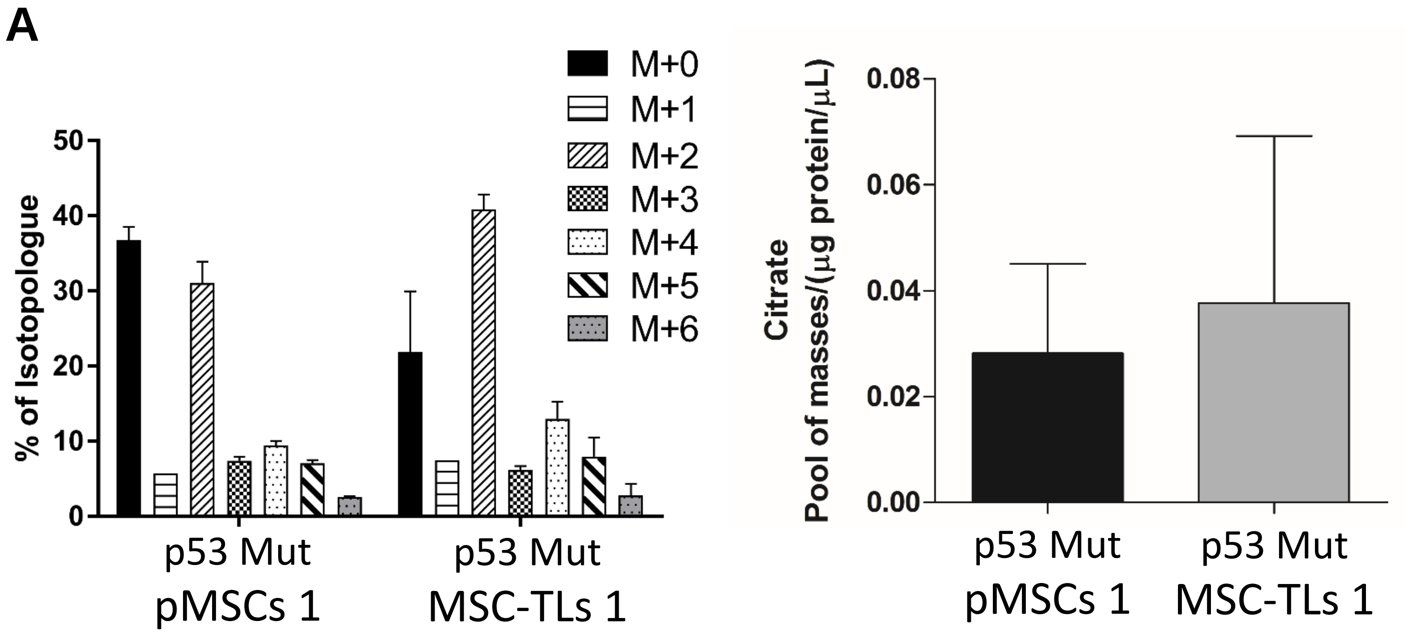
A
M+0
M+1
M+2
M+3
M+4
M+5
M+6
% of Isotopologue
p53 Mut
pMSCs 1
p53 Mut
MSC-TLs 1
Citrate
Pool of masses/(μg protein/μL)
p53 Mut
pMSCs 1
p53 Mut
MSC-TLs 1

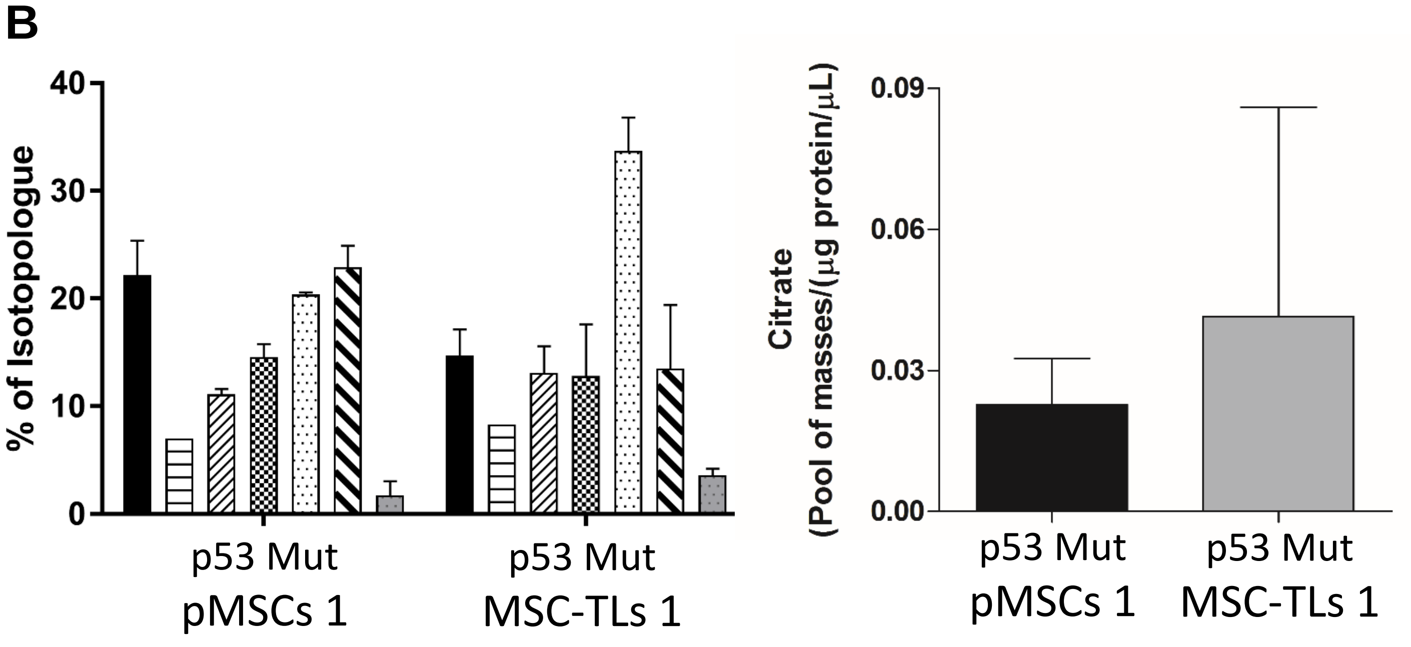
B
% of Isotopologue
p53 Mut
pMSCs 1
p53 Mut
MSC-TLs 1
Citrate
(Pool of masses/(μg protein/μL)
p53 Mut
pMSCs 1
p53 Mut
MSC-TLs 1

Figure S2

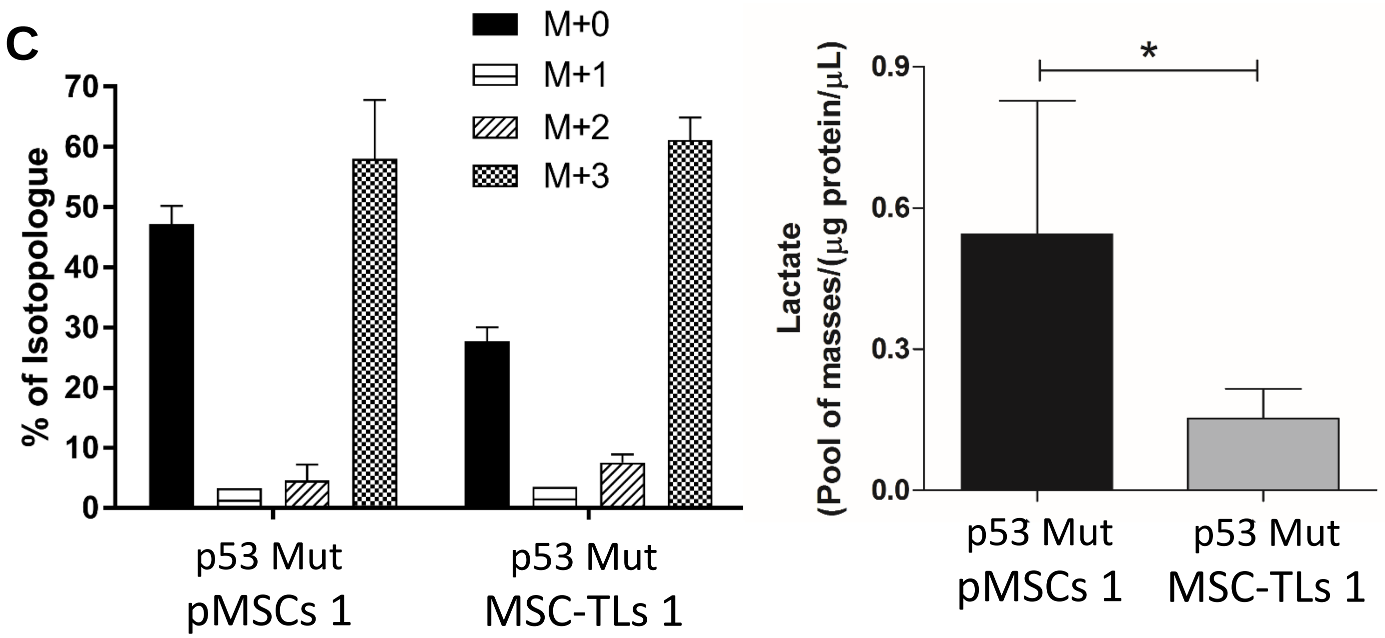
C
M+0
M+1
M+2
M+3
% of Isotopologue
70
60
50
40
30
20
10
0
p53 Mut pMSCs 1
p53 Mut MSC-TLs 1
Lactate (Pool of masses/(μg protein/μL)
0.9
0.6
0.3
0.0
*
p53 Mut pMSCs 1
p53 Mut MSC-TLs 1

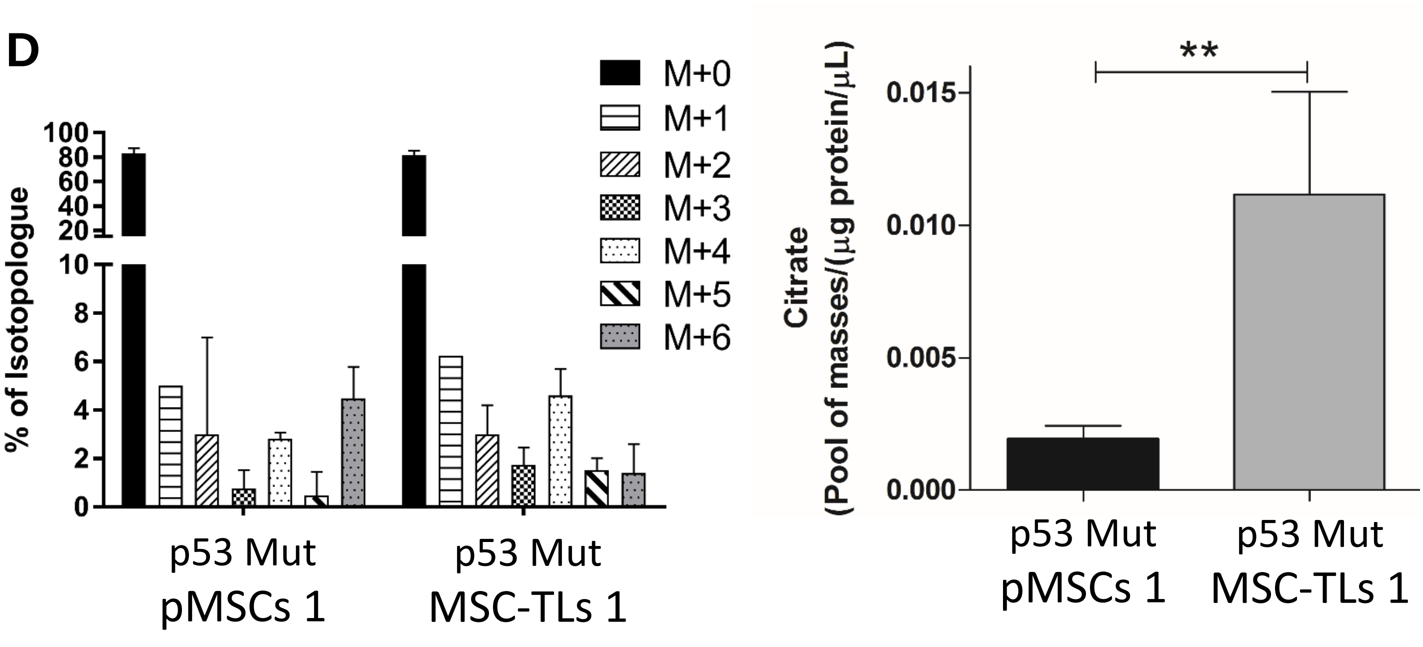
D
M+0
M+1
M+2
M+3
M+4
M+5
M+6
% of Isotopologue
100
80
60
40
20
10
8
6
4
2
0
p53 Mut pMSCs 1
p53 Mut MSC-TLs 1
Citrate (Pool of masses/(μg protein/μL)
0.015
0.010
0.005
0.000
**
p53 Mut pMSCs 1
p53 Mut MSC-TLs 1

Figure S2

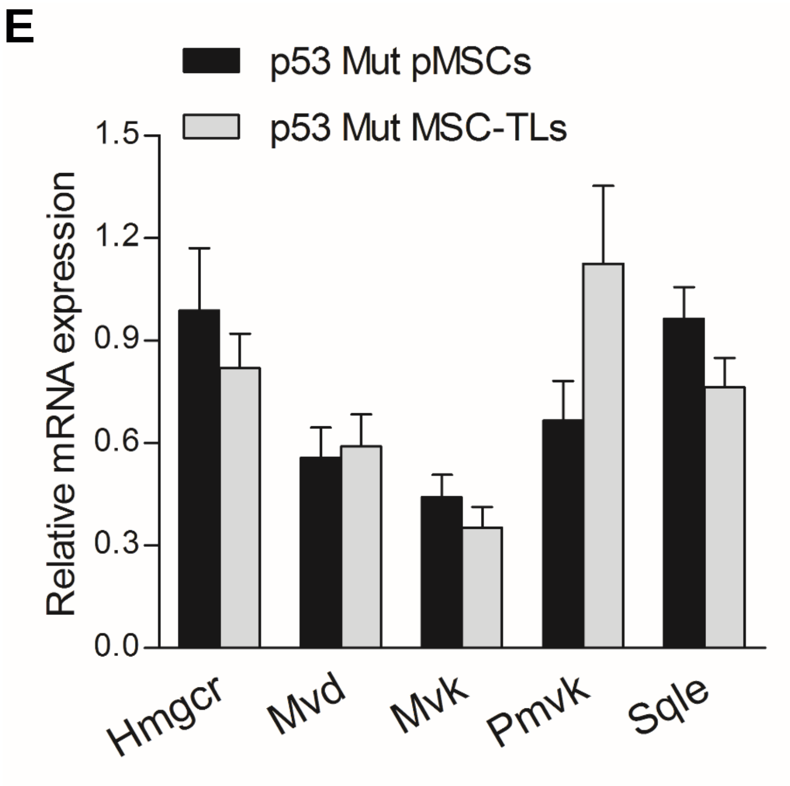
E
p53 Mut pMSCs
p53 Mut MSC-TLs
Relative mRNA expression
1.5
1.2
0.9
0.6
0.3
0.0
Hmgcr
Mvd
Mvk
Pmvk
Sqle

Figure S4

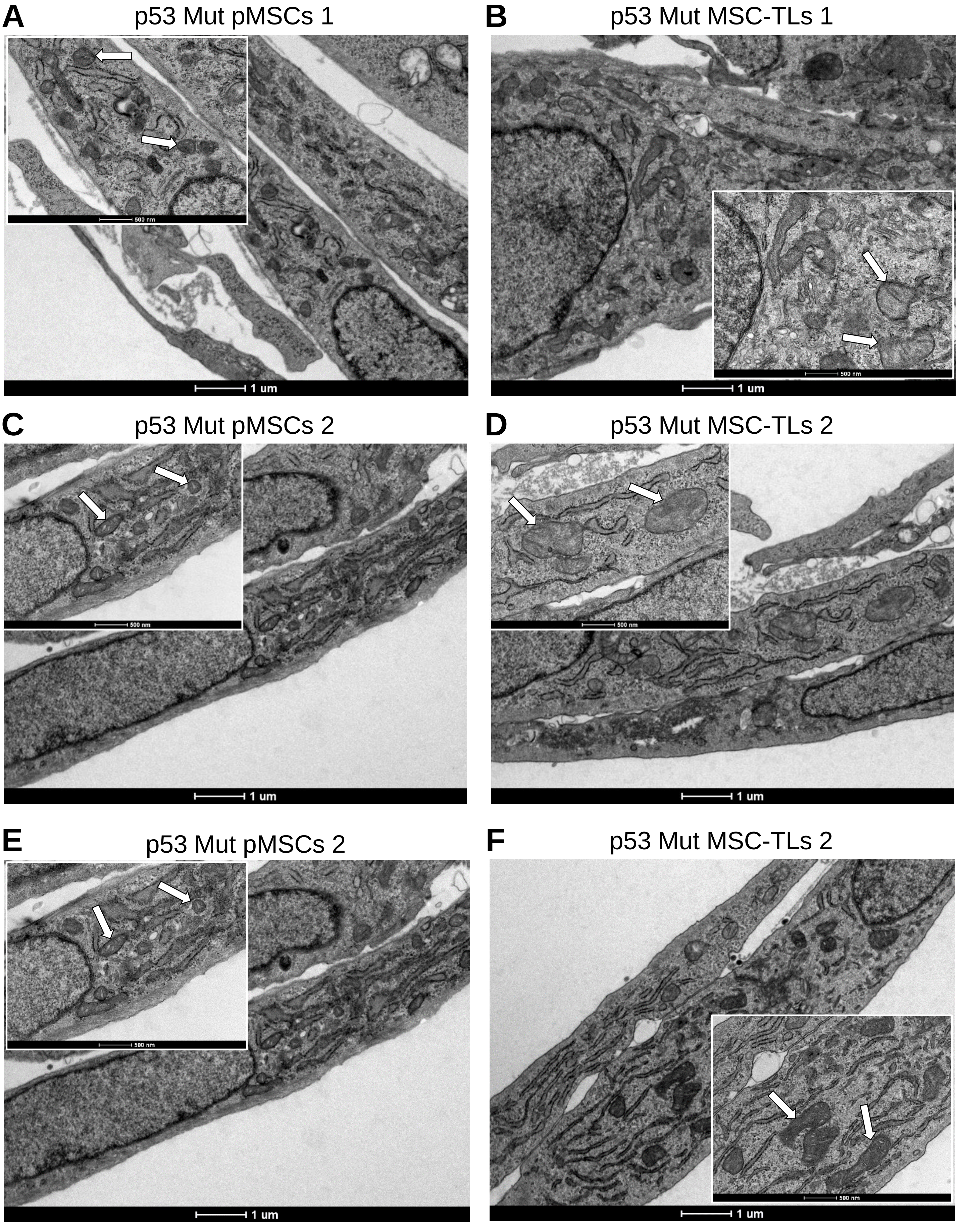
A
p53 Mut pMSCs 1
500 nm
1 um
B
p53 Mut MSC-TLs 1
500 nm
1 um
C
p53 Mut pMSCs 2
500 nm
1 um
D
p53 Mut MSC-TLs 2
500 nm
1 um
E
p53 Mut pMSCs 2
500 nm
1 um
F
p53 Mut MSC-TLs 2
500 nm
1 um

Figure S4

G

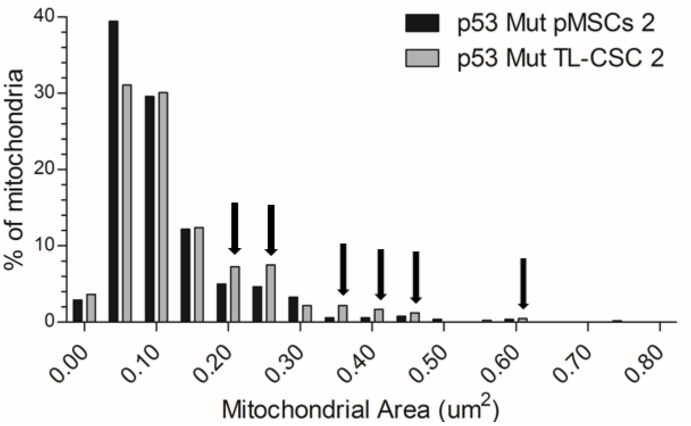

H

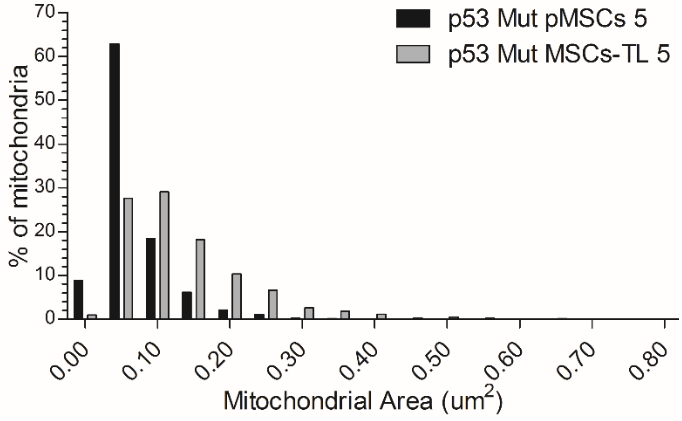

I p53 Mut pMSCs 5

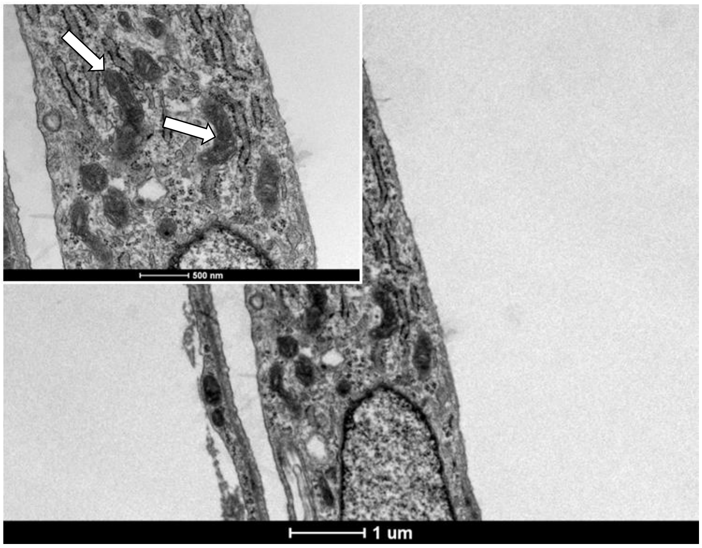

J p53 Mut MSC-TLs 5

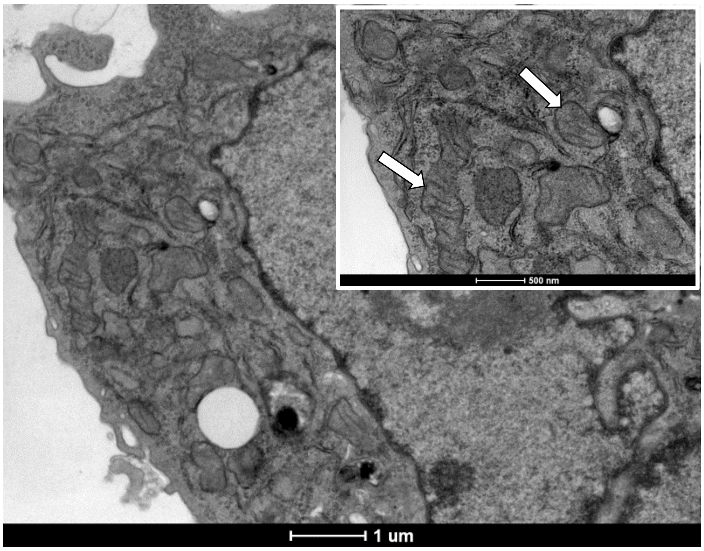

K p53 Mut pMSCs 5

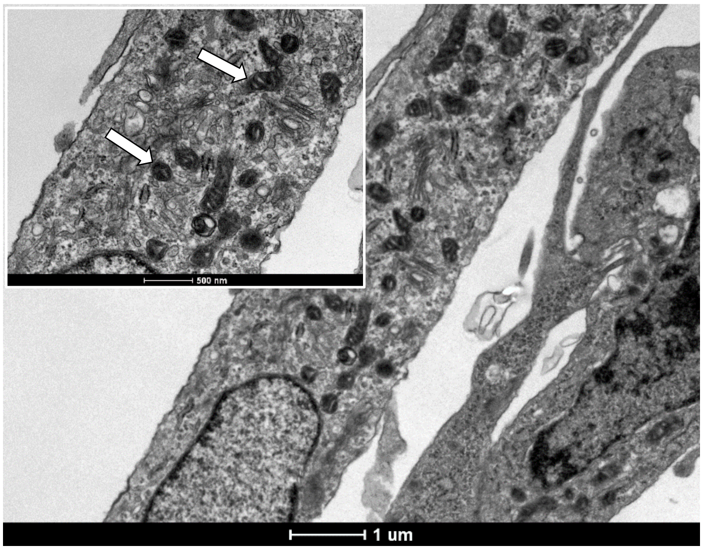

L p53 Mut MSC-TLs 5

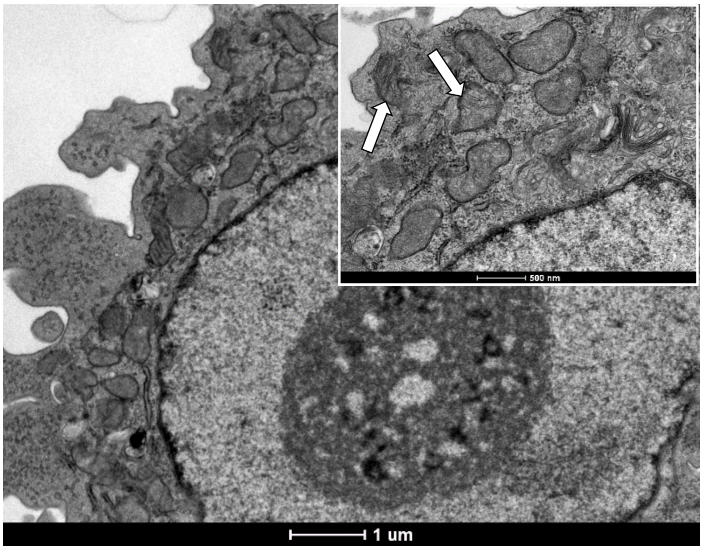

Figure S5

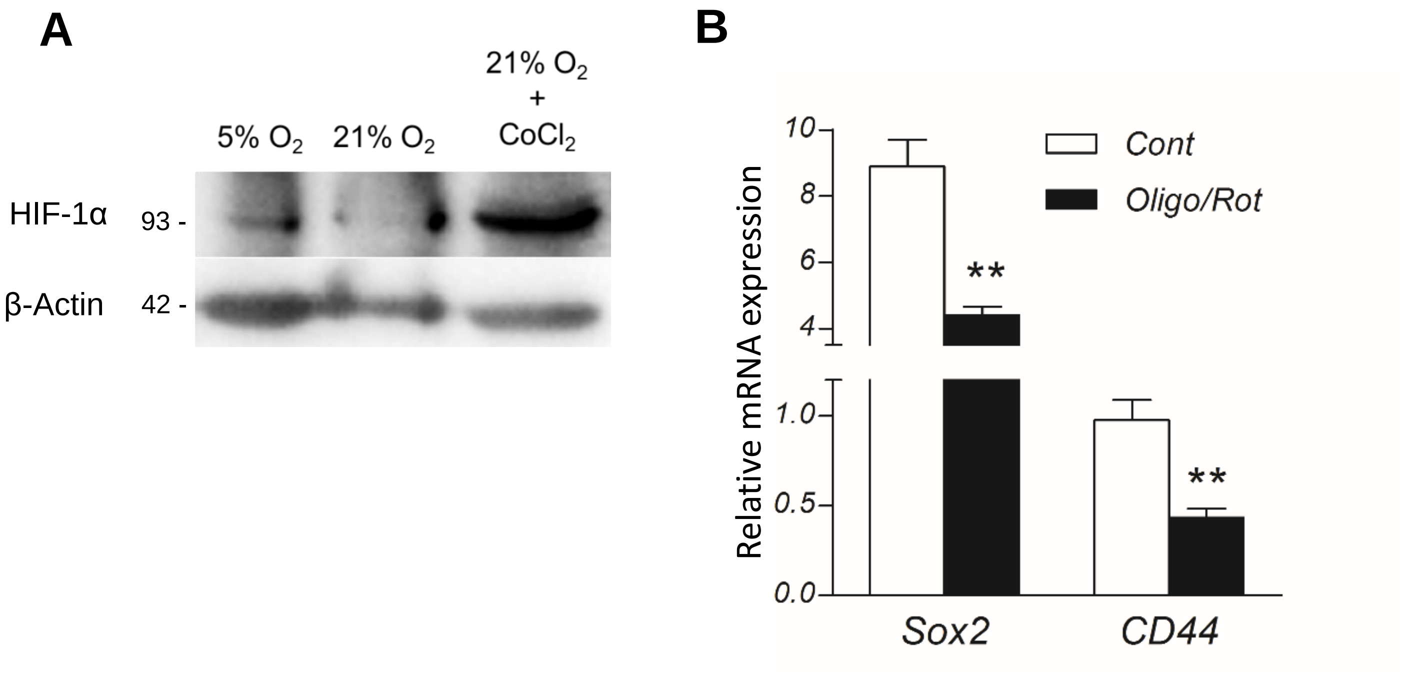
A
5% O2
21% O2
21% O2 + CoCl2
HIF-1α
93 -
β-Actin
42 -
B
Relative mRNA expression
10
8
6
4
1.0
0.5
0.0
Cont
Oligo/Rot
**
**
Sox2
CD44

Figure S6

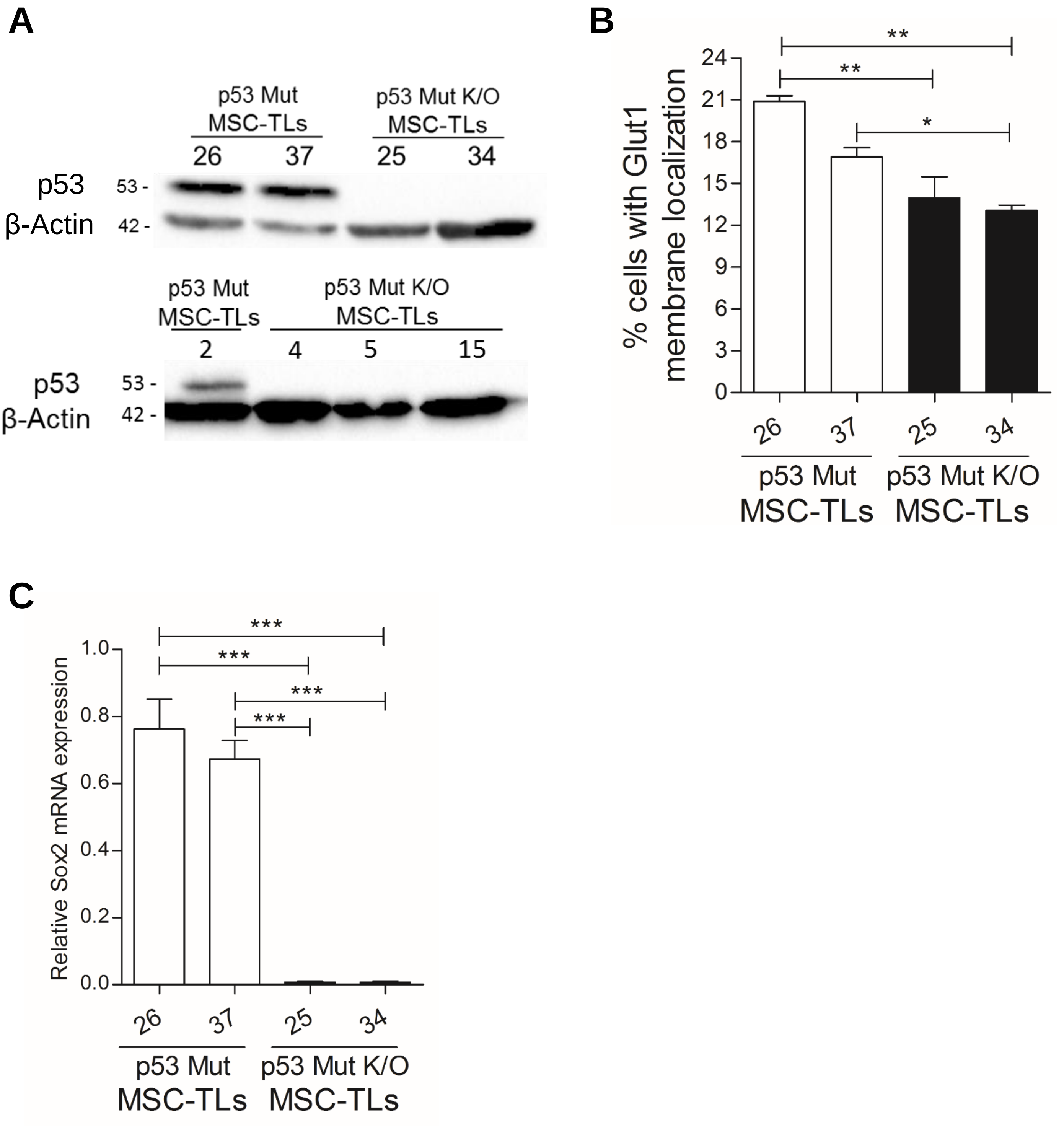
A
p53 Mut
MSC-TLs
26
37
p53 Mut K/O
MSC-TLs
25
34
p53
β-Actin
53 -
42 -
p53 Mut
MSC-TLs
2
p53 Mut K/O
MSC-TLs
4
5
15
p53
β-Actin
53 -
42 -
B
% cells with Glut1 membrane localization
0
3
6
9
12
15
18
21
24
**
**
*
26
37
25
34
p53 Mut MSC-TLs
p53 Mut K/O MSC-TLs
C
Relative Sox2 mRNA expression
0.0
0.2
0.4
0.6
0.8
1.0
***
***
***
***
26
37
25
34
p53 Mut MSC-TLs
p53 Mut K/O MSC-TLs

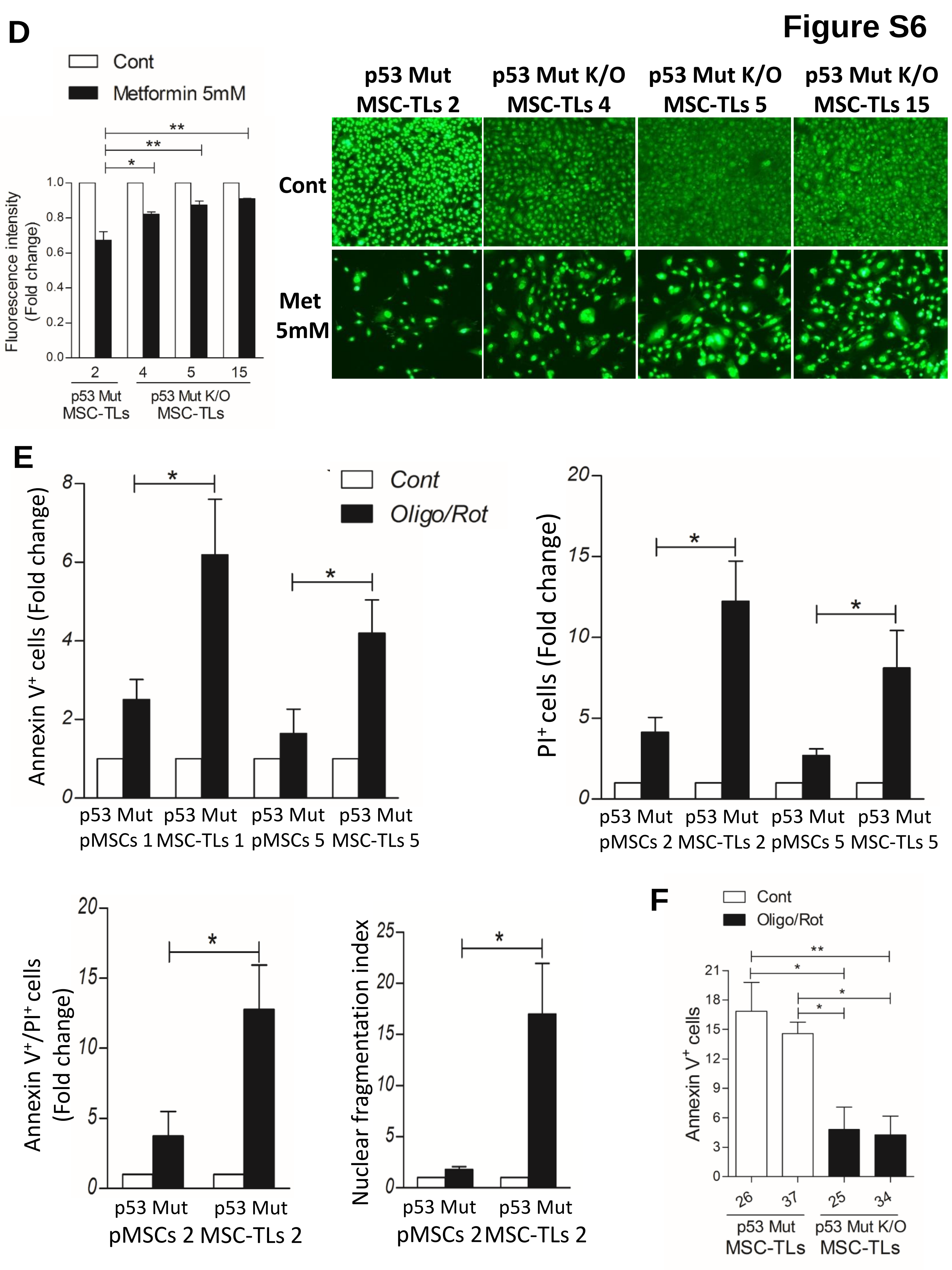
Figure S6
D
Cont
Metformin 5mM
Fluorescence intensity (Fold change)
p53 Mut MSC-TLs
p53 Mut K/O MSC-TLs
p53 Mut MSC-TLs 2
p53 Mut K/O MSC-TLs 4
p53 Mut K/O MSC-TLs 5
p53 Mut K/O MSC-TLs 15
Cont
Met 5mM
E
Cont
Oligo/Rot
Annexin V+ cells (Fold change)
p53 Mut pMSCs 1
p53 Mut MSC-TLs 1
p53 Mut pMSCs 5
p53 Mut MSC-TLs 5
PI+ cells (Fold change)
p53 Mut pMSCs 2
p53 Mut MSC-TLs 2
p53 Mut pMSCs 5
p53 Mut MSC-TLs 5
Annexin V+/PI+ cells (Fold change)
p53 Mut pMSCs 2
p53 Mut MSC-TLs 2
Nuclear fragmentation index
p53 Mut pMSCs 2
p53 Mut MSC-TLs 2
F
Cont
Oligo/Rot
Annexin V+ cells
26
37
25
34
p53 Mut MSC-TLs
p53 Mut K/O MSC-TLs
