# Supplementary material for: Mutant p53-dependent mitochondrial metabolic alterations in a mesenchymal stem cell-based model of progressive malignancy
Source: Cell Death Differ. 2018 Nov 9;26(9):1566–81. doi: 10.1038/s41418-018-0227-z (PMC6748146; doi:10.1038/s41418-018-0227-z)
Supplement: Supplementary file 2 — Legend supplementary figures [file 41418_2018_227_MOESM2_ESM.pdf]

Supplementary Figure legends

Figure S1. Related to Figure 1

The p53-null H1299 cells were compared with their counterpart stably expressing the R175H p53 Mut protein. Likewise, MEFs derived from the R172H p53 mutation knock-in mice, as well as control MEFs obtained from the corresponding WTp53 mice were compared.

(A) Proliferation assay. $2x10^5$ cells were plated at day 0 and counted after 1, 2 and 3 days in culture. Cells were counted in duplicate at each time point. At least three experiments were performed.

(B) Gating strategy for SP identification.

(C) Hoechst 33342 staining of parental p53 WT and parental p53 Mut MSCs. Verapamil was used to inhibit SP formation (right hand panel). Location of SP is indicated by gate. Dot plots are representative of 2 independent experiments.

(D) 3-^{13}C-lactate secretion over time assessed by stable isotope D-[1,6-^{13}C]-glucose labeling followed by GC/MS acquisition and analysis. Significance is shown for the differences between the areas under the curve (Time*genotype). The experiment was repeated twice.

(E) Mass isotopologues distribution of each analyzed time point. The detected isotopologues are M+0 and M+1 whereas M+3 served as an internal standard and added equally to each sample (Methods).

Data are presented as mean ± SEM of at two independent experiments. *$p<0.05$, Two-tailed unpaired Student’s t-test.

(F) Representative Western blot analysis of p53 protein levels.

(G) Glucose uptake was evaluated by 2-NBDG incubation (50 µM, for 30 minutes) by Imaging Flow Cytometry (IFC).

(H) Relative mRNA expression of GLUT1, Hexokinase2 (HK2), Pyruvate dehydrogenase kinase 1 (PDK1) and Lactate dehydrogenase-A (LDH-A) in H1299 cell line expressing R175H mutation and p53-null H1299 control cells.

(I) Relative mRNA expression of GLUT1, HK2, PDK1 and Monocarboxylate transporter 1 (MCT1) in MEFs expressing R172H mutation compared with p53 WT MEFs.

Data are presented as mean ± SEM of at least three independent experiments. *p<0.05, **p<0.01, ***p<0.001. Two-tailed unpaired Student's t-test.

Figure S2. Related to Figure 2

(A) GC-MS measurements of cell extracts from p53 Mut pMSCs and p53 Mut MSC-TLs incubated for 7 hours with RPMI containing 10mM of uniformly labeled 13C-Glucose. Left panel: Citrate isotopologue distribution. Right panel: Citrate pool of masses normalized to ribitol as internal standard and protein.

(B) GC-MS measurements of cell extracts from p53 Mut pMSCs and p53 Mut MSC-TLs incubated for 7 hours with RPMI containing 4mM of uniformly labeled 13C-Glutamine. Left panel: Citrate isotopologue distribution. Right panel: Citrate pool of masses normalized to ribitol as internal standard and to protein.

(C) GC-MS measurements of tumor extracts derived from p53 Mut pMSCs and p53 Mut MSC TLs subcutaneously injected. Once established, tumors were isolated and incubated for 9 hours with DMEM containing 10mM of uniformly labeled 13C-Glucose. Left

panel: Percentage of 13C enrichment for lactate as % M+3. Right panel: Lactate pool of masses normalized to ribitol as internal standard and protein. A minimum of three mice per cohort was used.

(D) GC-MS measurements of tumor extracts derived from p53 Mut pMSCs and p53 Mut MSC-TLs subcutaneously injected. Once established, tumors were isolated and incubated for 9 hours with DMEM containing 4mM of uniformly labeled 13C-Glutamine. Left panel: Percentage of 13C enrichment for Citrate as % M+4. Right panel: Citrate pool of masses normalized to ribitol as internal standard and protein. A minimum of three mice per cohort was used.

(E) Relative mRNA expression of Hmgcr, Mvd, Mvk, Pmvk and Sqle in p53 Mut MSC-TLs versus p53 Mut pMSCs.

Data are presented as mean ± S.D in (A-D) and ± SEM (E) of three independent experiments. *$p<0.05$, **$p<0.01$. Paired Student's t-test.

Figure S4. Related to Figure 4

(A, B) Representative EM micrographs (scale bar in big and small panels, 1µM and 500nm, respectively) of p53 Mut MSC-TLs 1 compared to p53 Mut pMSCs 1. Arrows indicates representative mitochondria.

(C-F) Representative EM micrographs (scale bar in big and small panels, 1µM and 500nm, respectively) of p53 Mut MSC-TLs 2 compared to p53 Mut pMSCs 2. Arrows indicates representative mitochondria.

(G) Distribution of mitochondria areas in EM micrographs of p53 Mut MSC-TLs 2 compared to p53 Mut pMSCs 2.

(H) Distribution of mitochondria areas in EM micrographs of p53 Mut MSC-TLs 5 compared to p53 Mut pMSCs 5.

(I-L) Representative EM micrographs (scale bar in big and small panels, 1µM and 500nm, respectively) of p53 Mut MSC-TLs 5 compared to p53 Mut pMSCs 5.

Figure S5. Related to Figure 5

(A) Representative Western blot analysis of HIF1α protein levels under 5% O_2, 21% O_2 and 21% O_2 + $CoCl_2$ (150µM, 20 hr), respectively.

(B) Relative mRNA expression of Sox2 and CD44 after 48 hr of treatment with Oligomycin (1µM) + Rotenone (0.5 µM).

Data are presented as mean ± SEM of at least three independent experiments.

*p<0.05, **p<0.01. Two-tailed paired Student's ttest in (A).

Figure S6. Related to Figure 6

(A) Representative Western blot analysis of p53 protein levels in p53 Mut MSC-TLs versus p53 Mut K/O MSC-TLs.

(B) Localization levels of GLUT1 protein at the plasma membrane evaluated by Imaging Flow Cytometry (IFC).

(C) Relative mRNA expression of Sox2.

Data are presented as mean ± SEM of at least three independent experiments. *p<0.05, **p<0.01, ***p< 0.001. One-way, two-sided ANOVA.

(D) Fluorescence intensity relative to nuclei staining after 96 hr of treatment with Metformin (5mM).

(E, F) Apoptotic cells after 48 hr of treatment with Oligomycin (1μM) + Rotenone (1 μM) were assessed by Imaging Flow Cytometry (IFC). Each value was normalized on its correspondent non-treated. For details see material and methods.

Data are presented as mean ± SEM of at least three independent experiments. *p<0.05, **p<0.01, ***p< 0.001. Two-way ANOVA (D), Two-tailed paired Student's ttest (E), One-way, two-sided ANOVA (F).
