# Supplementary material for: Mutant p53-dependent mitochondrial metabolic alterations in a mesenchymal stem cell-based model of progressive malignancy
Source: Cell Death Differ. 2018 Nov 9;26(9):1566–81. doi: 10.1038/s41418-018-0227-z (PMC6748146; doi:10.1038/s41418-018-0227-z)
Supplement: Supplementary file 3 — Supplementary table 1 [file 41418_2018_227_MOESM3_ESM.pdf]

| NAME | GS<br> foll | GS DETAILS | SIZE |
|---|---|---|---|
| PID_DNA_PK_PATHWAY | PID_DNA_I | Details ... | 16 |
| HOWLIN_CITED1_TARGETS_2_DN | HOWLIN_C | Details ... | 16 |
| COLLIS_PRKDC_REGULATORS | COLLIS_PRI | Details ... | 15 |
| HEDENFALK_BREAST_CANCER_BRACX_DN | HEDENFALI | Details ... | 20 |
| FERRANDO_TAL1_NEIGHBORS | FERRANDO | Details ... | 18 |
| LIN_MELANOMA_COPY_NUMBER_UP | LIN_MELAN | Details ... | 65 |
| PARK_TRETINOIN_RESPONSE_AND_PML_RARA_FUSION | PARK_TRET | Details ... | 29 |
| PID_EPHA2_FWD_PATHWAY | PID_EPHA2 | Details ... | 18 |
| REACTOME_CITRIC_ACID_CYCLE_TCA_CYCLE | REACTOME | Details ... | 18 |
| LAMB_CCND1_TARGETS | LAMB_CCN | Details ... | 19 |
| WALLACE_PROSTATE_CANCER_UP | WALLACE_ | Details ... | 19 |
| CHIARADONNA_NEOPLASTIC_TRANSFORMATION_KRAS_CDC25_U | CHIARADO | Details ... | 49 |
| CHEN_PDGF_TARGETS | CHEN_PDG | Details ... | 18 |
| PEDERSEN_METASTASIS_BY_ERBB2_ISOFORM_1 | PEDERSEN_ | Details ... | 45 |
| KUNINGER_IGF1_VS_PDGFB_TARGETS_DN | KUNINGER_ | Details ... | 43 |
| KOHOUTEK_CCNT1_TARGETS | KOHOUTEK | Details ... | 45 |
| DELACROIX_RAR_TARGETS_UP | DELACROIX | Details ... | 45 |
| TONKS_TARGETS_OF_RUNX1_RUNX1T1_FUSION_SUSTAINED_IN_ | TONKS_TAI | Details ... | 21 |
| BARRIER_CANCER_RELAPSE_TUMOR_SAMPLE_UP | BARRIER_C | Details ... | 15 |
| FIRESTEIN_CTNNB1_PATHWAY | FIRESTEIN_ | Details ... | 30 |
| CHENG_RESPONSE_TO_NICKEL_ACETATE | CHENG_RESPONSE_TC | | 39 |
| LANDIS_ERBB2_BREAST_PRENEOPLASTIC_UP | LANDIS_ERBB2_BREAS | | 17 |
| CHEOK_RESPONSE_TO_MERCAPTOPURINE_DN | CHEOK_RESPONSE_TO | | 18 |
| WARTERS_IR_RESPONSE_5GY | WARTERS_IR_RESPON | | 37 |
| DOANE_BREAST_CANCER_CLASSES_DN | DOANE_BREAST_CANC | | 32 |
| MARCINIAK_ER_STRESS_RESPONSE_VIA_CHOP | MARCINIAK_ER_STRES | | 23 |
| GARGALOVIC_RESPONSE_TO_OXIDIZED_PHOSPHOLIPIDS_BLUE_U | GARGALOVIC_RESPON | | 111 |
| SAGIV_CD24_TARGETS_DN | SAGIV_CD24_TARGETS | | 42 |
| COLLIS_PRKDC_SUBSTRATES | COLLIS_PRKDC_SUBST | | 19 |
| CAIRO_HEPATOBLASTOMA_POOR_SURVIVAL | CAIRO_HEPATOBLAST( | | 16 |
| STONER_ESOPHAGEAL_CARCINOGENESIS_UP | STONER_ESOPHAGEAL | | 34 |
| BIOCARTA_MITOCHONDRIA_PATHWAY | BIOCARTA_MITOCHON | | 21 |
| MEISSNER_BRAIN_HCP_WITH_H3K4ME2 | MEISSNER_BRAIN_HCI | | 16 |
| SHIN_B_CELL_LYMPHOMA_CLUSTER_5 | SHIN_B_CELL_LYMPHC | | 17 |
| KEGG_DRUG_METABOLISM_OTHER_ENZYMES | KEGG_DRUG_METABC | | 29 |
| NAGASHIMA_EGF_SIGNALING_UP | NAGASHIMA_EGF_SIG | | 57 |
| WELCH_GATA1_TARGETS | WELCH_GATA1_TARGI | | 19 |
| CHOW_RASSF1_TARGETS_UP | CHOW_RASSF1_TARGI | | 24 |
| REACTOME_SYNTHESIS_OF_PA | REACTOME_SYNTHESIS | | 27 |
| REACTOME_TELOMERE_MAINTENANCE | REACTOME_TELOMER | | 62 |
| MUELLER_COMMON_TARGETS_OF_AML_FUSIONS_DN | MUELLER_COMMON_ | | 29 |
| NADERI_BREAST_CANCER_PROGNOSIS_UP | NADERI_BREAST_CAN( | | 47 |
| CHEN_HOXA5_TARGETS_9HR_UP | CHEN_HOXA5_TARGET | | 183 |
| WHITFIELD_CELL_CYCLE_G1_S | WHITFIELD_CELL_CYCL | | 122 |
| MOOTHA_TCA | MOOTHA_TCA | | 15 |
| FIGUEROA_AML_METHYLATION_CLUSTER_2_UP | FIGUEROA_AML_METH | | 43 |
| GARGALOVIC_RESPONSE_TO_OXIDIZED_PHOSPHOLIPIDS_RED_UI | GARGALOVIC_RESPON | | 17 |
| WANG_RESPONSE_TO_BEXAROTENE_UP | WANG_RESPONSE_TO | | 30 |
| REACTOME_MITOCHONDRIAL_PROTEIN_IMPORT | REACTOME_MITOCHO | | 46 |

| | | |
|---|---|---|
| ST_TUMOR_NECROSIS_FACTOR_PATHWAY | ST_TUMOR_NECROSIS | 29 |
| REACTOME_METABOLISM_OF_POLYAMINES | REACTOME_METABOL | 15 |
| FAELT_B_CLL_WITH_VH3_21_DN | FAELT_B_CLL_WITH_V | 45 |
| NIKOLSKY_BREAST_CANCER_22Q13_AMPLICON | NIKOLSKY_BREAST_CA | 15 |
| KEGG_ALPHA_LINOLENIC_ACID_METABOLISM | KEGG_ALPHA_LINOLEI | 18 |
| BIOCARTA_WNT_PATHWAY | BIOCARTA_WNT_PATH | 26 |
| CHIARADONNA_NEOPLASTIC_TRANSFORMATION_KRAS_UP | CHIARADONNA_NEOPI | 120 |
| KEGG_LINOLEIC_ACID_METABOLISM | KEGG_LINOLEIC_ACID_ | 19 |
| NIKOLSKY_BREAST_CANCER_8Q23_Q24_AMPLICON | NIKOLSKY_BREAST_CA | 132 |
| REACTOME_PYRIMIDINE_METABOLISM | REACTOME_PYRIMIDII | 24 |
| ZEMBUTSU_SENSITIVITY_TO_METHOTREXATE | ZEMBUTSU_SENSITIVIT | 18 |
| PID_REELIN_PATHWAY | PID_REELIN_PATHWAY | 29 |
| GRAHAM_CML_QUIESCENT_VS_CML_DIVIDING_UP | GRAHAM_CML_QUIES | 22 |
| YANG_BREAST_CANCER_ESR1_LASER_DN | YANG_BREAST_CANCE | 47 |
| SHAFFER_IRF4_TARGETS_IN_ACTIVATED_B_LYMPHOCYTE | SHAFFER_IRF4_TARGE | 74 |
| LEE_METASTASIS_AND_ALTERNATIVE_SPLICING_DN | LEE_METASTASIS_ANE | 44 |
| TURASHVILI_BREAST_NORMAL_DUCTAL_VS_LOBULAR_UP | TURASHVILI_BREAST_I | 57 |
| KEGG_ALANINE_ASPARTATE_AND_GLUTAMATE_METABOLISM | KEGG_ALANINE_ASPAI | 31 |
| FISCHER_G1_S_CELL_CYCLE | FISCHER_G1_S_CELL_( | 185 |
| BASSO_CD40_SIGNALING_DN | BASSO_CD40_SIGNALI | 61 |
| REACTOME_ACYL_CHAIN_REMODELLING_OF_PG | REACTOME_ACYL_CHA | 16 |
| ACEVEDO_FGFR1_TARGETS_IN_PROSTATE_CANCER_MODEL_UP | ACEVEDO_FGFR1_TAR | 265 |
| DUTTA_APOPTOSIS_VIA_NFKB | DUTTA_APOPTOSIS_VI | 28 |
| REACTOME_EXTENSION_OF_TELOMERES | REACTOME_EXTENSIO | 27 |
| CHANDRAN_METASTASIS_TOP50_UP | CHANDRAN_METASTA | 33 |
| KEGG_MISMATCH_REPAIR | KEGG_MISMATCH_REI | 22 |
| SWEET_KRAS_TARGETS_DN | SWEET_KRAS_TARGET | 57 |
| PID_P73PATHWAY | PID_P73PATHWAY | 71 |
| LIANG_HEMATOPOIESIS_STEM_CELL_NUMBER_LARGE_VS_TINY_ | LIANG_HEMATOPOIES | 43 |
| PID_ARF6_PATHWAY | PID_ARF6_PATHWAY | 33 |
| REACTOME_DNA_STRAND_ELONGATION | REACTOME_DNA_STR/ | 30 |
| REACTOME_ACYL_CHAIN_REMODELLING_OF_PE | REACTOME_ACYL_CHA | 21 |
| BIOCARTA_G2_PATHWAY | BIOCARTA_G2_PATHW | 23 |
| OUYANG_PROSTATE_CANCER_MARKERS | OUYANG_PROSTATE_( | 19 |
| MARIADASON_RESPONSE_TO_CURCUMIN_SULINDAC_7 | MARIADASON_RESPOI | 15 |
| NIKOLSKY_BREAST_CANCER_1Q21_AMPLICON | NIKOLSKY_BREAST_CA | 36 |
| MARSHALL_VIRAL_INFECTION_RESPONSE_DN | MARSHALL_VIRAL_INF | 29 |
| OKAWA_NEUROBLASTOMA_1P36_31_DELETION | OKAWA_NEUROBLAST | 22 |
| KEGG_DNA_REPLICATION | KEGG_DNA_REPLICATI | 35 |
| KEGG_HOMOLOGOUS_RECOMBINATION | KEGG_HOMOLOGOUS_ | 27 |
| REACTOME_ACYL_CHAIN_REMODELLING_OF_PS | REACTOME_ACYL_CHA | 15 |
| WHITE_NEUROBLASTOMA_WITH_1P36.3_DELETION | WHITE_NEUROBLASTC | 20 |
| RICKMAN_TUMOR_DIFFERENTIATED_WELL_VS_MODERATELY_DI | RICKMAN_TUMOR_DII | 102 |
| REACTOME_HIV_LIFE_CYCLE | REACTOME_HIV_LIFE_ | 105 |
| SESTO_RESPONSE_TO_UV_C1 | SESTO_RESPONSE_TO_ | 64 |
| HAHTOLA_CTCL_CUTANEOUS | HAHTOLA_CTCL_CUTA | 22 |
| MARZEC_IL2_SIGNALING_UP | MARZEC_IL2_SIGNALII | 110 |
| PID_HIV_NEF_PATHWAY | PID_HIV_NEF_PATHW/ | 34 |
| AMIT_EGF_RESPONSE_120_MCF10A | AMIT_EGF_RESPONSE_ | 41 |
| KOBAYASHI_EGFR_SIGNALING_24HR_DN | KOBAYASHI_EGFR_SIG | 239 |

| | | |
|---|---|---|
| WONG_IFNA2_RESISTANCE_UP | WONG_IFNA2_RESIST/ | 16 |
| REACTOME_METABOLISM_OF_VITAMINS_AND_COFACTORS | REACTOME_METABOL | 49 |
| REACTOME_ACTIVATION_OF_THE_PRE_REPLICATIVE_COMPLEX | REACTOME_ACTIVATIC | 29 |
| REACTOME_SYNTHESIS_OF_PC | REACTOME_SYNTHESIS | 18 |
| BIOCARTA_ACH_PATHWAY | BIOCARTA_ACH_PATH | 15 |
| KAUFFMANN_DNA_REPAIR_GENES | KAUFFMANN_DNA_RE | 216 |
| WHITFIELD_CELL_CYCLE_S | WHITFIELD_CELL_CYCL | 137 |
| BEIER_GLIOMA_STEM_CELL_UP | BEIER_GLIOMA_STEM_ | 33 |
| DAZARD_UV_RESPONSE_CLUSTER_G24 | DAZARD_UV_RESPONS | 22 |
| SONG_TARGETS_OF_IE86_CMV_PROTEIN | SONG_TARGETS_OF_IE | 58 |
| HOFFMANN_PRE_BI_TO_LARGE_PRE_BII_LYMPHOCYTE_UP | HOFFMANN_PRE_BI_T | 34 |
| REACTOME_CTNNB1_PHOSPHORYLATION_CASCADE | REACTOME_CTNNB1_I | 15 |
| SANSOM_APC_TARGETS_UP | SANSOM_APC_TARGET | 117 |
| CHEOK_RESPONSE_TO_MERCAPTOPURINE_AND_HD_MTX_DN | CHEOK_RESPONSE_TO | 21 |
| REACTOME_INTERACTIONS_OF_VPR_WITH_HOST_CELLULAR_PR( | REACTOME_INTERACT | 31 |
| GARGALOVIC_RESPONSE_TO_OXIDIZED_PHOSPHOLIPIDS_GREEN_ | GARGALOVIC_RESPON | 22 |
| DAZARD_RESPONSE_TO_UV_SCC_DN | DAZARD_RESPONSE_T | 113 |
| KEGG_ETHER_LIPID_METABOLISM | KEGG_ETHER_LIPID_M | 32 |
| GARGALOVIC_RESPONSE_TO_OXIDIZED_PHOSPHOLIPIDS_BLACK_ | GARGALOVIC_RESPON | 31 |
| DUTERTRE_ESTRADIOL_RESPONSE_6HR_UP | DUTERTRE_ESTRADIOL | 216 |
| REN_BOUND_BY_E2F | REN_BOUND_BY_E2F | 58 |
| REACTOME_TRANSCRIPTIONAL_ACTIVITY_OF_SMAD2_SMAD3_SI | REACTOME_TRANSCRI | 36 |
| PID_ERBB_NETWORK_PATHWAY | PID_ERBB_NETWORK_ | 15 |
| REACTOME_CYTOSOLIC_TRNA_AMINOACYLATION | REACTOME_CYTOSOLI | 24 |
| KOKKINAKIS_METHIONINE_DEPRIVATION_96HR_DN | KOKKINAKIS_METHION | 75 |
| GENTILE_UV_RESPONSE_CLUSTER_D1 | GENTILE_UV_RESPONS | 16 |
| REACTOME_DESTABILIZATION_OF_MRNA_BY_KSRP | REACTOME_DESTABILI | 17 |
| BILANGES_RAPAMYCIN_SENSITIVE_GENES | BILANGES_RAPAMYCIN | 37 |
| KORKOLA_CORRELATED_WITH_POU5F1 | KORKOLA_CORRELATE | 27 |
| BRACHAT_RESPONSE_TO_METHOTREXATE_UP | BRACHAT_RESPONSE_ | 21 |
| BROWNE_HCMV_INFECTION_16HR_UP | BROWNE_HCMV_INFE | 207 |
| DE_YY1_TARGETS_UP | DE_YY1_TARGETS_UP | 19 |
| WANG_RECURRENT_LIVER_CANCER_UP | WANG_RECURRENT_L | 19 |
| KAUFFMANN_MELANOMA_RELAPSE_UP | KAUFFMANN_MELANC | 58 |
| DING_LUNG_CANCER_EXPRESSION_BY_COPY_NUMBER | DING_LUNG_CANCER_ | 95 |
| SCHRAMM_INHBA_TARGETS_DN | SCHRAMM_INHBA_TA | 24 |
| PUJANA_BRCA_CENTERED_NETWORK | PUJANA_BRCA_CENTE | 114 |
| BIOCARTA_STRESS_PATHWAY | BIOCARTA_STRESS_PA | 25 |
| DORN_ADENOVIRUS_INFECTION_32HR_DN | DORN_ADENOVIRUS_I | 38 |
| BRUINS_UVC_RESPONSE_MIDDLE | BRUINS_UVC_RESPON | 84 |
| WANG_RESPONSE_TO_GSK3_INHIBITOR_SB216763_DN | WANG_RESPONSE_TO | 334 |
| LANDIS_BREAST_CANCER_PROGRESSION_UP | LANDIS_BREAST_CANC | 42 |
| REACTOME_LAGGING_STRAND_SYNTHESIS | REACTOME_LAGGING_ | 19 |
| KEGG_CITRATE_CYCLE_TCA_CYCLE | KEGG_CITRATE_CYCLE | 28 |
| BURTON_ADIPOGENESIS_PEAK_AT_8HR | BURTON_ADIPOGENES | 38 |
| REACTOME_MEIOTIC_RECOMBINATION | REACTOME_MEIOTIC_ | 66 |
| ROYLANCE_BREAST_CANCER_16Q_COPY_NUMBER_DN | ROYLANCE_BREAST_C/ | 22 |
| PID_NFKAPPAB_ATYPICAL_PATHWAY | PID_NFKAPPAB_ATYPI( | 17 |
| PUIFFE_INVASION_INHIBITED_BY_ASCITES_DN | PUIFFE_INVASION_INH | 133 |
| BOSCO_EPITHELIAL_DIFFERENTIATION_MODULE | BOSCO_EPITHELIAL_DI | 53 |

| | | |
|---|---|---|
| TUOMISTO_TUMOR_SUPPRESSION_BY_COL13A1_DN | TUOMISTO_TUMOR_S | 17 |
| ROESSLER_LIVER_CANCER_METASTASIS_DN | ROESSLER_LIVER_CAN | 44 |
| REACTOME_PROCESSIVE_SYNTHESIS_ON_THE_LAGGING_STRAND | REACTOME_PROCESSI | 15 |
| REACTOME_ACTIVATION_OF_ATR_IN_RESPONSE_TO_REPLICATIC | REACTOME_ACTIVATIC | 35 |
| BIOCARTA_ATM_PATHWAY | BIOCARTA_ATM_PATH | 18 |
| BIOCARTA_CHEMICAL_PATHWAY | BIOCARTA_CHEMICAL_ | 21 |
| KOBAYASHI_EGFR_SIGNALING_6HR_DN | KOBAYASHI_EGFR_SIG | 18 |
| REACTOME_G1_S_SPECIFIC_TRANSCRIPTION | REACTOME_G1_S_SPE | 17 |
| REACTOME_MAP_KINASE_ACTIVATION_IN_TLR_CASCADE | REACTOME_MAP_KIN | 49 |
| HOFMANN_MYELODYSPLASTIC_SYNDROM_RISK_UP | HOFMANN_MYELODY | 23 |
| RHEIN_ALL_GLUCOCORTICOID_THERAPY_UP | RHEIN_ALL_GLUCOCO | 69 |
| REACTOME_PACKAGING_OF_TELOMERE_ENDS | REACTOME_PACKAGIN | 35 |
| BIOCARTA_ETS_PATHWAY | BIOCARTA_ETS_PATH | 17 |
| ROESSLER_LIVER_CANCER_METASTASIS_UP | ROESSLER_LIVER_CAN | 99 |
| ZHENG_FOXP3_TARGETS_UP | ZHENG_FOXP3_TARGE | 25 |
| DURCHDEWALD_SKIN_CARCINOGENESIS_DN | DURCHDEWALD_SKIN_ | 246 |
| SU_THYMUS | SU_THYMUS | 17 |
| REACTOME_G2_M_CHECKPOINTS | REACTOME_G2_M_CH | 41 |
| MORI_MATURE_B_LYMPHOCYTE_DN | MORI_MATURE_B_LYI | 72 |
| PARENT_MTOR_SIGNALING_DN | PARENT_MTOR_SIGN | 41 |
| MAHADEVAN_RESPONSE_TO_MP470_DN | MAHADEVAN_RESPON | 18 |
| GALINDO_IMMUNE_RESPONSE_TO_ENTEROTOXIN | GALINDO_IMMUNE_R | 79 |
| REACTOME_DOUBLE_STRAND_BREAK_REPAIR | REACTOME_DOUBLE_ | 21 |
| REACTOME_AMINE_DERIVED_HORMONES | REACTOME_AMINE_D | 15 |
| DORN_ADENOVIRUS_INFECTION_24HR_DN | DORN_ADENOVIRUS_I | 42 |
| DAIRKEE_CANCER_PRONE_RESPONSE_BPA | DAIRKEE_CANCER_PR | 49 |
| REACTOME_JNK_C_JUN_KINASES_PHOSPHORYLATION_AND_ACT | REACTOME_JNK_C_JU | 16 |
| LE_NEURONAL_DIFFERENTIATION_DN | LE_NEURONAL_DIFFER | 19 |
| PUJANA_XPRSS_INT_NETWORK | PUJANA_XPRSS_INT_N | 157 |
| REACTOME_PLATELET_SENSITIZATION_BY_LDL | REACTOME_PLATELET_ | 16 |
| MMS_MOUSE_LYMPH_HIGH_4HRS_UP | MMS_MOUSE_LYMPH | 34 |
| REACTOME_FACTORS_INVOLVED_IN_MEGAKARYOCYTE_DEVELOI | REACTOME_FACTORS_ | 110 |
| YAGI_AML_SURVIVAL | YAGI_AML_SURVIVAL | 121 |
| ZHANG_RESPONSE_TO_CANTHARIDIN_DN | ZHANG_RESPONSE_TC | 65 |
| BURTON_ADIPOGENESIS_1 | BURTON_ADIPOGENES | 33 |
| WAKASUGI_HAVE_ZNF143_BINDING_SITES | WAKASUGI_HAVE_ZNI | 57 |
| VECCHI_GASTRIC_CANCER_EARLY_UP | VECCHI_GASTRIC_CAN | 387 |
| YANG_BREAST_CANCER_ESR1_BULK_DN | YANG_BREAST_CANCE | 22 |
| REACTOME_TRNA_AMINOACYLATION | REACTOME_TRNA_AM | 42 |
| PID_PI3KCI_AKT_PATHWAY | PID_PI3KCI_AKT_PATH | 35 |
| REACTOME_E2F_MEDIATED_REGULATION_OF_DNA_REPLICATIOI | REACTOME_E2F_MED | 32 |
| DIRMEIER_LMP1_RESPONSE_EARLY | DIRMEIER_LMP1_RESP | 64 |
| KEGG_ONE_CARBON_POOL_BY_FOLATE | KEGG_ONE_CARBON_ | 17 |
| AGUIRRE_PANCREATIC_CANCER_COPY_NUMBER_DN | AGUIRRE_PANCREATIC | 220 |
| REACTOME_SMAD2_SMAD3_SMAD4_HETEROTRIMER_REGULATE | REACTOME_SMAD2_S | 25 |
| FINETTI_BREAST_CANCERS_KINOME_GRAY | FINETTI_BREAST_CANC | 15 |
| VIETOR_IFRD1_TARGETS | VIETOR_IFRD1_TARGE | 23 |
| SANSOM_APC_TARGETS | SANSOM_APC_TARGE | 190 |
| BIOCARTA_TNFR2_PATHWAY | BIOCARTA_TNFR2_PAT | 18 |
| SU_PANCREAS | SU_PANCREAS | 44 |

| | | |
|---|---|---|
| OUILLETTE_CLL_13Q14_DELETION_UP | OUILLETTE_CLL_13Q1 | 60 |
| DELACROIX_RAR_BOUND_ES | DELACROIX_RAR_BOU | 419 |
| MATTHEWS_AP1_TARGETS | MATTHEWS_AP1_TAR | 16 |
| REACTOME_BASIGIN_INTERACTIONS | REACTOME_BASIGIN_I | 23 |
| REACTOME_DEPOSITION_OF_NEW_CENPA_CONTAINING_NUCLE | REACTOME_DEPOSITIO | 51 |
| BENPORATH_PROLIFERATION | BENPORATH_PROLIFEI | 136 |
| PID_FOXO_PATHWAY | PID_FOXO_PATHWAY | 48 |
| DORN_ADENOVIRUS_INFECTION_48HR_DN | DORN_ADENOVIRUS_I | 39 |
| BURTON_ADIPOGENESIS_PEAK_AT_16HR | BURTON_ADIPOGENES | 38 |
| STEIN_ESRRA_TARGETS_RESPONSIVE_TO_ESTROGEN_UP | STEIN_ESRRA_TARGET | 24 |
| BOQUEST_STEM_CELL_DN | BOQUEST_STEM_CELL | 195 |
| MAGRANGEAS_MULTIPLE_MYELOMA_IGG_VS_IGA_DN | MAGRANGEAS_MULTI | 22 |
| BROWNE_HCMV_INFECTION_18HR_UP | BROWNE_HCMV_INFE | 167 |
| REACTOME_RESOLUTION_OF_AP_SITES_VIA_THE_MULTIPLE_NU | REACTOME_RESOLUTI | 17 |
| REACTOME_ENOS_ACTIVATION_AND_REGULATION | REACTOME_ENOS_AC | 19 |
| AKL_HTLV1_INFECTION_UP | AKL_HTLV1_INFECTIOI | 24 |
| PID_CMYB_PATHWAY | PID_CMYB_PATHWAY | 79 |
| WILLIAMS_ESR1_TARGETS_UP | WILLIAMS_ESR1_TARG | 25 |
| MUELLER_METHYLATED_IN_GLIOBLASTOMA | MUELLER_METHYLATE | 35 |
| AMIT_EGF_RESPONSE_120_HELA | AMIT_EGF_RESPONSE_ | 65 |
| REACTOME_METABOLISM_OF_NUCLEOTIDES | REACTOME_METABOL | 69 |
| PYEON_HPV_POSITIVE_TUMORS_UP | PYEON_HPV_POSITIVE | 75 |
| BOYLAN_MULTIPLE_MYELOMA_C_CLUSTER_UP | BOYLAN_MULTIPLE_M | 37 |
| BROCKE_APOPTOSIS_REVERSED_BY_IL6 | BROCKE_APOPTOSIS_I | 135 |
| DEN_INTERACT_WITH_LCA5 | DEN_INTERACT_WITH_ | 26 |
| MUELLER_PLURINET | MUELLER_PLURINET | 284 |
| REACTOME_ACYL_CHAIN_REMODELLING_OF_PC | REACTOME_ACYL_CHA | 22 |
| LI_WILMS_TUMOR_VS_FETAL_KIDNEY_1_DN | LI_WILMS_TUMOR_VS | 156 |
| BROWNE_HCMV_INFECTION_48HR_UP | BROWNE_HCMV_INFE | 160 |
| NEMETH_INFLAMMATORY_RESPONSE_LPS_UP | NEMETH_INFLAMMAT | 81 |
| BARIS_THYROID_CANCER_UP | BARIS_THYROID_CANC | 23 |
| MOOTHA_PGC | MOOTHA_PGC | 390 |
| ZHANG_GATA6_TARGETS_DN | ZHANG_GATA6_TARGI | 62 |
| GENTILE_UV_HIGH_DOSE_UP | GENTILE_UV_HIGH_DC | 23 |
| BIOCARTA_DC_PATHWAY | BIOCARTA_DC_PATHW | 22 |
| REACTOME_TRANSPORT_OF_MATURE_MRNA_DERIVED_FROM_/ | REACTOME_TRANSPOI | 32 |
| SMIRNOV_RESPONSE_TO_IR_2HR_UP | SMIRNOV_RESPONSE_ | 47 |
| KEGG_GLYCEROPHOSPHOLIPID_METABOLISM | KEGG_GLYCEROPHOSF | 75 |
| BONOME_OVARIAN_CANCER_POOR_SURVIVAL_DN | BONOME_OVARIAN_C | 20 |
| MAHADEVAN_RESPONSE_TO_MP470_UP | MAHADEVAN_RESPON | 17 |
| IZADPANAH_STEM_CELL_ADIPOSE_VS_BONE_UP | IZADPANAH_STEM_CE | 107 |
| PENG_GLUCOSE_DEPRIVATION_UP | PENG_GLUCOSE_DEPR | 42 |
| REACTOME_DOWNREGULATION_OF_SMAD2_3_SMAD4_TRANSC | REACTOME_DOWNRE | 19 |
| BIOCARTA_CASPASE_PATHWAY | BIOCARTA_CASPASE_F | 22 |
| GAVIN_IL2_RESPONSIVE_FOXP3_TARGETS_UP | GAVIN_IL2_RESPONSI\ | 17 |
| WALLACE_PROSTATE_CANCER_RACE_DN | WALLACE_PROSTATE_ | 65 |
| CHANG_CYCLING_GENES | CHANG_CYCLING_GEN | 135 |
| TAYLOR_METHYLATED_IN_ACUTE_LYMPHOBLASTIC_LEUKEMIA | TAYLOR_METHYLATED | 71 |
| WARTERS_RESPONSE_TO_IR_SKIN | WARTERS_RESPONSE_ | 63 |
| TIAN_TNF_SIGNALING_NOT_VIA_NFKB | TIAN_TNF_SIGNALING | 21 |

| | | |
|---|---|---|
| SHETH_LIVER_CANCER_VS_TXNIP_LOSS_PAM6 | SHETH_LIVER_CANCER | 44 |
| CREIGHTON_ENDOCRINE_THERAPY_RESISTANCE_2 | CREIGHTON_ENDOCRI | 337 |
| BILBAN_B_CLL_LPL_DN | BILBAN_B_CLL_LPL_DI | 38 |
| YEGNASUBRAMANIAN_PROSTATE_CANCER | YEGNASUBRAMANIAN | 107 |
| SWEET_KRAS_ONCOGENIC_SIGNATURE | SWEET_KRAS_ONCOGI | 85 |
| BERENJENO_ROCK_SIGNALING_NOT_VIA_RHOA_UP | BERENJENO_ROCK_SI( | 27 |
| VALK_AML_CLUSTER_7 | VALK_AML_CLUSTER_ | 23 |
| REACTOME_LATE_PHASE_OF_HIV_LIFE_CYCLE | REACTOME_LATE_PH/ | 92 |
| ABRAMSON_INTERACT_WITH_AIRE | ABRAMSON_INTERACT | 42 |
| REACTOME_NUCLEAR_EVENTS_KINASE_AND_TRANSCRIPTION_F/ | REACTOME_NUCLEAR_ | 24 |
| ISHIDA_E2F_TARGETS | ISHIDA_E2F_TARGETS | 51 |
| BIOCARTA_ATRBRCA_PATHWAY | BIOCARTA_ATRBRCA_I | 20 |
| SARRIO_EPITHELIAL_MESENCHYMAL_TRANSITION_UP | SARRIO_EPITHELIAL_N | 164 |
| MARKS_HDAC_TARGETS_DN | MARKS_HDAC_TARGET | 15 |
| MATTHEWS_SKIN_CARCINOGENESIS_VIA_JUN | MATTHEWS_SKIN_CAF | 15 |
| REACTOME_SIGNALING_BY_TGF_BETA_RECEPTOR_COMPLEX | REACTOME_SIGNALIN( | 60 |
| KEGG_APOPTOSIS | KEGG_APOPTOSIS | 79 |
| BOYAULT_LIVER_CANCER_SUBCLASS_G23_UP | BOYAULT_LIVER_CAN( | 48 |
| CHICAS_RB1_TARGETS_SENESCENT | CHICAS_RB1_TARGETS | 500 |
| REACTOME_RNA_POL_I_PROMOTER_OPENING | REACTOME_RNA_POL_ | 44 |
| BROWN_MYELOID_CELL_DEVELOPMENT_DN | BROWN_MYELOID_CE | 117 |
| DORMOY_ELAVL1_TARGETS | DORMOY_ELAVL1_TAF | 16 |
| PUJANA_BRCA2_PCC_NETWORK | PUJANA_BRCA2_PCC_ | 389 |
| PENG_GLUCOSE_DEPRIVATION_DN | PENG_GLUCOSE_DEPR | 157 |
| RODRIGUES_NTN1_AND_DCC_TARGETS | RODRIGUES_NTN1_AN | 31 |
| WU_HBX_TARGETS_2_UP | WU_HBX_TARGETS_2_ | 22 |
| MURAKAMI_UV_RESPONSE_24HR | MURAKAMI_UV_RESP( | 18 |
| PYEON_CANCER_HEAD_AND_NECK_VS_CERVICAL_UP | PYEON_CANCER_HEAD | 166 |
| INGA_TP53_TARGETS | INGA_TP53_TARGETS | 15 |
| FARMER_BREAST_CANCER_CLUSTER_2 | FARMER_BREAST_CAN | 32 |
| REACTOME_ACTIVATION_OF_GENES_BY_ATF4 | REACTOME_ACTIVATI( | 24 |
| KIM_WT1_TARGETS_UP | KIM_WT1_TARGETS_L | 204 |
| ACOSTA_PROLIFERATION_INDEPENDENT_MYC_TARGETS_UP | ACOSTA_PROLIFERATI( | 75 |
| ST_ERK1_ERK2_MAPK_PATHWAY | ST_ERK1_ERK2_MAPK_ | 32 |
| REACTOME_NUCLEOTIDE_BINDING_DOMAIN_LEUCINE_RICH_REI | REACTOME_NUCLEOTI | 42 |
| PID_HDAC_CLASSI_PATHWAY | PID_HDAC_CLASSI_PA | 66 |
| LIN_TUMOR_ESCAPE_FROM_IMMUNE_ATTACK | LIN_TUMOR_ESCAPE_ | 15 |
| PID_ATF2_PATHWAY | PID_ATF2_PATHWAY | 57 |
| KEGG_AMINOACYL_TRNA_BIOSYNTHESIS | KEGG_AMINOACYL_TF | 41 |
| REACTOME_BASE_EXCISION_REPAIR | REACTOME_BASE_EXC | 19 |
| PID_AURORA_A_PATHWAY | PID_AURORA_A_PATH | 29 |
| VERNELL_RETINOBLASTOMA_PATHWAY_UP | VERNELL_RETINOBLAS | 69 |
| PID_NFKAPPAB_CANONICAL_PATHWAY | PID_NFKAPPAB_CANO | 23 |
| EGUCHI_CELL_CYCLE_RB1_TARGETS | EGUCHI_CELL_CYCLE_I | 23 |
| REACTOME_TRANSCRIPTION_COUPLED_NER_TC_NER | REACTOME_TRANSCRI | 43 |
| BIOCARTA_MCM_PATHWAY | BIOCARTA_MCM_PATI | 17 |
| HUANG_GATA2_TARGETS_DN | HUANG_GATA2_TARG | 71 |
| PLASARI_TGFB1_TARGETS_10HR_UP | PLASARI_TGFB1_TARG | 191 |
| KEGG_BASE_EXCISION_REPAIR | KEGG_BASE_EXCISION | 33 |
| MARCHINI_TRABECTEDIN_RESISTANCE_UP | MARCHINI_TRABECTEI | 17 |

| | | |
|---|---|---|
| REACTOME_MAPK_TARGETS_NUCLEAR_EVENTS_MEDIATED_BY_ | REACTOME_MAPK_TA | 30 |
| SHIPP_DLBCL_CURED_VS_FATAL_DN | SHIPP_DLBCL_CURED_ | 40 |
| PID_TNF_PATHWAY | PID_TNF_PATHWAY | 45 |
| SMID_BREAST_CANCER_LUMINAL_A_DN | SMID_BREAST_CANCE | 18 |
| KAUFFMANN_DNA_REPLICATION_GENES | KAUFFMANN_DNA_RE | 137 |
| NIKOLSKY_BREAST_CANCER_6P24_P22_AMPLICON | NIKOLSKY_BREAST_CA | 20 |
| WAMUNYOKOLI_OVARIAN_CANCER_LMP_UP | WAMUNYOKOLI_OVA | 236 |
| OUELLET_CULTURED_OVARIAN_CANCER_INVASIVE_VS_LMP_UP | OUELLET_CULTURED_ | 66 |
| OISHI_CHOLANGIOMA_STEM_CELL_LIKE_UP | OISHI_CHOLANGIOMA | 289 |
| BENPORATH_ES_1 | BENPORATH_ES_1 | 353 |
| OXFORD_RALA_OR_RALB_TARGETS_UP | OXFORD_RALA_OR_R | 48 |
| YAMAZAKI_TCEB3_TARGETS_DN | YAMAZAKI_TCEB3_TA | 199 |
| DELACROIX_RARG_BOUND_MEF | DELACROIX_RARG_BO | 345 |
| DELLA_RESPONSE_TO_TSA_AND_BUTYRATE | DELLA_RESPONSE_TO_ | 21 |
| COLLER_MYC_TARGETS_UP | COLLER_MYC_TARGET | 24 |
| PUJANA_BREAST_CANCER_WITH_BRCA1_MUTATED_UP | PUJANA_BREAST_CAN | 54 |
| REACTOME_SYNTHESIS_OF_GLYCOSYLPHOSPHATIDYLINOSITOL_ | REACTOME_SYNTHESI | 17 |
| ZHANG_TLX_TARGETS_DN | ZHANG_TLX_TARGETS_ | 86 |
| KEGG_PYRIMIDINE_METABOLISM | KEGG_PYRIMIDINE_M | 95 |
| NIKOLSKY_BREAST_CANCER_8Q12_Q22_AMPLICON | NIKOLSKY_BREAST_CA | 116 |
| REACTOME_RNA_POL_I_TRANSCRIPTION | REACTOME_RNA_POL_ | 68 |
| PID_BARD1_PATHWAY | PID_BARD1_PATHWA | 28 |
| DORSAM_HOXA9_TARGETS_UP | DORSAM_HOXA9_TAR | 35 |
| ZHANG_TLX_TARGETS_60HR_DN | ZHANG_TLX_TARGETS_ | 264 |
| PID_TELOMERASE_PATHWAY | PID_TELOMERASE_PA | 67 |
| REACTOME_GOLGI_ASSOCIATED_VESICLE_BIOGENESIS | REACTOME_GOLGI_AS | 49 |
| REACTOME_TRANSFERRIN_ENDOCYTOSIS_AND_RECYCLING | REACTOME_TRANSFE | 23 |
| REACTOME_CHROMOSOME_MAINTENANCE | REACTOME_CHROMO | 105 |
| REACTOME_G0_AND_EARLY_G1 | REACTOME_G0_AND_ | 23 |
| MONNIER_POSTRADIATION_TUMOR_ESCAPE_UP | MONNIER_POSTRADIA | 367 |
| FOURNIER_ACINAR_DEVELOPMENT_LATE_DN | FOURNIER_ACINAR_D | 21 |
| BIDUS_METASTASIS_UP | BIDUS_METASTASIS_U | 202 |
| PID_TCR_CALCIUM_PATHWAY | PID_TCR_CALCIUM_P | 27 |
| FURUKAWA_DUSP6_TARGETS_PCI35_DN | FURUKAWA_DUSP6_T | 66 |
| GAVIN_FOXP3_TARGETS_CLUSTER_T4 | GAVIN_FOXP3_TARGE | 89 |
| PID_HDAC_CLASSIII_PATHWAY | PID_HDAC_CLASSIII_P | 24 |
| KEGG_EPITHELIAL_CELL_SIGNALING_IN_HELICOBACTER_PYLORI_ | KEGG_EPITHELIAL_CEL | 67 |
| EPPERT_PROGENITOR | EPPERT_PROGENITOR | 127 |
| NOJIMA_SFRP2_TARGETS_DN | NOJIMA_SFRP2_TARG | 24 |
| LUCAS_HNF4A_TARGETS_UP | LUCAS_HNF4A_TARGE | 53 |
| KUROZUMI_RESPONSE_TO_ONCOCYTIC_VIRUS_AND_CYCLIC_RG | KUROZUMI_RESPONSE | 20 |
| PID_ATR_PATHWAY | PID_ATR_PATHWAY | 39 |
| PID_EPHA_FWDPATHWAY | PID_EPHA_FWDPATH | 34 |
| BHATTACHARYA_EMBRYONIC_STEM_CELL | BHATTACHARYA_EMB | 83 |
| REACTOME_REGULATION_OF_GLUCOKINASE_BY_GLUCOKINASE_ | REACTOME_REGULATI | 27 |
| KRIEG_HYPOXIA_VIA_KDM3A | KRIEG_HYPOXIA_VIA_ | 48 |
| ZHAN_MULTIPLE_MYELOMA_MF_DN | ZHAN_MULTIPLE_MYE | 36 |
| CAVARD_LIVER_CANCER_MALIGNANT_VS_BENIGN | CAVARD_LIVER_CANC | 25 |
| WANG_METHYLATED_IN_BREAST_CANCER | WANG_METHYLATED_ | 32 |
| REACTOME_TRANSPORT_OF_RIBONUCLEOPROTEINS_INTO_THE_ | REACTOME_TRANSPO | 27 |

| | | |
|---|---|---|
| KOKKINAKIS_METHIONINE_DEPRIVATION_48HR_DN | KOKKINAKIS_METHION | 64 |
| REACTOME_ERK_MAPK_TARGETS | REACTOME_ERK_MAP | 21 |
| SUZUKI_RESPONSE_TO_TSA_AND_DECITABINE_1B | SUZUKI_RESPONSE_TC | 18 |
| VANOEVELEN_MYOGENESIS_SIN3A_TARGETS | VANOEVELEN_MYOGE | 202 |
| THEILGAARD_NEUTROPHIL_AT_SKIN_WOUND_UP | THEILGAARD_NEUTRO | 71 |
| SUNG_METASTASIS_STROMA_DN | SUNG_METASTASIS_ST | 46 |
| FINETTI_BREAST_CANCER_KINOME_RED | FINETTI_BREAST_CANC | 16 |
| DAIRKEE_CANCER_PRONE_RESPONSE_BPA_E2 | DAIRKEE_CANCER_PRC | 111 |
| REACTOME_TRAF6_MEDIATED_INDUCTION_OF_NFKB_AND_MAF | REACTOME_TRAF6_M | 73 |
| WANG_CLIM2_TARGETS_UP | WANG_CLIM2_TARGET | 206 |
| CHIANG_LIVER_CANCER_SUBCLASS_PROLIFERATION_UP | CHIANG_LIVER_CANCE | 164 |
| SANSOM_APC_MYC_TARGETS | SANSOM_APC_MYC_T | 196 |
| JOSEPH_RESPONSE_TO_SODIUM_BUTYRATE_UP | JOSEPH_RESPONSE_TC | 29 |
| KEGG_NUCLEOTIDE_EXCISION_REPAIR | KEGG_NUCLEOTIDE_E) | 43 |
| REACTOME_INSULIN_RECEPTOR_RECYCLING | REACTOME_INSULIN_I | 21 |
| ONDER_CDH1_TARGETS_3_DN | ONDER_CDH1_TARGET | 49 |
| KEGG_BASAL_TRANSCRIPTION_FACTORS | KEGG_BASAL_TRANSC | 33 |
| REACTOME_G1_S_TRANSITION | REACTOME_G1_S_TRA | 103 |
| DELPUECH_FOXO3_TARGETS_DN | DELPUECH_FOXO3_TA | 39 |
| REACTOME_TIGHT_JUNCTION_INTERACTIONS | REACTOME_TIGHT_JU | 29 |
| KONG_E2F3_TARGETS | KONG_E2F3_TARGETS | 94 |
| CROMER_METASTASIS_DN | CROMER_METASTASIS | 78 |
| GALLUZZI_PREVENT_MITOCHONDIAL_PERMEABILIZATION | GALLUZZI_PREVENT_M | 21 |
| GRAHAM_NORMAL_QUIESCENT_VS_NORMAL_DIVIDING_DN | GRAHAM_NORMAL_Q | 83 |
| MARKEY_RB1_CHRONIC_LOF_UP | MARKEY_RB1_CHRON | 111 |
| ZHOU_CELL_CYCLE_GENES_IN_IR_RESPONSE_6HR | ZHOU_CELL_CYCLE_GE | 81 |
| REACTOME_RNA_POL_II_TRANSCRIPTION_PRE_INITIATION_AND_ | REACTOME_RNA_POL_ | 38 |
| PID_P38_ALPHA_BETA_DOWNSTREAM_PATHWAY | PID_P38_ALPHA_BETA | 37 |
| LI_CYTIDINE_ANALOG_PATHWAY | LI_CYTIDINE_ANALOG_ | 17 |
| KEGG_PROXIMAL_TUBULE_BICARBONATE_RECLAMATION | KEGG_PROXIMAL_TUE | 20 |
| SHAFFER_IRF4_MULTIPLE_MYELOMA_PROGRAM | SHAFFER_IRF4_MULTII | 34 |
| VANTVEER_BREAST_CANCER_METASTASIS_DN | VANTVEER_BREAST_C/ | 107 |
| SUH_COEXPRESSED_WITH_ID1_AND_ID2_UP | SUH_COEXPRESSED_W | 18 |
| FRASOR_RESPONSE_TO_SERM_OR_FULVESTRANT_DN | FRASOR_RESPONSE_T( | 50 |
| REACTOME_MITOTIC_G1_G1_S_PHASES | REACTOME_MITOTIC_ | 127 |
| JACKSON_DNMT1_TARGETS_UP | JACKSON_DNMT1_TAF | 76 |
| REACTOME_PURINE_METABOLISM | REACTOME_PURINE_M | 32 |
| NUNODA_RESPONSE_TO_DASATINIB_IMATINIB_UP | NUNODA_RESPONSE_ | 29 |
| REACTOME_NUCLEOTIDE_EXCISION_REPAIR | REACTOME_NUCLEOTI | 48 |
| REACTOME_METABOLISM_OF_AMINO_ACIDS_AND_DERIVATIVES | REACTOME_METABOL | 191 |
| MANTOVANI_VIRAL_GPCR_SIGNALING_DN | MANTOVANI_VIRAL_G | 42 |
| ZHAN_MULTIPLE_MYELOMA_HP_DN | ZHAN_MULTIPLE_MYE | 42 |
| BOYAULT_LIVER_CANCER_SUBCLASS_G3_UP | BOYAULT_LIVER_CANC | 181 |
| MOOTHA_HUMAN_MITODB_6_2002 | MOOTHA_HUMAN_M | 410 |
| REACTOME_SIGNALING_BY_HIPPO | REACTOME_SIGNALIN( | 20 |
| MANN_RESPONSE_TO_AMIFOSTINE_UP | MANN_RESPONSE_TO_ | 20 |
| HE_PTEN_TARGETS_UP | HE_PTEN_TARGETS_U | 15 |
| NAGASHIMA_NRG1_SIGNALING_UP | NAGASHIMA_NRG1_SI | 168 |
| REACTOME_KINESINS | REACTOME_KINESINS | 22 |
| IWANAGA_CARCINOGENESIS_BY_KRAS_DN | IWANAGA_CARCINOGI | 114 |

| | | |
|---|---|---|
| EHLERS_ANEUPLOIDY_UP | EHLERS_ANEUPLOIDY_ | 41 |
| LEE_DOUBLE_POLAR_THYMOCYTE | LEE_DOUBLE_POLAR_ | 21 |
| CHIANG_LIVER_CANCER_SUBCLASS_UNANNOTATED_DN | CHIANG_LIVER_CANCE | 177 |
| REACTOME_DNA_REPAIR | REACTOME_DNA_REP/ | 101 |
| DUTERTRE_ESTRADIOL_RESPONSE_24HR_UP | DUTERTRE_ESTRADIOL | 306 |
| MCBRYAN_PUBERTAL_TGFB1_TARGETS_DN | MCBRYAN_PUBERTAL_ | 60 |
| ZHANG_BREAST_CANCER_PROGENITORS_UP | ZHANG_BREAST_CANC | 381 |
| REACTOME_RNA_POL_I_TRANSCRIPTION_INITIATION | REACTOME_RNA_POL_ | 23 |
| GARGALOVIC_RESPONSE_TO_OXIDIZED_PHOSPHOLIPIDS_MAGEN | GARGALOVIC_RESPON | 22 |
| MORI_PRE_BI_LYMPHOCYTE_DN | MORI_PRE_BI_LYMPH | 72 |
| REACTOME_GLYCEROPHOSPHOLIPID_BIOSYNTHESIS | REACTOME_GLYCEROF | 82 |
| SA_TRKA_RECEPTOR | SA_TRKA_RECEPTOR | 16 |
| ZEMBUTSU_SENSITIVITY_TO_CISPLATIN | ZEMBUTSU_SENSITIVIT | 19 |
| PID_P38_MK2_PATHWAY | PID_P38_MK2_PATHW | 21 |
| ODONNELL_TARGETS_OF_MYC_AND_TFRC_DN | ODONNELL_TARGETS_ | 42 |
| AMUNDSON_GENOTOXIC_SIGNATURE | AMUNDSON_GENOTO | 98 |
| REACTOME_FATTY_ACYL_COA_BIOSYNTHESIS | REACTOME_FATTY_AC | 18 |
| FOURNIER_ACINAR_DEVELOPMENT_LATE_2 | FOURNIER_ACINAR_DI | 259 |
| ABE_VEGFA_TARGETS_30MIN | ABE_VEGFA_TARGETS_ | 28 |
| ST_FAS_SIGNALING_PATHWAY | ST_FAS_SIGNALING_P/ | 62 |
| REACTOME_RNA_POL_I_RNA_POL_III_AND_MITOCHONDRIAL_TF | REACTOME_RNA_POL_ | 100 |
| WHITEFORD_PEDIATRIC_CANCER_MARKERS | WHITEFORD_PEDIATRI | 114 |
| SCHLOSSER_MYC_TARGETS_REPRESSED_BY_SERUM | SCHLOSSER_MYC_TAR | 148 |
| DEBOSSCHER_NFKB_TARGETS_REPRESSED_BY_GLUCOCORTICOID | DEBOSSCHER_NFKB_T/ | 22 |
| IVANOVSKA_MIR106B_TARGETS | IVANOVSKA_MIR106B_ | 82 |
| REACTOME_IRON_UPTAKE_AND_TRANSPORT | REACTOME_IRON_UPT | 32 |
| SCHUHMACHER_MYC_TARGETS_UP | SCHUHMACHER_MYC_ | 75 |
| REACTOME_ACTIVATED_TAK1_MEDIATES_P38_MAPK_ACTIVATIC | REACTOME_ACTIVATE | 17 |
| KANG_DOXORUBICIN_RESISTANCE_UP | KANG_DOXORUBICIN_ | 50 |
| ZHAN_LATE_DIFFERENTIATION_GENES_DN | ZHAN_LATE_DIFFEREN | 15 |
| MITSIADES_RESPONSE_TO_APLIDIN_DN | MITSIADES_RESPONSE | 233 |
| ROSTY_CERVICAL_CANCER_PROLIFERATION_CLUSTER | ROSTY_CERVICAL_CAN | 134 |
| CAMPS_COLON_CANCER_COPY_NUMBER_DN | CAMPS_COLON_CANC | 33 |
| KEGG_ARACHIDONIC_ACID_METABOLISM | KEGG_ARACHIDONIC_ | 45 |
| PHONG_TNF_TARGETS_UP | PHONG_TNF_TARGETS | 63 |
| PID_CASPASE_PATHWAY | PID_CASPASE_PATHW | 50 |
| WHITFIELD_CELL_CYCLE_LITERATURE | WHITFIELD_CELL_CYCL | 44 |
| SESTO_RESPONSE_TO_UV_C3 | SESTO_RESPONSE_TO_ | 20 |
| CAMPS_COLON_CANCER_COPY_NUMBER_UP | CAMPS_COLON_CANC | 78 |
| KANG_CISPLATIN_RESISTANCE_UP | KANG_CISPLATIN_RESI | 16 |
| REACTOME_POST_TRANSLATIONAL_MODIFICATION_SYNTHESIS_( | REACTOME_POST_TR/ | 26 |
| GHANDHI_DIRECT_IRRADIATION_UP | GHANDHI_DIRECT_IRR | 87 |
| MARSON_FOXP3_TARGETS_STIMULATED_UP | MARSON_FOXP3_TAR( | 29 |
| RAMALHO_STEMNESS_UP | RAMALHO_STEMNESS_ | 193 |
| LOPEZ_MBD_TARGETS_IMPRINTED_AND_X_LINKED | LOPEZ_MBD_TARGETS | 17 |
| ZHANG_RESPONSE_TO_CANTHARIDIN_UP | ZHANG_RESPONSE_TC | 19 |
| REACTOME_NOD1_2_SIGNALING_PATHWAY | REACTOME_NOD1_2_ | 29 |
| MISSIAGLIA_REGULATED_BY_METHYLATION_DN | MISSIAGLIA_REGULATI | 112 |
| REACTOME_S_PHASE | REACTOME_S_PHASE | 105 |
| GOLDRATH_ANTIGEN_RESPONSE | GOLDRATH_ANTIGEN_ | 318 |

| | | |
|---|---|---|
| VANTVEER_BREAST_CANCER_BRCA1_UP | VANTVEER_BREAST_C/ | 31 |
| KORKOLA_TERATOMA | KORKOLA_TERATOMA | 39 |
| RAGHAVACHARI_PLATELET_SPECIFIC_GENES | RAGHAVACHARI_PLAT | 66 |
| REACTOME_SULFUR_AMINO_ACID_METABOLISM | REACTOME_SULFUR_/ | 24 |
| SIG_CHEMOTAXIS | SIG_CHEMOTAXIS | 45 |
| GRANDVAUX_IRF3_TARGETS_DN | GRANDVAUX_IRF3_TA | 17 |
| HOLLERN_EMT_BREAST_TUMOR_DN | HOLLERN_EMT_BREAS | 87 |
| REACTOME_REGULATION_OF_GENE_EXPRESSION_IN_BETA_CELL | REACTOME_REGULATI | 19 |
| ZHOU_TNF_SIGNALING_30MIN | ZHOU_TNF_SIGNALIN( | 52 |
| KANNAN_TP53_TARGETS_DN | KANNAN_TP53_TARGE | 21 |
| WONG_MITOCHONDRIA_GENE_MODULE | WONG_MITOCHONDR | 212 |
| REACTOME_DEADENYLATION_DEPENDENT_MRNA_DECAY | REACTOME_DEADENY | 43 |
| PID_IL3_PATHWAY | PID_IL3_PATHWAY | 26 |
| BENPORATH_ES_2 | BENPORATH_ES_2 | 40 |
| PID_P53_DOWNSTREAM_PATHWAY | PID_P53_DOWNSTREA | 122 |
| FUJII_YBX1_TARGETS_DN | FUJII_YBX1_TARGETS_ | 190 |
| BIOCARTA_HIVNEF_PATHWAY | BIOCARTA_HIVNEF_PA | 56 |
| LE_EGR2_TARGETS_UP | LE_EGR2_TARGETS_UI | 104 |
| REACTOME_MEIOSIS | REACTOME_MEIOSIS | 93 |
| HU_ANGIOGENESIS_DN | HU_ANGIOGENESIS_DI | 34 |
| KAMMINGA_EZH2_TARGETS | KAMMINGA_EZH2_TA | 41 |
| FERRANDO_T_ALL_WITH_MLL_ENL_FUSION_DN | FERRANDO_T_ALL_WI | 81 |
| HAHTOLA_MYCOSIS_FUNGOIDES_CD4_DN | HAHTOLA_MYCOSIS_F | 107 |
| HOEGERKORP_CD44_TARGETS_DIRECT_UP | HOEGERKORP_CD44_T | 26 |
| SCIBETTA_KDM5B_TARGETS_UP | SCIBETTA_KDM5B_TAI | 15 |
| TAKEDA_TARGETS_OF_NUP98_HOXA9_FUSION_6HR_DN | TAKEDA_TARGETS_OF_ | 38 |
| REACTOME_SYNTHESIS_OF_DNA | REACTOME_SYNTHESIS | 90 |
| KEGG_RNA_DEGRADATION | KEGG_RNA_DEGRADAT | 57 |
| KASLER_HDAC7_TARGETS_1_DN | KASLER_HDAC7_TARG | 17 |
| BIOCARTA_CD40_PATHWAY | BIOCARTA_CD40_PATI | 15 |
| KIM_PTEN_TARGETS_UP | KIM_PTEN_TARGETS_I | 17 |
| WANG_TNF_TARGETS | WANG_TNF_TARGETS | 24 |
| REACTOME_NEP_NS2_INTERACTS_WITH_THE_CELLULAR_EXPORT | REACTOME_NEP_NS2_ | 27 |
| GENTILE_RESPONSE_CLUSTER_D3 | GENTILE_RESPONSE_C | 59 |
| REACTOME_MITOCHONDRIAL_TRNA_AMINOACYLATION | REACTOME_MITOCHO | 21 |
| ZHOU_CELL_CYCLE_GENES_IN_IR_RESPONSE_24HR | ZHOU_CELL_CYCLE_GI | 119 |
| MOLENAAR_TARGETS_OF_CCND1_AND_CDK4_DN | MOLENAAR_TARGETS_ | 48 |
| RASHI_NFKB1_TARGETS | RASHI_NFKB1_TARGET | 18 |
| MARIADASON_RESPONSE_TO_CURCUMIN_SULINDAC_5 | MARIADASON_RESPOI | 23 |
| REACTOME_RNA_POL_II_PRE_TRANSCRIPTION_EVENTS | REACTOME_RNA_POL_ | 50 |
| REACTOME_TAK1_ACTIVATES_NFKB_BY_PHOSPHORYLATION_AN | REACTOME_TAK1_ACT | 22 |
| AMIT_EGF_RESPONSE_480_HELA | AMIT_EGF_RESPONSE_ | 151 |
| SESTO_RESPONSE_TO_UV_C5 | SESTO_RESPONSE_TO_ | 45 |
| PID_AR_TF_PATHWAY | PID_AR_TF_PATHWAY | 51 |
| HOLLMANN_APOPTOSIS_VIA_CD40_UP | HOLLMANN_APOPTOS | 184 |
| MATZUK_IMPLANTATION_AND_UTERINE | MATZUK_IMPLANTATI | 21 |
| SOTIRIOU_BREAST_CANCER_GRADE_1_VS_3_UP | SOTIRIOU_BREAST_CA | 140 |
| HEDENFALK_BREAST_CANCER_HEREDITARY_VS_SPORADIC | HEDENFALK_BREAST_( | 47 |
| BILANGES_RAPAMYCIN_SENSITIVE_VIA_TSC1_AND_TSC2 | BILANGES_RAPAMYCII | 67 |
| REACTOME_ASSOCIATION_OF_TRIC_CCT_WITH_TARGET_PROTEII | REACTOME_ASSOCIAT | 26 |

| | | |
|---|---|---|
| LIANG_HEMATOPOIESIS_STEM_CELL_NUMBER_SMALL_VS_HUGE | LIANG_HEMATOPOIES | 31 |
| BROWNE_HCMV_INFECTION_2HR_DN | BROWNE_HCMV_INFE | 48 |
| SCIAN_INVERSED_TARGETS_OF_TP53_AND_TP73_DN | SCIAN_INVERSED_TAR | 28 |
| LINDSTEDT_DENDRITIC_CELL_MATURATION_B | LINDSTEDT_DENDRITI( | 51 |
| NIELSEN_LIPOSARCOMA_DN | NIELSEN_LIPOSARCOM | 19 |
| PID_INTEGRIN_A4B1_PATHWAY | PID_INTEGRIN_A4B1_I | 33 |
| RIZ_ERYTHROID_DIFFERENTIATION | RIZ_ERYTHROID_DIFFE | 74 |
| PID_E2F_PATHWAY | PID_E2F_PATHWAY | 69 |
| TARTE_PLASMA_CELL_VS_B_LYMPHOCYTE_DN | TARTE_PLASMA_CELL_ | 32 |
| VILIMAS_NOTCH1_TARGETS_UP | VILIMAS_NOTCH1_TAI | 50 |
| SANSOM_APC_TARGETS_REQUIRE_MYC | SANSOM_APC_TARGET | 191 |
| KIM_WT1_TARGETS_8HR_UP | KIM_WT1_TARGETS_8 | 154 |
| IWANAGA_E2F1_TARGETS_INDUCED_BY_SERUM | IWANAGA_E2F1_TARG | 29 |
| SMITH_TERT_TARGETS_UP | SMITH_TERT_TARGETS | 137 |
| REACTOME_GLOBAL_GENOMIC_NER_GG_NER | REACTOME_GLOBAL_( | 32 |
| REACTOME_TRANSCRIPTION | REACTOME_TRANSCRI | 176 |
| MANALO_HYPOXIA_DN | MANALO_HYPOXIA_DI | 274 |
| MOLENAAR_TARGETS_OF_CCND1_AND_CDK4_UP | MOLENAAR_TARGETS_ | 52 |
| MORI_LARGE_PRE_BII_LYMPHOCYTE_DN | MORI_LARGE_PRE_BII_ | 52 |
| REACTOME_TRIGLYCERIDE_BIOSYNTHESIS | REACTOME_TRIGLYCEI | 37 |
| DAZARD_UV_RESPONSE_CLUSTER_G2 | DAZARD_UV_RESPONS | 29 |
| LINDSTEDT_DENDRITIC_CELL_MATURATION_C | LINDSTEDT_DENDRITI( | 62 |
| REACTOME_AMYLOIDS | REACTOME_AMYLOIDS | 61 |
| REACTOME_TRANSPORT_OF_MATURE_TRANSCRIPT_TO_CYTOPL/ | REACTOME_TRANSPOI | 52 |
| LUND_SILENCED_BY_METHYLATION | LUND_SILENCED_BY_N | 16 |
| LEE_METASTASIS_AND_RNA_PROCESSING_UP | LEE_METASTASIS_AND | 17 |
| HEDENFALK_BREAST_CANCER_BRCA1_VS_BRCA2 | HEDENFALK_BREAST_( | 153 |
| BHAT_ESR1_TARGETS_VIA_AKT1_UP | BHAT_ESR1_TARGETS_ | 260 |
| EPPERT_LSC_R | EPPERT_LSC_R | 34 |
| STEIN_ESR1_TARGETS | STEIN_ESR1_TARGETS | 81 |
| KEGG_SMALL_CELL_LUNG_CANCER | KEGG_SMALL_CELL_LU | 82 |
| REACTOME_NFKB_AND_MAP_KINASES_ACTIVATION_MEDIATED_ | REACTOME_NFKB_ANI | 69 |
| WANG_TARGETS_OF_MLL_CBP_FUSION_UP | WANG_TARGETS_OF_I | 40 |
| VANHARANTA_UTERINE_FIBROID_DN | VANHARANTA_UTERIN | 59 |
| CEBALLOS_TARGETS_OF_TP53_AND_MYC_DN | CEBALLOS_TARGETS_( | 35 |
| KEGG_MATURITY_ONSET_DIABETES_OF_THE_YOUNG | KEGG_MATURITY_ONS | 24 |
| PUJANA_BREAST_CANCER_LIT_INT_NETWORK | PUJANA_BREAST_CAN | 96 |
| CHOW_RASSF1_TARGETS_DN | CHOW_RASSF1_TARGI | 29 |
| JUBAN_TARGETS_OF_SPI1_AND_FLI1_DN | JUBAN_TARGETS_OF_! | 85 |
| REACTOME_CELL_CELL_JUNCTION_ORGANIZATION | REACTOME_CELL_CELI | 56 |
| SUMI_HNF4A_TARGETS | SUMI_HNF4A_TARGET | 27 |
| DEURIG_T_CELL_PROLYMPHOCYTIC_LEUKEMIA_UP | DEURIG_T_CELL_PROL | 326 |
| MCMURRAY_TP53_HRAS_COOPERATION_RESPONSE_UP | MCMURRAY_TP53_HR | 25 |
| WU_APOPTOSIS_BY_CDKN1A_VIA_TP53 | WU_APOPTOSIS_BY_C | 53 |
| REACTOME_M_G1_TRANSITION | REACTOME_M_G1_TR | 77 |
| LEE_RECENT_THYMIC_EMIGRANT | LEE_RECENT_THYMIC_ | 192 |
| BROWNE_HCMV_INFECTION_12HR_UP | BROWNE_HCMV_INFE | 92 |
| YORDY_RECIPROCAL_REGULATION_BY_ETS1_AND_SP100_DN | YORDY_RECIPROCAL_I | 67 |
| ST_B_CELL_ANTIGEN_RECEPTOR | ST_B_CELL_ANTIGEN_ | 38 |
| GRADE_COLON_AND_RECTAL_CANCER_UP | GRADE_COLON_AND_ | 267 |

| | | |
|---|---|---|
| REACTOME_PERK_REGULATED_GENE_EXPRESSION | REACTOME_PERK_REG | 27 |
| SEKI_INFLAMMATORY_RESPONSE_LPS_UP | SEKI_INFLAMMATORY_ | 74 |
| SHAFFER_IRF4_TARGETS_IN_ACTIVATED_DENDRITIC_CELL | SHAFFER_IRF4_TARGE | 60 |
| HINATA_NFKB_TARGETS_FIBROBLAST_UP | HINATA_NFKB_TARGE | 81 |
| XU_HGF_SIGNALING_NOT_VIA_AKT1_6HR | XU_HGF_SIGNALING_N | 26 |
| BERENJENO_TRANSFORMED_BY_RHOA_FOREVER_DN | BERENJENO_TRANSFO | 31 |
| VARELA_ZMPSTE24_TARGETS_UP | VARELA_ZMPSTE24_TA | 37 |
| BOYLAN_MULTIPLE_MYELOMA_C_D_UP | BOYLAN_MULTIPLE_M | 128 |
| YAN_ESCAPE_FROM_ANOIKIS | YAN_ESCAPE_FROM_A | 21 |
| LABBE_TARGETS_OF_TGFB1_AND_WNT3A_DN | LABBE_TARGETS_OF_T | 98 |
| KOBAYASHI_RESPONSE_TO_ROMIDEPSIN | KOBAYASHI_RESPONSI | 18 |
| REACTOME_HIV_INFECTION | REACTOME_HIV_INFEC | 179 |
| REACTOME_CELL_CYCLE | REACTOME_CELL_CYC | 378 |
| BIOCARTA_DEATH_PATHWAY | BIOCARTA_DEATH_PA | 31 |
| WESTON_VEGFA_TARGETS_3HR | WESTON_VEGFA_TAR( | 70 |
| OUYANG_PROSTATE_CANCER_PROGRESSION_DN | OUYANG_PROSTATE_C | 19 |
| HU_GENOTOXIN_ACTION_DIRECT_VS_INDIRECT_24HR | HU_GENOTOXIN_ACTI | 49 |
| LEIN_OLIGODENDROCYTE_MARKERS | LEIN_OLIGODENDROC | 67 |
| BASSO_B_LYMPHOCYTE_NETWORK | BASSO_B_LYMPHOCYT | 138 |
| BHAT_ESR1_TARGETS_VIA_AKT1_DN | BHAT_ESR1_TARGETS_ | 77 |
| GEORGES_CELL_CYCLE_MIR192_TARGETS | GEORGES_CELL_CYCLE | 60 |
| REACTOME_LOSS_OF_NLP_FROM_MITOTIC_CENTROSOMES | REACTOME_LOSS_OF_ | 53 |
| PARK_TRETINOIN_RESPONSE_AND_RARA_PLZF_FUSION | PARK_TRETINOIN_RES | 21 |
| SLEBOS_HEAD_AND_NECK_CANCER_WITH_HPV_UP | SLEBOS_HEAD_AND_N | 74 |
| REACTOME_MYD88_MAL_CASCADE_INITIATED_ON_PLASMA_ME | REACTOME_MYD88_M | 79 |
| KEGG_NOD_LIKE_RECEPTOR_SIGNALING_PATHWAY | KEGG_NOD_LIKE_RECI | 55 |
| WORSCHECH_TUMOR_REJECTION_UP | WORSCHECH_TUMOR_ | 52 |
| MENSSEN_MYC_TARGETS | MENSSEN_MYC_TARG | 50 |
| LOCKWOOD_AMPLIFIED_IN_LUNG_CANCER | LOCKWOOD_AMPLIFIE | 200 |
| ST_JNK_MAPK_PATHWAY | ST_JNK_MAPK_PATHW | 38 |
| BILD_HRAS_ONCOGENIC_SIGNATURE | BILD_HRAS_ONCOGEN | 231 |
| REACTOME_MICRORNA_MIRNA_BIOGENESIS | REACTOME_MICRORN | 22 |
| LENAOUR_DENDRITIC_CELL_MATURATION_DN | LENAOUR_DENDRITIC_ | 117 |
| SHEPARD_BMYB_MORPHOLINO_UP | SHEPARD_BMYB_MOR | 192 |
| PID_VEGFR1_PATHWAY | PID_VEGFR1_PATHWA | 26 |
| MOOTHA_MITOCHONDRIA | MOOTHA_MITOCHON | 420 |
| SIG_PIP3_SIGNALING_IN_CARDIAC_MYOCTES | SIG_PIP3_SIGNALING_ | 66 |
| TANG_SENESCENCE_TP53_TARGETS_DN | TANG_SENESCENCE_T | 51 |
| CUI_TCF21_TARGETS_DN | CUI_TCF21_TARGETS_ | 28 |
| GALLUZZI_PERMEABILIZE_MITOCHONDRIA | GALLUZZI_PERMEABIL | 41 |
| SCHLINGEMANN_SKIN_CARCINOGENESIS_TPA_UP | SCHLINGEMANN_SKIN_ | 33 |
| RICKMAN_TUMOR_DIFFERENTIATED_WELL_VS_POORLY_DN | RICKMAN_TUMOR_DII | 339 |
| REACTOME_SYNTHESIS_OF_BILE_ACIDS_AND_BILE_SALTS | REACTOME_SYNTHESIS | 18 |
| LEE_CALORIE_RESTRICTION_NEOCORTEX_DN | LEE_CALORIE_RESTRIC | 84 |
| WATANABE_RECTAL_CANCER_RADIOTHERAPY_RESPONSIVE_UP | WATANABE_RECTAL_C | 95 |
| KEGG_GNRH_SIGNALING_PATHWAY | KEGG_GNRH_SIGNALII | 95 |
| CROONQUIST_NRAS_SIGNALING_DN | CROONQUIST_NRAS_S | 70 |
| PROVENZANI_METASTASIS_UP | PROVENZANI_METAST | 180 |
| AMUNDSON_RESPONSE_TO_ARSENITE | AMUNDSON_RESPONS | 197 |
| SCHURINGA_STAT5A_TARGETS_UP | SCHURINGA_STAT5A_ | 17 |

| | | |
|---|---|---|
| LINDGREN_BLADDER_CANCER_CLUSTER_3_UP | LINDGREN_BLADDER_( | 295 |
| ZHAN_MULTIPLE_MYELOMA_PR_UP | ZHAN_MULTIPLE_MYE | 43 |
| ZHANG_ANTIVIRAL_RESPONSE_TO_RIBAVIRIN_DN | ZHANG_ANTIVIRAL_RE | 51 |
| WANG_TARGETS_OF_MLL_CBP_FUSION_DN | WANG_TARGETS_OF_I | 43 |
| ZHONG_RESPONSE_TO_AZACITIDINE_AND_TSA_DN | ZHONG_RESPONSE_TC | 59 |
| JISON_SICKLE_CELL_DISEASE_DN | JISON_SICKLE_CELL_DI | 169 |
| WANG_ESOPHAGUS_CANCER_VS_NORMAL_DN | WANG_ESOPHAGUS_C | 95 |
| SUBTIL_PROGESTIN_TARGETS | SUBTIL_PROGESTIN_T/ | 34 |
| REACTOME_RECRUITMENT_OF_MITOTIC_CENTROSOME_PROTEII | REACTOME_RECRUITM | 60 |
| HASLINGER_B_CLL_WITH_6Q21_DELETION | HASLINGER_B_CLL_WI | 16 |
| HOFMANN_MYELODYSPLASTIC_SYNDROM_LOW_RISK_DN | HOFMANN_MYELODYS | 29 |
| FRASOR_TAMOXIFEN_RESPONSE_UP | FRASOR_TAMOXIFEN_ | 47 |
| ONO_AML1_TARGETS_UP | ONO_AML1_TARGETS_ | 23 |
| RASHI_RESPONSE_TO_IONIZING_RADIATION_1 | RASHI_RESPONSE_TO_ | 40 |
| ST_P38_MAPK_PATHWAY | ST_P38_MAPK_PATHV | 37 |
| REACTOME_MRNA_CAPPING | REACTOME_MRNA_C/ | 27 |
| CAIRO_HEPATOBLASTOMA_UP | CAIRO_HEPATOBLAST( | 188 |
| REACTOME_SYNTHESIS_AND_INTERCONVERSION_OF_NUCLEOTII | REACTOME_SYNTHESIS | 17 |
| DAZARD_RESPONSE_TO_UV_SCC_UP | DAZARD_RESPONSE_T | 110 |
| TIEN_INTESTINE_PROBIOTICS_6HR_DN | TIEN_INTESTINE_PROE | 156 |
| CROONQUIST_IL6_DEPRIVATION_DN | CROONQUIST_IL6_DEF | 95 |
| SMID_BREAST_CANCER_RELAPSE_IN_PLEURA_DN | SMID_BREAST_CANCEI | 19 |
| LEE_EARLY_T_LYMPHOCYTE_UP | LEE_EARLY_T_LYMPHC | 94 |
| YIH_RESPONSE_TO_ARSENITE_C3 | YIH_RESPONSE_TO_AI | 34 |
| HAHTOLA_SEZARY_SYNDROM_UP | HAHTOLA_SEZARY_SYI | 85 |
| ADDYA_ERYTHROID_DIFFERENTIATION_BY_HEMIN | ADDYA_ERYTHROID_D | 64 |
| REACTOME_ENDOSOMAL_SORTING_COMPLEX_REQUIRED_FOR_T | REACTOME_ENDOSON | 25 |
| MARSON_FOXP3_CORE_DIRECT_TARGETS | MARSON_FOXP3_COR | 18 |
| REACTOME_G1_PHASE | REACTOME_G1_PHASI | 33 |
| VECCHI_GASTRIC_CANCER_ADVANCED_VS_EARLY_DN | VECCHI_GASTRIC_CAN | 110 |
| KANG_DOXORUBICIN_RESISTANCE_DN | KANG_DOXORUBICIN_ | 17 |
| SENESE_HDAC1_TARGETS_UP | SENESE_HDAC1_TARG | 406 |
| BIOCARTA_NFKB_PATHWAY | BIOCARTA_NFKB_PATI | 23 |
| KIM_MYC_AMPLIFICATION_TARGETS_UP | KIM_MYC_AMPLIFICAT | 183 |
| BOYAULT_LIVER_CANCER_SUBCLASS_G12_UP | BOYAULT_LIVER_CAN( | 36 |
| BOYAULT_LIVER_CANCER_SUBCLASS_G5_DN | BOYAULT_LIVER_CAN( | 22 |
| SCHLOSSER_MYC_TARGETS_AND_SERUM_RESPONSE_UP | SCHLOSSER_MYC_TAR | 45 |
| FERREIRA_EWINGS_SARCOMA_UNSTABLE_VS_STABLE_UP | FERREIRA_EWINGS_SA | 152 |
| NOUZOVA_METHYLATED_IN_APL | NOUZOVA_METHYLAT | 55 |
| ZEMBUTSU_SENSITIVITY_TO_VINCRISTINE | ZEMBUTSU_SENSITIVI1 | 18 |
| YU_MYC_TARGETS_UP | YU_MYC_TARGETS_UF | 40 |
| LIU_COMMON_CANCER_GENES | LIU_COMMON_CANCE | 63 |
| BIOCARTA_MTA3_PATHWAY | BIOCARTA_MTA3_PAT | 17 |
| GENTILE_UV_LOW_DOSE_UP | GENTILE_UV_LOW_DC | 25 |
| HEIDENBLAD_AMPLICON_12P11_12_DN | HEIDENBLAD_AMPLIC( | 21 |
| ONDER_CDH1_TARGETS_2_DN | ONDER_CDH1_TARGET | 420 |
| KEGG_PORPHYRIN_AND_CHLOROPHYLL_METABOLISM | KEGG_PORPHYRIN_AN | 29 |
| JAZAG_TGFB1_SIGNALING_VIA_SMAD4_DN | JAZAG_TGFB1_SIGNAL | 58 |
| KIM_ALL_DISORDERS_CALB1_CORR_DN | KIM_ALL_DISORDERS_ | 33 |
| MIDORIKAWA_AMPLIFIED_IN_LIVER_CANCER | MIDORIKAWA_AMPLIF | 50 |

| | | |
|---|---|---|
| LOPES_METHYLATED_IN_COLON_CANCER_DN | LOPES_METHYLATED_ | 26 |
| HASLINGER_B_CLL_WITH_CHROMOSOME_12_TRISOMY | HASLINGER_B_CLL_WI | 23 |
| CHEMNITZ_RESPONSE_TO_PROSTAGLANDIN_E2_UP | CHEMNITZ_RESPONSE_ | 129 |
| ZUCCHI_METASTASIS_DN | ZUCCHI_METASTASIS_ | 40 |
| KEGG_MAPK_SIGNALING_PATHWAY | KEGG_MAPK_SIGNALI | 257 |
| REACTOME_MITOTIC_PROMETAPHASE | REACTOME_MITOTIC_ | 85 |
| DOANE_RESPONSE_TO_ANDROGEN_UP | DOANE_RESPONSE_TO | 152 |
| YANG_BREAST_CANCER_ESR1_BULK_UP | YANG_BREAST_CANCE | 24 |
| PICCALUGA_ANGIOIMMUNOBLASTIC_LYMPHOMA_DN | PICCALUGA_ANGIOIM | 126 |
| RICKMAN_TUMOR_DIFFERENTIATED_WELL_VS_POORLY_UP | RICKMAN_TUMOR_DI | 210 |
| PID_RHODOPSIN_PATHWAY | PID_RHODOPSIN_PATH | 23 |
| FLECHNER_BIOPSY_KIDNEY_TRANSPLANT_OK_VS_DONOR_DN | FLECHNER_BIOPSY_KI | 23 |
| PID_LKB1_PATHWAY | PID_LKB1_PATHWAY | 45 |
| REACTOME_DNA_REPLICATION | REACTOME_DNA_REPI | 186 |
| REACTOME_MITOTIC_G2_G2_M_PHASES | REACTOME_MITOTIC_ | 75 |
| HOFMANN_CELL_LYMPHOMA_UP | HOFMANN_CELL_LYM | 46 |
| KENNY_CTNNB1_TARGETS_UP | KENNY_CTNNB1_TAR | 47 |
| REACTOME_ASSEMBLY_OF_THE_PRE_REPLICATIVE_COMPLEX | REACTOME_ASSEMBL | 63 |
| BURTON_ADIPOGENESIS_2 | BURTON_ADIPOGENES | 72 |
| SHIN_B_CELL_LYMPHOMA_CLUSTER_3 | SHIN_B_CELL_LYMPH | 28 |
| RICKMAN_HEAD_AND_NECK_CANCER_C | RICKMAN_HEAD_AND_ | 89 |
| ZHANG_TLX_TARGETS_36HR_DN | ZHANG_TLX_TARGETS_ | 174 |
| AUNG_GASTRIC_CANCER | AUNG_GASTRIC_CANC | 50 |
| PID_MYC_PATHWAY | PID_MYC_PATHWAY | 24 |
| FAELT_B_CLL_WITH_VH3_21_UP | FAELT_B_CLL_WITH_V | 42 |
| HOOI_ST7_TARGETS_UP | HOOI_ST7_TARGETS_ | 75 |
| TONKS_TARGETS_OF_RUNX1_RUNX1T1_FUSION_MONOCYTE_UP | TONKS_TARGETS_OF_ | 190 |
| FISCHER_G2_M_CELL_CYCLE | FISCHER_G2_M_CELL_ | 215 |
| PID_MTOR_4PATHWAY | PID_MTOR_4PATHWA | 68 |
| XU_RESPONSE_TO_TRETINOIN_AND_NSC682994_UP | XU_RESPONSE_TO_TR | 16 |
| REACTOME_METABOLISM_OF_NON_CODING_RNA | REACTOME_METABOL | 47 |
| ROYLANCE_BREAST_CANCER_16Q_COPY_NUMBER_UP | ROYLANCE_BREAST_C | 51 |
| FLECHNER_PBL_KIDNEY_TRANSPLANT_REJECTED_VS_OK_DN | FLECHNER_PBL_KIDNE | 49 |
| CEBALLOS_TARGETS_OF_TP53_AND_MYC_UP | CEBALLOS_TARGETS_ | 20 |
| RICKMAN_METASTASIS_UP | RICKMAN_METASTASI | 289 |
| KASLER_HDAC7_TARGETS_1_UP | KASLER_HDAC7_TARG | 186 |
| SHAFFER_IRF4_TARGETS_IN_PLASMA_CELL_VS_MATURE_B_LYM | SHAFFER_IRF4_TARGE | 65 |
| BENPORATH_ES_CORE_NINE_CORRELATED | BENPORATH_ES_CORE | 98 |
| FONTAINE_PAPILLARY_THYROID_CARCINOMA_UP | FONTAINE_PAPILLARY_ | 58 |
| BIOCARTA_CHREBP2_PATHWAY | BIOCARTA_CHREBP2_ | 40 |
| REACTOME_CELL_CYCLE_MITOTIC | REACTOME_CELL_CYC | 301 |
| ZHAN_MULTIPLE_MYELOMA_CD1_DN | ZHAN_MULTIPLE_MYE | 37 |
| RUIZ_TNC_TARGETS_DN | RUIZ_TNC_TARGETS_ | 137 |
| DELPUECH_FOXO3_TARGETS_UP | DELPUECH_FOXO3_TA | 63 |
| BIOCARTA_FAS_PATHWAY | BIOCARTA_FAS_PATH | 28 |
| TOYOTA_TARGETS_OF_MIR34B_AND_MIR34C | TOYOTA_TARGETS_OF | 395 |
| CONCANNON_APOPTOSIS_BY_EPOXOMICIN_UP | CONCANNON_APOPT | 220 |
| BIOCARTA_IL12_PATHWAY | BIOCARTA_IL12_PATH | 21 |
| UROSEVIC_RESPONSE_TO_IMIQUIMOD | UROSEVIC_RESPONSE_ | 17 |
| HEIDENBLAD_AMPLICON_8Q24_UP | HEIDENBLAD_AMPLIC | 30 |

| | | |
|---|---|---|
| BURTON_ADIPOGENESIS_3 | BURTON_ADIPOGENES | 98 |
| STEIN_ESRRA_TARGETS_UP | STEIN_ESRRA_TARGET | 345 |
| ACEVEDO_LIVER_TUMOR_VS_NORMAL_ADJACENT_TISSUE_DN | ACEVEDO_LIVER_TUM | 234 |
| KEGG_GLYOXYLATE_AND_DICARBOXYLATE_METABOLISM | KEGG_GLYOXYLATE_AI | 16 |
| AMIT_SERUM_RESPONSE_120_MCF10A | AMIT_SERUM_RESPON | 61 |
| IVANOVA_HEMATOPOIESIS_EARLY_PROGENITOR | IVANOVA_HEMATOPO | 472 |
| BILD_E2F3_ONCOGENIC_SIGNATURE | BILD_E2F3_ONCOGEN | 211 |
| KIM_WT1_TARGETS_DN | KIM_WT1_TARGETS_D | 422 |
| VILLANUEVA_LIVER_CANCER_KRT19_UP | VILLANUEVA_LIVER_C | 155 |
| CHARAFE_BREAST_CANCER_BASAL_VS_MESENCHYMAL_UP | CHARAFE_BREAST_CAI | 103 |
| REACTOME_N_GLYCAN_ANTENNAE_ELONGATION_IN_THE_MEDI | REACTOME_N_GLYCAI | 18 |
| ZWANG_CLASS_3_TRANSIENTLY_INDUCED_BY_EGF | ZWANG_CLASS_3_TRA | 209 |
| ZHAN_MULTIPLE_MYELOMA_PR_DN | ZHAN_MULTIPLE_MYE | 44 |
| WEST_ADRENOCORTICAL_CARCINOMA_VS_ADENOMA_DN | WEST_ADRENOCORTIC | 17 |
| SPIRA_SMOKERS_LUNG_CANCER_UP | SPIRA_SMOKERS_LUN | 36 |
| SCHUETZ_BREAST_CANCER_DUCTAL_INVASIVE_DN | SCHUETZ_BREAST_CAI | 77 |
| REACTOME_FORMATION_OF_THE_HIV1_EARLY_ELONGATION_C | REACTOME_FORMATIO | 26 |
| CHANG_CORE_SERUM_RESPONSE_UP | CHANG_CORE_SERUM | 201 |
| PID_FANCONI_PATHWAY | PID_FANCONI_PATHW | 45 |
| GESERICK_TERT_TARGETS_DN | GESERICK_TERT_TARG | 21 |
| REACTOME_DEADENYLATION_OF_MRNA | REACTOME_DEADENY | 18 |
| ZWANG_EGF_PERSISTENTLY_DN | ZWANG_EGF_PERSISTI | 50 |
| CREIGHTON_ENDOCRINE_THERAPY_RESISTANCE_1 | CREIGHTON_ENDOCRI | 468 |
| ZHAN_MULTIPLE_MYELOMA_SUBGROUPS | ZHAN_MULTIPLE_MYE | 30 |
| KEGG_SYSTEMIC_LUPUS_ERYTHEMATOSUS | KEGG_SYSTEMIC_LUPU | 91 |
| IVANOVA_HEMATOPOIESIS_LATE_PROGENITOR | IVANOVA_HEMATOPO | 493 |
| WILLIAMS_ESR2_TARGETS_UP | WILLIAMS_ESR2_TARG | 25 |
| SASAKI_ADULT_T_CELL_LEUKEMIA | SASAKI_ADULT_T_CELI | 163 |
| KEGG_TYPE_I_DIABETES_MELLITUS | KEGG_TYPE_I_DIABETI | 21 |
| HUMMERICH_BENIGN_SKIN_TUMOR_DN | HUMMERICH_BENIGN_ | 18 |
| MARTENS_BOUND_BY_PML_RARA_FUSION | MARTENS_BOUND_BY | 414 |
| REACTOME_FORMATION_OF_TRANSCRIPTION_COUPLED_NER_T | REACTOME_FORMATIO | 28 |
| PIONTEK_PKD1_TARGETS_UP | PIONTEK_PKD1_TARGI | 32 |
| GARCIA_TARGETS_OF_FLI1_AND_DAX1_DN | GARCIA_TARGETS_OF_ | 150 |
| STEIN_ESRRA_TARGETS | STEIN_ESRRA_TARGET | 468 |
| LANDIS_ERBB2_BREAST_TUMORS_65_UP | LANDIS_ERBB2_BREAS | 21 |
| GRABARCZYK_BCL11B_TARGETS_UP | GRABARCZYK_BCL11B_ | 67 |
| MORI_IMMATURE_B_LYMPHOCYTE_UP | MORI_IMMATURE_B_ | 49 |
| SETLUR_PROSTATE_CANCER_TMPRSS2_ERG_FUSION_DN | SETLUR_PROSTATE_CA | 18 |
| REACTOME_HOMOLOGOUS_RECOMBINATION_REPAIR_OF_REPLI | REACTOME_HOMOLO | 15 |
| MARTINEZ_RESPONSE_TO_TRABECTEDIN_DN | MARTINEZ_RESPONSE_ | 250 |
| LANDEMAINE_LUNG_METASTASIS | LANDEMAINE_LUNG_I | 18 |
| REACTOME_RNA_POL_III_TRANSCRIPTION_INITIATION_FROM_TY | REACTOME_RNA_POL_ | 25 |
| WINNEPENNINCKX_MELANOMA_METASTASIS_UP | WINNEPENNINCKX_MI | 149 |
| GAVIN_FOXP3_TARGETS_CLUSTER_P6 | GAVIN_FOXP3_TARGE | 87 |
| ST_GA12_PATHWAY | ST_GA12_PATHWAY | 23 |
| HUTTMANN_B_CLL_POOR_SURVIVAL_DN | HUTTMANN_B_CLL_P | 53 |
| BHATI_G2M_ARREST_BY_2METHOXYESTRADIOL_UP | BHATI_G2M_ARREST_ | 109 |
| SA_CASPASE_CASCADE | SA_CASPASE_CASCADI | 17 |
| IKEDA_MIR30_TARGETS_DN | IKEDA_MIR30_TARGET | 27 |

| | | |
|---|---|---|
| YANG_BREAST_CANCER_ESR1_LASER_UP | YANG_BREAST_CANCE | 30 |
| JEON_SMAD6_TARGETS_DN | JEON_SMAD6_TARGET | 18 |
| KEGG_ALDOSTERONE_REGULATED_SODIUM_REABSORPTION | KEGG_ALDOSTERONE_ | 41 |
| RASHI_RESPONSE_TO_IONIZING_RADIATION_2 | RASHI_RESPONSE_TO_ | 124 |
| REACTOME_TRANS_GOLGI_NETWORK_VESICLE_BUDDING | REACTOME_TRANS_G( | 54 |
| SANA_RESPONSE_TO_IFNG_DN | SANA_RESPONSE_TO_ | 80 |
| TAVOR_CEBPA_TARGETS_DN | TAVOR_CEBPA_TARGE | 26 |
| WALLACE_JAK2_TARGETS_UP | WALLACE_JAK2_TARG | 24 |
| LI_WILMS_TUMOR_ANAPLASTIC_UP | LI_WILMS_TUMOR_AN | 19 |
| SABATES_COLORECTAL_ADENOMA_UP | SABATES_COLORECTAI | 117 |
| RIZ_ERYTHROID_DIFFERENTIATION_CCNE1 | RIZ_ERYTHROID_DIFFE | 39 |
| BREDEMEYER_RAG_SIGNALING_NOT_VIA_ATM_DN | BREDEMEYER_RAG_SI( | 53 |
| YAGI_AML_RELAPSE_PROGNOSIS | YAGI_AML_RELAPSE_F | 34 |
| IRITANI_MAD1_TARGETS_DN | IRITANI_MAD1_TARGE | 46 |
| HUMMEL_BURKITTS_LYMPHOMA_UP | HUMMEL_BURKITTS_L | 36 |
| ZHOU_INFLAMMATORY_RESPONSE_FIMA_DN | ZHOU_INFLAMMATOR | 228 |
| SHEPARD_CRUSH_AND_BURN_MUTANT_UP | SHEPARD_CRUSH_AND | 177 |
| ABE_VEGFA_TARGETS | ABE_VEGFA_TARGETS | 19 |
| LAU_APOPTOSIS_CDKN2A_UP | LAU_APOPTOSIS_CDKI | 51 |
| ST_GAQ_PATHWAY | ST_GAQ_PATHWAY | 27 |
| RIZ_ERYTHROID_DIFFERENTIATION_HBZ | RIZ_ERYTHROID_DIFFE | 40 |
| MAGRANGEAS_MULTIPLE_MYELOMA_IGG_VS_IGA_UP | MAGRANGEAS_MULTI | 19 |
| REACTOME_RNA_POL_III_TRANSCRIPTION | REACTOME_RNA_POL_ | 32 |
| MA_MYELOID_DIFFERENTIATION_DN | MA_MYELOID_DIFFERI | 44 |
| REACTOME_MEIOTIC_SYNAPSIS | REACTOME_MEIOTIC_ | 58 |
| RAY_TUMORIGENESIS_BY_ERBB2_CDC25A_UP | RAY_TUMORIGENESIS_ | 98 |
| KEGG_UBIQUITIN_MEDIATED_PROTEOLYSIS | KEGG_UBIQUITIN_MEI | 129 |
| REACTOME_MITOTIC_M_M_G1_PHASES | REACTOME_MITOTIC_ | 166 |
| PID_FOXM1_PATHWAY | PID_FOXM1_PATHWA | 40 |
| BRACHAT_RESPONSE_TO_CAMPTOTHECIN_UP | BRACHAT_RESPONSE_ | 23 |
| JOHNSTONE_PARVB_TARGETS_2_DN | JOHNSTONE_PARVB_T | 307 |
| MIKKELSEN_MCV6_LCP_WITH_H3K27ME3 | MIKKELSEN_MCV6_LC | 21 |
| PID_CONE_PATHWAY | PID_CONE_PATHWAY | 21 |
| BREDEMEYER_RAG_SIGNALING_VIA_ATM_NOT_VIA_NFKB_DN | BREDEMEYER_RAG_SI( | 35 |
| XU_HGF_TARGETS_INDUCED_BY_AKT1_6HR | XU_HGF_TARGETS_INI | 17 |
| GRAESSMANN_RESPONSE_TO_MC_AND_SERUM_DEPRIVATION_I | GRAESSMANN_RESPOI | 75 |
| LABBE_WNT3A_TARGETS_UP | LABBE_WNT3A_TARGE | 107 |
| GUO_TARGETS_OF_IRS1_AND_IRS2 | GUO_TARGETS_OF_IR! | 89 |
| REACTOME_GRB2_SOS_PROVIDES_LINKAGE_TO_MAPK_SIGNALII | REACTOME_GRB2_SO! | 15 |
| NOUZOVA_TRETINOIN_AND_H4_ACETYLATION | NOUZOVA_TRETINOIN | 122 |
| STEARMAN_LUNG_CANCER_EARLY_VS_LATE_UP | STEARMAN_LUNG_CA | 115 |
| DANG_REGULATED_BY_MYC_UP | DANG_REGULATED_BY | 64 |
| KEGG_CELL_CYCLE | KEGG_CELL_CYCLE | 121 |
| SWEET_LUNG_CANCER_KRAS_UP | SWEET_LUNG_CANCEI | 453 |
| PEART_HDAC_PROLIFERATION_CLUSTER_DN | PEART_HDAC_PROLIFE | 71 |
| MARKS_HDAC_TARGETS_UP | MARKS_HDAC_TARGET | 18 |
| LANDIS_ERBB2_BREAST_TUMORS_324_UP | LANDIS_ERBB2_BREAS | 138 |
| GEORGANTAS_HSC_MARKERS | GEORGANTAS_HSC_M | 60 |
| GHANDHI_BYSTANDER_IRRADIATION_UP | GHANDHI_BYSTANDEF | 72 |
| REACTOME_CELL_DEATH_SIGNALLING_VIA_NRAGE_NRIF_AND_N | REACTOME_CELL_DEA | 58 |

| | | |
|---|---|---|
| STEIN_ESRRA_TARGETS_RESPONSIVE_TO_ESTROGEN_DN | STEIN_ESRRA_TARGET | 39 |
| MORI_IMMATURE_B_LYMPHOCYTE_DN | MORI_IMMATURE_B_ | 88 |
| REACTOME_SHC_MEDIATED_CASCADE | REACTOME_SHC_MED | 26 |
| GINESTIER_BREAST_CANCER_ZNF217_AMPLIFIED_DN | GINESTIER_BREAST_CA | 290 |
| SARTIPY_NORMAL_AT_INSULIN_RESISTANCE_UP | SARTIPY_NORMAL_AT_ | 33 |
| REACTOME_INTRINSIC_PATHWAY_FOR_APOPTOSIS | REACTOME_INTRINSIC | 28 |
| PEDERSEN_METASTASIS_BY_ERBB2_ISOFORM_6 | PEDERSEN_METASTAS | 26 |
| YAO_TEMPORAL_RESPONSE_TO_PROGESTERONE_CLUSTER_5 | YAO_TEMPORAL_RESP | 28 |
| MCBRYAN_PUBERTAL_BREAST_5_6WK_UP | MCBRYAN_PUBERTAL_ | 110 |
| PID_IL23_PATHWAY | PID_IL23_PATHWAY | 37 |
| WINTER_HYPOXIA_UP | WINTER_HYPOXIA_UP | 85 |
| KEGG_RNA_POLYMERASE | KEGG_RNA_POLYMERA | 27 |
| GRABARCZYK_BCL11B_TARGETS_DN | GRABARCZYK_BCL11B_ | 52 |
| GEISS_RESPONSE_TO_DSRNA_UP | GEISS_RESPONSE_TO_ | 35 |
| ST_T_CELL_SIGNAL_TRANSDUCTION | ST_T_CELL_SIGNAL_TR | 45 |
| GROSS_ELK3_TARGETS_DN | GROSS_ELK3_TARGETS | 32 |
| SAGIV_CD24_TARGETS_UP | SAGIV_CD24_TARGETS | 21 |
| REACTOME_PYRUVATE_METABOLISM_AND_CITRIC_ACID_TCA_C | REACTOME_PYRUVATE | 39 |
| REACTOME_ORC1_REMOVAL_FROM_CHROMATIN | REACTOME_ORC1_REM | 65 |
| PID_MYC_ACTIV_PATHWAY | PID_MYC_ACTIV_PATH | 77 |
| GARGALOVIC_RESPONSE_TO_OXIDIZED_PHOSPHOLIPIDS_YELLOV | GARGALOVIC_RESPON | 25 |
| MIKKELSEN_ES_ICP_WITH_H3K27ME3 | MIKKELSEN_ES_ICP_W | 40 |
| GOTZMANN_EPITHELIAL_TO_MESENCHYMAL_TRANSITION_DN | GOTZMANN_EPITHELIA | 196 |
| KEGG_VEGF_SIGNALING_PATHWAY | KEGG_VEGF_SIGNALIN | 73 |
| REACTOME_RNA_POL_II_TRANSCRIPTION | REACTOME_RNA_POL_ | 91 |
| MORI_LARGE_PRE_BII_LYMPHOCYTE_UP | MORI_LARGE_PRE_BII_ | 81 |
| REACTOME_AMINO_ACID_SYNTHESIS_AND_INTERCONVERSION_ | REACTOME_AMINO_A | 16 |
| HOLLERN_SQUAMOUS_BREAST_TUMOR | HOLLERN_SQUAMOUS | 111 |
| DORN_ADENOVIRUS_INFECTION_12HR_DN | DORN_ADENOVIRUS_I | 33 |
| RATTENBACHER_BOUND_BY_CELF1 | RATTENBACHER_BOUN | 361 |
| AIGNER_ZEB1_TARGETS | AIGNER_ZEB1_TARGET | 34 |
| FARMER_BREAST_CANCER_CLUSTER_7 | FARMER_BREAST_CAN | 18 |
| SCHLOSSER_MYC_TARGETS_AND_SERUM_RESPONSE_DN | SCHLOSSER_MYC_TAR | 46 |
| DAIRKEE_TERT_TARGETS_UP | DAIRKEE_TERT_TARGE | 329 |
| WANG_CISPLATIN_RESPONSE_AND_XPC_UP | WANG_CISPLATIN_RES | 183 |
| XU_CREBBP_TARGETS_UP | XU_CREBBP_TARGETS_ | 24 |
| HEIDENBLAD_AMPLICON_8Q24_DN | HEIDENBLAD_AMPLIC | 42 |
| REACTOME_FANCONI_ANEMIA_PATHWAY | REACTOME_FANCONI_ | 19 |
| PID_ERA_GENOMIC_PATHWAY | PID_ERA_GENOMIC_P | 64 |
| REACTOME_SIGNALING_BY_THE_B_CELL_RECEPTOR_BCR | REACTOME_SIGNALIN | 119 |
| SHEDDEN_LUNG_CANCER_POOR_SURVIVAL_A6 | SHEDDEN_LUNG_CAN | 422 |
| BEIER_GLIOMA_STEM_CELL_DN | BEIER_GLIOMA_STEM_ | 53 |
| BROWNE_HCMV_INFECTION_6HR_DN | BROWNE_HCMV_INFE | 146 |
| YAO_TEMPORAL_RESPONSE_TO_PROGESTERONE_CLUSTER_6 | YAO_TEMPORAL_RESP | 67 |
| PID_CD40_PATHWAY | PID_CD40_PATHWAY | 30 |
| BROWNE_HCMV_INFECTION_10HR_UP | BROWNE_HCMV_INFE | 86 |
| RASHI_RESPONSE_TO_IONIZING_RADIATION_3 | RASHI_RESPONSE_TO_ | 45 |
| FARMER_BREAST_CANCER_BASAL_VS_LULMINAL | FARMER_BREAST_CAN | 304 |
| ONO_FOXP3_TARGETS_DN | ONO_FOXP3_TARGETS | 39 |
| RODWELL_AGING_KIDNEY_DN | RODWELL_AGING_KID | 125 |

| | | |
|---|---|---|
| ODONNELL_TFRC_TARGETS_DN | ODONNELL_TFRC_TAR | 120 |
| KEGG_FC_EPSILON_RI_SIGNALING_PATHWAY | KEGG_FC_EPSILON_RI_ | 77 |
| REACTOME_APOPTOTIC_EXECUTION_PHASE | REACTOME_APOPTOT | 49 |
| KEGG_ARGININE_AND_PROLINE_METABOLISM | KEGG_ARGININE_AND_ | 51 |
| CHESLER_BRAIN_HIGHEST_EXPRESSION | CHESLER_BRAIN_HIGH | 37 |
| DALESSIO_TSA_RESPONSE | DALESSIO_TSA_RESPO | 20 |
| KEGG_DORSO_VENTRAL_AXIS_FORMATION | KEGG_DORSO_VENTRA | 22 |
| KEGG_REGULATION_OF_AUTOPHAGY | KEGG_REGULATION_C | 28 |
| JIANG_VHL_TARGETS | JIANG_VHL_TARGETS | 122 |
| GENTILE_UV_RESPONSE_CLUSTER_D4 | GENTILE_UV_RESPONS | 51 |
| REACTOME_ABORTIVE_ELONGATION_OF_HIV1_TRANSCRIPT_IN_ | REACTOME_ABORTIVE | 17 |
| REN_ALVEOLAR_RHABDOMYOSARCOMA_UP | REN_ALVEOLAR_RHAB | 96 |
| APPIERTO_RESPONSE_TO_FENRETINIDE_UP | APPIERTO_RESPONSE_ | 35 |
| MULLIGHAN_NPM1_MUTATED_SIGNATURE_2_DN | MULLIGHAN_NPM1_M | 72 |
| MARKEY_RB1_ACUTE_LOF_UP | MARKEY_RB1_ACUTE_ | 219 |
| BECKER_TAMOXIFEN_RESISTANCE_DN | BECKER_TAMOXIFEN_ | 48 |
| SIG_CD40PATHWAYMAP | SIG_CD40PATHWAYM | 34 |
| MAYBURD_RESPONSE_TO_L663536_DN | MAYBURD_RESPONSE_ | 48 |
| LY_AGING_PREMATURE_DN | LY_AGING_PREMATUR | 29 |
| BIOCARTA_PITX2_PATHWAY | BIOCARTA_PITX2_PAT | 15 |
| CHARAFE_BREAST_CANCER_LUMINAL_VS_MESENCHYMAL_UP | CHARAFE_BREAST_CA | 393 |
| VANASSE_BCL2_TARGETS_UP | VANASSE_BCL2_TARG | 35 |
| HALMOS_CEBPA_TARGETS_DN | HALMOS_CEBPA_TAR | 43 |
| STAMBOLSKY_BOUND_BY_MUTATED_TP53 | STAMBOLSKY_BOUND_ | 15 |
| ST_PHOSPHOINOSITIDE_3_KINASE_PATHWAY | ST_PHOSPHOINOSITID | 37 |
| MOHANKUMAR_HOXA1_TARGETS_UP | MOHANKUMAR_HOXA | 385 |
| XU_RESPONSE_TO_TRETINOIN_AND_NSC682994_DN | XU_RESPONSE_TO_TR | 15 |
| REACTOME_PREFOLDIN_MEDIATED_TRANSFER_OF_SUBSTRATE_ | REACTOME_PREFOLDI | 24 |
| HORIUCHI_WTAP_TARGETS_DN | HORIUCHI_WTAP_TAR | 287 |
| ZWANG_CLASS_1_TRANSIENTLY_INDUCED_BY_EGF | ZWANG_CLASS_1_TRA | 454 |
| HIRSCH_CELLULAR_TRANSFORMATION_SIGNATURE_UP | HIRSCH_CELLULAR_TR | 224 |
| GOLDRATH_HOMEOSTATIC_PROLIFERATION | GOLDRATH_HOMEOST | 158 |
| KYNG_DNA_DAMAGE_BY_UV | KYNG_DNA_DAMAGE_ | 60 |
| BROWNE_HCMV_INFECTION_30MIN_DN | BROWNE_HCMV_INFE | 130 |
| REACTOME_BILE_ACID_AND_BILE_SALT_METABOLISM | REACTOME_BILE_ACID | 23 |
| GAZDA_DIAMOND_BLACKFAN_ANEMIA_PROGENITOR_UP | GAZDA_DIAMOND_BL | 33 |
| NIKOLSKY_BREAST_CANCER_16P13_AMPLICON | NIKOLSKY_BREAST_CA | 100 |
| PENG_RAPAMYCIN_RESPONSE_DN | PENG_RAPAMYCIN_RE | 229 |
| SCIAN_CELL_CYCLE_TARGETS_OF_TP53_AND_TP73_DN | SCIAN_CELL_CYCLE_T | 22 |
| PID_NEPHRIN_NEPH1_PATHWAY | PID_NEPHRIN_NEPH1_ | 31 |
| KEGG_P53_SIGNALING_PATHWAY | KEGG_P53_SIGNALING | 62 |
| FLECHNER_PBL_KIDNEY_TRANSPLANT_OK_VS_DONOR_UP | FLECHNER_PBL_KIDNE | 140 |
| TIMOFEEVA_GROWTH_STRESS_VIA_STAT1_DN | TIMOFEEVA_GROWTH | 15 |
| MULLIGAN_NTF3_SIGNALING_VIA_INSR_AND_IGF1R_UP | MULLIGAN_NTF3_SIGN | 23 |
| PAPASPYRIDONOS_UNSTABLE_ATEROSCLEROTIC_PLAQUE_UP | PAPASPYRIDONOS_UN | 47 |
| GROSS_HYPOXIA_VIA_ELK3_AND_HIF1A_UP | GROSS_HYPOXIA_VIA_ | 137 |
| BIOCARTA_AKT_PATHWAY | BIOCARTA_AKT_PATH | 20 |
| GREENBAUM_E2A_TARGETS_DN | GREENBAUM_E2A_TA | 19 |
| PID_ATM_PATHWAY | PID_ATM_PATHWAY | 33 |
| PEDERSEN_METASTASIS_BY_ERBB2_ISOFORM_7 | PEDERSEN_METASTAS | 341 |

| | | |
|---|---|---|
| JAZAG_TGFB1_SIGNALING_UP | JAZAG_TGFB1_SIGNAL | 100 |
| BOYLAN_MULTIPLE_MYELOMA_C_CLUSTER_DN | BOYLAN_MULTIPLE_M | 29 |
| MILI_PSEUDOPODIA_HAPTOTAXIS_UP | MILI_PSEUDOPODIA_H | 468 |
| REACTOME_DOWNSTREAM_SIGNALING_EVENTS_OF_B_CELL_RE | REACTOME_DOWNSTR | 90 |
| BIOCARTA_P53HYPOXIA_PATHWAY | BIOCARTA_P53HYPOXI | 20 |
| QUINTENS_EMBRYONIC_BRAIN_RESPONSE_TO_IR | QUINTENS_EMBRYONI | 69 |
| BANDRES_RESPONSE_TO_CARMUSTIN_MGMT_48HR_DN | BANDRES_RESPONSE_ | 150 |
| HESS_TARGETS_OF_HOXA9_AND_MEIS1_UP | HESS_TARGETS_OF_H | 64 |
| GARGALOVIC_RESPONSE_TO_OXIDIZED_PHOSPHOLIPIDS_TURQU | GARGALOVIC_RESPON | 46 |
| GOLUB_ALL_VS_AML_UP | GOLUB_ALL_VS_AML_ | 23 |
| REACTOME_FORMATION_OF_RNA_POL_II_ELONGATION_COMPL | REACTOME_FORMATI | 35 |
| PID_THROMBIN_PAR4_PATHWAY | PID_THROMBIN_PAR4 | 15 |
| GRAHAM_CML_DIVIDING_VS_NORMAL_QUIESCENT_UP | GRAHAM_CML_DIVIDI | 171 |
| WHITFIELD_CELL_CYCLE_G2_M | WHITFIELD_CELL_CYCL | 203 |
| GENTILE_UV_RESPONSE_CLUSTER_D2 | GENTILE_UV_RESPONS | 39 |
| SCHEIDEREIT_IKK_INTERACTING_PROTEINS | SCHEIDEREIT_IKK_INTE | 58 |
| REACTOME_CELL_CYCLE_CHECKPOINTS | REACTOME_CELL_CYC | 110 |
| WATANABE_COLON_CANCER_MSI_VS_MSS_UP | WATANABE_COLON_C | 25 |
| OUELLET_OVARIAN_CANCER_INVASIVE_VS_LMP_UP | OUELLET_OVARIAN_CA | 112 |
| REACTOME_PKA_MEDIATED_PHOSPHORYLATION_OF_CREB | REACTOME_PKA_MED | 16 |
| WONG_EMBRYONIC_STEM_CELL_CORE | WONG_EMBRYONIC_S | 326 |
| CHIARETTI_T_ALL_REFRACTORY_TO_THERAPY | CHIARETTI_T_ALL_REF | 28 |
| YOKOE_CANCER_TESTIS_ANTIGENS | YOKOE_CANCER_TEST | 31 |
| CAFFAREL_RESPONSE_TO_THC_24HR_5_UP | CAFFAREL_RESPONSE_ | 30 |
| LASTOWSKA_COAMPLIFIED_WITH_MYCN | LASTOWSKA_COAMPL | 38 |
| KYNG_NORMAL_AGING_UP | KYNG_NORMAL_AGIN | 15 |
| HASLINGER_B_CLL_WITH_17P13_DELETION | HASLINGER_B_CLL_WI | 19 |
| REACTOME_SIGNALING_BY_WNT | REACTOME_SIGNALIN | 61 |
| REACTOME_MRNA_3_END_PROCESSING | REACTOME_MRNA_3_ | 33 |
| HUNSBERGER_EXERCISE_REGULATED_GENES | HUNSBERGER_EXERCIS | 31 |
| CHEOK_RESPONSE_TO_MERCAPTOPURINE_AND_LD_MTX_DN | CHEOK_RESPONSE_TO | 17 |
| XU_GH1_AUTOCRINE_TARGETS_UP | XU_GH1_AUTOCRINE_ | 216 |
| FRASOR_RESPONSE_TO_ESTRADIOL_DN | FRASOR_RESPONSE_T | 76 |
| BOYLAN_MULTIPLE_MYELOMA_D_CLUSTER_UP | BOYLAN_MULTIPLE_M | 27 |
| HOLLEMAN_PREDNISOLONE_RESISTANCE_B_ALL_UP | HOLLEMAN_PREDNISO | 20 |
| ZHAN_MULTIPLE_MYELOMA_CD1_UP | ZHAN_MULTIPLE_MYE | 43 |
| PID_LYSOPHOSPHOLIPID_PATHWAY | PID_LYSOPHOSPHOLIP | 65 |
| GAVIN_PDE3B_TARGETS | GAVIN_PDE3B_TARGE | 20 |
| BIOCARTA_TOLL_PATHWAY | BIOCARTA_TOLL_PATH | 36 |
| BARRIER_CANCER_RELAPSE_NORMAL_SAMPLE_UP | BARRIER_CANCER_REL | 29 |
| PID_FAS_PATHWAY | PID_FAS_PATHWAY | 36 |
| LINSLEY_MIR16_TARGETS | LINSLEY_MIR16_TARG | 191 |
| PID_CDC42_PATHWAY | PID_CDC42_PATHWAY | 67 |
| NATSUME_RESPONSE_TO_INTERFERON_BETA_DN | NATSUME_RESPONSE_ | 48 |
| REACTOME_SIGNALING_BY_INSULIN_RECEPTOR | REACTOME_SIGNALIN | 101 |
| HASLINGER_B_CLL_WITH_MUTATED_VH_GENES | HASLINGER_B_CLL_WI | 16 |
| GINESTIER_BREAST_CANCER_20Q13_AMPLIFICATION_DN | GINESTIER_BREAST_CA | 147 |
| KEGG_NON_SMALL_CELL_LUNG_CANCER | KEGG_NON_SMALL_CI | 52 |
| HELLER_SILENCED_BY_METHYLATION_DN | HELLER_SILENCED_BY_ | 96 |
| MYLLYKANGAS_AMPLIFICATION_HOT_SPOT_17 | MYLLYKANGAS_AMPLI | 17 |

| | | |
|---|---|---|
| SEIDEN_MET_SIGNALING | SEIDEN_MET_SIGNALI | 18 |
| ZHU_CMV_24_HR_UP | ZHU_CMV_24_HR_UP | 87 |
| WONG_PROTEASOME_GENE_MODULE | WONG_PROTEASOME_ | 47 |
| MCLACHLAN_DENTAL_CARIES_UP | MCLACHLAN_DENTAL_ | 202 |
| AMUNDSON_GAMMA_RADIATION_RESPONSE | AMUNDSON_GAMMA_ | 39 |
| REACTOME_APOPTOSIS | REACTOME_APOPTOSI | 137 |
| REACTOME_SCFSKP2_MEDIATED_DEGRADATION_OF_P27_P21 | REACTOME_SCFSKP2_ | 52 |
| HEIDENBLAD_AMPLICON_12P11_12_UP | HEIDENBLAD_AMPLIC | 32 |
| BIOCARTA_GATA3_PATHWAY | BIOCARTA_GATA3_PA | 15 |
| REACTOME_DOWNSTREAM_TCR_SIGNALING | REACTOME_DOWNST | 27 |
| SHARMA_PILOCYTIC_ASTROCYTOMA_LOCATION_UP | SHARMA_PILOCYTIC_ | 22 |
| LIU_CMYB_TARGETS_UP | LIU_CMYB_TARGETS_ | 143 |
| PID_SMAD2_3NUCLEAR_PATHWAY | PID_SMAD2_3NUCLEA | 81 |
| YE_METASTATIC_LIVER_CANCER | YE_METASTATIC_LIVE | 21 |
| RHODES_UNDIFFERENTIATED_CANCER | RHODES_UNDIFFEREN | 66 |
| KIM_RESPONSE_TO_TSA_AND_DECITABINE_UP | KIM_RESPONSE_TO_T | 117 |
| ZHANG_RESPONSE_TO_IKK_INHIBITOR_AND_TNF_UP | ZHANG_RESPONSE_TC | 199 |
| REACTOME_REGULATION_OF_SIGNALING_BY_CBL | REACTOME_REGULATI | 18 |
| REACTOME_PI3K_CASCADE | REACTOME_PI3K_CAS | 66 |
| KYNG_WERNER_SYNDROM_AND_NORMAL_AGING_UP | KYNG_WERNER_SYND | 84 |
| ZHENG_GLIOBLASTOMA_PLASTICITY_UP | ZHENG_GLIOBLASTOM | 234 |
| BIOCARTA_CARM_ER_PATHWAY | BIOCARTA_CARM_ER_ | 34 |
| DARWICHE_SKIN_TUMOR_PROMOTER_UP | DARWICHE_SKIN_TUM | 125 |
| GROSS_HYPOXIA_VIA_ELK3_DN | GROSS_HYPOXIA_VIA_ | 148 |
| REACTOME_IMMUNOREGULATORY_INTERACTIONS_BETWEEN_A | REACTOME_IMMUNO | 44 |
| HILLION_HMGA1B_TARGETS | HILLION_HMGA1B_TA | 88 |
| BIOCARTA_GSK3_PATHWAY | BIOCARTA_GSK3_PATH | 27 |
| OKAMOTO_LIVER_CANCER_MULTICENTRIC_OCCURRENCE_UP | OKAMOTO_LIVER_CAN | 22 |
| HOFFMANN_LARGE_TO_SMALL_PRE_BII_LYMPHOCYTE_UP | HOFFMANN_LARGE_T | 153 |
| REACTOME_AQUAPORIN_MEDIATED_TRANSPORT | REACTOME_AQUAPOR | 48 |
| WANG_BARRETTS_ESOPHAGUS_UP | WANG_BARRETTS_ESC | 44 |
| BURTON_ADIPOGENESIS_PEAK_AT_24HR | BURTON_ADIPOGENES | 41 |
| ROME_INSULIN_TARGETS_IN_MUSCLE_DN | ROME_INSULIN_TARG | 158 |
| BROWNE_HCMV_INFECTION_8HR_DN | BROWNE_HCMV_INFE | 43 |
| MATTIOLI_MGUS_VS_PCL | MATTIOLI_MGUS_VS_ | 95 |
| REACTOME_CDT1_ASSOCIATION_WITH_THE_CDC6_ORC_ORIGIN_ | REACTOME_CDT1_ASS | 54 |
| KEGG_CYSTEINE_AND_METHIONINE_METABOLISM | KEGG_CYSTEINE_AND_ | 33 |
| DAIRKEE_CANCER_PRONE_RESPONSE_E2 | DAIRKEE_CANCER_PRC | 28 |
| CHEMELLO_SOLEUS_VS_EDL_MYOFIBERS_UP | CHEMELLO_SOLEUS_V | 34 |
| REACTOME_ION_TRANSPORT_BY_P_TYPE_ATPASES | REACTOME_ION_TRAN | 33 |
| BANDRES_RESPONSE_TO_CARMUSTIN_WITHOUT_MGMT_48HR_ | BANDRES_RESPONSE_ | 29 |
| REACTOME_REGULATORY_RNA_PATHWAYS | REACTOME_REGULATC | 25 |
| WEST_ADRENOCORTICAL_CARCINOMA_VS_ADENOMA_UP | WEST_ADRENOCORTIC | 19 |
| REACTOME_SYNTHESIS_OF_PIPS_AT_THE_PLASMA_MEMBRANE | REACTOME_SYNTHESIS | 30 |
| SASSON_RESPONSE_TO_FORSKOLIN_UP | SASSON_RESPONSE_T | 86 |
| LINDSTEDT_DENDRITIC_CELL_MATURATION_A | LINDSTEDT_DENDRITIC | 63 |
| PURBEY_TARGETS_OF_CTBP1_AND_SATB1_DN | PURBEY_TARGETS_OF_ | 166 |
| TAKEDA_TARGETS_OF_NUP98_HOXA9_FUSION_3D_DN | TAKEDA_TARGETS_OF_ | 27 |
| REACTOME_REGULATION_OF_KIT_SIGNALING | REACTOME_REGULATI | 15 |
| HUPER_BREAST_BASAL_VS_LUMINAL_UP | HUPER_BREAST_BASA | 45 |

| | | |
|---|---|---|
| REACTOME_DARPP_32_EVENTS | REACTOME_DARPP_32 | 23 |
| REACTOME_REGULATION_OF_BETA_CELL_DEVELOPMENT | REACTOME_REGULATI | 29 |
| KEGG_GLYCOSYLPHOSPHATIDYLINOSITOL_GPI_ANCHOR_BIOSYNT | KEGG_GLYCOSYLPHOS | 24 |
| NADLER_HYPERGLYCEMIA_AT_OBESITY | NADLER_HYPERGLYCEI | 57 |
| NAM_FXYD5_TARGETS_DN | NAM_FXYD5_TARGETS | 18 |
| KIM_ALL_DISORDERS_OLIGODENDROCYTE_NUMBER_CORR_DN | KIM_ALL_DISORDERS_ | 28 |
| REACTOME_APOPTOTIC_CLEAVAGE_OF_CELLULAR_PROTEINS | REACTOME_APOPTOT | 35 |
| WORSCHECH_TUMOR_EVASION_AND_TOLEROGENICITY_UP | WORSCHECH_TUMOR_ | 30 |
| REACTOME_PROCESSING_OF_CAPPED_INTRONLESS_PRE_MRNA | REACTOME_PROCESSII | 22 |
| BROWNE_HCMV_INFECTION_8HR_UP | BROWNE_HCMV_INFE | 90 |
| VANTVEER_BREAST_CANCER_ESR1_DN | VANTVEER_BREAST_C/ | 219 |
| WAMUNYOKOLI_OVARIAN_CANCER_GRADES_1_2_UP | WAMUNYOKOLI_OVAI | 125 |
| WINZEN_DEGRADED_VIA_KHSRP | WINZEN_DEGRADED_\ | 97 |
| REACTOME_ACYL_CHAIN_REMODELLING_OF_PI | REACTOME_ACYL_CH/ | 15 |
| KANG_FLUOROURACIL_RESISTANCE_UP | KANG_FLUOROURACIL | 21 |
| REACTOME_NRAGE_SIGNALS_DEATH_THROUGH_JNK | REACTOME_NRAGE_SI | 43 |
| REACTOME_INTERACTION_BETWEEN_L1_AND_ANKYRINS | REACTOME_INTERACT | 19 |
| KEGG_PRIMARY_BILE_ACID_BIOSYNTHESIS | KEGG_PRIMARY_BILE_ | 15 |
| REACTOME_RNA_POL_III_TRANSCRIPTION_INITIATION_FROM_TY | REACTOME_RNA_POL_ | 23 |
| PODAR_RESPONSE_TO_ADAPHOSTIN_UP | PODAR_RESPONSE_TC | 131 |
| LIANG_SILENCED_BY_METHYLATION_2 | LIANG_SILENCED_BY_I | 47 |
| MAEKAWA_ATF2_TARGETS | MAEKAWA_ATF2_TAR | 24 |
| BORLAK_LIVER_CANCER_EGF_UP | BORLAK_LIVER_CANCE | 53 |
| RAO_BOUND_BY_SALL4 | RAO_BOUND_BY_SALL | 203 |
| RODRIGUES_NTN1_TARGETS_DN | RODRIGUES_NTN1_TA | 143 |
| REACTOME_PROTEIN_FOLDING | REACTOME_PROTEIN_ | 48 |
| DELASERNA_MYOD_TARGETS_UP | DELASERNA_MYOD_T/ | 85 |
| KIM_MYCN_AMPLIFICATION_TARGETS_UP | KIM_MYCN_AMPLIFIC/ | 85 |
| HANN_RESISTANCE_TO_BCL2_INHIBITOR_UP | HANN_RESISTANCE_T( | 28 |
| MIKKELSEN_MEF_ICP_WITH_H3K27ME3 | MIKKELSEN_MEF_ICP_ | 182 |
| MACLACHLAN_BRCA1_TARGETS_UP | MACLACHLAN_BRCA1_ | 21 |
| MOOTHA_VOXPHOS | MOOTHA_VOXPHOS | 83 |
| KUMAR_PATHOGEN_LOAD_BY_MACROPHAGES | KUMAR_PATHOGEN_L | 216 |
| ANDERSEN_CHOLANGIOCARCINOMA_CLASS1 | ANDERSEN_CHOLANGI | 53 |
| LEE_LIVER_CANCER_SURVIVAL_DN | LEE_LIVER_CANCER_SU | 162 |
| GROSS_HYPOXIA_VIA_ELK3_AND_HIF1A_DN | GROSS_HYPOXIA_VIA_ | 101 |
| REACTOME_FORMATION_OF_TUBULIN_FOLDING_INTERMEDIATE | REACTOME_FORMATI( | 19 |
| PARK_HSC_VS_MULTIPOTENT_PROGENITORS_DN | PARK_HSC_VS_MULTII | 18 |
| REACTOME_ACTIVATION_OF_NF_KAPPAB_IN_B_CELLS | REACTOME_ACTIVATI( | 60 |
| REACTOME_SIGNALING_BY_FGFR1_MUTANTS | REACTOME_SIGNALIN( | 28 |
| BOYAULT_LIVER_CANCER_SUBCLASS_G123_DN | BOYAULT_LIVER_CAN( | 49 |
| PID_IL2_PI3K_PATHWAY | PID_IL2_PI3K_PATHW/ | 34 |
| CAFFAREL_RESPONSE_TO_THC_DN | CAFFAREL_RESPONSE_ | 26 |
| GARY_CD5_TARGETS_DN | GARY_CD5_TARGETS_ | 404 |
| KYNG_DNA_DAMAGE_BY_4NQO | KYNG_DNA_DAMAGE_ | 31 |
| GROSS_HYPOXIA_VIA_HIF1A_UP | GROSS_HYPOXIA_VIA_ | 76 |
| BYSTROEM_CORRELATED_WITH_IL5_DN | BYSTROEM_CORRELAT | 60 |
| BASSO_CD40_SIGNALING_UP | BASSO_CD40_SIGNALI | 92 |
| ZHAN_V2_LATE_DIFFERENTIATION_GENES | ZHAN_V2_LATE_DIFFE | 41 |
| WU_HBX_TARGETS_3_UP | WU_HBX_TARGETS_3_ | 18 |

| | | |
|---|---|---|
| RODRIGUES_THYROID_CARCINOMA_DN | RODRIGUES_THYROID_ | 66 |
| JEPSEN_SMRT_TARGETS | JEPSEN_SMRT_TARGET | 32 |
| YAO_TEMPORAL_RESPONSE_TO_PROGESTERONE_CLUSTER_2 | YAO_TEMPORAL_RESP | 79 |
| REACTOME_RNA_POL_III_CHAIN_ELONGATION | REACTOME_RNA_POL_ | 17 |
| PRAMOONJAGO_SOX4_TARGETS_UP | PRAMOONJAGO_SOX4 | 51 |
| PYEON_CANCER_HEAD_AND_NECK_VS_CERVICAL_DN | PYEON_CANCER_HEAD | 23 |
| NICK_RESPONSE_TO_PROC_TREATMENT_DN | NICK_RESPONSE_TO_P | 22 |
| ZHONG_SECRETOME_OF_LUNG_CANCER_AND_MACROPHAGE | ZHONG_SECRETOME_ | 76 |
| JAEGER_METASTASIS_DN | JAEGER_METASTASIS_ | 229 |
| ANDERSEN_CHOLANGIOCARCINOMA_CLASS2 | ANDERSEN_CHOLANGI | 156 |
| REACTOME_GLUCOSE_TRANSPORT | REACTOME_GLUCOSE_ | 38 |
| BIOCARTA_TALL1_PATHWAY | BIOCARTA_TALL1_PAT | 15 |
| KEGG_PURINE_METABOLISM | KEGG_PURINE_METAB | 153 |
| BIOCARTA_P53_PATHWAY | BIOCARTA_P53_PATHV | 15 |
| DAIRKEE_TERT_TARGETS_DN | DAIRKEE_TERT_TARGE | 103 |
| PID_REG_GR_PATHWAY | PID_REG_GR_PATHWA | 78 |
| BRUINS_UVC_RESPONSE_VIA_TP53_GROUP_D | BRUINS_UVC_RESPON | 252 |
| LEIN_CEREBELLUM_MARKERS | LEIN_CEREBELLUM_M | 78 |
| REACTOME_REGULATION_OF_ORNITHINE_DECARBOXYLASE_ODC | REACTOME_REGULATI | 48 |
| DACOSTA_LOW_DOSE_UV_RESPONSE_VIA_ERCC3_XPCS_UP | DACOSTA_LOW_DOSE_ | 16 |
| LI_WILMS_TUMOR_VS_FETAL_KIDNEY_2_UP | LI_WILMS_TUMOR_VS | 30 |
| BOYAULT_LIVER_CANCER_SUBCLASS_G123_UP | BOYAULT_LIVER_CANC | 43 |
| REACTOME_TCR_SIGNALING | REACTOME_TCR_SIGN | 44 |
| GRAHAM_CML_QUIESCENT_VS_NORMAL_DIVIDING_UP | GRAHAM_CML_QUIES | 50 |
| REACTOME_ACTIVATION_OF_BH3_ONLY_PROTEINS | REACTOME_ACTIVATIO | 15 |
| BORCZUK_MALIGNANT_MESOTHELIOMA_UP | BORCZUK_MALIGNANT | 284 |
| NUMATA_CSF3_SIGNALING_VIA_STAT3 | NUMATA_CSF3_SIGNA | 21 |
| PENG_GLUTAMINE_DEPRIVATION_DN | PENG_GLUTAMINE_DE | 320 |
| REACTOME_CYCLIN_E_ASSOCIATED_EVENTS_DURING_G1_S_TRA | REACTOME_CYCLIN_E_ | 61 |
| BLUM_RESPONSE_TO_SALIRASIB_DN | BLUM_RESPONSE_TO_ | 328 |
| VERNELL_RETINOBLASTOMA_PATHWAY_DN | VERNELL_RETINOBLAS | 18 |
| RHEIN_ALL_GLUCOCORTICOID_THERAPY_DN | RHEIN_ALL_GLUCOCO | 344 |
| IVANOVA_HEMATOPOIESIS_MATURE_CELL | IVANOVA_HEMATOPO | 261 |
| DORN_ADENOVIRUS_INFECTION_12HR_UP | DORN_ADENOVIRUS_I | 28 |
| BIOCARTA_TNFR1_PATHWAY | BIOCARTA_TNFR1_PAT | 29 |
| SIG_PIP3_SIGNALING_IN_B_LYMPHOCYTES | SIG_PIP3_SIGNALING_ | 35 |
| RAHMAN_TP53_TARGETS_PHOSPHORYLATED | RAHMAN_TP53_TARG | 21 |
| GAZIN_EPIGENETIC_SILENCING_BY_KRAS | GAZIN_EPIGENETIC_SI | 24 |
| REACTOME_HOST_INTERACTIONS_OF_HIV_FACTORS | REACTOME_HOST_INT | 115 |
| KEGG_T_CELL_RECEPTOR_SIGNALING_PATHWAY | KEGG_T_CELL_RECEPT | 105 |
| GUTIERREZ_CHRONIC_LYMPHOCYTIC_LEUKEMIA_DN | GUTIERREZ_CHRONIC_ | 51 |
| REACTOME_CYCLIN_A_B1_ASSOCIATED_EVENTS_DURING_G2_M | REACTOME_CYCLIN_A | 15 |
| MURAKAMI_UV_RESPONSE_6HR_DN | MURAKAMI_UV_RESP | 20 |
| MIKKELSEN_MCV6_ICP_WITH_H3K27ME3 | MIKKELSEN_MCV6_ICP | 67 |
| WANG_NFKB_TARGETS | WANG_NFKB_TARGET | 24 |
| PID_FRA_PATHWAY | PID_FRA_PATHWAY | 36 |
| PID_PI3K_PLC_TRK_PATHWAY | PID_PI3K_PLC_TRK_PA | 34 |
| GARY_CD5_TARGETS_UP | GARY_CD5_TARGETS_ | 431 |
| VALK_AML_CLUSTER_8 | VALK_AML_CLUSTER_ | 23 |
| ACEVEDO_LIVER_CANCER_WITH_H3K9ME3_DN | ACEVEDO_LIVER_CANC | 90 |

| | | |
|---|---|---|
| NELSON_RESPONSE_TO_ANDROGEN_DN | NELSON_RESPONSE_T( | 19 |
| PID_RAS_PATHWAY | PID_RAS_PATHWAY | 28 |
| LI_LUNG_CANCER | LI_LUNG_CANCER | 40 |
| REACTOME_ELONGATION_ARREST_AND_RECOVERY | REACTOME_ELONGATI | 24 |
| HOELZEL_NF1_TARGETS_UP | HOELZEL_NF1_TARGET | 128 |
| BASAKI_YBX1_TARGETS_UP | BASAKI_YBX1_TARGET | 261 |
| WANG_RECURRENT_LIVER_CANCER_DN | WANG_RECURRENT_L | 16 |
| DANG_MYC_TARGETS_UP | DANG_MYC_TARGETS_ | 138 |
| MIKKELSEN_MEF_ICP_WITH_H3K4ME3_AND_H3K27ME3 | MIKKELSEN_MEF_ICP_ | 38 |
| LABBE_TGFB1_TARGETS_DN | LABBE_TGFB1_TARGET | 95 |
| REACTOME_ION_CHANNEL_TRANSPORT | REACTOME_ION_CHAI | 53 |
| BIOCARTA_RACCYCD_PATHWAY | BIOCARTA_RACCYCD_I | 25 |
| SHEN_SMARCA2_TARGETS_DN | SHEN_SMARCA2_TAR( | 273 |
| REACTOME_REGULATION_OF_APOPTOSIS | REACTOME_REGULATI | 56 |
| HINATA_NFKB_TARGETS_KERATINOCYTE_DN | HINATA_NFKB_TARGE | 20 |
| LIN_NPAS4_TARGETS_UP | LIN_NPAS4_TARGETS_ | 143 |
| REACTOME_RNA_POL_III_TRANSCRIPTION_TERMINATION | REACTOME_RNA_POL_ | 19 |
| JIANG_AGING_HYPOTHALAMUS_DN | JIANG_AGING_HYPOTI | 40 |
| LIU_BREAST_CANCER | LIU_BREAST_CANCER | 23 |
| PID_PI3KCI_PATHWAY | PID_PI3KCI_PATHWAY | 48 |
| BROWNE_HCMV_INFECTION_6HR_UP | BROWNE_HCMV_INFE | 62 |
| PID_MET_PATHWAY | PID_MET_PATHWAY | 79 |
| AMIT_EGF_RESPONSE_240_MCF10A | AMIT_EGF_RESPONSE_ | 19 |
| SHIPP_DLBCL_VS_FOLLICULAR_LYMPHOMA_UP | SHIPP_DLBCL_VS_FOLI | 43 |
| KEGG_B_CELL_RECEPTOR_SIGNALING_PATHWAY | KEGG_B_CELL_RECEPT | 73 |
| GRAHAM_CML_QUIESCENT_VS_NORMAL_QUIESCENT_DN | GRAHAM_CML_QUIES | 38 |
| BOYAULT_LIVER_CANCER_SUBCLASS_G1_DN | BOYAULT_LIVER_CAN( | 33 |
| LIU_VAV3_PROSTATE_CARCINOGENESIS_DN | LIU_VAV3_PROSTATE_ | 16 |
| PID_HEDGEHOG_GLI_PATHWAY | PID_HEDGEHOG_GLI_I | 48 |
| ZAMORA_NOS2_TARGETS_UP | ZAMORA_NOS2_TARG | 64 |
| KEGG_TASTE_TRANSDUCTION | KEGG_TASTE_TRANSD | 26 |
| REACTOME_DESTABILIZATION_OF_MRNA_BY_AUF1_HNRNP_D0 | REACTOME_DESTABILI | 50 |
| TARTE_PLASMA_CELL_VS_PLASMABLAST_DN | TARTE_PLASMA_CELL_ | 298 |
| JAIN_NFKB_SIGNALING | JAIN_NFKB_SIGNALIN( | 70 |
| PID_AMB2_NEUTROPHILS_PATHWAY | PID_AMB2_NEUTROPI | 40 |
| BOUDOUKHA_BOUND_BY_IGF2BP2 | BOUDOUKHA_BOUND_ | 101 |
| LAIHO_COLORECTAL_CANCER_SERRATED_DN | LAIHO_COLORECTAL_( | 72 |
| FERRARI_RESPONSE_TO_FENRETINIDE_UP | FERRARI_RESPONSE_T | 21 |
| MOREAUX_B_LYMPHOCYTE_MATURATION_BY_TACI_DN | MOREAUX_B_LYMPHO | 63 |
| CROONQUIST_NRAS_SIGNALING_UP | CROONQUIST_NRAS_S | 36 |
| KYNG_ENVIRONMENTAL_STRESS_RESPONSE_DN | KYNG_ENVIRONMENT, | 18 |
| MURAKAMI_UV_RESPONSE_6HR_UP | MURAKAMI_UV_RESP( | 32 |
| DACOSTA_UV_RESPONSE_VIA_ERCC3_XPCS_UP | DACOSTA_UV_RESPON | 26 |
| LIU_SOX4_TARGETS_UP | LIU_SOX4_TARGETS_U | 120 |
| VILLANUEVA_LIVER_CANCER_KRT19_DN | VILLANUEVA_LIVER_C/ | 55 |
| ZHAN_MULTIPLE_MYELOMA_CD1_VS_CD2_UP | ZHAN_MULTIPLE_MYE | 63 |
| GRAHAM_NORMAL_QUIESCENT_VS_NORMAL_DIVIDING_UP | GRAHAM_NORMAL_Q | 64 |
| LY_AGING_MIDDLE_DN | LY_AGING_MIDDLE_DI | 16 |
| ST_GA13_PATHWAY | ST_GA13_PATHWAY | 34 |
| GUENTHER_GROWTH_SPHERICAL_VS_ADHERENT_UP | GUENTHER_GROWTH_ | 21 |

| | | |
|---|---|---|
| REACTOME_REGULATION_OF_WATER_BALANCE_BY_RENAL_AQU | REACTOME_REGULATI | 42 |
| TRAYNOR_RETT_SYNDROM_DN | TRAYNOR_RETT_SYND | 17 |
| KEGG_RIBOFLAVIN_METABOLISM | KEGG_RIBOFLAVIN_MI | 16 |
| JAZAERI_BREAST_CANCER_BRCA1_VS_BRCA2_UP | JAZAERI_BREAST_CAN | 48 |
| YU_BAP1_TARGETS | YU_BAP1_TARGETS | 27 |
| PAL_PRMT5_TARGETS_DN | PAL_PRMT5_TARGETS_ | 29 |
| HU_GENOTOXIC_DAMAGE_4HR | HU_GENOTOXIC_DAM | 35 |
| HUMMERICH_BENIGN_SKIN_TUMOR_UP | HUMMERICH_BENIGN_ | 16 |
| GALE_APL_WITH_FLT3_MUTATED_UP | GALE_APL_WITH_FLT3 | 49 |
| TOMIDA_METASTASIS_DN | TOMIDA_METASTASIS_ | 17 |
| REACTOME_THROMBIN_SIGNALLING_THROUGH_PROTEINASE_AC | REACTOME_THROMBI | 32 |
| LIM_MAMMARY_STEM_CELL_DN | LIM_MAMMARY_STEM | 394 |
| TONKS_TARGETS_OF_RUNX1_RUNX1T1_FUSION_SUSTAINDED_IN | TONKS_TARGETS_OF_ | 41 |
| GROSS_HYPOXIA_VIA_ELK3_ONLY_DN | GROSS_HYPOXIA_VIA_ | 40 |
| SHIPP_DLBCL_VS_FOLLICULAR_LYMPHOMA_DN | SHIPP_DLBCL_VS_FOLL | 41 |
| LASTOWSKA_NEUROBLASTOMA_COPY_NUMBER_UP | LASTOWSKA_NEUROB | 165 |
| REACTOME_GLUCONEOGENESIS | REACTOME_GLUCONE | 28 |
| PID_CD8_TCR_PATHWAY | PID_CD8_TCR_PATHW | 50 |
| LEE_AGING_MUSCLE_UP | LEE_AGING_MUSCLE_ | 42 |
| BIOCARTA_CK1_PATHWAY | BIOCARTA_CK1_PATH | 15 |
| VALK_AML_CLUSTER_1 | VALK_AML_CLUSTER_ | 27 |
| KEGG_SPLICEOSOME | KEGG_SPLICEOSOME | 121 |
| AMIT_EGF_RESPONSE_40_HELA | AMIT_EGF_RESPONSE_ | 42 |
| MOREAUX_MULTIPLE_MYELOMA_BY_TACI_DN | MOREAUX_MULTIPLE_ | 150 |
| KEGG_PARKINSONS_DISEASE | KEGG_PARKINSONS_D | 109 |
| BIOCARTA_G1_PATHWAY | BIOCARTA_G1_PATHW | 27 |
| RASHI_RESPONSE_TO_IONIZING_RADIATION_6 | RASHI_RESPONSE_TO_ | 76 |
| HERNANDEZ_ABERRANT_MITOSIS_BY_DOCETACEL_2NM_DN | HERNANDEZ_ABERRAN | 23 |
| TORCHIA_TARGETS_OF_EWSR1_FLI1_FUSION_TOP20_UP | TORCHIA_TARGETS_OI | 17 |
| SARTIPY_BLUNTED_BY_INSULIN_RESISTANCE_UP | SARTIPY_BLUNTED_BY | 18 |
| REACTOME_MRNA_SPLICING_MINOR_PATHWAY | REACTOME_MRNA_SP | 41 |
| WANG_BARRETTS_ESOPHAGUS_DN | WANG_BARRETTS_ESC | 23 |
| WEIGEL_OXIDATIVE_STRESS_BY_HNE_AND_H2O2 | WEIGEL_OXIDATIVE_S | 39 |
| PID_AURORA_B_PATHWAY | PID_AURORA_B_PATH | 38 |
| MORI_EMU_MYC_LYMPHOMA_BY_ONSET_TIME_DN | MORI_EMU_MYC_LYM | 16 |
| THILLAINADESAN_ZNF217_TARGETS_UP | THILLAINADESAN_ZNF | 42 |
| GRANDVAUX_IRF3_TARGETS_UP | GRANDVAUX_IRF3_TA | 15 |
| TONG_INTERACT_WITH_PTTG1 | TONG_INTERACT_WIT | 49 |
| GAUSSMANN_MLL_AF4_FUSION_TARGETS_C_DN | GAUSSMANN_MLL_AF | 19 |
| GAZDA_DIAMOND_BLACKFAN_ANEMIA_PROGENITOR_DN | GAZDA_DIAMOND_BL | 60 |
| SAKAI_TUMOR_INFILTRATING_MONOCYTES_DN | SAKAI_TUMOR_INFILT | 73 |
| BIOCARTA_PROTEASOME_PATHWAY | BIOCARTA_PROTEASO | 28 |
| ONO_AML1_TARGETS_DN | ONO_AML1_TARGETS_ | 39 |
| REACTOME_CLEAVAGE_OF_GROWING_TRANSCRIPT_IN_THE_TER | REACTOME_CLEAVAGI | 41 |
| REACTOME_PI3K_AKT_ACTIVATION | REACTOME_PI3K_AKT_ | 36 |
| DAZARD_UV_RESPONSE_CLUSTER_G28 | DAZARD_UV_RESPONS | 18 |
| KEGG_AMYOTROPHIC_LATERAL_SCLEROSIS_ALS | KEGG_AMYOTROPHIC_ | 51 |
| AFFAR_YY1_TARGETS_DN | AFFAR_YY1_TARGETS_ | 218 |
| MASRI_RESISTANCE_TO_TAMOXIFEN_AND_AROMATASE_INHIBIT | MASRI_RESISTANCE_T | 19 |
| PID_RB_1PATHWAY | PID_RB_1PATHWAY | 63 |

| | | |
|---|---|---|
| CUI_GLUCOSE_DEPRIVATION | CUI_GLUCOSE_DEPRIV | 58 |
| PODAR_RESPONSE_TO_ADAPHOSTIN_DN | PODAR_RESPONSE_TC | 15 |
| BOSCO_ALLERGEN_INDUCED_TH2_ASSOCIATED_MODULE | BOSCO_ALLERGEN_INI | 137 |
| ONDER_CDH1_TARGETS_1_DN | ONDER_CDH1_TARGET | 154 |
| HANSON_HRAS_SIGNALING_VIA_NFKB | HANSON_HRAS_SIGNA | 21 |
| KAYO_AGING_MUSCLE_DN | KAYO_AGING_MUSCLE | 113 |
| SIG_INSULIN_RECEPTOR_PATHWAY_IN_CARDIAC_MYOCYTES | SIG_INSULIN_RECEPTC | 50 |
| BAKER_HEMATOPOIESIS_STAT3_TARGETS | BAKER_HEMATOPOIES | 16 |
| HOWLIN_CITED1_TARGETS_1_DN | HOWLIN_CITED1_TAR( | 33 |
| REACTOME_RNA_POL_I_TRANSCRIPTION_TERMINATION | REACTOME_RNA_POL_ | 20 |
| PACHER_TARGETS_OF_IGF1_AND_IGF2_UP | PACHER_TARGETS_OF_ | 35 |
| BILD_MYC_ONCOGENIC_SIGNATURE | BILD_MYC_ONCOGENI | 179 |
| OUILLETTE_CLL_13Q14_DELETION_DN | OUILLETTE_CLL_13Q14 | 53 |
| CHOI_ATL_CHRONIC_VS_ACUTE_DN | CHOI_ATL_CHRONIC_V | 18 |
| SHAFFER_IRF4_TARGETS_IN_MYELOMA_VS_MATURE_B_LYMPHC | SHAFFER_IRF4_TARGET | 98 |
| LIN_APC_TARGETS | LIN_APC_TARGETS | 75 |
| KEGG_OXIDATIVE_PHOSPHORYLATION | KEGG_OXIDATIVE_PHC | 112 |
| KRIGE_AMINO_ACID_DEPRIVATION | KRIGE_AMINO_ACID_I | 29 |
| CHO_NR4A1_TARGETS | CHO_NR4A1_TARGETS | 30 |
| NIKOLSKY_BREAST_CANCER_17Q11_Q21_AMPLICON | NIKOLSKY_BREAST_CA | 110 |
| DAUER_STAT3_TARGETS_UP | DAUER_STAT3_TARGE | 45 |
| STARK_PREFRONTAL_CORTEX_22Q11_DELETION_DN | STARK_PREFRONTAL_( | 459 |
| REACTOME_MRNA_PROCESSING | REACTOME_MRNA_PF | 151 |
| REACTOME_PROCESSING_OF_CAPPED_INTRON_CONTAINING_PR | REACTOME_PROCESSI | 134 |
| DOANE_BREAST_CANCER_ESR1_UP | DOANE_BREAST_CANC | 99 |
| BIOCARTA_TID_PATHWAY | BIOCARTA_TID_PATHV | 18 |
| WEST_ADRENOCORTICAL_TUMOR_UP | WEST_ADRENOCORTIC | 276 |
| GOLUB_ALL_VS_AML_DN | GOLUB_ALL_VS_AML_ | 22 |
| HUANG_FOXA2_TARGETS_DN | HUANG_FOXA2_TARG | 35 |
| WILENSKY_RESPONSE_TO_DARAPLADIB | WILENSKY_RESPONSE_ | 28 |
| POMEROY_MEDULLOBLASTOMA_PROGNOSIS_DN | POMEROY_MEDULLOE | 43 |
| KARLSSON_TGFB1_TARGETS_UP | KARLSSON_TGFB1_TAI | 120 |
| HOLLERN_MICROACINAR_BREAST_TUMOR_UP | HOLLERN_MICROACIN | 30 |
| BIOCARTA_TH1TH2_PATHWAY | BIOCARTA_TH1TH2_P/ | 16 |
| NEMETH_INFLAMMATORY_RESPONSE_LPS_DN | NEMETH_INFLAMMAT | 30 |
| ONO_FOXP3_TARGETS_UP | ONO_FOXP3_TARGETS | 23 |
| SENGUPTA_NASOPHARYNGEAL_CARCINOMA_WITH_LMP1_DN | SENGUPTA_NASOPHAI | 131 |
| WANG_METASTASIS_OF_BREAST_CANCER_ESR1_DN | WANG_METASTASIS_( | 25 |
| ST_INTERLEUKIN_4_PATHWAY | ST_INTERLEUKIN_4_P/ | 23 |
| PID_IL12_2PATHWAY | PID_IL12_2PATHWAY | 57 |
| MAGRANGEAS_MULTIPLE_MYELOMA_IGLL_VS_IGLK_UP | MAGRANGEAS_MULTI | 39 |
| PRAMOONJAGO_SOX4_TARGETS_DN | PRAMOONJAGO_SOX4 | 49 |
| FOSTER_KDM1A_TARGETS_UP | FOSTER_KDM1A_TAR( | 230 |
| NIELSEN_SCHWANNOMA_UP | NIELSEN_SCHWANNOI | 16 |
| REACTOME_DESTABILIZATION_OF_MRNA_BY_BRF1 | REACTOME_DESTABILI | 17 |
| SCHEIDEREIT_IKK_TARGETS | SCHEIDEREIT_IKK_TAR | 17 |
| ZHU_CMV_ALL_UP | ZHU_CMV_ALL_UP | 113 |
| REACTOME_MYOGENESIS | REACTOME_MYOGENE | 26 |
| REACTOME_P53_INDEPENDENT_G1_S_DNA_DAMAGE_CHECKPOI | REACTOME_P53_INDE | 48 |
| JAERVINEN_AMPLIFIED_IN_LARYNGEAL_CANCER | JAERVINEN_AMPLIFIEI | 35 |

| | | |
|---|---|---|
| JIANG_HYPOXIA_VIA_VHL | JIANG_HYPOXIA_VIA_V | 32 |
| KEGG_PRION_DISEASES | KEGG_PRION_DISEASE | 33 |
| REACTOME_AUTODEGRADATION_OF_THE_E3_UBIQUITIN_LIGASE | REACTOME_AUTODEG | 46 |
| PENG_LEUCINE_DEPRIVATION_DN | PENG_LEUCINE_DEPRI | 179 |
| GUTIERREZ_MULTIPLE_MYELOMA_UP | GUTIERREZ_MULTIPLE | 30 |
| PID_CIRCADIAN_PATHWAY | PID_CIRCADIAN_PATH | 15 |
| PID_BCR_5PATHWAY | PID_BCR_5PATHWAY | 63 |
| VANHARANTA_UTERINE_FIBROID_WITH_7Q_DELETION_UP | VANHARANTA_UTERIN | 65 |
| SIMBULAN_PARP1_TARGETS_DN | SIMBULAN_PARP1_TA | 17 |
| PID_INTEGRIN2_PATHWAY | PID_INTEGRIN2_PATH | 27 |
| NAKAJIMA_EOSINOPHIL | NAKAJIMA_EOSINOPH | 27 |
| ZHAN_V1_LATE_DIFFERENTIATION_GENES_DN | ZHAN_V1_LATE_DIFFE | 15 |
| HUPER_BREAST_BASAL_VS_LUMINAL_DN | HUPER_BREAST_BASA | 53 |
| ROVERSI_GLIOMA_LOH_REGIONS | ROVERSI_GLIOMA_LO | 43 |
| TAKAO_RESPONSE_TO_UVB_RADIATION_UP | TAKAO_RESPONSE_TO | 82 |
| ZHAN_MULTIPLE_MYELOMA_HP_UP | ZHAN_MULTIPLE_MYE | 42 |
| MEINHOLD_OVARIAN_CANCER_LOW_GRADE_DN | MEINHOLD_OVARIAN_ | 20 |
| AMUNDSON_DNA_DAMAGE_RESPONSE_TP53 | AMUNDSON_DNA_DA | 15 |
| ABE_VEGFA_TARGETS_2HR | ABE_VEGFA_TARGETS_ | 32 |
| NATSUME_RESPONSE_TO_INTERFERON_BETA_UP | NATSUME_RESPONSE_ | 65 |
| CHOI_ATL_STAGE_PREDICTOR | CHOI_ATL_STAGE_PRE | 35 |
| WANG_LSD1_TARGETS_DN | WANG_LSD1_TARGETS | 38 |
| KRIEG_KDM3A_TARGETS_NOT_HYPOXIA | KRIEG_KDM3A_TARGE | 177 |
| REACTOME_CHEMOKINE_RECEPTORS_BIND_CHEMOKINES | REACTOME_CHEMOKI | 48 |
| SMID_BREAST_CANCER_RELAPSE_IN_LUNG_UP | SMID_BREAST_CANCE | 19 |
| REACTOME_CROSS_PRESENTATION_OF_SOLUBLE_EXOGENOUS_A | REACTOME_CROSS_PF | 45 |
| PID_TCR_PATHWAY | PID_TCR_PATHWAY | 63 |
| AIYAR_COBRA1_TARGETS_UP | AIYAR_COBRA1_TARG | 35 |
| MCCLUNG_COCAIN_REWARD_4WK | MCCLUNG_COCAIN_R | 71 |
| PID_P38_ALPHA_BETA_PATHWAY | PID_P38_ALPHA_BETA | 31 |
| CHANGOLKAR_H2AFY_TARGETS_DN | CHANGOLKAR_H2AFY_ | 38 |
| SAKAI_CHRONIC_HEPATITIS_VS_LIVER_CANCER_UP | SAKAI_CHRONIC_HEPA | 76 |
| BIOCARTA_NO2IL12_PATHWAY | BIOCARTA_NO2IL12_P | 17 |
| AIYAR_COBRA1_TARGETS_DN | AIYAR_COBRA1_TARG | 23 |
| REACTOME_CDK_MEDIATED_PHOSPHORYLATION_AND_REMOVA | REACTOME_CDK_MED | 46 |
| MORI_PRE_BI_LYMPHOCYTE_UP | MORI_PRE_BI_LYMPH | 77 |
| PENG_LEUCINE_DEPRIVATION_UP | PENG_LEUCINE_DEPRI | 129 |
| BIOCARTA_41BB_PATHWAY | BIOCARTA_41BB_PATH | 17 |
| WELCSH_BRCA1_TARGETS_DN | WELCSH_BRCA1_TARG | 138 |
| REACTOME_BOTULINUM_NEUROTOXICITY | REACTOME_BOTULINU | 15 |
| MIKKELSEN_MEF_LCP_WITH_H3K27ME3 | MIKKELSEN_MEF_LCP_ | 59 |
| BIOCARTA_CELLCYCLE_PATHWAY | BIOCARTA_CELLCYCLE_ | 23 |
| GREENBAUM_E2A_TARGETS_UP | GREENBAUM_E2A_TA | 30 |
| REACTOME_SCF_BETA_TRCP_MEDIATED_DEGRADATION_OF_EM | REACTOME_SCF_BETA | 48 |
| MEISSNER_BRAIN_HCP_WITH_H3K4ME2_AND_H3K27ME3 | MEISSNER_BRAIN_HCP | 56 |
| IVANOVA_HEMATOPOIESIS_INTERMEDIATE_PROGENITOR | IVANOVA_HEMATOPO | 134 |
| BIOCARTA_LAIR_PATHWAY | BIOCARTA_LAIR_PATH | 16 |
| KERLEY_RESPONSE_TO_CISPLATIN_UP | KERLEY_RESPONSE_TO | 38 |
| ZEMBUTSU_SENSITIVITY_TO_CYCLOPHOSPHAMIDE | ZEMBUTSU_SENSITIVIT | 15 |
| REACTOME_REGULATION_OF_MRNA_STABILITY_BY_PROTEINS_T | REACTOME_REGULATI | 81 |

| | | |
|---|---|---|
| FARMER_BREAST_CANCER_CLUSTER_5 | FARMER_BREAST_CAN | 17 |
| KEGG_GLYCOSPHINGOLIPID_BIOSYNTHESIS_GANGLIO_SERIES | KEGG_GLYCOSPHINGC | 15 |
| GAL_LEUKEMIC_STEM_CELL_DN | GAL_LEUKEMIC_STEM | 211 |
| SCHLESINGER_H3K27ME3_IN_NORMAL_AND_METHYLATED_IN_( | SCHLESINGER_H3K27N | 27 |
| GAJATE_RESPONSE_TO_TRABECTEDIN_DN | GAJATE_RESPONSE_TC | 19 |
| ZWANG_EGF_PERSISTENTLY_UP | ZWANG_EGF_PERSISTI | 27 |
| WANG_TUMOR_INVASIVENESS_UP | WANG_TUMOR_INVAS | 354 |
| LEIN_NEURON_MARKERS | LEIN_NEURON_MARKI | 65 |
| REACTOME_TCA_CYCLE_AND_RESPIRATORY_ELECTRON_TRANSP( | REACTOME_TCA_CYCL | 112 |
| REACTOME_P53_DEPENDENT_G1_DNA_DAMAGE_RESPONSE | REACTOME_P53_DEPE | 52 |
| MISSIAGLIA_REGULATED_BY_METHYLATION_UP | MISSIAGLIA_REGULATI | 111 |
| TERAMOTO_OPN_TARGETS_CLUSTER_7 | TERAMOTO_OPN_TAR | 17 |
| REACTOME_AUTODEGRADATION_OF_CDH1_BY_CDH1_APC_C | REACTOME_AUTODEG | 56 |
| YUAN_ZNF143_PARTNERS | YUAN_ZNF143_PARTN | 22 |
| KOINUMA_COLON_CANCER_MSI_UP | KOINUMA_COLON_CA | 15 |
| CHEMELLO_SOLEUS_VS_EDL_MYOFIBERS_DN | CHEMELLO_SOLEUS_V | 19 |
| LUI_TARGETS_OF_PAX8_PPARG_FUSION | LUI_TARGETS_OF_PAX | 32 |
| PID_EPO_PATHWAY | PID_EPO_PATHWAY | 33 |
| ACOSTA_PROLIFERATION_INDEPENDENT_MYC_TARGETS_DN | ACOSTA_PROLIFERATI( | 104 |
| REACTOME_VIF_MEDIATED_DEGRADATION_OF_APOBEC3G | REACTOME_VIF_MEDI | 48 |
| RHODES_CANCER_META_SIGNATURE | RHODES_CANCER_ME | 62 |
| IGARASHI_ATF4_TARGETS_DN | IGARASHI_ATF4_TARG | 82 |
| REACTOME_POST_NMDA_RECEPTOR_ACTIVATION_EVENTS | REACTOME_POST_NM | 32 |
| ZEMBUTSU_SENSITIVITY_TO_NIMUSTINE | ZEMBUTSU_SENSITIVIT | 15 |
| REACTOME_CTLA4_INHIBITORY_SIGNALING | REACTOME_CTLA4_IN | 21 |
| PID_IL6_7_PATHWAY | PID_IL6_7_PATHWAY | 46 |
| LEE_LIVER_CANCER_SURVIVAL_UP | LEE_LIVER_CANCER_SI | 153 |
| AMIT_SERUM_RESPONSE_40_MCF10A | AMIT_SERUM_RESPON | 30 |
| LIN_NPAS4_TARGETS_DN | LIN_NPAS4_TARGETS_ | 61 |
| FLOTHO_PEDIATRIC_ALL_THERAPY_RESPONSE_UP | FLOTHO_PEDIATRIC_A | 50 |
| ALTEMEIER_RESPONSE_TO_LPS_WITH_MECHANICAL_VENTILATIC | ALTEMEIER_RESPONSE | 113 |
| BROWNE_HCMV_INFECTION_14HR_UP | BROWNE_HCMV_INFE | 138 |
| LIU_IL13_MEMORY_MODEL_UP | LIU_IL13_MEMORY_M | 16 |
| CHAUHAN_RESPONSE_TO_METHOXYESTRADIOL_UP | CHAUHAN_RESPONSE_ | 48 |
| GAURNIER_PSMD4_TARGETS | GAURNIER_PSMD4_TA | 48 |
| REACTOME_MRNA_SPLICING | REACTOME_MRNA_SP | 105 |
| REACTOME_FORMATION_OF_INCISION_COMPLEX_IN_GG_NER | REACTOME_FORMATI( | 20 |
| REACTOME_DEGRADATION_OF_THE_EXTRACELLULAR_MATRIX | REACTOME_DEGRADA | 26 |
| MEINHOLD_OVARIAN_CANCER_LOW_GRADE_UP | MEINHOLD_OVARIAN_ | 18 |
| HASLINGER_B_CLL_WITH_13Q14_DELETION | HASLINGER_B_CLL_WI | 21 |
| HOFMANN_MYELODYSPLASTIC_SYNDROM_LOW_RISK_UP | HOFMANN_MYELODYS | 20 |
| MATTIOLI_MGUS_VS_MULTIPLE_MYELOMA | MATTIOLI_MGUS_VS_ | 16 |
| KEGG_ADIPOCYTOKINE_SIGNALING_PATHWAY | KEGG_ADIPOCYTOKINI | 67 |
| KEGG_PYRUVATE_METABOLISM | KEGG_PYRUVATE_ME | 36 |
| REACTOME_PEPTIDE_LIGAND_BINDING_RECEPTORS | REACTOME_PEPTIDE_I | 167 |
| SIG_BCR_SIGNALING_PATHWAY | SIG_BCR_SIGNALING_I | 46 |
| NAGY_TFTC_COMPONENTS_HUMAN | NAGY_TFTC_COMPON | 17 |
| KEGG_PROTEASOME | KEGG_PROTEASOME | 44 |
| STARK_HYPPOCAMPUS_22Q11_DELETION_DN | STARK_HYPPOCAMPUS | 18 |
| ELVIDGE_HIF1A_AND_HIF2A_TARGETS_UP | ELVIDGE_HIF1A_AND_ | 36 |

| | | |
|---|---|---|
| ELVIDGE_HYPOXIA_DN | ELVIDGE_HYPOXIA_DN | 134 |
| PID_CD8_TCR_DOWNSTREAM_PATHWAY | PID_CD8_TCR_DOWNS | 56 |
| LUI_THYROID_CANCER_PAX8_PPARG_DN | LUI_THYROID_CANCER | 43 |
| KUROZUMI_RESPONSE_TO_ONCOCYTIC_VIRUS | KUROZUMI_RESPONSE | 42 |
| KUNINGER_IGF1_VS_PDGFB_TARGETS_UP | KUNINGER_IGF1_VS_P | 77 |
| REACTOME_DESTABILIZATION_OF_MRNA_BY_TRISTETRAPROLIN_ | REACTOME_DESTABILI | 17 |
| MCGOWAN_RSP6_TARGETS_UP | MCGOWAN_RSP6_TAR | 17 |
| ELVIDGE_HIF1A_TARGETS_UP | ELVIDGE_HIF1A_TARG | 62 |
| MCCLUNG_COCAINE_REWARD_5D | MCCLUNG_COCAINE_I | 78 |
| CAIRO_PML_TARGETS_BOUND_BY_MYC_UP | CAIRO_PML_TARGETS_ | 22 |
| GARGALOVIC_RESPONSE_TO_OXIDIZED_PHOSPHOLIPIDS_GREEN_ | GARGALOVIC_RESPON | 23 |
| PID_PLK1_PATHWAY | PID_PLK1_PATHWAY | 45 |
| WANG_METASTASIS_OF_BREAST_CANCER_ESR1_UP | WANG_METASTASIS_C | 19 |
| NIKOLSKY_BREAST_CANCER_5P15_AMPLICON | NIKOLSKY_BREAST_CA | 24 |
| SANA_TNF_SIGNALING_UP | SANA_TNF_SIGNALING | 73 |
| LY_AGING_OLD_DN | LY_AGING_OLD_DN | 55 |
| FLORIO_NEOCORTEX_BASAL_RADIAL_GLIA_DN | FLORIO_NEOCORTEX_I | 168 |
| REACTOME_SYNTHESIS_OF_PIPS_AT_THE_GOLGI_MEMBRANE | REACTOME_SYNTHESIS | 16 |
| LEE_LIVER_CANCER_MYC_DN | LEE_LIVER_CANCER_M | 55 |
| REACTOME_REGULATION_OF_MITOTIC_CELL_CYCLE | REACTOME_REGULATI | 76 |
| KYNG_RESPONSE_TO_H2O2_VIA_ERCC6 | KYNG_RESPONSE_TO_ | 16 |
| MATZUK_MEIOTIC_AND_DNA_REPAIR | MATZUK_MEIOTIC_AN | 39 |
| CHANG_IMMORTALIZED_BY_HPV31_DN | CHANG_IMMORTALIZE | 55 |
| WEBER_METHYLATED_ICP_IN_FIBROBLAST | WEBER_METHYLATED_ | 19 |
| RADAEVA_RESPONSE_TO_IFNA1_UP | RADAEVA_RESPONSE_ | 45 |
| SCHLOSSER_MYC_AND_SERUM_RESPONSE_SYNERGY | SCHLOSSER_MYC_AND | 30 |
| PID_IL12_STAT4_PATHWAY | PID_IL12_STAT4_PATH | 31 |
| KEGG_RIG_I_LIKE_RECEPTOR_SIGNALING_PATHWAY | KEGG_RIG_I_LIKE_REC | 64 |
| REACTOME_APC_C_CDH1_MEDIATED_DEGRADATION_OF_CDC20 | REACTOME_APC_C_CD | 64 |
| MORI_EMU_MYC_LYMPHOMA_BY_ONSET_TIME_UP | MORI_EMU_MYC_LYM | 99 |
| REICHERT_MITOSIS_LIN9_TARGETS | REICHERT_MITOSIS_LI | 28 |
| REACTOME_APC_C_CDC20_MEDIATED_DEGRADATION_OF_MITC | REACTOME_APC_C_CD | 65 |
| REACTOME_METAL_ION_SLC_TRANSPORTERS | REACTOME_METAL_IC | 22 |
| ASGHARZADEH_NEUROBLASTOMA_POOR_SURVIVAL_DN | ASGHARZADEH_NEUR | 36 |
| ZHANG_ANTIVIRAL_RESPONSE_TO_RIBAVIRIN_UP | ZHANG_ANTIVIRAL_RE | 25 |
| WHITFIELD_CELL_CYCLE_G2 | WHITFIELD_CELL_CYCL | 162 |
| YAO_TEMPORAL_RESPONSE_TO_PROGESTERONE_CLUSTER_11 | YAO_TEMPORAL_RESP | 97 |
| REACTOME_METABOLISM_OF_RNA | REACTOME_METABOL | 249 |
| FERRANDO_HOX11_NEIGHBORS | FERRANDO_HOX11_NI | 17 |
| REACTOME_GABA_SYNTHESIS_RELEASE_REUPTAKE_AND_DEGRA | REACTOME_GABA_SYN | 17 |
| NAKAMURA_CANCER_MICROENVIRONMENT_DN | NAKAMURA_CANCER_ | 44 |
| REACTOME_RESPIRATORY_ELECTRON_TRANSPORT | REACTOME_RESPIRAT | 62 |
| NIKOLSKY_BREAST_CANCER_12Q24_AMPLICON | NIKOLSKY_BREAST_CA | 15 |
| BIOCARTA_CYTOKINE_PATHWAY | BIOCARTA_CYTOKINE_ | 21 |
| REACTOME_RESPIRATORY_ELECTRON_TRANSPORT_ATP_SYNTHE | REACTOME_RESPIRAT | 77 |
| MOSERLE_IFNA_RESPONSE | MOSERLE_IFNA_RESPO | 23 |
| WANG_RESPONSE_TO_FORSKOLIN_UP | WANG_RESPONSE_TO | 20 |
| REACTOME_INHIBITION_OF_THE_PROTEOLYTIC_ACTIVITY_OF_AP | REACTOME_INHIBITIO | 18 |
| FARMER_BREAST_CANCER_CLUSTER_1 | FARMER_BREAST_CAN | 36 |
| REACTOME_APC_CDC20_MEDIATED_DEGRADATION_OF_NEK2A | REACTOME_APC_CDC2 | 21 |

| ES | NES | NOM p-val | FDR q-val | FWER p-val | RANK AT M | LEADING EDGE |
|---|---|---|---|---|---|---|
| 0.680545 | 1.672257 | 0.013619 | 1 | 0.86 | 1858 | tags=44%, list=8%, signal=47% |
| 0.631202 | 1.65186 | 0.004902 | 1 | 0.938 | 2859 | tags=56%, list=12%, signal=64% |
| 0.604464 | 1.644647 | 0.014706 | 1 | 0.952 | 2099 | tags=40%, list=9%, signal=44% |
| 0.491411 | 1.616967 | 0.018939 | 1 | 0.98 | 3236 | tags=35%, list=14%, signal=41% |
| 0.686999 | 1.599272 | 0 | 1 | 0.999 | 4556 | tags=56%, list=19%, signal=69% |
| 0.480857 | 1.586804 | 0 | 1 | 0.999 | 3386 | tags=38%, list=14%, signal=45% |
| 0.598134 | 1.58587 | 0.041441 | 1 | 0.999 | 3307 | tags=55%, list=14%, signal=64% |
| 0.564362 | 1.534517 | 0.03252 | 1 | 1 | 3536 | tags=56%, list=15%, signal=65% |
| 0.667918 | 1.497019 | 0.058601 | 1 | 1 | 4100 | tags=50%, list=18%, signal=61% |
| 0.495566 | 1.494685 | 0.00491 | 1 | 1 | 2494 | tags=32%, list=11%, signal=35% |
| 0.601752 | 1.487991 | 0.03964 | 1 | 1 | 5283 | tags=53%, list=23%, signal=68% |
| 0.583409 | 1.473757 | 0.041096 | 1 | 1 | 4437 | tags=59%, list=19%, signal=73% |
| 0.567627 | 1.47203 | 0.033074 | 1 | 1 | 1488 | tags=39%, list=6%, signal=41% |
| 0.545624 | 1.467806 | 0.036907 | 1 | 1 | 1948 | tags=33%, list=8%, signal=36% |
| 0.568725 | 1.461357 | 0.084922 | 1 | 1 | 2148 | tags=37%, list=9%, signal=41% |
| 0.539844 | 1.459822 | 0.024834 | 1 | 1 | 5150 | tags=51%, list=22%, signal=65% |
| 0.516488 | 1.44987 | 0.018364 | 1 | 1 | 1794 | tags=42%, list=8%, signal=46% |
| 0.562629 | 1.435799 | 0.053381 | 1 | 1 | 3777 | tags=48%, list=16%, signal=57% |
| 0.583532 | 1.431081 | 0.073529 | 1 | 1 | 5709 | tags=67%, list=24%, signal=88% |
| 0.532281 | 1.424943 | 0.061111 | 1 | 1 | 4349 | tags=50%, list=19%, signal=61% |
| 0.47903 | 1.422437 | 0.042254 | 1 | 1 | 7331 | tags=62%, list=31%, signal=89% |
| 0.606915 | 1.420582 | 0.053963 | 1 | 1 | 3520 | tags=59%, list=15%, signal=69% |
| 0.577177 | 1.419244 | 0.066318 | 1 | 1 | 4785 | tags=56%, list=20%, signal=70% |
| 0.5274 | 1.41806 | 0.086667 | 1 | 1 | 1710 | tags=35%, list=7%, signal=38% |
| 0.509406 | 1.417695 | 0.047022 | 1 | 1 | 3313 | tags=47%, list=14%, signal=55% |
| 0.646336 | 1.414467 | 0.08167 | 1 | 1 | 4486 | tags=61%, list=19%, signal=75% |
| 0.44695 | 1.413051 | 0.005146 | 1 | 1 | 3897 | tags=41%, list=17%, signal=49% |
| 0.494085 | 1.408886 | 0.029762 | 1 | 1 | 5291 | tags=62%, list=23%, signal=80% |
| 0.544234 | 1.40763 | 0.041958 | 1 | 1 | 5391 | tags=63%, list=23%, signal=82% |
| 0.602536 | 1.404729 | 0.085981 | 1 | 1 | 1236 | tags=25%, list=5%, signal=26% |
| 0.498275 | 1.401695 | 0.098993 | 1 | 1 | 3977 | tags=38%, list=17%, signal=46% |
| 0.523636 | 1.398434 | 0.102178 | 1 | 1 | 1313 | tags=33%, list=6%, signal=35% |
| 0.597964 | 1.395762 | 0.085859 | 1 | 1 | 4009 | tags=50%, list=17%, signal=60% |
| 0.517665 | 1.392352 | 0.056385 | 1 | 1 | 6598 | tags=59%, list=28%, signal=82% |
| 0.556331 | 1.391015 | 0.059701 | 1 | 1 | 2847 | tags=34%, list=12%, signal=39% |
| 0.483389 | 1.390908 | 0.086601 | 1 | 1 | 2417 | tags=42%, list=10%, signal=47% |
| 0.520025 | 1.390236 | 0.055133 | 1 | 1 | 1802 | tags=32%, list=8%, signal=34% |
| 0.527433 | 1.378759 | 0.094444 | 1 | 1 | 5673 | tags=67%, list=24%, signal=88% |
| 0.452378 | 1.378506 | 0.017065 | 1 | 1 | 3725 | tags=41%, list=16%, signal=48% |
| 0.644528 | 1.377772 | 0.146691 | 1 | 1 | 6320 | tags=73%, list=27%, signal=99% |
| 0.512538 | 1.376122 | 0.040541 | 1 | 1 | 6608 | tags=59%, list=28%, signal=82% |
| 0.580406 | 1.375183 | 0.134513 | 1 | 1 | 6560 | tags=66%, list=28%, signal=91% |
| 0.370577 | 1.37032 | 0.083607 | 1 | 1 | 4644 | tags=38%, list=20%, signal=47% |
| 0.489269 | 1.367954 | 0.150838 | 1 | 1 | 5807 | tags=59%, list=25%, signal=78% |
| 0.619253 | 1.367864 | 0.145129 | 1 | 1 | 4100 | tags=47%, list=18%, signal=57% |
| 0.433237 | 1.367752 | 0.047945 | 1 | 1 | 5487 | tags=42%, list=23%, signal=55% |
| 0.637017 | 1.366832 | 0.082192 | 1 | 1 | 3122 | tags=41%, list=13%, signal=47% |
| 0.477196 | 1.365899 | 0.052464 | 1 | 1 | 4737 | tags=47%, list=20%, signal=58% |
| 0.572904 | 1.362678 | 0.110294 | 1 | 1 | 6391 | tags=61%, list=27%, signal=84% |

| | | | | | | |
|---|---|---|---|---|---|---|
| 0.505894 | 1.359484 | 0.089161 | 1 | 1 | 4008 | tags=45%, list=17%, signal=54% |
| 0.590537 | 1.357941 | 0.125461 | 1 | 1 | 3673 | tags=60%, list=16%, signal=71% |
| 0.40354 | 1.357871 | 0.02901 | 1 | 1 | 3233 | tags=33%, list=14%, signal=39% |
| 0.628716 | 1.357648 | 0.13447 | 1 | 1 | 4947 | tags=47%, list=21%, signal=59% |
| 0.546558 | 1.354487 | 0.086882 | 1 | 1 | 3725 | tags=39%, list=16%, signal=46% |
| 0.425918 | 1.351679 | 0.054795 | 1 | 1 | 3254 | tags=38%, list=14%, signal=45% |
| 0.491358 | 1.349196 | 0.156089 | 1 | 1 | 4708 | tags=48%, list=20%, signal=59% |
| 0.522608 | 1.347971 | 0.092496 | 1 | 1 | 3725 | tags=42%, list=16%, signal=50% |
| 0.496888 | 1.347184 | 0.15371 | 1 | 1 | 3536 | tags=43%, list=15%, signal=51% |
| 0.563286 | 1.343886 | 0.144246 | 1 | 1 | 5873 | tags=63%, list=25%, signal=83% |
| 0.49419 | 1.342458 | 0.094855 | 1 | 1 | 3496 | tags=39%, list=15%, signal=46% |
| 0.507774 | 1.341374 | 0.109244 | 1 | 1 | 4151 | tags=45%, list=18%, signal=54% |
| 0.513352 | 1.340498 | 0.089922 | 1 | 1 | 6304 | tags=59%, list=27%, signal=81% |
| 0.41168 | 1.339397 | 0.063574 | 1 | 1 | 5167 | tags=49%, list=22%, signal=63% |
| 0.425998 | 1.339114 | 0.103328 | 1 | 1 | 3368 | tags=35%, list=14%, signal=41% |
| 0.4086 | 1.335919 | 0.075286 | 1 | 1 | 4767 | tags=41%, list=20%, signal=51% |
| 0.404612 | 1.334177 | 0.055743 | 1 | 1 | 3647 | tags=40%, list=16%, signal=48% |
| 0.467583 | 1.329596 | 0.051887 | 1 | 1 | 6928 | tags=52%, list=30%, signal=73% |
| 0.450853 | 1.324943 | 0.172161 | 1 | 1 | 5590 | tags=52%, list=24%, signal=68% |
| 0.426247 | 1.324823 | 0.08121 | 1 | 1 | 2984 | tags=33%, list=13%, signal=37% |
| 0.563759 | 1.322628 | 0.132597 | 1 | 1 | 6412 | tags=63%, list=27%, signal=86% |
| 0.394216 | 1.32217 | 0.024038 | 1 | 1 | 5624 | tags=47%, list=24%, signal=61% |
| 0.492729 | 1.320863 | 0.108514 | 1 | 1 | 2932 | tags=46%, list=13%, signal=53% |
| 0.70448 | 1.319756 | 0.189139 | 1 | 1 | 5788 | tags=85%, list=25%, signal=113% |
| 0.437 | 1.319027 | 0.117537 | 1 | 1 | 4285 | tags=33%, list=18%, signal=41% |
| 0.665636 | 1.318543 | 0.191205 | 1 | 1 | 6392 | tags=77%, list=27%, signal=106% |
| 0.412273 | 1.315717 | 0.081129 | 1 | 1 | 3848 | tags=39%, list=16%, signal=46% |
| 0.410507 | 1.313461 | 0.120066 | 1 | 1 | 5796 | tags=46%, list=25%, signal=62% |
| 0.409899 | 1.313016 | 0.079861 | 1 | 1 | 4068 | tags=33%, list=17%, signal=39% |
| 0.458232 | 1.310551 | 0.141005 | 1 | 1 | 4991 | tags=55%, list=21%, signal=69% |
| 0.728193 | 1.308561 | 0.186508 | 1 | 1 | 4580 | tags=80%, list=20%, signal=99% |
| 0.474747 | 1.307475 | 0.055 | 1 | 1 | 3725 | tags=43%, list=16%, signal=51% |
| 0.594346 | 1.307417 | 0.180702 | 1 | 1 | 5796 | tags=57%, list=25%, signal=75% |
| 0.519234 | 1.30738 | 0.106646 | 1 | 1 | 5717 | tags=63%, list=24%, signal=83% |
| 0.565613 | 1.306791 | 0.151402 | 1 | 1 | 4702 | tags=60%, list=20%, signal=75% |
| 0.514219 | 1.305755 | 0.14733 | 1 | 1 | 3348 | tags=42%, list=14%, signal=49% |
| 0.459024 | 1.304135 | 0.100334 | 1 | 1 | 1937 | tags=28%, list=8%, signal=30% |
| 0.570925 | 1.303713 | 0.13059 | 1 | 1 | 7022 | tags=68%, list=30%, signal=97% |
| 0.679094 | 1.302692 | 0.22973 | 1 | 1 | 5246 | tags=74%, list=22%, signal=96% |
| 0.586319 | 1.301986 | 0.224335 | 1 | 1 | 7502 | tags=70%, list=32%, signal=103% |
| 0.505813 | 1.298351 | 0.083893 | 1 | 1 | 3725 | tags=53%, list=16%, signal=63% |
| 0.567611 | 1.296745 | 0.152364 | 1 | 1 | 7022 | tags=70%, list=30%, signal=100% |
| 0.407744 | 1.295323 | 0.051852 | 1 | 1 | 4193 | tags=37%, list=18%, signal=45% |
| 0.519816 | 1.293536 | 0.261818 | 1 | 1 | 5880 | tags=50%, list=25%, signal=67% |
| 0.420601 | 1.291432 | 0.144621 | 1 | 1 | 3602 | tags=33%, list=15%, signal=39% |
| 0.459948 | 1.290589 | 0.140962 | 1 | 1 | 2601 | tags=32%, list=11%, signal=36% |
| 0.403839 | 1.285979 | 0.108896 | 1 | 1 | 5868 | tags=47%, list=25%, signal=63% |
| 0.402873 | 1.285862 | 0.079872 | 1 | 1 | 3051 | tags=26%, list=13%, signal=30% |
| 0.416838 | 1.285383 | 0.06535 | 1 | 1 | 4038 | tags=37%, list=17%, signal=44% |
| 0.583336 | 1.284639 | 0.286252 | 1 | 1 | 5580 | tags=63%, list=24%, signal=82% |

| | | | | | | |
|---|---|---|---|---|---|---|
| 0.432992 | 1.282703 | 0.101881 | 1 | 1 | 4358 | tags=38%, list=19%, signal=46% |
| 0.410841 | 1.281529 | 0.116883 | 1 | 1 | 3554 | tags=37%, list=15%, signal=43% |
| 0.705808 | 1.281031 | 0.225989 | 1 | 1 | 5182 | tags=83%, list=22%, signal=106% |
| 0.538319 | 1.280501 | 0.164602 | 1 | 1 | 6405 | tags=72%, list=27%, signal=99% |
| 0.524381 | 1.279896 | 0.171123 | 1 | 1 | 3921 | tags=60%, list=17%, signal=72% |
| 0.457433 | 1.279724 | 0.251838 | 1 | 1 | 5962 | tags=51%, list=25%, signal=68% |
| 0.445231 | 1.279227 | 0.199248 | 1 | 1 | 5348 | tags=50%, list=23%, signal=64% |
| 0.396857 | 1.277981 | 0.066919 | 1 | 1 | 1552 | tags=27%, list=7%, signal=29% |
| 0.473549 | 1.275229 | 0.139219 | 1 | 1 | 3082 | tags=36%, list=13%, signal=42% |
| 0.604926 | 1.27517 | 0.297989 | 1 | 1 | 5607 | tags=67%, list=24%, signal=88% |
| 0.430464 | 1.274636 | 0.11938 | 1 | 1 | 5501 | tags=56%, list=23%, signal=73% |
| 0.467474 | 1.274154 | 0.166362 | 1 | 1 | 5852 | tags=60%, list=25%, signal=80% |
| 0.390906 | 1.273772 | 0.117978 | 1 | 1 | 2654 | tags=28%, list=11%, signal=32% |
| 0.477035 | 1.272291 | 0.191829 | 1 | 1 | 6361 | tags=57%, list=27%, signal=78% |
| 0.578734 | 1.272157 | 0.363167 | 1 | 1 | 5880 | tags=61%, list=25%, signal=82% |
| 0.518779 | 1.271493 | 0.209262 | 1 | 1 | 4458 | tags=55%, list=19%, signal=67% |
| 0.355515 | 1.271427 | 0.056075 | 1 | 1 | 4412 | tags=35%, list=19%, signal=43% |
| 0.394554 | 1.271388 | 0.091772 | 1 | 1 | 3725 | tags=34%, list=16%, signal=41% |
| 0.455776 | 1.270634 | 0.207012 | 1 | 1 | 2712 | tags=35%, list=12%, signal=40% |
| 0.377884 | 1.269898 | 0.153014 | 1 | 1 | 5808 | tags=53%, list=25%, signal=70% |
| 0.604112 | 1.268078 | 0.3213 | 1 | 1 | 5607 | tags=62%, list=24%, signal=81% |
| 0.408191 | 1.266961 | 0.138632 | 1 | 1 | 6365 | tags=58%, list=27%, signal=80% |
| 0.466495 | 1.266311 | 0.150316 | 1 | 1 | 3342 | tags=53%, list=14%, signal=62% |
| 0.651398 | 1.266117 | 0.232824 | 1 | 1 | 7774 | tags=88%, list=33%, signal=131% |
| 0.411937 | 1.265937 | 0.150641 | 1 | 1 | 6866 | tags=51%, list=29%, signal=71% |
| 0.519848 | 1.264281 | 0.196565 | 1 | 1 | 2409 | tags=38%, list=10%, signal=42% |
| 0.629135 | 1.263555 | 0.265537 | 1 | 1 | 5857 | tags=76%, list=25%, signal=102% |
| 0.434749 | 1.259399 | 0.152685 | 1 | 1 | 2409 | tags=30%, list=10%, signal=33% |
| 0.488132 | 1.259005 | 0.197869 | 1 | 1 | 7092 | tags=67%, list=30%, signal=96% |
| 0.48515 | 1.258713 | 0.164983 | 1 | 1 | 1440 | tags=29%, list=6%, signal=30% |
| 0.358977 | 1.257662 | 0.118584 | 1 | 1 | 4410 | tags=36%, list=19%, signal=44% |
| 0.517125 | 1.254345 | 0.126488 | 1 | 1 | 7868 | tags=79%, list=34%, signal=119% |
| 0.477057 | 1.254021 | 0.179245 | 1 | 1 | 5531 | tags=53%, list=24%, signal=69% |
| 0.613452 | 1.252378 | 0.273256 | 1 | 1 | 7808 | tags=86%, list=33%, signal=129% |
| 0.408467 | 1.251862 | 0.20794 | 1 | 1 | 5776 | tags=46%, list=25%, signal=61% |
| 0.514729 | 1.251419 | 0.261682 | 1 | 1 | 3011 | tags=38%, list=13%, signal=43% |
| 0.564242 | 1.250853 | 0.328302 | 1 | 1 | 7330 | tags=68%, list=31%, signal=99% |
| 0.447135 | 1.250847 | 0.186087 | 1 | 1 | 6866 | tags=48%, list=29%, signal=68% |
| 0.412414 | 1.250538 | 0.172308 | 1 | 1 | 5831 | tags=47%, list=25%, signal=63% |
| 0.348667 | 1.250157 | 0.118297 | 1 | 1 | 4169 | tags=37%, list=18%, signal=45% |
| 0.423588 | 1.247696 | 0.248696 | 1 | 1 | 5612 | tags=48%, list=24%, signal=62% |
| 0.451088 | 1.247121 | 0.167752 | 1 | 1 | 2995 | tags=40%, list=13%, signal=46% |
| 0.70114 | 1.246304 | 0.271484 | 1 | 1 | 6320 | tags=89%, list=27%, signal=122% |
| 0.470192 | 1.245229 | 0.230038 | 1 | 1 | 4100 | tags=36%, list=18%, signal=43% |
| 0.438647 | 1.244253 | 0.182906 | 1 | 1 | 2030 | tags=37%, list=9%, signal=40% |
| 0.549855 | 1.243172 | 0.24552 | 1 | 1 | 6320 | tags=53%, list=27%, signal=72% |
| 0.547912 | 1.242036 | 0.286957 | 1 | 1 | 3326 | tags=45%, list=14%, signal=53% |
| 0.499567 | 1.241752 | 0.207207 | 1 | 1 | 2413 | tags=35%, list=10%, signal=39% |
| 0.331273 | 1.24104 | 0.097879 | 1 | 1 | 4600 | tags=33%, list=20%, signal=41% |
| 0.439716 | 1.239585 | 0.166189 | 1 | 1 | 6634 | tags=60%, list=28%, signal=84% |

| | | | | | | |
|---|---|---|---|---|---|---|
| 0.557369 | 1.237357 | 0.259194 | 1 | 1 | 6591 | tags=53%, list=28%, signal=74% |
| 0.399471 | 1.235399 | 0.160494 | 1 | 1 | 2775 | tags=32%, list=12%, signal=36% |
| 0.68968 | 1.234324 | 0.281955 | 1 | 1 | 6320 | tags=87%, list=27%, signal=119% |
| 0.680822 | 1.232782 | 0.265469 | 1 | 1 | 5182 | tags=80%, list=22%, signal=103% |
| 0.564072 | 1.231775 | 0.254025 | 1 | 1 | 7358 | tags=78%, list=31%, signal=113% |
| 0.414692 | 1.231443 | 0.150165 | 1 | 1 | 5857 | tags=48%, list=25%, signal=63% |
| 0.607428 | 1.230416 | 0.259259 | 1 | 1 | 1754 | tags=50%, list=7%, signal=54% |
| 0.685963 | 1.230272 | 0.308958 | 1 | 1 | 5943 | tags=76%, list=25%, signal=102% |
| 0.349359 | 1.230058 | 0.154514 | 1 | 1 | 5540 | tags=43%, list=24%, signal=56% |
| 0.473755 | 1.229047 | 0.187994 | 1 | 1 | 5245 | tags=48%, list=22%, signal=62% |
| 0.358398 | 1.228789 | 0.076692 | 1 | 1 | 2738 | tags=26%, list=12%, signal=29% |
| 0.592587 | 1.228541 | 0.220826 | 1 | 1 | 6249 | tags=60%, list=27%, signal=82% |
| 0.494361 | 1.228057 | 0.216549 | 1 | 1 | 4628 | tags=47%, list=20%, signal=59% |
| 0.340199 | 1.226174 | 0.103093 | 1 | 1 | 3571 | tags=30%, list=15%, signal=36% |
| 0.436608 | 1.2256 | 0.207447 | 1 | 1 | 3320 | tags=40%, list=14%, signal=47% |
| 0.318149 | 1.225092 | 0.080357 | 1 | 1 | 3440 | tags=29%, list=15%, signal=34% |
| 0.454449 | 1.224878 | 0.181388 | 1 | 1 | 5813 | tags=41%, list=25%, signal=55% |
| 0.647665 | 1.22446 | 0.290385 | 1 | 1 | 5353 | tags=73%, list=23%, signal=95% |
| 0.429777 | 1.224425 | 0.246479 | 1 | 1 | 4575 | tags=44%, list=20%, signal=55% |
| 0.432742 | 1.224064 | 0.166667 | 1 | 1 | 4279 | tags=51%, list=18%, signal=63% |
| 0.497001 | 1.223812 | 0.226415 | 1 | 1 | 5283 | tags=44%, list=23%, signal=57% |
| 0.435023 | 1.223497 | 0.20177 | 1 | 1 | 2314 | tags=33%, list=10%, signal=36% |
| 0.596295 | 1.223472 | 0.243137 | 1 | 1 | 2110 | tags=38%, list=9%, signal=42% |
| 0.501734 | 1.221545 | 0.238434 | 1 | 1 | 5395 | tags=47%, list=23%, signal=61% |
| 0.372431 | 1.221539 | 0.131693 | 1 | 1 | 1187 | tags=17%, list=5%, signal=18% |
| 0.457459 | 1.221215 | 0.227826 | 1 | 1 | 7452 | tags=71%, list=32%, signal=105% |
| 0.475543 | 1.220373 | 0.245318 | 1 | 1 | 3739 | tags=44%, list=16%, signal=52% |
| 0.551999 | 1.220052 | 0.269231 | 1 | 1 | 6095 | tags=58%, list=26%, signal=78% |
| 0.556659 | 1.218188 | 0.370229 | 1 | 1 | 7330 | tags=69%, list=31%, signal=99% |
| 0.464778 | 1.216737 | 0.21777 | 1 | 1 | 5852 | tags=56%, list=25%, signal=75% |
| 0.443206 | 1.215202 | 0.255238 | 1 | 1 | 5167 | tags=47%, list=22%, signal=60% |
| 0.336857 | 1.214811 | 0.143564 | 1 | 1 | 4509 | tags=31%, list=19%, signal=38% |
| 0.30998 | 1.214508 | 0.082781 | 1 | 1 | 3171 | tags=24%, list=14%, signal=28% |
| 0.484837 | 1.214402 | 0.254937 | 1 | 1 | 5874 | tags=55%, list=25%, signal=74% |
| 0.425299 | 1.214235 | 0.18 | 1 | 1 | 2279 | tags=30%, list=10%, signal=34% |
| 0.503749 | 1.214147 | 0.269531 | 1 | 1 | 5900 | tags=53%, list=25%, signal=70% |
| 0.449031 | 1.213463 | 0.292035 | 1 | 1 | 6074 | tags=50%, list=26%, signal=67% |
| 0.513523 | 1.21279 | 0.202381 | 1 | 1 | 4886 | tags=59%, list=21%, signal=75% |
| 0.55594 | 1.211214 | 0.254753 | 1 | 1 | 7774 | tags=79%, list=33%, signal=117% |
| 0.387285 | 1.207764 | 0.175862 | 1 | 1 | 5882 | tags=49%, list=25%, signal=65% |
| 0.604051 | 1.207418 | 0.327715 | 1 | 1 | 5353 | tags=66%, list=23%, signal=85% |
| 0.362591 | 1.207005 | 0.195402 | 1 | 1 | 5555 | tags=42%, list=24%, signal=55% |
| 0.518108 | 1.20687 | 0.258348 | 1 | 1 | 5003 | tags=47%, list=21%, signal=60% |
| 0.354152 | 1.206425 | 0.229205 | 1 | 1 | 4333 | tags=35%, list=19%, signal=43% |
| 0.424994 | 1.206362 | 0.231579 | 1 | 1 | 5615 | tags=52%, list=24%, signal=68% |
| 0.427104 | 1.206191 | 0.245098 | 1 | 1 | 865 | tags=20%, list=4%, signal=21% |
| 0.465215 | 1.206021 | 0.213445 | 1 | 1 | 5838 | tags=52%, list=25%, signal=69% |
| 0.316379 | 1.205715 | 0.135621 | 1 | 1 | 2842 | tags=24%, list=12%, signal=27% |
| 0.472654 | 1.205243 | 0.274854 | 1 | 1 | 6866 | tags=50%, list=29%, signal=71% |
| 0.367313 | 1.205178 | 0.144781 | 1 | 1 | 5094 | tags=34%, list=22%, signal=43% |

| | | | | | | |
|---|---|---|---|---|---|---|
| 0.370921 | 1.205075 | 0.18705 | 1 | 1 | 3361 | tags=33%, list=14%, signal=39% |
| 0.293494 | 1.204484 | 0.06266 | 1 | 1 | 4436 | tags=32%, list=19%, signal=38% |
| 0.436291 | 1.203899 | 0.19702 | 1 | 1 | 2795 | tags=44%, list=12%, signal=50% |
| 0.418164 | 1.203817 | 0.19376 | 1 | 1 | 92 | tags=13%, list=0%, signal=13% |
| 0.57496 | 1.202803 | 0.322404 | 1 | 1 | 7805 | tags=78%, list=33%, signal=117% |
| 0.518294 | 1.202452 | 0.314991 | 1 | 1 | 5718 | tags=52%, list=24%, signal=69% |
| 0.37098 | 1.202292 | 0.182131 | 1 | 1 | 6821 | tags=50%, list=29%, signal=70% |
| 0.384654 | 1.202075 | 0.178306 | 1 | 1 | 5831 | tags=44%, list=25%, signal=58% |
| 0.586221 | 1.201498 | 0.372477 | 1 | 1 | 4580 | tags=63%, list=20%, signal=78% |
| 0.442668 | 1.201305 | 0.220826 | 1 | 1 | 1496 | tags=29%, list=6%, signal=31% |
| 0.397084 | 1.199365 | 0.227907 | 1 | 1 | 4672 | tags=43%, list=20%, signal=53% |
| 0.407408 | 1.19883 | 0.193162 | 1 | 1 | 671 | tags=18%, list=3%, signal=19% |
| 0.307806 | 1.198478 | 0.103914 | 1 | 1 | 4483 | tags=37%, list=19%, signal=46% |
| 0.549074 | 1.196961 | 0.325279 | 1 | 1 | 5875 | tags=65%, list=25%, signal=86% |
| 0.419948 | 1.196957 | 0.155449 | 1 | 1 | 2816 | tags=32%, list=12%, signal=36% |
| 0.441004 | 1.196938 | 0.243243 | 1 | 1 | 5503 | tags=46%, list=23%, signal=60% |
| 0.338237 | 1.196729 | 0.129518 | 1 | 1 | 5717 | tags=44%, list=24%, signal=58% |
| 0.465772 | 1.196561 | 0.256778 | 1 | 1 | 4952 | tags=52%, list=21%, signal=66% |
| 0.439138 | 1.195811 | 0.208824 | 1 | 1 | 2145 | tags=29%, list=9%, signal=31% |
| 0.357766 | 1.192867 | 0.159057 | 1 | 1 | 1614 | tags=28%, list=7%, signal=30% |
| 0.40995 | 1.191819 | 0.268376 | 1 | 1 | 3631 | tags=33%, list=16%, signal=39% |
| 0.424315 | 1.191651 | 0.293624 | 1 | 1 | 6279 | tags=59%, list=27%, signal=80% |
| 0.433295 | 1.191449 | 0.300178 | 1 | 1 | 7525 | tags=59%, list=32%, signal=87% |
| 0.356367 | 1.190032 | 0.176471 | 1 | 1 | 4321 | tags=39%, list=18%, signal=48% |
| 0.496872 | 1.189727 | 0.328996 | 1 | 1 | 6137 | tags=50%, list=26%, signal=68% |
| 0.486747 | 1.189644 | 0.354015 | 1 | 1 | 5432 | tags=52%, list=23%, signal=67% |
| 0.423562 | 1.189334 | 0.187296 | 1 | 1 | 3725 | tags=36%, list=16%, signal=43% |
| 0.452406 | 1.189111 | 0.349177 | 1 | 1 | 5665 | tags=52%, list=24%, signal=68% |
| 0.335457 | 1.188544 | 0.176211 | 1 | 1 | 4647 | tags=39%, list=20%, signal=49% |
| 0.375859 | 1.18773 | 0.201493 | 1 | 1 | 3276 | tags=31%, list=14%, signal=36% |
| 0.390253 | 1.187169 | 0.193069 | 1 | 1 | 5643 | tags=43%, list=24%, signal=57% |
| 0.312322 | 1.186791 | 0.190549 | 1 | 1 | 5744 | tags=41%, list=25%, signal=53% |
| 0.387965 | 1.186491 | 0.180851 | 1 | 1 | 3878 | tags=37%, list=17%, signal=44% |
| 0.45445 | 1.185113 | 0.238422 | 1 | 1 | 1026 | tags=22%, list=4%, signal=23% |
| 0.528636 | 1.184873 | 0.258224 | 1 | 1 | 6612 | tags=45%, list=28%, signal=63% |
| 0.55079 | 1.183304 | 0.414159 | 1 | 1 | 5880 | tags=56%, list=25%, signal=75% |
| 0.376272 | 1.182667 | 0.172131 | 1 | 1 | 1442 | tags=19%, list=6%, signal=20% |
| 0.346912 | 1.180959 | 0.163328 | 1 | 1 | 6513 | tags=45%, list=28%, signal=63% |
| 0.455502 | 1.179856 | 0.269982 | 1 | 1 | 6006 | tags=60%, list=26%, signal=81% |
| 0.535355 | 1.17816 | 0.291727 | 1 | 1 | 6914 | tags=71%, list=30%, signal=100% |
| 0.346563 | 1.178012 | 0.197248 | 1 | 1 | 2798 | tags=32%, list=12%, signal=36% |
| 0.380119 | 1.176412 | 0.214176 | 1 | 1 | 4839 | tags=43%, list=21%, signal=54% |
| 0.378779 | 1.176121 | 0.226937 | 1 | 1 | 4489 | tags=42%, list=19%, signal=52% |
| 0.445435 | 1.175512 | 0.230384 | 1 | 1 | 5575 | tags=55%, list=24%, signal=72% |
| 0.505811 | 1.173893 | 0.278899 | 1 | 1 | 5993 | tags=53%, list=26%, signal=71% |
| 0.313882 | 1.173883 | 0.129679 | 1 | 1 | 794 | tags=15%, list=3%, signal=16% |
| 0.530161 | 1.172924 | 0.340824 | 1 | 1 | 6281 | tags=60%, list=27%, signal=82% |
| 0.345401 | 1.172685 | 0.201893 | 1 | 1 | 4338 | tags=37%, list=19%, signal=45% |
| 0.351913 | 1.17113 | 0.156442 | 1 | 1 | 1568 | tags=22%, list=7%, signal=24% |
| 0.523309 | 1.171056 | 0.263538 | 1 | 1 | 4233 | tags=43%, list=18%, signal=52% |

| | | | | | | |
|---|---|---|---|---|---|---|
| 0.342939 | 1.170863 | 0.164521 | 1 | 1 | 2795 | tags=27%, list=12%, signal=31% |
| 0.34553 | 1.170851 | 0.232082 | 1 | 1 | 3320 | tags=32%, list=14%, signal=36% |
| 0.368859 | 1.170462 | 0.213333 | 1 | 1 | 4666 | tags=37%, list=20%, signal=46% |
| 0.341882 | 1.170287 | 0.206897 | 1 | 1 | 5675 | tags=45%, list=24%, signal=59% |
| 0.353037 | 1.169285 | 0.213166 | 1 | 1 | 5555 | tags=42%, list=24%, signal=55% |
| 0.504104 | 1.169249 | 0.256735 | 1 | 1 | 3432 | tags=44%, list=15%, signal=52% |
| 0.477836 | 1.169025 | 0.257749 | 1 | 1 | 6152 | tags=43%, list=26%, signal=59% |
| 0.482257 | 1.168065 | 0.373626 | 1 | 1 | 5880 | tags=48%, list=25%, signal=64% |
| 0.532591 | 1.168015 | 0.411321 | 1 | 1 | 7174 | tags=62%, list=31%, signal=89% |
| 0.349147 | 1.16762 | 0.181818 | 1 | 1 | 4641 | tags=38%, list=20%, signal=47% |
| 0.631825 | 1.167273 | 0.377863 | 1 | 1 | 7437 | tags=78%, list=32%, signal=115% |
| 0.561725 | 1.167192 | 0.338462 | 1 | 1 | 7358 | tags=75%, list=31%, signal=109% |
| 0.466296 | 1.16696 | 0.344643 | 1 | 1 | 5647 | tags=59%, list=24%, signal=77% |
| 0.506519 | 1.166306 | 0.296361 | 1 | 1 | 6840 | tags=67%, list=29%, signal=94% |
| 0.405596 | 1.166174 | 0.223333 | 1 | 1 | 3878 | tags=40%, list=17%, signal=48% |
| 0.33873 | 1.165922 | 0.232682 | 1 | 1 | 5615 | tags=45%, list=24%, signal=59% |
| 0.309707 | 1.165445 | 0.178849 | 1 | 1 | 3105 | tags=27%, list=13%, signal=31% |
| 0.475372 | 1.165083 | 0.294521 | 1 | 1 | 4478 | tags=44%, list=19%, signal=54% |
| 0.318972 | 1.16472 | 0.191395 | 1 | 1 | 5962 | tags=45%, list=25%, signal=59% |
| 0.549007 | 1.163082 | 0.3049 | 1 | 1 | 3667 | tags=43%, list=16%, signal=51% |
| 0.385059 | 1.162396 | 0.276423 | 1 | 1 | 6225 | tags=51%, list=27%, signal=70% |
| 0.416229 | 1.16225 | 0.248418 | 1 | 1 | 2270 | tags=31%, list=10%, signal=35% |
| 0.447231 | 1.161597 | 0.345283 | 1 | 1 | 6320 | tags=53%, list=27%, signal=72% |
| 0.344216 | 1.161354 | 0.249191 | 1 | 1 | 3993 | tags=34%, list=17%, signal=40% |
| 0.464086 | 1.161059 | 0.234589 | 1 | 1 | 2320 | tags=26%, list=10%, signal=29% |
| 0.391469 | 1.160768 | 0.231041 | 1 | 1 | 2966 | tags=32%, list=13%, signal=36% |
| 0.497711 | 1.159677 | 0.3 | 1 | 1 | 7448 | tags=61%, list=32%, signal=90% |
| 0.395448 | 1.158466 | 0.305357 | 1 | 1 | 5828 | tags=53%, list=25%, signal=70% |
| 0.434559 | 1.158255 | 0.277338 | 1 | 1 | 1277 | tags=33%, list=5%, signal=35% |
| 0.566468 | 1.15712 | 0.356239 | 1 | 1 | 6074 | tags=53%, list=26%, signal=72% |
| 0.541023 | 1.155604 | 0.33829 | 1 | 1 | 6914 | tags=67%, list=30%, signal=94% |
| 0.320704 | 1.155467 | 0.182222 | 1 | 1 | 3391 | tags=35%, list=14%, signal=40% |
| 0.392744 | 1.155467 | 0.249551 | 1 | 1 | 5930 | tags=47%, list=25%, signal=62% |
| 0.377768 | 1.155214 | 0.257475 | 1 | 1 | 4641 | tags=44%, list=20%, signal=54% |
| 0.381941 | 1.155125 | 0.250923 | 1 | 1 | 2590 | tags=29%, list=11%, signal=32% |
| 0.318983 | 1.154979 | 0.210435 | 1 | 1 | 5651 | tags=41%, list=24%, signal=54% |
| 0.603896 | 1.154793 | 0.334477 | 1 | 1 | 4761 | tags=73%, list=20%, signal=92% |
| 0.380659 | 1.153437 | 0.259615 | 1 | 1 | 5870 | tags=47%, list=25%, signal=63% |
| 0.500958 | 1.153187 | 0.353464 | 1 | 1 | 7774 | tags=71%, list=33%, signal=106% |
| 0.520322 | 1.153089 | 0.325714 | 1 | 1 | 5875 | tags=58%, list=25%, signal=77% |
| 0.484088 | 1.152804 | 0.345631 | 1 | 1 | 7559 | tags=69%, list=32%, signal=102% |
| 0.490847 | 1.152658 | 0.403738 | 1 | 1 | 4811 | tags=49%, list=21%, signal=62% |
| 0.410948 | 1.152334 | 0.246777 | 1 | 1 | 5426 | tags=48%, list=23%, signal=62% |
| 0.694712 | 1.152265 | 0.382692 | 1 | 1 | 5552 | tags=78%, list=24%, signal=102% |
| 0.518393 | 1.152193 | 0.377495 | 1 | 1 | 5246 | tags=58%, list=22%, signal=75% |
| 0.616338 | 1.151848 | 0.377691 | 1 | 1 | 4673 | tags=71%, list=20%, signal=88% |
| 0.34876 | 1.151725 | 0.214724 | 1 | 1 | 5880 | tags=44%, list=25%, signal=58% |
| 0.367725 | 1.151522 | 0.22292 | 1 | 1 | 2726 | tags=30%, list=12%, signal=33% |
| 0.48213 | 1.151265 | 0.325581 | 1 | 1 | 5875 | tags=58%, list=25%, signal=77% |
| 0.404657 | 1.151079 | 0.232278 | 1 | 1 | 2048 | tags=41%, list=9%, signal=45% |

| | | | | | | |
|---|---|---|---|---|---|---|
| 0.350173 | 1.150957 | 0.275387 | 1 | 1 | 4641 | tags=37%, list=20%, signal=46% |
| 0.372561 | 1.150559 | 0.292064 | 1 | 1 | 5703 | tags=50%, list=24%, signal=66% |
| 0.326832 | 1.150247 | 0.195382 | 1 | 1 | 1959 | tags=20%, list=8%, signal=22% |
| 0.622363 | 1.149125 | 0.352294 | 1 | 1 | 5516 | tags=56%, list=24%, signal=73% |
| 0.434707 | 1.148351 | 0.379859 | 1 | 1 | 5962 | tags=57%, list=25%, signal=76% |
| 0.473028 | 1.147823 | 0.308348 | 1 | 1 | 2989 | tags=30%, list=13%, signal=34% |
| 0.295577 | 1.14733 | 0.15505 | 1 | 1 | 4238 | tags=28%, list=18%, signal=34% |
| 0.341811 | 1.147307 | 0.242089 | 1 | 1 | 3833 | tags=32%, list=16%, signal=38% |
| 0.304016 | 1.147272 | 0.247387 | 1 | 1 | 5442 | tags=35%, list=23%, signal=45% |
| 0.370517 | 1.146578 | 0.309603 | 1 | 1 | 6011 | tags=52%, list=26%, signal=69% |
| 0.559375 | 1.146435 | 0.379699 | 1 | 1 | 5861 | tags=60%, list=25%, signal=80% |
| 0.347727 | 1.144914 | 0.239063 | 1 | 1 | 4268 | tags=39%, list=18%, signal=47% |
| 0.299018 | 1.143734 | 0.188406 | 1 | 1 | 4183 | tags=35%, list=18%, signal=42% |
| 0.422921 | 1.143343 | 0.277865 | 1 | 1 | 2902 | tags=38%, list=12%, signal=43% |
| 0.538846 | 1.143153 | 0.36414 | 1 | 1 | 4560 | tags=63%, list=19%, signal=78% |
| 0.579687 | 1.142947 | 0.416507 | 1 | 1 | 7330 | tags=72%, list=31%, signal=105% |
| 0.422977 | 1.142236 | 0.295337 | 1 | 1 | 3419 | tags=29%, list=15%, signal=34% |
| 0.581549 | 1.142078 | 0.422349 | 1 | 1 | 7808 | tags=79%, list=33%, signal=118% |
| 0.425037 | 1.141577 | 0.360485 | 1 | 1 | 5918 | tags=52%, list=25%, signal=69% |
| 0.334312 | 1.140726 | 0.233279 | 1 | 1 | 6134 | tags=44%, list=26%, signal=59% |
| 0.488324 | 1.13976 | 0.32316 | 1 | 1 | 5719 | tags=51%, list=24%, signal=68% |
| 0.541028 | 1.138329 | 0.356164 | 1 | 1 | 8074 | tags=68%, list=34%, signal=103% |
| 0.337328 | 1.137824 | 0.283531 | 1 | 1 | 1766 | tags=23%, list=8%, signal=25% |
| 0.502693 | 1.137111 | 0.411335 | 1 | 1 | 6281 | tags=61%, list=27%, signal=82% |
| 0.351745 | 1.136738 | 0.283972 | 1 | 1 | 5899 | tags=43%, list=25%, signal=58% |
| 0.314568 | 1.136679 | 0.249164 | 1 | 1 | 2676 | tags=20%, list=11%, signal=23% |
| 0.481874 | 1.13652 | 0.355009 | 1 | 1 | 6539 | tags=57%, list=28%, signal=78% |
| 0.481412 | 1.136265 | 0.383636 | 1 | 1 | 6006 | tags=59%, list=26%, signal=79% |
| 0.577185 | 1.136073 | 0.358349 | 1 | 1 | 5571 | tags=57%, list=24%, signal=74% |
| 0.382357 | 1.135963 | 0.370236 | 1 | 1 | 5895 | tags=44%, list=25%, signal=58% |
| 0.53729 | 1.135358 | 0.4 | 1 | 1 | 4952 | tags=57%, list=21%, signal=72% |
| 0.413746 | 1.134798 | 0.362816 | 1 | 1 | 5855 | tags=45%, list=25%, signal=59% |
| 0.41364 | 1.133659 | 0.33211 | 1 | 1 | 5714 | tags=41%, list=24%, signal=54% |
| 0.489158 | 1.133642 | 0.366541 | 1 | 1 | 5026 | tags=41%, list=21%, signal=52% |
| 0.335732 | 1.132679 | 0.229277 | 1 | 1 | 3976 | tags=36%, list=17%, signal=43% |
| 0.395215 | 1.132407 | 0.253012 | 1 | 1 | 5651 | tags=54%, list=24%, signal=71% |
| 0.336262 | 1.131578 | 0.215625 | 1 | 1 | 6539 | tags=43%, list=28%, signal=60% |
| 0.386704 | 1.131309 | 0.298182 | 1 | 1 | 4519 | tags=39%, list=19%, signal=49% |
| 0.465071 | 1.13027 | 0.301109 | 1 | 1 | 5044 | tags=50%, list=22%, signal=64% |
| 0.385191 | 1.13022 | 0.287319 | 1 | 1 | 3415 | tags=38%, list=15%, signal=44% |
| 0.488971 | 1.129887 | 0.318482 | 1 | 1 | 7965 | tags=45%, list=34%, signal=68% |
| 0.546871 | 1.129463 | 0.38716 | 1 | 1 | 6500 | tags=62%, list=28%, signal=85% |
| 0.372695 | 1.128864 | 0.264085 | 1 | 1 | 2413 | tags=21%, list=10%, signal=23% |
| 0.412679 | 1.1288 | 0.344948 | 1 | 1 | 6849 | tags=53%, list=29%, signal=75% |
| 0.492783 | 1.128389 | 0.412587 | 1 | 1 | 7874 | tags=67%, list=34%, signal=100% |
| 0.36733 | 1.128198 | 0.261164 | 1 | 1 | 2015 | tags=27%, list=9%, signal=30% |
| 0.366057 | 1.128164 | 0.226974 | 1 | 1 | 1466 | tags=25%, list=6%, signal=27% |
| 0.356478 | 1.127595 | 0.241265 | 1 | 1 | 2733 | tags=28%, list=12%, signal=32% |
| 0.360763 | 1.127569 | 0.21626 | 1 | 1 | 3177 | tags=34%, list=14%, signal=40% |
| 0.532171 | 1.12667 | 0.427305 | 1 | 1 | 5880 | tags=56%, list=25%, signal=74% |

| | | | | | | |
|---|---|---|---|---|---|---|
| 0.343431 | 1.126585 | 0.239812 | 1 | 1 | 5291 | tags=36%, list=23%, signal=46% |
| 0.343689 | 1.126559 | 0.2832 | 1 | 1 | 4641 | tags=38%, list=20%, signal=47% |
| 0.448868 | 1.12589 | 0.322148 | 1 | 1 | 5932 | tags=61%, list=25%, signal=82% |
| 0.308776 | 1.125588 | 0.250426 | 1 | 1 | 4945 | tags=36%, list=21%, signal=45% |
| 0.347341 | 1.125418 | 0.268489 | 1 | 1 | 6369 | tags=49%, list=27%, signal=68% |
| 0.429303 | 1.124524 | 0.348797 | 1 | 1 | 5952 | tags=63%, list=25%, signal=84% |
| 0.671154 | 1.124439 | 0.3583 | 1 | 1 | 6074 | tags=69%, list=26%, signal=93% |
| 0.354381 | 1.123786 | 0.258752 | 1 | 1 | 5428 | tags=46%, list=23%, signal=60% |
| 0.283413 | 1.123018 | 0.218155 | 1 | 1 | 5540 | tags=34%, list=24%, signal=45% |
| 0.291825 | 1.122872 | 0.172205 | 1 | 1 | 5544 | tags=40%, list=24%, signal=52% |
| 0.393856 | 1.122821 | 0.363793 | 1 | 1 | 4694 | tags=41%, list=20%, signal=51% |
| 0.316749 | 1.121934 | 0.304348 | 1 | 1 | 5426 | tags=40%, list=23%, signal=51% |
| 0.376265 | 1.121914 | 0.280936 | 1 | 1 | 2099 | tags=24%, list=9%, signal=26% |
| 0.493335 | 1.121603 | 0.409449 | 1 | 1 | 5788 | tags=58%, list=25%, signal=77% |
| 0.471102 | 1.120632 | 0.349727 | 1 | 1 | 6539 | tags=57%, list=28%, signal=79% |
| 0.406141 | 1.120197 | 0.268466 | 1 | 1 | 6462 | tags=53%, list=28%, signal=73% |
| 0.426194 | 1.120048 | 0.392857 | 1 | 1 | 5716 | tags=42%, list=24%, signal=56% |
| 0.494233 | 1.119687 | 0.407678 | 1 | 1 | 5428 | tags=57%, list=23%, signal=74% |
| 0.465071 | 1.119519 | 0.328302 | 1 | 1 | 6257 | tags=46%, list=27%, signal=63% |
| 0.447327 | 1.117703 | 0.316742 | 1 | 1 | 4627 | tags=38%, list=20%, signal=47% |
| 0.561691 | 1.115779 | 0.434211 | 1 | 1 | 7437 | tags=67%, list=32%, signal=98% |
| 0.353337 | 1.115538 | 0.285484 | 1 | 1 | 5219 | tags=42%, list=22%, signal=54% |
| 0.416682 | 1.115266 | 0.302491 | 1 | 1 | 2338 | tags=29%, list=10%, signal=32% |
| 0.557209 | 1.115221 | 0.398467 | 1 | 1 | 7453 | tags=72%, list=32%, signal=106% |
| 0.341146 | 1.115128 | 0.303544 | 1 | 1 | 3995 | tags=35%, list=17%, signal=42% |
| 0.557696 | 1.114614 | 0.424242 | 1 | 1 | 7847 | tags=75%, list=34%, signal=113% |
| 0.4815 | 1.114264 | 0.390019 | 1 | 1 | 5020 | tags=45%, list=21%, signal=57% |
| 0.378759 | 1.11363 | 0.357143 | 1 | 1 | 7033 | tags=49%, list=30%, signal=69% |
| 0.468996 | 1.113294 | 0.354955 | 1 | 1 | 5873 | tags=53%, list=25%, signal=71% |
| 0.424698 | 1.112694 | 0.373494 | 1 | 1 | 7792 | tags=55%, list=33%, signal=82% |
| 0.385342 | 1.112367 | 0.296684 | 1 | 1 | 3364 | tags=29%, list=14%, signal=34% |
| 0.392837 | 1.112222 | 0.355401 | 1 | 1 | 3330 | tags=35%, list=14%, signal=40% |
| 0.383097 | 1.111532 | 0.314516 | 1 | 1 | 4683 | tags=44%, list=20%, signal=56% |
| 0.544765 | 1.110977 | 0.451977 | 1 | 1 | 5552 | tags=56%, list=24%, signal=73% |
| 0.471512 | 1.109428 | 0.399635 | 1 | 1 | 5428 | tags=54%, list=23%, signal=70% |
| 0.358621 | 1.109373 | 0.2592 | 1 | 1 | 3415 | tags=34%, list=15%, signal=40% |
| 0.383631 | 1.107238 | 0.340909 | 1 | 1 | 1796 | tags=25%, list=8%, signal=27% |
| 0.445348 | 1.106882 | 0.374101 | 1 | 1 | 7502 | tags=66%, list=32%, signal=96% |
| 0.477409 | 1.106407 | 0.401852 | 1 | 1 | 5788 | tags=58%, list=25%, signal=77% |
| 0.300996 | 1.106201 | 0.281583 | 1 | 1 | 5860 | tags=44%, list=25%, signal=58% |
| 0.362407 | 1.105614 | 0.309859 | 1 | 1 | 5859 | tags=50%, list=25%, signal=67% |
| 0.335563 | 1.105579 | 0.316071 | 1 | 1 | 2145 | tags=21%, list=9%, signal=24% |
| 0.415246 | 1.104441 | 0.428319 | 1 | 1 | 7341 | tags=57%, list=31%, signal=83% |
| 0.319787 | 1.104296 | 0.335593 | 1 | 1 | 5971 | tags=40%, list=25%, signal=53% |
| 0.381886 | 1.103598 | 0.303704 | 1 | 1 | 2142 | tags=30%, list=9%, signal=33% |
| 0.405078 | 1.103468 | 0.329231 | 1 | 1 | 2761 | tags=35%, list=12%, signal=40% |
| 0.495353 | 1.103242 | 0.33279 | 1 | 1 | 1671 | tags=40%, list=7%, signal=43% |
| 0.314306 | 1.102811 | 0.267356 | 1 | 1 | 4074 | tags=36%, list=17%, signal=43% |
| 0.457315 | 1.102623 | 0.32963 | 1 | 1 | 7847 | tags=64%, list=34%, signal=96% |
| 0.304463 | 1.101854 | 0.254786 | 1 | 1 | 5482 | tags=35%, list=23%, signal=46% |

| | | | | | | |
|---|---|---|---|---|---|---|
| 0.37011 | 1.100541 | 0.313916 | 1 | 1 | 4127 | tags=41%, list=18%, signal=50% |
| 0.447815 | 1.100527 | 0.359401 | 1 | 1 | 4630 | tags=38%, list=20%, signal=47% |
| 0.392342 | 1.100308 | 0.378378 | 1 | 1 | 5246 | tags=44%, list=22%, signal=56% |
| 0.423825 | 1.10022 | 0.393258 | 1 | 1 | 5899 | tags=49%, list=25%, signal=65% |
| 0.4348 | 1.100114 | 0.409253 | 1 | 1 | 6291 | tags=56%, list=27%, signal=75% |
| 0.354301 | 1.100113 | 0.345428 | 1 | 1 | 2338 | tags=33%, list=10%, signal=37% |
| 0.37587 | 1.099804 | 0.361624 | 1 | 1 | 4731 | tags=41%, list=20%, signal=50% |
| 0.4729 | 1.099163 | 0.406427 | 1 | 1 | 5719 | tags=57%, list=24%, signal=75% |
| 0.415842 | 1.098556 | 0.357274 | 1 | 1 | 3897 | tags=36%, list=17%, signal=44% |
| 0.305298 | 1.098105 | 0.280587 | 1 | 1 | 4896 | tags=35%, list=21%, signal=44% |
| 0.31581 | 1.097811 | 0.290476 | 1 | 1 | 6412 | tags=46%, list=27%, signal=64% |
| 0.40074 | 1.09668 | 0.32363 | 1 | 1 | 2792 | tags=38%, list=12%, signal=43% |
| 0.369127 | 1.096647 | 0.299342 | 1 | 1 | 3134 | tags=26%, list=13%, signal=30% |
| 0.418298 | 1.096094 | 0.391844 | 1 | 1 | 2972 | tags=29%, list=13%, signal=33% |
| 0.537307 | 1.095837 | 0.379576 | 1 | 1 | 6868 | tags=76%, list=29%, signal=108% |
| 0.294448 | 1.095347 | 0.272464 | 1 | 1 | 5418 | tags=34%, list=23%, signal=44% |
| 0.426683 | 1.0953 | 0.338409 | 1 | 1 | 2813 | tags=28%, list=12%, signal=32% |
| 0.409092 | 1.094987 | 0.404762 | 1 | 1 | 4329 | tags=38%, list=18%, signal=46% |
| 0.415436 | 1.094263 | 0.310236 | 1 | 1 | 5714 | tags=46%, list=24%, signal=61% |
| 0.30822 | 1.093985 | 0.281159 | 1 | 1 | 5715 | tags=35%, list=24%, signal=47% |
| 0.420716 | 1.0935 | 0.361818 | 1 | 1 | 6006 | tags=45%, list=26%, signal=60% |
| 0.498185 | 1.0919 | 0.434535 | 1 | 1 | 5607 | tags=54%, list=24%, signal=70% |
| 0.413494 | 1.091847 | 0.402647 | 1 | 1 | 5313 | tags=43%, list=23%, signal=56% |
| 0.440761 | 1.091777 | 0.360485 | 1 | 1 | 6612 | tags=55%, list=28%, signal=76% |
| 0.308853 | 1.091618 | 0.313433 | 1 | 1 | 2765 | tags=28%, list=12%, signal=32% |
| 0.415669 | 1.091041 | 0.398564 | 1 | 1 | 7204 | tags=56%, list=31%, signal=81% |
| 0.450623 | 1.090731 | 0.382671 | 1 | 1 | 4533 | tags=44%, list=19%, signal=54% |
| 0.397237 | 1.090085 | 0.344762 | 1 | 1 | 5884 | tags=53%, list=25%, signal=71% |
| 0.586693 | 1.089782 | 0.434363 | 1 | 1 | 7437 | tags=72%, list=32%, signal=105% |
| 0.446981 | 1.089274 | 0.366609 | 1 | 1 | 7255 | tags=47%, list=31%, signal=68% |
| 0.397415 | 1.088904 | 0.381679 | 1 | 1 | 6291 | tags=49%, list=27%, signal=66% |
| 0.547246 | 1.088204 | 0.450758 | 1 | 1 | 7865 | tags=71%, list=34%, signal=106% |
| 0.361632 | 1.08767 | 0.340909 | 1 | 1 | 1280 | tags=18%, list=5%, signal=19% |
| 0.357041 | 1.086995 | 0.300614 | 1 | 1 | 3725 | tags=29%, list=16%, signal=34% |
| 0.351718 | 1.086254 | 0.331126 | 1 | 1 | 5909 | tags=46%, list=25%, signal=61% |
| 0.330939 | 1.085494 | 0.324841 | 1 | 1 | 5717 | tags=44%, list=24%, signal=58% |
| 0.54348 | 1.084543 | 0.425183 | 1 | 1 | 6868 | tags=66%, list=29%, signal=93% |
| 0.374966 | 1.083941 | 0.314516 | 1 | 1 | 4494 | tags=40%, list=19%, signal=49% |
| 0.301239 | 1.08353 | 0.341385 | 1 | 1 | 6423 | tags=38%, list=27%, signal=53% |
| 0.444452 | 1.083197 | 0.332149 | 1 | 1 | 1396 | tags=25%, list=6%, signal=27% |
| 0.378978 | 1.082822 | 0.381555 | 1 | 1 | 3419 | tags=27%, list=15%, signal=31% |
| 0.335553 | 1.082738 | 0.311522 | 1 | 1 | 2902 | tags=29%, list=12%, signal=33% |
| 0.374391 | 1.08183 | 0.298555 | 1 | 1 | 3164 | tags=24%, list=14%, signal=28% |
| 0.324532 | 1.081633 | 0.361854 | 1 | 1 | 5915 | tags=42%, list=25%, signal=56% |
| 0.422086 | 1.08145 | 0.393414 | 1 | 1 | 2479 | tags=29%, list=11%, signal=33% |
| 0.401949 | 1.081184 | 0.420792 | 1 | 1 | 473 | tags=16%, list=2%, signal=16% |
| 0.368088 | 1.080802 | 0.342857 | 1 | 1 | 1313 | tags=24%, list=6%, signal=26% |
| 0.484053 | 1.080739 | 0.464491 | 1 | 1 | 7452 | tags=64%, list=32%, signal=94% |
| 0.472348 | 1.080598 | 0.415441 | 1 | 1 | 5428 | tags=57%, list=23%, signal=74% |
| 0.338383 | 1.080366 | 0.332797 | 1 | 1 | 5584 | tags=42%, list=24%, signal=55% |

| | | | | | | |
|---|---|---|---|---|---|---|
| 0.418538 | 1.079854 | 0.358209 | 1 | 1 | 5872 | tags=48%, list=25%, signal=64% |
| 0.384745 | 1.079206 | 0.335453 | 1 | 1 | 5465 | tags=38%, list=23%, signal=50% |
| 0.307062 | 1.07902 | 0.279699 | 1 | 1 | 1898 | tags=23%, list=8%, signal=25% |
| 0.397684 | 1.078901 | 0.370618 | 1 | 1 | 5860 | tags=46%, list=25%, signal=61% |
| 0.31564 | 1.078598 | 0.304821 | 1 | 1 | 1581 | tags=22%, list=7%, signal=24% |
| 0.411382 | 1.078482 | 0.384083 | 1 | 1 | 2812 | tags=35%, list=12%, signal=40% |
| 0.354857 | 1.078053 | 0.311295 | 1 | 1 | 4573 | tags=33%, list=20%, signal=41% |
| 0.414948 | 1.077661 | 0.349291 | 1 | 1 | 9034 | tags=74%, list=39%, signal=120% |
| 0.333358 | 1.077527 | 0.31562 | 1 | 1 | 5941 | tags=42%, list=25%, signal=57% |
| 0.431063 | 1.077182 | 0.394654 | 1 | 1 | 5504 | tags=52%, list=24%, signal=68% |
| 0.354739 | 1.077112 | 0.359168 | 1 | 1 | 4706 | tags=33%, list=20%, signal=40% |
| 0.43067 | 1.076878 | 0.441989 | 1 | 1 | 5834 | tags=58%, list=25%, signal=77% |
| 0.359323 | 1.076604 | 0.322581 | 1 | 1 | 2692 | tags=35%, list=11%, signal=39% |
| 0.416658 | 1.076382 | 0.427562 | 1 | 1 | 5943 | tags=50%, list=25%, signal=67% |
| 0.293323 | 1.075535 | 0.305707 | 1 | 1 | 4953 | tags=37%, list=21%, signal=47% |
| 0.411003 | 1.075384 | 0.40884 | 1 | 1 | 5590 | tags=50%, list=24%, signal=65% |
| 0.313511 | 1.075344 | 0.328947 | 1 | 1 | 5796 | tags=39%, list=25%, signal=52% |
| 0.443118 | 1.075185 | 0.411559 | 1 | 1 | 5613 | tags=54%, list=24%, signal=71% |
| 0.419747 | 1.073459 | 0.396797 | 1 | 1 | 5352 | tags=42%, list=23%, signal=54% |
| 0.402805 | 1.073335 | 0.396022 | 1 | 1 | 5525 | tags=47%, list=24%, signal=62% |
| 0.588659 | 1.073014 | 0.478599 | 1 | 1 | 5861 | tags=59%, list=25%, signal=78% |
| 0.385889 | 1.072916 | 0.389073 | 1 | 1 | 5580 | tags=44%, list=24%, signal=58% |
| 0.317777 | 1.072821 | 0.326087 | 1 | 1 | 5933 | tags=40%, list=25%, signal=54% |
| 0.427037 | 1.072281 | 0.368507 | 1 | 1 | 7323 | tags=58%, list=31%, signal=84% |
| 0.390048 | 1.071995 | 0.344291 | 1 | 1 | 5483 | tags=53%, list=23%, signal=70% |
| 0.365921 | 1.071961 | 0.340807 | 1 | 1 | 1681 | tags=29%, list=7%, signal=31% |
| 0.484232 | 1.071842 | 0.436191 | 1 | 1 | 4926 | tags=58%, list=21%, signal=73% |
| 0.408945 | 1.070927 | 0.391304 | 1 | 1 | 5723 | tags=51%, list=24%, signal=67% |
| 0.462144 | 1.070851 | 0.412758 | 1 | 1 | 1071 | tags=29%, list=5%, signal=31% |
| 0.417838 | 1.070701 | 0.373188 | 1 | 1 | 6866 | tags=53%, list=29%, signal=75% |
| 0.454191 | 1.070613 | 0.378151 | 1 | 1 | 5245 | tags=53%, list=22%, signal=68% |
| 0.427336 | 1.069783 | 0.434856 | 1 | 1 | 7275 | tags=63%, list=31%, signal=91% |
| 0.512295 | 1.069638 | 0.431127 | 1 | 1 | 5880 | tags=56%, list=25%, signal=74% |
| 0.368144 | 1.069268 | 0.426386 | 1 | 1 | 4560 | tags=37%, list=19%, signal=46% |
| 0.453674 | 1.069165 | 0.394393 | 1 | 1 | 5956 | tags=52%, list=25%, signal=70% |
| 0.476588 | 1.069101 | 0.459459 | 1 | 1 | 7858 | tags=71%, list=34%, signal=107% |
| 0.489921 | 1.068841 | 0.424581 | 1 | 1 | 7147 | tags=69%, list=31%, signal=99% |
| 0.418725 | 1.067864 | 0.398998 | 1 | 1 | 6866 | tags=61%, list=29%, signal=86% |
| 0.350164 | 1.067016 | 0.315024 | 1 | 1 | 2381 | tags=26%, list=10%, signal=29% |
| 0.435686 | 1.066976 | 0.46223 | 1 | 1 | 5152 | tags=40%, list=22%, signal=51% |
| 0.33872 | 1.06697 | 0.343907 | 1 | 1 | 3254 | tags=27%, list=14%, signal=32% |
| 0.297422 | 1.066835 | 0.330159 | 1 | 1 | 2966 | tags=30%, list=13%, signal=34% |
| 0.31117 | 1.066724 | 0.33042 | 1 | 1 | 3759 | tags=31%, list=16%, signal=37% |
| 0.300568 | 1.066622 | 0.345178 | 1 | 1 | 5796 | tags=33%, list=25%, signal=44% |
| 0.2878 | 1.066257 | 0.333333 | 1 | 1 | 3610 | tags=27%, list=15%, signal=32% |
| 0.379252 | 1.066189 | 0.397554 | 1 | 1 | 5714 | tags=52%, list=24%, signal=69% |
| 0.547556 | 1.066033 | 0.439771 | 1 | 1 | 8050 | tags=71%, list=34%, signal=108% |
| 0.330299 | 1.065298 | 0.402337 | 1 | 1 | 2597 | tags=26%, list=11%, signal=29% |
| 0.367996 | 1.065146 | 0.424908 | 1 | 1 | 4351 | tags=40%, list=19%, signal=49% |
| 0.414914 | 1.064305 | 0.364452 | 1 | 1 | 4273 | tags=50%, list=18%, signal=61% |

| | | | | | | |
|---|---|---|---|---|---|---|
| 0.366998 | 1.064125 | 0.335799 | 1 | 1 | 6234 | tags=52%, list=27%, signal=70% |
| 0.356546 | 1.063901 | 0.35625 | 1 | 1 | 3688 | tags=35%, list=16%, signal=42% |
| 0.365901 | 1.063606 | 0.440433 | 1 | 1 | 5208 | tags=36%, list=22%, signal=46% |
| 0.328307 | 1.062993 | 0.321377 | 1 | 1 | 2932 | tags=29%, list=13%, signal=34% |
| 0.394817 | 1.062756 | 0.37702 | 1 | 1 | 1116 | tags=32%, list=5%, signal=33% |
| 0.359716 | 1.062748 | 0.386023 | 1 | 1 | 3730 | tags=42%, list=16%, signal=50% |
| 0.396144 | 1.062629 | 0.410546 | 1 | 1 | 5324 | tags=46%, list=23%, signal=59% |
| 0.409195 | 1.061583 | 0.426497 | 1 | 1 | 5651 | tags=51%, list=24%, signal=67% |
| 0.347626 | 1.061425 | 0.396197 | 1 | 1 | 2738 | tags=31%, list=12%, signal=35% |
| 0.351086 | 1.06036 | 0.360555 | 1 | 1 | 2902 | tags=26%, list=12%, signal=30% |
| 0.363768 | 1.060015 | 0.437736 | 1 | 1 | 5835 | tags=44%, list=25%, signal=58% |
| 0.297678 | 1.059731 | 0.331316 | 1 | 1 | 3105 | tags=29%, list=13%, signal=33% |
| 0.455076 | 1.059081 | 0.401887 | 1 | 1 | 4694 | tags=55%, list=20%, signal=69% |
| 0.309899 | 1.058806 | 0.390119 | 1 | 1 | 4853 | tags=35%, list=21%, signal=44% |
| 0.454962 | 1.058738 | 0.434783 | 1 | 1 | 5788 | tags=59%, list=25%, signal=79% |
| 0.40543 | 1.058567 | 0.441392 | 1 | 1 | 5719 | tags=39%, list=24%, signal=51% |
| 0.460751 | 1.05852 | 0.456044 | 1 | 1 | 5930 | tags=53%, list=25%, signal=70% |
| 0.383363 | 1.057146 | 0.399648 | 1 | 1 | 2710 | tags=33%, list=12%, signal=37% |
| 0.319735 | 1.056809 | 0.35 | 1 | 1 | 5491 | tags=42%, list=23%, signal=55% |
| 0.350114 | 1.056719 | 0.362903 | 1 | 1 | 2813 | tags=27%, list=12%, signal=31% |
| 0.407349 | 1.056347 | 0.363782 | 1 | 1 | 6655 | tags=62%, list=28%, signal=87% |
| 0.311121 | 1.054603 | 0.341632 | 1 | 1 | 5020 | tags=42%, list=21%, signal=53% |
| 0.400433 | 1.053838 | 0.419411 | 1 | 1 | 6306 | tags=44%, list=27%, signal=60% |
| 0.484533 | 1.053752 | 0.423759 | 1 | 1 | 5880 | tags=48%, list=25%, signal=64% |
| 0.387149 | 1.053648 | 0.4304 | 1 | 1 | 2099 | tags=31%, list=9%, signal=34% |
| 0.562084 | 1.052491 | 0.448405 | 1 | 1 | 7019 | tags=65%, list=30%, signal=92% |
| 0.306583 | 1.051654 | 0.411003 | 1 | 1 | 5283 | tags=40%, list=23%, signal=51% |
| 0.292704 | 1.050986 | 0.346045 | 1 | 1 | 4514 | tags=35%, list=19%, signal=43% |
| 0.321146 | 1.050304 | 0.39469 | 1 | 1 | 2506 | tags=26%, list=11%, signal=30% |
| 0.319029 | 1.049977 | 0.368976 | 1 | 1 | 4704 | tags=43%, list=20%, signal=54% |
| 0.294567 | 1.048486 | 0.376033 | 1 | 1 | 5857 | tags=52%, list=25%, signal=70% |
| 0.262153 | 1.048369 | 0.332842 | 1 | 1 | 5540 | tags=35%, list=24%, signal=45% |
| 0.322525 | 1.0479 | 0.383178 | 1 | 1 | 4170 | tags=25%, list=18%, signal=30% |
| 0.334625 | 1.047173 | 0.349711 | 1 | 1 | 3403 | tags=32%, list=15%, signal=38% |
| 0.338759 | 1.046798 | 0.392157 | 1 | 1 | 6897 | tags=54%, list=29%, signal=77% |
| 0.372771 | 1.046512 | 0.389351 | 1 | 1 | 9034 | tags=63%, list=39%, signal=102% |
| 0.409174 | 1.046064 | 0.434109 | 1 | 1 | 7358 | tags=56%, list=31%, signal=82% |
| 0.373509 | 1.046043 | 0.383607 | 1 | 1 | 4457 | tags=41%, list=19%, signal=51% |
| 0.380142 | 1.045267 | 0.468966 | 1 | 1 | 5682 | tags=46%, list=24%, signal=60% |
| 0.358727 | 1.045057 | 0.421409 | 1 | 1 | 4627 | tags=39%, list=20%, signal=49% |
| 0.403783 | 1.044729 | 0.453782 | 1 | 1 | 6114 | tags=59%, list=26%, signal=80% |
| 0.258351 | 1.043858 | 0.326877 | 1 | 1 | 3777 | tags=28%, list=16%, signal=33% |
| 0.400287 | 1.040568 | 0.389134 | 1 | 1 | 2600 | tags=40%, list=11%, signal=45% |
| 0.479199 | 1.040459 | 0.434866 | 1 | 1 | 7046 | tags=57%, list=30%, signal=81% |
| 0.473476 | 1.040097 | 0.489796 | 1 | 1 | 4926 | tags=57%, list=21%, signal=72% |
| 0.266991 | 1.039382 | 0.335756 | 1 | 1 | 4775 | tags=35%, list=20%, signal=43% |
| 0.295086 | 1.038991 | 0.389231 | 1 | 1 | 4278 | tags=28%, list=18%, signal=34% |
| 0.300912 | 1.038368 | 0.393189 | 1 | 1 | 3478 | tags=36%, list=15%, signal=42% |
| 0.33153 | 1.037278 | 0.432039 | 1 | 1 | 3352 | tags=37%, list=14%, signal=43% |
| 0.346631 | 1.036639 | 0.438061 | 1 | 1 | 5378 | tags=40%, list=23%, signal=51% |

| | | | | | | |
|---|---|---|---|---|---|---|
| 0.461952 | 1.03653 | 0.433645 | 1 | 1 | 5656 | tags=56%, list=24%, signal=73% |
| 0.377582 | 1.036344 | 0.425159 | 1 | 1 | 6316 | tags=55%, list=27%, signal=76% |
| 0.314839 | 1.036204 | 0.415842 | 1 | 1 | 2748 | tags=30%, list=12%, signal=34% |
| 0.290117 | 1.035672 | 0.390625 | 1 | 1 | 3035 | tags=30%, list=13%, signal=34% |
| 0.374484 | 1.035412 | 0.413194 | 1 | 1 | 917 | tags=27%, list=4%, signal=28% |
| 0.373173 | 1.035365 | 0.451043 | 1 | 1 | 1564 | tags=29%, list=7%, signal=31% |
| 0.347496 | 1.035138 | 0.463972 | 1 | 1 | 5329 | tags=43%, list=23%, signal=56% |
| 0.293907 | 1.034781 | 0.38307 | 1 | 1 | 5459 | tags=40%, list=23%, signal=52% |
| 0.469013 | 1.034635 | 0.435233 | 1 | 1 | 5673 | tags=48%, list=24%, signal=63% |
| 0.305085 | 1.034103 | 0.405899 | 1 | 1 | 4187 | tags=31%, list=18%, signal=37% |
| 0.452572 | 1.033946 | 0.401254 | 1 | 1 | 4068 | tags=56%, list=17%, signal=67% |
| 0.390697 | 1.033846 | 0.421442 | 1 | 1 | 3944 | tags=35%, list=17%, signal=42% |
| 0.411459 | 1.033448 | 0.463878 | 1 | 1 | 6010 | tags=50%, list=26%, signal=67% |
| 0.334004 | 1.033427 | 0.431062 | 1 | 1 | 3051 | tags=26%, list=13%, signal=30% |
| 0.331934 | 1.033025 | 0.399713 | 1 | 1 | 5522 | tags=40%, list=24%, signal=52% |
| 0.345712 | 1.032571 | 0.380719 | 1 | 1 | 4737 | tags=37%, list=20%, signal=46% |
| 0.328264 | 1.032351 | 0.385037 | 1 | 1 | 5790 | tags=37%, list=25%, signal=49% |
| 0.344261 | 1.031286 | 0.42978 | 1 | 1 | 5555 | tags=40%, list=24%, signal=53% |
| 0.334231 | 1.030393 | 0.447735 | 1 | 1 | 5445 | tags=39%, list=23%, signal=51% |
| 0.365194 | 1.030299 | 0.440536 | 1 | 1 | 4154 | tags=38%, list=18%, signal=46% |
| 0.371852 | 1.029949 | 0.40177 | 1 | 1 | 6291 | tags=47%, list=27%, signal=64% |
| 0.354349 | 1.029754 | 0.410959 | 1 | 1 | 4332 | tags=32%, list=18%, signal=39% |
| 0.371867 | 1.029706 | 0.398637 | 1 | 1 | 1844 | tags=29%, list=8%, signal=31% |
| 0.379126 | 1.029396 | 0.407609 | 1 | 1 | 4640 | tags=41%, list=20%, signal=50% |
| 0.257842 | 1.029205 | 0.405121 | 1 | 1 | 5540 | tags=32%, list=24%, signal=41% |
| 0.305872 | 1.028884 | 0.372488 | 1 | 1 | 6370 | tags=44%, list=27%, signal=60% |
| 0.351071 | 1.028381 | 0.427711 | 1 | 1 | 6304 | tags=46%, list=27%, signal=63% |
| 0.441118 | 1.027807 | 0.453532 | 1 | 1 | 2676 | tags=36%, list=11%, signal=41% |
| 0.297228 | 1.027798 | 0.483051 | 1 | 1 | 5859 | tags=44%, list=25%, signal=58% |
| 0.360449 | 1.027364 | 0.435154 | 1 | 1 | 4511 | tags=39%, list=19%, signal=49% |
| 0.3004 | 1.027038 | 0.427184 | 1 | 1 | 2612 | tags=25%, list=11%, signal=28% |
| 0.428361 | 1.026784 | 0.432971 | 1 | 1 | 6730 | tags=64%, list=29%, signal=89% |
| 0.30212 | 1.026276 | 0.406926 | 1 | 1 | 3366 | tags=25%, list=14%, signal=29% |
| 0.274486 | 1.026158 | 0.4304 | 1 | 1 | 3949 | tags=29%, list=17%, signal=35% |
| 0.35955 | 1.025709 | 0.419753 | 1 | 1 | 2816 | tags=31%, list=12%, signal=35% |
| 0.290205 | 1.025689 | 0.462633 | 1 | 1 | 5729 | tags=37%, list=24%, signal=48% |
| 0.293819 | 1.025132 | 0.403175 | 1 | 1 | 5882 | tags=42%, list=25%, signal=56% |
| 0.460879 | 1.02483 | 0.455535 | 1 | 1 | 7437 | tags=67%, list=32%, signal=97% |
| 0.401412 | 1.02469 | 0.485086 | 1 | 1 | 6214 | tags=50%, list=27%, signal=68% |
| 0.311307 | 1.024625 | 0.457143 | 1 | 1 | 1255 | tags=17%, list=5%, signal=18% |
| 0.36236 | 1.02436 | 0.437398 | 1 | 1 | 1829 | tags=21%, list=8%, signal=23% |
| 0.260092 | 1.023495 | 0.430894 | 1 | 1 | 4193 | tags=30%, list=18%, signal=36% |
| 0.365151 | 1.023142 | 0.423895 | 1 | 1 | 3072 | tags=39%, list=13%, signal=45% |
| 0.28716 | 1.02301 | 0.404946 | 1 | 1 | 4633 | tags=35%, list=20%, signal=43% |
| 0.291653 | 1.022475 | 0.443038 | 1 | 1 | 4628 | tags=35%, list=20%, signal=43% |
| 0.297487 | 1.022186 | 0.384071 | 1 | 1 | 4960 | tags=36%, list=21%, signal=45% |
| 0.516057 | 1.021433 | 0.474088 | 1 | 1 | 7858 | tags=80%, list=34%, signal=120% |
| 0.297814 | 1.0212 | 0.471025 | 1 | 1 | 4773 | tags=36%, list=20%, signal=45% |
| 0.263467 | 1.020896 | 0.470499 | 1 | 1 | 3276 | tags=25%, list=14%, signal=29% |
| 0.421085 | 1.020589 | 0.446203 | 1 | 1 | 5644 | tags=53%, list=24%, signal=70% |

| | | | | | | |
|---|---|---|---|---|---|---|
| 0.353291 | 1.020499 | 0.410882 | 1 | 1 | 5952 | tags=44%, list=25%, signal=58% |
| 0.545205 | 1.020354 | 0.472119 | 1 | 1 | 8050 | tags=63%, list=34%, signal=96% |
| 0.387008 | 1.020338 | 0.466443 | 1 | 1 | 5895 | tags=49%, list=25%, signal=65% |
| 0.351869 | 1.019212 | 0.42 | 1 | 1 | 3918 | tags=35%, list=17%, signal=42% |
| 0.336724 | 1.018753 | 0.418519 | 1 | 1 | 4456 | tags=37%, list=19%, signal=46% |
| 0.260725 | 1.018685 | 0.402985 | 1 | 1 | 4217 | tags=25%, list=18%, signal=31% |
| 0.276546 | 1.018591 | 0.372828 | 1 | 1 | 4048 | tags=28%, list=17%, signal=34% |
| 0.33749 | 1.018335 | 0.433172 | 1 | 1 | 5044 | tags=50%, list=22%, signal=64% |
| 0.34139 | 1.018263 | 0.426104 | 1 | 1 | 4332 | tags=32%, list=18%, signal=39% |
| 0.428228 | 1.017721 | 0.464143 | 1 | 1 | 5312 | tags=56%, list=23%, signal=73% |
| 0.379979 | 1.01729 | 0.426288 | 1 | 1 | 1696 | tags=24%, list=7%, signal=26% |
| 0.309846 | 1.017258 | 0.427419 | 1 | 1 | 6082 | tags=51%, list=26%, signal=69% |
| 0.390172 | 1.01723 | 0.47193 | 1 | 1 | 4746 | tags=30%, list=20%, signal=38% |
| 0.315249 | 1.016961 | 0.40137 | 1 | 1 | 4978 | tags=40%, list=21%, signal=51% |
| 0.302832 | 1.016885 | 0.434535 | 1 | 1 | 3822 | tags=27%, list=16%, signal=32% |
| 0.483181 | 1.016289 | 0.510989 | 1 | 1 | 5551 | tags=52%, list=24%, signal=68% |
| 0.271218 | 1.015665 | 0.383046 | 1 | 1 | 4659 | tags=34%, list=20%, signal=42% |
| 0.419775 | 1.014104 | 0.436149 | 1 | 1 | 3631 | tags=35%, list=16%, signal=42% |
| 0.289665 | 1.014046 | 0.427019 | 1 | 1 | 6031 | tags=46%, list=26%, signal=62% |
| 0.248814 | 1.013881 | 0.459016 | 1 | 1 | 4511 | tags=33%, list=19%, signal=40% |
| 0.504348 | 1.013768 | 0.500956 | 1 | 1 | 7858 | tags=73%, list=34%, signal=109% |
| 0.415891 | 1.01365 | 0.455988 | 1 | 1 | 5671 | tags=42%, list=24%, signal=56% |
| 0.454859 | 1.013179 | 0.480519 | 1 | 1 | 7892 | tags=65%, list=34%, signal=97% |
| 0.322356 | 1.013174 | 0.414802 | 1 | 1 | 2249 | tags=26%, list=10%, signal=29% |
| 0.286644 | 1.012658 | 0.455099 | 1 | 1 | 5901 | tags=38%, list=25%, signal=50% |
| 0.290555 | 1.012492 | 0.438597 | 1 | 1 | 4509 | tags=28%, list=19%, signal=35% |
| 0.318364 | 1.012077 | 0.42576 | 1 | 1 | 4367 | tags=24%, list=19%, signal=29% |
| 0.42598 | 1.012049 | 0.490725 | 1 | 1 | 6022 | tags=50%, list=26%, signal=67% |
| 0.382673 | 1.011025 | 0.461268 | 1 | 1 | 5044 | tags=45%, list=22%, signal=58% |
| 0.279211 | 1.010827 | 0.441997 | 1 | 1 | 4271 | tags=30%, list=18%, signal=37% |
| 0.425477 | 1.010619 | 0.463221 | 1 | 1 | 7499 | tags=53%, list=32%, signal=78% |
| 0.246813 | 1.01022 | 0.45706 | 1 | 1 | 4727 | tags=32%, list=20%, signal=39% |
| 0.313243 | 1.010185 | 0.447552 | 1 | 1 | 6866 | tags=48%, list=29%, signal=68% |
| 0.284274 | 1.010035 | 0.442623 | 1 | 1 | 5925 | tags=42%, list=25%, signal=55% |
| 0.331452 | 1.009639 | 0.469218 | 1 | 1 | 5809 | tags=36%, list=25%, signal=48% |
| 0.39562 | 1.009512 | 0.474441 | 1 | 1 | 3500 | tags=27%, list=15%, signal=32% |
| 0.409597 | 1.009334 | 0.458633 | 1 | 1 | 7970 | tags=62%, list=34%, signal=94% |
| 0.409916 | 1.009165 | 0.454717 | 1 | 1 | 7147 | tags=59%, list=31%, signal=84% |
| 0.302705 | 1.009093 | 0.422059 | 1 | 1 | 626 | tags=15%, list=3%, signal=15% |
| 0.403084 | 1.008493 | 0.451078 | 1 | 1 | 2948 | tags=33%, list=13%, signal=38% |
| 0.496697 | 1.008327 | 0.496255 | 1 | 1 | 5353 | tags=48%, list=23%, signal=61% |
| 0.305701 | 1.00808 | 0.480865 | 1 | 1 | 2172 | tags=22%, list=9%, signal=24% |
| 0.37282 | 1.00777 | 0.469194 | 0.999578 | 1 | 2700 | tags=35%, list=12%, signal=40% |
| 0.352695 | 1.007768 | 0.476692 | 0.998035 | 1 | 5831 | tags=48%, list=25%, signal=64% |
| 0.350349 | 1.007131 | 0.435556 | 0.998703 | 1 | 4520 | tags=43%, list=19%, signal=53% |
| 0.300129 | 1.007062 | 0.424354 | 0.997362 | 1 | 3993 | tags=30%, list=17%, signal=35% |
| 0.347087 | 1.006295 | 0.457143 | 0.998368 | 1 | 2761 | tags=21%, list=12%, signal=23% |
| 0.299289 | 1.005945 | 0.460614 | 0.998009 | 1 | 2409 | tags=22%, list=10%, signal=25% |
| 0.350023 | 1.004652 | 0.483553 | 1 | 1 | 3633 | tags=27%, list=16%, signal=32% |
| 0.33705 | 1.003903 | 0.46568 | 1 | 1 | 6218 | tags=46%, list=27%, signal=62% |

| | | | | | | |
|---|---|---|---|---|---|---|
| 0.369065 | 1.003796 | 0.440758 | 1 | 1 | 3567 | tags=38%, list=15%, signal=45% |
| 0.407223 | 1.003752 | 0.47099 | 0.998957 | 1 | 5530 | tags=48%, list=24%, signal=63% |
| 0.380549 | 1.002834 | 0.433962 | 1 | 1 | 5937 | tags=48%, list=25%, signal=64% |
| 0.34721 | 1.00235 | 0.471204 | 1 | 1 | 1094 | tags=13%, list=5%, signal=13% |
| 0.283521 | 1.002178 | 0.482143 | 0.999482 | 1 | 4144 | tags=28%, list=18%, signal=34% |
| 0.462098 | 1.002109 | 0.489362 | 0.998152 | 1 | 8098 | tags=69%, list=35%, signal=106% |
| 0.274115 | 1.00189 | 0.44152 | 0.997288 | 1 | 5671 | tags=39%, list=24%, signal=52% |
| 0.354146 | 1.001657 | 0.488688 | 0.996483 | 1 | 4770 | tags=38%, list=20%, signal=47% |
| 0.281138 | 1.001571 | 0.521886 | 0.99527 | 1 | 4643 | tags=31%, list=20%, signal=38% |
| 0.270037 | 1.001411 | 0.476268 | 0.994278 | 1 | 3495 | tags=28%, list=15%, signal=32% |
| 0.402221 | 0.999678 | 0.530097 | 0.998037 | 1 | 6282 | tags=35%, list=27%, signal=47% |
| 0.356738 | 0.999646 | 0.454254 | 0.996626 | 1 | 4081 | tags=35%, list=17%, signal=42% |
| 0.30394 | 0.998983 | 0.427083 | 0.997321 | 1 | 5882 | tags=42%, list=25%, signal=56% |
| 0.452201 | 0.998723 | 0.518304 | 0.996624 | 1 | 5962 | tags=56%, list=25%, signal=74% |
| 0.35964 | 0.99824 | 0.445098 | 0.996556 | 1 | 4388 | tags=33%, list=19%, signal=41% |
| 0.281782 | 0.997939 | 0.448175 | 0.995957 | 1 | 7324 | tags=48%, list=31%, signal=69% |
| 0.343196 | 0.997811 | 0.440177 | 0.99484 | 1 | 2996 | tags=32%, list=13%, signal=37% |
| 0.440107 | 0.997584 | 0.511236 | 0.994104 | 1 | 4802 | tags=54%, list=21%, signal=68% |
| 0.319536 | 0.997049 | 0.45283 | 0.99423 | 1 | 2913 | tags=32%, list=12%, signal=36% |
| 0.338124 | 0.996735 | 0.498141 | 0.993767 | 1 | 1037 | tags=21%, list=4%, signal=22% |
| 0.325324 | 0.996732 | 0.443995 | 0.992305 | 1 | 5906 | tags=40%, list=25%, signal=54% |
| 0.423454 | 0.996404 | 0.507576 | 0.992042 | 1 | 7335 | tags=59%, list=31%, signal=86% |
| 0.294043 | 0.996344 | 0.492832 | 0.990702 | 1 | 5865 | tags=34%, list=25%, signal=45% |
| 0.364289 | 0.994849 | 0.473384 | 0.993915 | 1 | 5324 | tags=38%, list=23%, signal=48% |
| 0.345717 | 0.994384 | 0.484375 | 0.993929 | 1 | 5803 | tags=38%, list=25%, signal=51% |
| 0.294242 | 0.993424 | 0.501385 | 0.995433 | 1 | 3488 | tags=29%, list=15%, signal=34% |
| 0.283368 | 0.99331 | 0.444785 | 0.994306 | 1 | 5474 | tags=41%, list=23%, signal=52% |
| 0.378516 | 0.99323 | 0.430451 | 0.993059 | 1 | 8065 | tags=61%, list=34%, signal=92% |
| 0.251384 | 0.993112 | 0.473227 | 0.991981 | 1 | 3579 | tags=24%, list=15%, signal=28% |
| 0.433236 | 0.993066 | 0.472546 | 0.990651 | 1 | 4358 | tags=50%, list=19%, signal=61% |
| 0.476286 | 0.993035 | 0.483696 | 0.9893 | 1 | 3944 | tags=43%, list=17%, signal=51% |
| 0.409893 | 0.992981 | 0.452007 | 0.987989 | 1 | 5558 | tags=51%, list=24%, signal=67% |
| 0.307555 | 0.992977 | 0.481422 | 0.98655 | 1 | 6023 | tags=41%, list=26%, signal=55% |
| 0.385611 | 0.992912 | 0.423645 | 0.985285 | 1 | 5283 | tags=45%, list=23%, signal=58% |
| 0.254892 | 0.992749 | 0.415435 | 0.984353 | 1 | 2920 | tags=19%, list=12%, signal=22% |
| 0.270547 | 0.992426 | 0.474662 | 0.983859 | 1 | 4556 | tags=27%, list=19%, signal=33% |
| 0.300553 | 0.991592 | 0.510938 | 0.984998 | 1 | 3276 | tags=26%, list=14%, signal=30% |
| 0.31849 | 0.991583 | 0.468582 | 0.9836 | 1 | 3978 | tags=33%, list=17%, signal=39% |
| 0.298272 | 0.990529 | 0.496644 | 0.985614 | 1 | 5533 | tags=36%, list=24%, signal=47% |
| 0.290931 | 0.989956 | 0.50487 | 0.98603 | 1 | 5882 | tags=38%, list=25%, signal=50% |
| 0.413489 | 0.989741 | 0.50099 | 0.98524 | 1 | 6010 | tags=50%, list=26%, signal=66% |
| 0.344332 | 0.98932 | 0.44702 | 0.984992 | 1 | 4378 | tags=32%, list=19%, signal=40% |
| 0.392355 | 0.988988 | 0.493827 | 0.984712 | 1 | 6392 | tags=57%, list=27%, signal=78% |
| 0.343103 | 0.988856 | 0.490909 | 0.983661 | 1 | 2099 | tags=24%, list=9%, signal=26% |
| 0.330306 | 0.988411 | 0.433099 | 0.983591 | 1 | 4008 | tags=29%, list=17%, signal=34% |
| 0.334863 | 0.988301 | 0.481766 | 0.982521 | 1 | 5590 | tags=39%, list=24%, signal=51% |
| 0.262176 | 0.987991 | 0.478708 | 0.982089 | 1 | 2785 | tags=23%, list=12%, signal=26% |
| 0.379456 | 0.98795 | 0.49913 | 0.980785 | 1 | 7439 | tags=43%, list=32%, signal=63% |
| 0.530674 | 0.987872 | 0.61062 | 0.97958 | 1 | 6699 | tags=47%, list=29%, signal=66% |
| 0.320906 | 0.987661 | 0.462366 | 0.978795 | 1 | 3721 | tags=37%, list=16%, signal=44% |

| | | | | | | |
|---|---|---|---|---|---|---|
| 0.45504 | 0.987016 | 0.518382 | 0.979388 | 1 | 5564 | tags=53%, list=24%, signal=69% |
| 0.244991 | 0.986565 | 0.490446 | 0.979418 | 1 | 3225 | tags=22%, list=14%, signal=25% |
| 0.261512 | 0.986444 | 0.522124 | 0.978392 | 1 | 4863 | tags=30%, list=21%, signal=37% |
| 0.355023 | 0.986358 | 0.512368 | 0.977366 | 1 | 3945 | tags=44%, list=17%, signal=53% |
| 0.308239 | 0.985929 | 0.482549 | 0.97731 | 1 | 3366 | tags=31%, list=14%, signal=36% |
| 0.254851 | 0.985786 | 0.450464 | 0.976352 | 1 | 5925 | tags=37%, list=25%, signal=49% |
| 0.262462 | 0.985513 | 0.537748 | 0.975835 | 1 | 4127 | tags=30%, list=18%, signal=36% |
| 0.275437 | 0.985216 | 0.424497 | 0.975278 | 1 | 4694 | tags=32%, list=20%, signal=40% |
| 0.347684 | 0.985196 | 0.440959 | 0.973946 | 1 | 5701 | tags=41%, list=24%, signal=53% |
| 0.313958 | 0.985185 | 0.447109 | 0.972604 | 1 | 5595 | tags=42%, list=24%, signal=55% |
| 0.416393 | 0.98506 | 0.438938 | 0.971634 | 1 | 3638 | tags=39%, list=16%, signal=46% |
| 0.293433 | 0.984674 | 0.533646 | 0.971327 | 1 | 2506 | tags=25%, list=11%, signal=28% |
| 0.35674 | 0.984132 | 0.55719 | 0.971486 | 1 | 5762 | tags=50%, list=25%, signal=66% |
| 0.363073 | 0.983295 | 0.461661 | 0.972589 | 1 | 3288 | tags=29%, list=14%, signal=34% |
| 0.315795 | 0.983059 | 0.445765 | 0.971919 | 1 | 1898 | tags=19%, list=8%, signal=21% |
| 0.299695 | 0.98304 | 0.467456 | 0.970604 | 1 | 4892 | tags=39%, list=21%, signal=49% |
| 0.479937 | 0.982985 | 0.513812 | 0.969443 | 1 | 5152 | tags=50%, list=22%, signal=64% |
| 0.377299 | 0.982766 | 0.502732 | 0.968713 | 1 | 4365 | tags=39%, list=19%, signal=47% |
| 0.429575 | 0.982585 | 0.50289 | 0.967886 | 1 | 6500 | tags=49%, list=28%, signal=68% |
| 0.378156 | 0.982498 | 0.486903 | 0.966865 | 1 | 1864 | tags=29%, list=8%, signal=31% |
| 0.408531 | 0.982161 | 0.485507 | 0.966583 | 1 | 5834 | tags=56%, list=25%, signal=74% |
| 0.305153 | 0.982008 | 0.51772 | 0.965641 | 1 | 4443 | tags=34%, list=19%, signal=42% |
| 0.244768 | 0.981706 | 0.517241 | 0.965184 | 1 | 4097 | tags=28%, list=17%, signal=33% |
| 0.431606 | 0.981534 | 0.539197 | 0.964314 | 1 | 5003 | tags=43%, list=21%, signal=55% |
| 0.370514 | 0.98118 | 0.460548 | 0.963981 | 1 | 7805 | tags=57%, list=33%, signal=85% |
| 0.24767 | 0.980659 | 0.482659 | 0.964152 | 1 | 4741 | tags=30%, list=20%, signal=37% |
| 0.353673 | 0.98051 | 0.475836 | 0.963231 | 1 | 1281 | tags=24%, list=5%, signal=25% |
| 0.292676 | 0.979899 | 0.479263 | 0.963849 | 1 | 5607 | tags=40%, list=24%, signal=53% |
| 0.396237 | 0.97981 | 0.519504 | 0.962791 | 1 | 4816 | tags=48%, list=21%, signal=60% |
| 0.496835 | 0.97944 | 0.598862 | 0.96258 | 1 | 7508 | tags=83%, list=32%, signal=123% |
| 0.251537 | 0.979178 | 0.551247 | 0.962084 | 1 | 3579 | tags=25%, list=15%, signal=29% |
| 0.428622 | 0.978363 | 0.522769 | 0.963353 | 1 | 5020 | tags=46%, list=21%, signal=59% |
| 0.338684 | 0.977928 | 0.504188 | 0.963405 | 1 | 5095 | tags=47%, list=22%, signal=60% |
| 0.382929 | 0.977702 | 0.511927 | 0.962672 | 1 | 5869 | tags=45%, list=25%, signal=59% |
| 0.234354 | 0.977211 | 0.530643 | 0.962778 | 1 | 4201 | tags=26%, list=18%, signal=32% |
| 0.349054 | 0.976493 | 0.499127 | 0.963376 | 1 | 3188 | tags=33%, list=14%, signal=39% |
| 0.280103 | 0.976179 | 0.539342 | 0.963006 | 1 | 2960 | tags=25%, list=13%, signal=29% |
| 0.30932 | 0.975944 | 0.445351 | 0.96241 | 1 | 4896 | tags=35%, list=21%, signal=44% |
| 0.338927 | 0.975801 | 0.468852 | 0.961548 | 1 | 5094 | tags=39%, list=22%, signal=50% |
| 0.51108 | 0.975615 | 0.573077 | 0.960844 | 1 | 7358 | tags=67%, list=31%, signal=97% |
| 0.270623 | 0.97491 | 0.428816 | 0.961651 | 1 | 5573 | tags=37%, list=24%, signal=48% |
| 0.360851 | 0.97485 | 0.508943 | 0.96054 | 1 | 2597 | tags=28%, list=11%, signal=31% |
| 0.365136 | 0.97476 | 0.488889 | 0.959548 | 1 | 5020 | tags=40%, list=21%, signal=51% |
| 0.424427 | 0.974414 | 0.510358 | 0.959179 | 1 | 5607 | tags=56%, list=24%, signal=74% |
| 0.388928 | 0.973929 | 0.489399 | 0.95931 | 1 | 5532 | tags=46%, list=24%, signal=60% |
| 0.353516 | 0.973928 | 0.471795 | 0.958031 | 1 | 4726 | tags=39%, list=20%, signal=49% |
| 0.336096 | 0.971847 | 0.503247 | 0.962664 | 1 | 4217 | tags=40%, list=18%, signal=48% |
| 0.289586 | 0.971626 | 0.4375 | 0.962023 | 1 | 3842 | tags=30%, list=16%, signal=36% |
| 0.366184 | 0.971346 | 0.522642 | 0.961537 | 1 | 6280 | tags=47%, list=27%, signal=64% |
| 0.35742 | 0.971005 | 0.507132 | 0.961292 | 1 | 3129 | tags=30%, list=13%, signal=34% |

| | | | | | | |
|---|---|---|---|---|---|---|
| 0.352896 | 0.970958 | 0.500907 | 0.960161 | 1 | 3162 | tags=30%, list=14%, signal=35% |
| 0.3994 | 0.970901 | 0.489362 | 0.95912 | 1 | 6868 | tags=72%, list=29%, signal=102% |
| 0.303195 | 0.970273 | 0.540115 | 0.959538 | 1 | 4004 | tags=32%, list=17%, signal=38% |
| 0.277458 | 0.970258 | 0.533813 | 0.958304 | 1 | 5993 | tags=43%, list=26%, signal=57% |
| 0.271082 | 0.969748 | 0.530142 | 0.958564 | 1 | 2676 | tags=19%, list=11%, signal=21% |
| 0.318287 | 0.968973 | 0.488487 | 0.959521 | 1 | 3469 | tags=33%, list=15%, signal=38% |
| 0.360927 | 0.967834 | 0.503852 | 0.961504 | 1 | 5370 | tags=35%, list=23%, signal=45% |
| 0.386244 | 0.967808 | 0.515548 | 0.960341 | 1 | 4926 | tags=50%, list=21%, signal=63% |
| 0.510428 | 0.967663 | 0.567669 | 0.959497 | 1 | 10207 | tags=84%, list=44%, signal=149% |
| 0.285211 | 0.967622 | 0.51436 | 0.958378 | 1 | 5430 | tags=38%, list=23%, signal=49% |
| 0.330112 | 0.967205 | 0.524476 | 0.95827 | 1 | 4766 | tags=38%, list=20%, signal=48% |
| 0.329727 | 0.966787 | 0.553655 | 0.958088 | 1 | 5565 | tags=47%, list=24%, signal=62% |
| 0.336313 | 0.966718 | 0.458763 | 0.957064 | 1 | 3777 | tags=26%, list=16%, signal=32% |
| 0.421915 | 0.966632 | 0.5 | 0.956086 | 1 | 6213 | tags=54%, list=27%, signal=74% |
| 0.327376 | 0.966476 | 0.499044 | 0.955295 | 1 | 4334 | tags=36%, list=19%, signal=44% |
| 0.2656 | 0.966468 | 0.609023 | 0.954058 | 1 | 3503 | tags=25%, list=15%, signal=29% |
| 0.309779 | 0.966373 | 0.533784 | 0.953075 | 1 | 4638 | tags=35%, list=20%, signal=43% |
| 0.370612 | 0.965859 | 0.495033 | 0.953168 | 1 | 6368 | tags=42%, list=27%, signal=58% |
| 0.297999 | 0.965628 | 0.52 | 0.952565 | 1 | 4038 | tags=37%, list=17%, signal=45% |
| 0.344338 | 0.964945 | 0.508604 | 0.953235 | 1 | 2502 | tags=33%, list=11%, signal=37% |
| 0.304761 | 0.964803 | 0.493716 | 0.952435 | 1 | 3737 | tags=30%, list=16%, signal=36% |
| 0.331654 | 0.964599 | 0.495327 | 0.951833 | 1 | 3788 | tags=37%, list=16%, signal=44% |
| 0.353399 | 0.96436 | 0.522073 | 0.951264 | 1 | 5020 | tags=34%, list=21%, signal=44% |
| 0.285495 | 0.964061 | 0.535771 | 0.950889 | 1 | 5537 | tags=36%, list=24%, signal=48% |
| 0.380462 | 0.962942 | 0.49639 | 0.953003 | 1 | 3667 | tags=34%, list=16%, signal=41% |
| 0.273808 | 0.96227 | 0.509589 | 0.953695 | 1 | 3808 | tags=32%, list=16%, signal=38% |
| 0.238291 | 0.961172 | 0.518836 | 0.955454 | 1 | 4308 | tags=24%, list=18%, signal=29% |
| 0.430028 | 0.961045 | 0.530075 | 0.954544 | 1 | 5962 | tags=54%, list=25%, signal=71% |
| 0.383298 | 0.960337 | 0.474954 | 0.95525 | 1 | 7718 | tags=58%, list=33%, signal=86% |
| 0.352311 | 0.959898 | 0.524074 | 0.955255 | 1 | 2371 | tags=30%, list=10%, signal=34% |
| 0.265931 | 0.95967 | 0.505376 | 0.95468 | 1 | 4441 | tags=32%, list=19%, signal=39% |
| 0.378325 | 0.958629 | 0.565436 | 0.956178 | 1 | 435 | tags=14%, list=2%, signal=15% |
| 0.430905 | 0.958531 | 0.559265 | 0.955219 | 1 | 6188 | tags=43%, list=26%, signal=58% |
| 0.322313 | 0.958487 | 0.462712 | 0.954107 | 1 | 3026 | tags=29%, list=13%, signal=33% |
| 0.400444 | 0.958426 | 0.552511 | 0.953059 | 1 | 3342 | tags=35%, list=14%, signal=41% |
| 0.296841 | 0.958058 | 0.561482 | 0.952873 | 1 | 4591 | tags=40%, list=20%, signal=50% |
| 0.297807 | 0.957681 | 0.493789 | 0.952646 | 1 | 5933 | tags=45%, list=25%, signal=60% |
| 0.273081 | 0.957519 | 0.485207 | 0.95191 | 1 | 3512 | tags=27%, list=15%, signal=32% |
| 0.325856 | 0.957475 | 0.536082 | 0.95087 | 1 | 4534 | tags=40%, list=19%, signal=50% |
| 0.245295 | 0.95744 | 0.60469 | 0.949767 | 1 | 4597 | tags=25%, list=20%, signal=31% |
| 0.256501 | 0.956137 | 0.556985 | 0.952308 | 1 | 3547 | tags=24%, list=15%, signal=29% |
| 0.373749 | 0.955975 | 0.48474 | 0.951613 | 1 | 5238 | tags=41%, list=22%, signal=52% |
| 0.358358 | 0.955805 | 0.487319 | 0.950914 | 1 | 5962 | tags=46%, list=25%, signal=62% |
| 0.238758 | 0.955772 | 0.635071 | 0.949836 | 1 | 4720 | tags=29%, list=20%, signal=36% |
| 0.361292 | 0.955342 | 0.49262 | 0.949966 | 1 | 6421 | tags=46%, list=27%, signal=64% |
| 0.30861 | 0.95471 | 0.566553 | 0.9504 | 1 | 1862 | tags=17%, list=8%, signal=18% |
| 0.26794 | 0.954276 | 0.533981 | 0.950401 | 1 | 4338 | tags=34%, list=19%, signal=42% |
| 0.315348 | 0.954086 | 0.576976 | 0.949717 | 1 | 3521 | tags=32%, list=15%, signal=37% |
| 0.317451 | 0.953942 | 0.525 | 0.948937 | 1 | 6369 | tags=53%, list=27%, signal=72% |
| 0.281915 | 0.953685 | 0.568628 | 0.948377 | 1 | 4125 | tags=33%, list=18%, signal=40% |

| | | | | | | |
|---|---|---|---|---|---|---|
| 0.381503 | 0.953155 | 0.53578 | 0.948505 | 1 | 5246 | tags=56%, list=22%, signal=73% |
| 0.436542 | 0.952852 | 0.518095 | 0.948148 | 1 | 5580 | tags=50%, list=24%, signal=65% |
| 0.314904 | 0.952697 | 0.512563 | 0.947422 | 1 | 2470 | tags=31%, list=11%, signal=34% |
| 0.243657 | 0.952577 | 0.511327 | 0.946621 | 1 | 5140 | tags=31%, list=22%, signal=39% |
| 0.315165 | 0.952549 | 0.563218 | 0.945515 | 1 | 2375 | tags=27%, list=10%, signal=30% |
| 0.315621 | 0.952534 | 0.502075 | 0.944391 | 1 | 3381 | tags=21%, list=14%, signal=25% |
| 0.401258 | 0.951987 | 0.536585 | 0.944673 | 1 | 5777 | tags=46%, list=25%, signal=61% |
| 0.355585 | 0.951782 | 0.546945 | 0.944052 | 1 | 3705 | tags=36%, list=16%, signal=42% |
| 0.263623 | 0.951686 | 0.606232 | 0.94315 | 1 | 2619 | tags=21%, list=11%, signal=23% |
| 0.369843 | 0.951544 | 0.547107 | 0.942407 | 1 | 6416 | tags=49%, list=27%, signal=67% |
| 0.305761 | 0.951496 | 0.54359 | 0.941368 | 1 | 4683 | tags=39%, list=20%, signal=48% |
| 0.448495 | 0.949542 | 0.558394 | 0.945762 | 1 | 5020 | tags=48%, list=21%, signal=61% |
| 0.291958 | 0.949311 | 0.535354 | 0.945238 | 1 | 4993 | tags=35%, list=21%, signal=44% |
| 0.356617 | 0.949177 | 0.591837 | 0.944439 | 1 | 3360 | tags=34%, list=14%, signal=40% |
| 0.319531 | 0.949152 | 0.578773 | 0.943358 | 1 | 3576 | tags=29%, list=15%, signal=34% |
| 0.369076 | 0.949033 | 0.570988 | 0.942561 | 1 | 2466 | tags=31%, list=11%, signal=35% |
| 0.3531 | 0.948908 | 0.519481 | 0.94174 | 1 | 4773 | tags=38%, list=20%, signal=48% |
| 0.348734 | 0.948752 | 0.502982 | 0.941013 | 1 | 4100 | tags=36%, list=18%, signal=43% |
| 0.409176 | 0.94834 | 0.52552 | 0.940934 | 1 | 4802 | tags=51%, list=21%, signal=64% |
| 0.361238 | 0.948129 | 0.537477 | 0.9404 | 1 | 3148 | tags=31%, list=13%, signal=36% |
| 0.340233 | 0.947613 | 0.562271 | 0.940633 | 1 | 1387 | tags=20%, list=6%, signal=21% |
| 0.358307 | 0.947441 | 0.572289 | 0.93993 | 1 | 6909 | tags=50%, list=30%, signal=71% |
| 0.253918 | 0.947125 | 0.524194 | 0.939605 | 1 | 2739 | tags=22%, list=12%, signal=25% |
| 0.269802 | 0.946916 | 0.6048 | 0.939001 | 1 | 3725 | tags=27%, list=16%, signal=32% |
| 0.398158 | 0.946884 | 0.550373 | 0.93796 | 1 | 5152 | tags=35%, list=22%, signal=45% |
| 0.445462 | 0.946031 | 0.547349 | 0.939058 | 1 | 6095 | tags=52%, list=26%, signal=70% |
| 0.478172 | 0.944638 | 0.647482 | 0.941756 | 1 | 5859 | tags=56%, list=25%, signal=75% |
| 0.305024 | 0.94416 | 0.500778 | 0.941758 | 1 | 6423 | tags=44%, list=27%, signal=61% |
| 0.317715 | 0.94379 | 0.531746 | 0.941609 | 1 | 3759 | tags=30%, list=16%, signal=36% |
| 0.229119 | 0.943328 | 0.680481 | 0.941755 | 1 | 6051 | tags=35%, list=26%, signal=47% |
| 0.327661 | 0.943195 | 0.556688 | 0.940916 | 1 | 4672 | tags=35%, list=20%, signal=44% |
| 0.327799 | 0.943052 | 0.566973 | 0.940172 | 1 | 2664 | tags=22%, list=11%, signal=25% |
| 0.405292 | 0.942989 | 0.548944 | 0.939217 | 1 | 2484 | tags=33%, list=11%, signal=36% |
| 0.255143 | 0.942557 | 0.522152 | 0.939164 | 1 | 5930 | tags=38%, list=25%, signal=50% |
| 0.273689 | 0.942135 | 0.563718 | 0.939264 | 1 | 5705 | tags=39%, list=24%, signal=52% |
| 0.32839 | 0.942091 | 0.571429 | 0.938238 | 1 | 3044 | tags=29%, list=13%, signal=33% |
| 0.287112 | 0.941923 | 0.519562 | 0.93755 | 1 | 5573 | tags=52%, list=24%, signal=69% |
| 0.429629 | 0.941661 | 0.545794 | 0.937104 | 1 | 3851 | tags=32%, list=16%, signal=38% |
| 0.264829 | 0.940684 | 0.583861 | 0.93859 | 1 | 4008 | tags=30%, list=17%, signal=36% |
| 0.253417 | 0.939698 | 0.557692 | 0.940073 | 1 | 5857 | tags=39%, list=25%, signal=51% |
| 0.363854 | 0.939572 | 0.505597 | 0.93931 | 1 | 5903 | tags=46%, list=25%, signal=61% |
| 0.287115 | 0.939479 | 0.618026 | 0.938467 | 1 | 5473 | tags=47%, list=23%, signal=61% |
| 0.276999 | 0.939311 | 0.598291 | 0.937719 | 1 | 3133 | tags=27%, list=13%, signal=31% |
| 0.317117 | 0.93834 | 0.580938 | 0.939116 | 1 | 5272 | tags=40%, list=23%, signal=52% |
| 0.311908 | 0.937988 | 0.613057 | 0.938917 | 1 | 6314 | tags=47%, list=27%, signal=64% |
| 0.264758 | 0.937866 | 0.601969 | 0.938136 | 1 | 5761 | tags=37%, list=25%, signal=49% |
| 0.261151 | 0.93732 | 0.616242 | 0.9384 | 1 | 2974 | tags=22%, list=13%, signal=25% |
| 0.237291 | 0.937261 | 0.6277 | 0.937436 | 1 | 5283 | tags=34%, list=23%, signal=43% |
| 0.315562 | 0.937164 | 0.590529 | 0.936624 | 1 | 5717 | tags=41%, list=24%, signal=54% |
| 0.279555 | 0.936614 | 0.553448 | 0.936953 | 1 | 2566 | tags=21%, list=11%, signal=23% |

| | | | | | | |
|---|---|---|---|---|---|---|
| 0.355047 | 0.935952 | 0.508197 | 0.937476 | 1 | 6914 | tags=52%, list=30%, signal=74% |
| 0.285049 | 0.935183 | 0.545603 | 0.938304 | 1 | 3739 | tags=26%, list=16%, signal=31% |
| 0.318648 | 0.934756 | 0.551089 | 0.938274 | 1 | 2618 | tags=24%, list=11%, signal=28% |
| 0.306239 | 0.933875 | 0.701062 | 0.939327 | 1 | 3132 | tags=31%, list=13%, signal=36% |
| 0.273806 | 0.933758 | 0.62064 | 0.93854 | 1 | 5169 | tags=35%, list=22%, signal=45% |
| 0.377308 | 0.933699 | 0.584939 | 0.937599 | 1 | 5907 | tags=40%, list=25%, signal=53% |
| 0.343793 | 0.933675 | 0.568218 | 0.936592 | 1 | 2470 | tags=27%, list=11%, signal=30% |
| 0.293464 | 0.933264 | 0.605096 | 0.936629 | 1 | 2101 | tags=14%, list=9%, signal=16% |
| 0.235292 | 0.933152 | 0.613162 | 0.935814 | 1 | 4665 | tags=29%, list=20%, signal=36% |
| 0.291892 | 0.932593 | 0.511265 | 0.936241 | 1 | 2782 | tags=29%, list=12%, signal=33% |
| 0.503198 | 0.932537 | 0.567362 | 0.935305 | 1 | 5152 | tags=53%, list=22%, signal=68% |
| 0.400348 | 0.930924 | 0.64526 | 0.93835 | 1 | 5809 | tags=56%, list=25%, signal=74% |
| 0.309552 | 0.930476 | 0.574766 | 0.938531 | 1 | 7275 | tags=49%, list=31%, signal=70% |
| 0.280605 | 0.930461 | 0.59271 | 0.937495 | 1 | 2842 | tags=21%, list=12%, signal=24% |
| 0.380197 | 0.93031 | 0.54461 | 0.936758 | 1 | 5391 | tags=47%, list=23%, signal=61% |
| 0.285136 | 0.929822 | 0.625592 | 0.936831 | 1 | 2738 | tags=21%, list=12%, signal=24% |
| 0.305391 | 0.928356 | 0.544839 | 0.939577 | 1 | 3739 | tags=29%, list=16%, signal=35% |
| 0.315498 | 0.928163 | 0.50478 | 0.939042 | 1 | 3533 | tags=23%, list=15%, signal=27% |
| 0.432618 | 0.927705 | 0.564576 | 0.939146 | 1 | 6277 | tags=62%, list=27%, signal=85% |
| 0.310177 | 0.927371 | 0.529963 | 0.938945 | 1 | 2722 | tags=27%, list=12%, signal=30% |
| 0.271211 | 0.926596 | 0.590379 | 0.939921 | 1 | 3424 | tags=26%, list=15%, signal=30% |
| 0.324092 | 0.92631 | 0.609023 | 0.939541 | 1 | 4344 | tags=31%, list=19%, signal=39% |
| 0.302777 | 0.92564 | 0.606876 | 0.94014 | 1 | 3386 | tags=30%, list=14%, signal=35% |
| 0.358514 | 0.925563 | 0.569966 | 0.939266 | 1 | 6582 | tags=47%, list=28%, signal=65% |
| 0.284328 | 0.925193 | 0.604134 | 0.93904 | 1 | 5882 | tags=43%, list=25%, signal=58% |
| 0.242837 | 0.924332 | 0.545317 | 0.940115 | 1 | 4109 | tags=28%, list=18%, signal=33% |
| 0.455857 | 0.92415 | 0.616056 | 0.939522 | 1 | 5283 | tags=53%, list=23%, signal=69% |
| 0.406549 | 0.923826 | 0.582721 | 0.93938 | 1 | 7093 | tags=58%, list=30%, signal=84% |
| 0.332882 | 0.923714 | 0.533088 | 0.938572 | 1 | 4694 | tags=35%, list=20%, signal=43% |
| 0.252215 | 0.923121 | 0.699115 | 0.938979 | 1 | 3883 | tags=28%, list=17%, signal=33% |
| 0.281658 | 0.921983 | 0.59633 | 0.940742 | 1 | 3675 | tags=29%, list=16%, signal=34% |
| 0.27039 | 0.921949 | 0.554577 | 0.939769 | 1 | 5770 | tags=35%, list=25%, signal=47% |
| 0.282308 | 0.921883 | 0.602787 | 0.938854 | 1 | 2736 | tags=20%, list=12%, signal=23% |
| 0.239656 | 0.921349 | 0.659864 | 0.939102 | 1 | 5908 | tags=37%, list=25%, signal=49% |
| 0.326856 | 0.920565 | 0.553704 | 0.939956 | 1 | 3072 | tags=30%, list=13%, signal=35% |
| 0.290106 | 0.920289 | 0.613846 | 0.939544 | 1 | 3184 | tags=21%, list=14%, signal=25% |
| 0.27526 | 0.919843 | 0.561567 | 0.939565 | 1 | 7299 | tags=44%, list=31%, signal=64% |
| 0.319941 | 0.91851 | 0.564338 | 0.941777 | 1 | 5884 | tags=40%, list=25%, signal=53% |
| 0.480036 | 0.918187 | 0.573801 | 0.941562 | 1 | 8050 | tags=77%, list=34%, signal=118% |
| 0.292975 | 0.917868 | 0.563973 | 0.941394 | 1 | 6171 | tags=48%, list=26%, signal=66% |
| 0.284941 | 0.917329 | 0.599026 | 0.941701 | 1 | 1732 | tags=19%, list=7%, signal=21% |
| 0.26809 | 0.917193 | 0.536458 | 0.94101 | 1 | 3161 | tags=26%, list=13%, signal=30% |
| 0.390511 | 0.916457 | 0.619211 | 0.941861 | 1 | 4625 | tags=40%, list=20%, signal=50% |
| 0.400406 | 0.915962 | 0.576655 | 0.942082 | 1 | 6004 | tags=52%, list=26%, signal=70% |
| 0.317199 | 0.915956 | 0.6 | 0.941043 | 1 | 2568 | tags=23%, list=11%, signal=26% |
| 0.290559 | 0.915667 | 0.624782 | 0.940643 | 1 | 3368 | tags=26%, list=14%, signal=31% |
| 0.31783 | 0.915365 | 0.588015 | 0.940351 | 1 | 7198 | tags=60%, list=31%, signal=87% |
| 0.343599 | 0.915317 | 0.622793 | 0.939433 | 1 | 4562 | tags=37%, list=19%, signal=46% |
| 0.389297 | 0.914842 | 0.569498 | 0.939609 | 1 | 7899 | tags=61%, list=34%, signal=91% |
| 0.235059 | 0.914774 | 0.698217 | 0.938712 | 1 | 2853 | tags=21%, list=12%, signal=23% |

| | | | | | | |
|---|---|---|---|---|---|---|
| 0.238671 | 0.914594 | 0.728045 | 0.938096 | 1 | 5785 | tags=35%, list=25%, signal=46% |
| 0.349225 | 0.914319 | 0.582011 | 0.93777 | 1 | 4887 | tags=52%, list=21%, signal=65% |
| 0.272432 | 0.914048 | 0.547025 | 0.937355 | 1 | 5787 | tags=37%, list=25%, signal=48% |
| 0.270145 | 0.913842 | 0.571906 | 0.936902 | 1 | 5857 | tags=39%, list=25%, signal=52% |
| 0.314668 | 0.912768 | 0.583333 | 0.93867 | 1 | 1277 | tags=20%, list=5%, signal=21% |
| 0.28182 | 0.91249 | 0.578231 | 0.938342 | 1 | 3041 | tags=25%, list=13%, signal=28% |
| 0.252475 | 0.912197 | 0.632964 | 0.937996 | 1 | 5847 | tags=39%, list=25%, signal=52% |
| 0.340037 | 0.911818 | 0.5681 | 0.937944 | 1 | 3978 | tags=38%, list=17%, signal=45% |
| 0.366657 | 0.911616 | 0.548701 | 0.937443 | 1 | 5564 | tags=46%, list=24%, signal=60% |
| 0.356405 | 0.911329 | 0.525755 | 0.937173 | 1 | 5178 | tags=52%, list=22%, signal=67% |
| 0.403283 | 0.911285 | 0.61426 | 0.936252 | 1 | 5152 | tags=40%, list=22%, signal=51% |
| 0.35009 | 0.910881 | 0.587931 | 0.936178 | 1 | 7038 | tags=60%, list=30%, signal=86% |
| 0.385089 | 0.91068 | 0.547619 | 0.935589 | 1 | 5580 | tags=47%, list=24%, signal=61% |
| 0.29908 | 0.909871 | 0.526126 | 0.936471 | 1 | 5877 | tags=39%, list=25%, signal=52% |
| 0.292277 | 0.909791 | 0.638261 | 0.935661 | 1 | 4404 | tags=36%, list=19%, signal=44% |
| 0.269072 | 0.909774 | 0.605128 | 0.93468 | 1 | 5133 | tags=34%, list=22%, signal=44% |
| 0.398948 | 0.909381 | 0.57393 | 0.93464 | 1 | 5482 | tags=48%, list=23%, signal=63% |
| 0.332271 | 0.909259 | 0.566667 | 0.933967 | 1 | 5802 | tags=40%, list=25%, signal=53% |
| 0.309508 | 0.908752 | 0.568421 | 0.934164 | 1 | 8033 | tags=58%, list=34%, signal=88% |
| 0.346189 | 0.908635 | 0.597956 | 0.933412 | 1 | 2594 | tags=31%, list=11%, signal=35% |
| 0.379491 | 0.908286 | 0.550285 | 0.933275 | 1 | 5952 | tags=47%, list=25%, signal=62% |
| 0.332708 | 0.90803 | 0.616613 | 0.932925 | 1 | 6491 | tags=50%, list=28%, signal=69% |
| 0.302622 | 0.908026 | 0.645609 | 0.931926 | 1 | 4146 | tags=29%, list=18%, signal=35% |
| 0.337885 | 0.907747 | 0.530973 | 0.931637 | 1 | 5246 | tags=40%, list=22%, signal=51% |
| 0.309342 | 0.907106 | 0.621818 | 0.932126 | 1 | 5465 | tags=42%, list=23%, signal=55% |
| 0.328554 | 0.906777 | 0.588665 | 0.931961 | 1 | 1294 | tags=20%, list=6%, signal=21% |
| 0.337907 | 0.906666 | 0.61597 | 0.931205 | 1 | 1175 | tags=21%, list=5%, signal=22% |
| 0.326927 | 0.906587 | 0.555357 | 0.930393 | 1 | 5852 | tags=48%, list=25%, signal=63% |
| 0.417255 | 0.906291 | 0.559041 | 0.930142 | 1 | 4797 | tags=33%, list=20%, signal=42% |
| 0.325085 | 0.906285 | 0.600939 | 0.929157 | 1 | 4308 | tags=32%, list=18%, signal=39% |
| 0.326058 | 0.906082 | 0.576512 | 0.928625 | 1 | 6053 | tags=47%, list=26%, signal=63% |
| 0.229869 | 0.905467 | 0.763291 | 0.929052 | 1 | 3892 | tags=25%, list=17%, signal=30% |
| 0.295904 | 0.905248 | 0.718519 | 0.928545 | 1 | 1374 | tags=17%, list=6%, signal=18% |
| 0.339639 | 0.904586 | 0.616822 | 0.929224 | 1 | 3848 | tags=48%, list=16%, signal=58% |
| 0.425949 | 0.90448 | 0.629845 | 0.928524 | 1 | 5877 | tags=55%, list=25%, signal=73% |
| 0.354618 | 0.903899 | 0.568471 | 0.928845 | 1 | 6499 | tags=56%, list=28%, signal=77% |
| 0.273051 | 0.903728 | 0.664516 | 0.928288 | 1 | 4204 | tags=31%, list=18%, signal=37% |
| 0.379326 | 0.903724 | 0.593592 | 0.927302 | 1 | 2839 | tags=20%, list=12%, signal=23% |
| 0.259581 | 0.903712 | 0.647163 | 0.92634 | 1 | 6370 | tags=36%, list=27%, signal=50% |
| 0.309407 | 0.903671 | 0.65008 | 0.925455 | 1 | 4818 | tags=41%, list=21%, signal=52% |
| 0.294861 | 0.903579 | 0.603531 | 0.924684 | 1 | 2932 | tags=25%, list=13%, signal=29% |
| 0.224553 | 0.902961 | 0.68369 | 0.9251 | 1 | 4665 | tags=29%, list=20%, signal=36% |
| 0.236639 | 0.901957 | 0.693467 | 0.926511 | 1 | 5632 | tags=34%, list=24%, signal=45% |
| 0.303766 | 0.901293 | 0.614754 | 0.926988 | 1 | 5208 | tags=38%, list=22%, signal=48% |
| 0.237855 | 0.900715 | 0.679487 | 0.927325 | 1 | 2796 | tags=23%, list=12%, signal=26% |
| 0.321289 | 0.900152 | 0.607023 | 0.927572 | 1 | 3375 | tags=31%, list=14%, signal=36% |
| 0.247782 | 0.899171 | 0.612319 | 0.928892 | 1 | 5736 | tags=35%, list=24%, signal=46% |
| 0.252146 | 0.89836 | 0.677698 | 0.929883 | 1 | 3381 | tags=27%, list=14%, signal=31% |
| 0.26681 | 0.897766 | 0.696429 | 0.930245 | 1 | 5530 | tags=39%, list=24%, signal=50% |
| 0.40574 | 0.897402 | 0.641953 | 0.93007 | 1 | 3036 | tags=35%, list=13%, signal=41% |

| | | | | | | |
|---|---|---|---|---|---|---|
| 0.32285 | 0.896839 | 0.570565 | 0.930408 | 1 | 4727 | tags=39%, list=20%, signal=49% |
| 0.295221 | 0.896726 | 0.591767 | 0.929683 | 1 | 4451 | tags=30%, list=19%, signal=37% |
| 0.315782 | 0.896725 | 0.607143 | 0.928716 | 1 | 5428 | tags=36%, list=23%, signal=47% |
| 0.27613 | 0.896623 | 0.627809 | 0.927901 | 1 | 5909 | tags=37%, list=25%, signal=49% |
| 0.442965 | 0.89654 | 0.590741 | 0.927124 | 1 | 8050 | tags=67%, list=34%, signal=101% |
| 0.287959 | 0.896359 | 0.593807 | 0.926631 | 1 | 3869 | tags=28%, list=17%, signal=34% |
| 0.386898 | 0.895558 | 0.548023 | 0.927597 | 1 | 3825 | tags=37%, list=16%, signal=44% |
| 0.285936 | 0.895437 | 0.662441 | 0.926976 | 1 | 2027 | tags=19%, list=9%, signal=20% |
| 0.334226 | 0.895311 | 0.628425 | 0.926289 | 1 | 2966 | tags=33%, list=13%, signal=38% |
| 0.278192 | 0.895077 | 0.596745 | 0.925916 | 1 | 7439 | tags=56%, list=32%, signal=81% |
| 0.357429 | 0.894976 | 0.633758 | 0.925179 | 1 | 4963 | tags=36%, list=21%, signal=46% |
| 0.249552 | 0.894722 | 0.687302 | 0.924781 | 1 | 2415 | tags=18%, list=10%, signal=20% |
| 0.237035 | 0.89469 | 0.696275 | 0.923909 | 1 | 5651 | tags=42%, list=24%, signal=55% |
| 0.340302 | 0.894565 | 0.634892 | 0.923225 | 1 | 3327 | tags=33%, list=14%, signal=39% |
| 0.419964 | 0.894184 | 0.581921 | 0.923161 | 1 | 7965 | tags=70%, list=34%, signal=105% |
| 0.280611 | 0.893672 | 0.663793 | 0.923446 | 1 | 5794 | tags=42%, list=25%, signal=55% |
| 0.27644 | 0.893164 | 0.634069 | 0.923695 | 1 | 2966 | tags=24%, list=13%, signal=27% |
| 0.317069 | 0.892622 | 0.647841 | 0.923962 | 1 | 2659 | tags=28%, list=11%, signal=31% |
| 0.252137 | 0.891703 | 0.646259 | 0.92506 | 1 | 3395 | tags=27%, list=14%, signal=32% |
| 0.248541 | 0.891 | 0.690583 | 0.925722 | 1 | 5465 | tags=35%, list=23%, signal=45% |
| 0.269985 | 0.890969 | 0.555716 | 0.924848 | 1 | 5908 | tags=43%, list=25%, signal=57% |
| 0.260778 | 0.8907 | 0.699837 | 0.924568 | 1 | 5044 | tags=26%, list=22%, signal=34% |
| 0.240651 | 0.890214 | 0.738028 | 0.924783 | 1 | 3048 | tags=23%, list=13%, signal=27% |
| 0.305505 | 0.889837 | 0.657556 | 0.924734 | 1 | 2466 | tags=25%, list=11%, signal=28% |
| 0.30961 | 0.889387 | 0.649148 | 0.924872 | 1 | 5523 | tags=36%, list=24%, signal=47% |
| 0.268263 | 0.889164 | 0.643505 | 0.924452 | 1 | 2816 | tags=23%, list=12%, signal=26% |
| 0.27178 | 0.888989 | 0.727666 | 0.923854 | 1 | 2722 | tags=26%, list=12%, signal=29% |
| 0.32279 | 0.8881 | 0.614853 | 0.925002 | 1 | 6100 | tags=45%, list=26%, signal=61% |
| 0.383288 | 0.887972 | 0.562738 | 0.924325 | 1 | 5421 | tags=47%, list=23%, signal=61% |
| 0.26689 | 0.887948 | 0.655987 | 0.923424 | 1 | 4204 | tags=31%, list=18%, signal=38% |
| 0.288135 | 0.887737 | 0.673701 | 0.922915 | 1 | 4108 | tags=30%, list=18%, signal=36% |
| 0.404094 | 0.8876 | 0.566728 | 0.922324 | 1 | 7901 | tags=66%, list=34%, signal=99% |
| 0.250788 | 0.88743 | 0.676638 | 0.921722 | 1 | 5593 | tags=37%, list=24%, signal=48% |
| 0.279977 | 0.886987 | 0.770227 | 0.921729 | 1 | 4143 | tags=30%, list=18%, signal=37% |
| 0.318836 | 0.886961 | 0.574861 | 0.920865 | 1 | 5868 | tags=41%, list=25%, signal=55% |
| 0.381205 | 0.886898 | 0.568015 | 0.9201 | 1 | 4802 | tags=48%, list=21%, signal=60% |
| 0.272981 | 0.886191 | 0.637771 | 0.920738 | 1 | 2522 | tags=21%, list=11%, signal=24% |
| 0.339268 | 0.885918 | 0.592087 | 0.92044 | 1 | 5713 | tags=50%, list=24%, signal=66% |
| 0.445807 | 0.885539 | 0.643178 | 0.920316 | 1 | 6315 | tags=65%, list=27%, signal=88% |
| 0.323948 | 0.885453 | 0.710456 | 0.919593 | 1 | 4243 | tags=42%, list=18%, signal=52% |
| 0.28659 | 0.884914 | 0.731278 | 0.920014 | 1 | 3500 | tags=28%, list=15%, signal=32% |
| 0.340001 | 0.884676 | 0.588785 | 0.919686 | 1 | 5020 | tags=44%, list=21%, signal=56% |
| 0.336808 | 0.884597 | 0.630182 | 0.918951 | 1 | 3786 | tags=32%, list=16%, signal=38% |
| 0.315263 | 0.884469 | 0.689597 | 0.918288 | 1 | 3062 | tags=33%, list=13%, signal=38% |
| 0.239858 | 0.884422 | 0.725086 | 0.917467 | 1 | 3633 | tags=23%, list=16%, signal=27% |
| 0.28719 | 0.884065 | 0.67033 | 0.917423 | 1 | 6033 | tags=40%, list=26%, signal=53% |
| 0.217539 | 0.883922 | 0.784993 | 0.916849 | 1 | 3449 | tags=23%, list=15%, signal=27% |
| 0.31324 | 0.883235 | 0.61244 | 0.917559 | 1 | 5433 | tags=33%, list=23%, signal=43% |
| 0.358189 | 0.882764 | 0.636929 | 0.917779 | 1 | 2470 | tags=33%, list=11%, signal=37% |
| 0.306522 | 0.882642 | 0.678238 | 0.917102 | 1 | 6854 | tags=53%, list=29%, signal=75% |

| | | | | | | |
|---|---|---|---|---|---|---|
| 0.309816 | 0.882412 | 0.606272 | 0.916699 | 1 | 5339 | tags=43%, list=23%, signal=56% |
| 0.317789 | 0.882382 | 0.688496 | 0.915879 | 1 | 9034 | tags=69%, list=39%, signal=112% |
| 0.297901 | 0.881367 | 0.613126 | 0.917173 | 1 | 3419 | tags=21%, list=15%, signal=24% |
| 0.254159 | 0.880634 | 0.705247 | 0.91782 | 1 | 2952 | tags=21%, list=13%, signal=24% |
| 0.322018 | 0.880163 | 0.695868 | 0.917953 | 1 | 5107 | tags=28%, list=22%, signal=35% |
| 0.27782 | 0.87999 | 0.679856 | 0.917397 | 1 | 1301 | tags=11%, list=6%, signal=11% |
| 0.298666 | 0.87994 | 0.609428 | 0.916606 | 1 | 2416 | tags=26%, list=10%, signal=29% |
| 0.329523 | 0.879925 | 0.674174 | 0.915733 | 1 | 5474 | tags=33%, list=23%, signal=43% |
| 0.412457 | 0.879723 | 0.615672 | 0.915309 | 1 | 3870 | tags=27%, list=17%, signal=33% |
| 0.251635 | 0.879594 | 0.697161 | 0.914665 | 1 | 4268 | tags=30%, list=18%, signal=37% |
| 0.246482 | 0.879427 | 0.66766 | 0.914152 | 1 | 4981 | tags=32%, list=21%, signal=41% |
| 0.252715 | 0.879315 | 0.70015 | 0.91349 | 1 | 5918 | tags=38%, list=25%, signal=50% |
| 0.30934 | 0.878542 | 0.734597 | 0.914265 | 1 | 4321 | tags=39%, list=18%, signal=48% |
| 0.338761 | 0.878417 | 0.655116 | 0.913635 | 1 | 3725 | tags=40%, list=16%, signal=48% |
| 0.316379 | 0.878136 | 0.655436 | 0.913377 | 1 | 4268 | tags=43%, list=18%, signal=52% |
| 0.303955 | 0.87804 | 0.676752 | 0.912707 | 1 | 3391 | tags=33%, list=14%, signal=38% |
| 0.385751 | 0.877946 | 0.674536 | 0.911974 | 1 | 5108 | tags=42%, list=22%, signal=54% |
| 0.353351 | 0.877808 | 0.717687 | 0.911433 | 1 | 3072 | tags=40%, list=13%, signal=46% |
| 0.370646 | 0.877718 | 0.614341 | 0.910696 | 1 | 5020 | tags=39%, list=21%, signal=50% |
| 0.279381 | 0.8774 | 0.69702 | 0.910549 | 1 | 4599 | tags=34%, list=20%, signal=42% |
| 0.352755 | 0.876841 | 0.656301 | 0.910933 | 1 | 6960 | tags=55%, list=30%, signal=79% |
| 0.332918 | 0.876816 | 0.629565 | 0.910095 | 1 | 6733 | tags=38%, list=29%, signal=53% |
| 0.296464 | 0.876576 | 0.666201 | 0.909728 | 1 | 3833 | tags=32%, list=16%, signal=38% |
| 0.242875 | 0.876432 | 0.687692 | 0.909172 | 1 | 4428 | tags=31%, list=19%, signal=37% |
| 0.262653 | 0.875505 | 0.649469 | 0.910262 | 1 | 3592 | tags=27%, list=15%, signal=31% |
| 0.305726 | 0.875257 | 0.616341 | 0.909877 | 1 | 3161 | tags=29%, list=13%, signal=34% |
| 0.37815 | 0.873751 | 0.703959 | 0.912408 | 1 | 5642 | tags=52%, list=24%, signal=68% |
| 0.260374 | 0.873305 | 0.699377 | 0.912457 | 1 | 3323 | tags=24%, list=14%, signal=27% |
| 0.315776 | 0.871717 | 0.696309 | 0.915191 | 1 | 5475 | tags=32%, list=23%, signal=42% |
| 0.264722 | 0.87108 | 0.769671 | 0.915629 | 1 | 7094 | tags=42%, list=30%, signal=60% |
| 0.296398 | 0.870814 | 0.675039 | 0.915354 | 1 | 5246 | tags=38%, list=22%, signal=49% |
| 0.306531 | 0.869963 | 0.606947 | 0.91626 | 1 | 4347 | tags=24%, list=19%, signal=29% |
| 0.22441 | 0.869757 | 0.798649 | 0.915774 | 1 | 4711 | tags=27%, list=20%, signal=34% |
| 0.302075 | 0.869033 | 0.6832 | 0.916529 | 1 | 4189 | tags=32%, list=18%, signal=39% |
| 0.325865 | 0.867702 | 0.590226 | 0.918653 | 1 | 5627 | tags=40%, list=24%, signal=52% |
| 0.271835 | 0.867653 | 0.600649 | 0.917878 | 1 | 5584 | tags=37%, list=24%, signal=48% |
| 0.425499 | 0.867557 | 0.624074 | 0.91716 | 1 | 6859 | tags=58%, list=29%, signal=82% |
| 0.370014 | 0.867469 | 0.626118 | 0.916433 | 1 | 5378 | tags=50%, list=23%, signal=65% |
| 0.318366 | 0.867455 | 0.611807 | 0.915575 | 1 | 5428 | tags=40%, list=23%, signal=52% |
| 0.31001 | 0.867105 | 0.651543 | 0.915489 | 1 | 2470 | tags=25%, list=11%, signal=28% |
| 0.267004 | 0.866732 | 0.728997 | 0.915356 | 1 | 5298 | tags=39%, list=23%, signal=50% |
| 0.282666 | 0.866243 | 0.641286 | 0.915546 | 1 | 5857 | tags=50%, list=25%, signal=67% |
| 0.391604 | 0.866023 | 0.609108 | 0.915138 | 1 | 4514 | tags=46%, list=19%, signal=57% |
| 0.310661 | 0.865676 | 0.582878 | 0.915031 | 1 | 4755 | tags=34%, list=20%, signal=42% |
| 0.270167 | 0.865484 | 0.729345 | 0.914593 | 1 | 2974 | tags=29%, list=13%, signal=33% |
| 0.333458 | 0.864825 | 0.627151 | 0.915103 | 1 | 7600 | tags=51%, list=32%, signal=76% |
| 0.254651 | 0.864161 | 0.735474 | 0.915738 | 1 | 7506 | tags=45%, list=32%, signal=66% |
| 0.266006 | 0.86414 | 0.691265 | 0.914901 | 1 | 6068 | tags=42%, list=26%, signal=57% |
| 0.26653 | 0.863693 | 0.6608 | 0.914928 | 1 | 4575 | tags=29%, list=20%, signal=36% |
| 0.323886 | 0.863686 | 0.68871 | 0.914068 | 1 | 5283 | tags=50%, list=23%, signal=65% |

| | | | | | | |
|---|---|---|---|---|---|---|
| 0.260631 | 0.863675 | 0.697324 | 0.913216 | 1 | 3195 | tags=23%, list=14%, signal=26% |
| 0.283878 | 0.863556 | 0.795082 | 0.912604 | 1 | 3082 | tags=34%, list=13%, signal=40% |
| 0.237307 | 0.86287 | 0.762295 | 0.913209 | 1 | 4237 | tags=25%, list=18%, signal=31% |
| 0.395757 | 0.862512 | 0.616387 | 0.913153 | 1 | 5020 | tags=47%, list=21%, signal=60% |
| 0.306819 | 0.861839 | 0.653916 | 0.913705 | 1 | 966 | tags=20%, list=4%, signal=20% |
| 0.378719 | 0.861811 | 0.731482 | 0.912928 | 1 | 6052 | tags=43%, list=26%, signal=59% |
| 0.30886 | 0.861657 | 0.7031 | 0.912411 | 1 | 5445 | tags=41%, list=23%, signal=53% |
| 0.23529 | 0.861404 | 0.720497 | 0.912123 | 1 | 3708 | tags=26%, list=16%, signal=31% |
| 0.258888 | 0.861119 | 0.70442 | 0.911889 | 1 | 5150 | tags=35%, list=22%, signal=44% |
| 0.263611 | 0.85989 | 0.70547 | 0.913608 | 1 | 5026 | tags=33%, list=21%, signal=42% |
| 0.328476 | 0.859781 | 0.620451 | 0.912995 | 1 | 7874 | tags=53%, list=34%, signal=79% |
| 0.313006 | 0.85973 | 0.690566 | 0.912251 | 1 | 6314 | tags=40%, list=27%, signal=55% |
| 0.257662 | 0.859122 | 0.71722 | 0.912638 | 1 | 2451 | tags=22%, list=10%, signal=24% |
| 0.341225 | 0.859058 | 0.65744 | 0.911918 | 1 | 5796 | tags=47%, list=25%, signal=62% |
| 0.270706 | 0.858949 | 0.756494 | 0.911325 | 1 | 3453 | tags=30%, list=15%, signal=35% |
| 0.208484 | 0.858523 | 0.876056 | 0.91138 | 1 | 5857 | tags=29%, list=25%, signal=39% |
| 0.236627 | 0.858069 | 0.810888 | 0.911403 | 1 | 5878 | tags=36%, list=25%, signal=48% |
| 0.250732 | 0.856806 | 0.803523 | 0.913256 | 1 | 4322 | tags=31%, list=18%, signal=38% |
| 0.349738 | 0.856619 | 0.642857 | 0.912825 | 1 | 4802 | tags=44%, list=21%, signal=55% |
| 0.343301 | 0.856145 | 0.669368 | 0.912908 | 1 | 5026 | tags=38%, list=21%, signal=48% |
| 0.315338 | 0.855993 | 0.704293 | 0.912346 | 1 | 3661 | tags=40%, list=16%, signal=47% |
| 0.340252 | 0.855696 | 0.641326 | 0.91216 | 1 | 5452 | tags=44%, list=23%, signal=57% |
| 0.24422 | 0.854199 | 0.768116 | 0.914403 | 1 | 7559 | tags=50%, list=32%, signal=74% |
| 0.258808 | 0.854189 | 0.809524 | 0.91357 | 1 | 438 | tags=12%, list=2%, signal=12% |
| 0.298184 | 0.854121 | 0.72619 | 0.912831 | 1 | 5857 | tags=33%, list=25%, signal=44% |
| 0.269182 | 0.853772 | 0.579848 | 0.912659 | 1 | 5671 | tags=36%, list=24%, signal=47% |
| 0.292123 | 0.853759 | 0.814077 | 0.911832 | 1 | 1884 | tags=24%, list=8%, signal=26% |
| 0.31761 | 0.853393 | 0.641682 | 0.911709 | 1 | 5923 | tags=41%, list=25%, signal=54% |
| 0.35288 | 0.852879 | 0.590226 | 0.912003 | 1 | 4388 | tags=36%, list=19%, signal=44% |
| 0.317618 | 0.852513 | 0.591241 | 0.911941 | 1 | 5612 | tags=45%, list=24%, signal=59% |
| 0.321478 | 0.852341 | 0.697436 | 0.91145 | 1 | 2597 | tags=33%, list=11%, signal=37% |
| 0.286835 | 0.852303 | 0.623352 | 0.910677 | 1 | 5760 | tags=41%, list=25%, signal=54% |
| 0.220004 | 0.851994 | 0.771341 | 0.910408 | 1 | 4546 | tags=25%, list=19%, signal=31% |
| 0.286465 | 0.851807 | 0.74359 | 0.909919 | 1 | 1187 | tags=14%, list=5%, signal=15% |
| 0.28562 | 0.851323 | 0.654412 | 0.910037 | 1 | 7998 | tags=52%, list=34%, signal=78% |
| 0.324306 | 0.851301 | 0.660142 | 0.909247 | 1 | 5857 | tags=43%, list=25%, signal=57% |
| 0.368428 | 0.851208 | 0.568047 | 0.908596 | 1 | 6167 | tags=43%, list=26%, signal=58% |
| 0.303094 | 0.850652 | 0.744224 | 0.908807 | 1 | 5060 | tags=33%, list=22%, signal=42% |
| 0.329457 | 0.850474 | 0.642593 | 0.908306 | 1 | 3944 | tags=34%, list=17%, signal=41% |
| 0.228328 | 0.849843 | 0.737303 | 0.908788 | 1 | 6416 | tags=34%, list=27%, signal=47% |
| 0.261232 | 0.849803 | 0.79483 | 0.908056 | 1 | 5397 | tags=33%, list=23%, signal=43% |
| 0.43601 | 0.848762 | 0.621377 | 0.909337 | 1 | 8059 | tags=60%, list=34%, signal=91% |
| 0.381513 | 0.847304 | 0.669935 | 0.91153 | 1 | 4591 | tags=35%, list=20%, signal=43% |
| 0.2803 | 0.846201 | 0.712251 | 0.912934 | 1 | 7448 | tags=43%, list=32%, signal=63% |
| 0.408646 | 0.845889 | 0.702417 | 0.912756 | 1 | 6315 | tags=58%, list=27%, signal=80% |
| 0.297723 | 0.845101 | 0.720749 | 0.913564 | 1 | 5868 | tags=44%, list=25%, signal=59% |
| 0.245883 | 0.844434 | 0.781437 | 0.914041 | 1 | 5882 | tags=44%, list=25%, signal=59% |
| 0.230168 | 0.844125 | 0.749594 | 0.91383 | 1 | 4640 | tags=27%, list=20%, signal=33% |
| 0.311004 | 0.844004 | 0.745066 | 0.913258 | 1 | 3835 | tags=26%, list=16%, signal=31% |
| 0.266518 | 0.84394 | 0.709122 | 0.912557 | 1 | 4277 | tags=29%, list=18%, signal=35% |

| | | | | | | |
|---|---|---|---|---|---|---|
| 0.33292 | 0.843763 | 0.6832 | 0.91205 | 1 | 6018 | tags=53%, list=26%, signal=71% |
| 0.284055 | 0.843392 | 0.75614 | 0.911877 | 1 | 3371 | tags=29%, list=14%, signal=33% |
| 0.314229 | 0.843245 | 0.684127 | 0.911406 | 1 | 5796 | tags=55%, list=25%, signal=73% |
| 0.376094 | 0.843124 | 0.684783 | 0.910802 | 1 | 5152 | tags=38%, list=22%, signal=48% |
| 0.274155 | 0.84202 | 0.791476 | 0.912096 | 1 | 3375 | tags=28%, list=14%, signal=33% |
| 0.318515 | 0.841671 | 0.659537 | 0.911947 | 1 | 4319 | tags=35%, list=18%, signal=43% |
| 0.349524 | 0.841349 | 0.631068 | 0.911749 | 1 | 2007 | tags=25%, list=9%, signal=27% |
| 0.365249 | 0.838961 | 0.621723 | 0.915801 | 1 | 6502 | tags=50%, list=28%, signal=69% |
| 0.346749 | 0.838914 | 0.661211 | 0.915074 | 1 | 5885 | tags=45%, list=25%, signal=60% |
| 0.264223 | 0.838559 | 0.76197 | 0.914932 | 1 | 3467 | tags=28%, list=15%, signal=33% |
| 0.275934 | 0.838502 | 0.828841 | 0.914258 | 1 | 4243 | tags=36%, list=18%, signal=44% |
| 0.292122 | 0.838363 | 0.721843 | 0.913682 | 1 | 1959 | tags=20%, list=8%, signal=22% |
| 0.226296 | 0.837687 | 0.854667 | 0.914137 | 1 | 5670 | tags=34%, list=24%, signal=44% |
| 0.329782 | 0.837583 | 0.657143 | 0.913599 | 1 | 3764 | tags=34%, list=16%, signal=40% |
| 0.3293 | 0.837158 | 0.742402 | 0.913636 | 1 | 253 | tags=15%, list=1%, signal=15% |
| 0.225392 | 0.837041 | 0.775036 | 0.913061 | 1 | 3583 | tags=23%, list=15%, signal=27% |
| 0.385148 | 0.836376 | 0.643275 | 0.913601 | 1 | 5020 | tags=42%, list=21%, signal=54% |
| 0.26491 | 0.836041 | 0.713826 | 0.913363 | 1 | 4332 | tags=30%, list=18%, signal=37% |
| 0.31781 | 0.835971 | 0.716561 | 0.912666 | 1 | 5696 | tags=35%, list=24%, signal=46% |
| 0.268559 | 0.835589 | 0.758621 | 0.91266 | 1 | 6185 | tags=40%, list=26%, signal=54% |
| 0.245211 | 0.835551 | 0.75 | 0.911928 | 1 | 5128 | tags=32%, list=22%, signal=41% |
| 0.235808 | 0.835064 | 0.732143 | 0.912003 | 1 | 5285 | tags=29%, list=23%, signal=37% |
| 0.337201 | 0.834265 | 0.715771 | 0.91264 | 1 | 382 | tags=21%, list=2%, signal=21% |
| 0.346384 | 0.834133 | 0.689524 | 0.912051 | 1 | 3602 | tags=37%, list=15%, signal=44% |
| 0.255719 | 0.83412 | 0.709677 | 0.911267 | 1 | 5857 | tags=37%, list=25%, signal=49% |
| 0.280914 | 0.833975 | 0.753582 | 0.910703 | 1 | 2601 | tags=24%, list=11%, signal=27% |
| 0.276232 | 0.833548 | 0.7616 | 0.910682 | 1 | 2738 | tags=27%, list=12%, signal=31% |
| 0.330011 | 0.8327 | 0.694915 | 0.911526 | 1 | 2921 | tags=31%, list=12%, signal=36% |
| 0.235241 | 0.832604 | 0.800664 | 0.910948 | 1 | 6282 | tags=38%, list=27%, signal=51% |
| 0.351029 | 0.830523 | 0.715909 | 0.914255 | 1 | 3737 | tags=38%, list=16%, signal=44% |
| 0.267773 | 0.829936 | 0.768982 | 0.914583 | 1 | 3710 | tags=27%, list=16%, signal=32% |
| 0.355384 | 0.829799 | 0.612245 | 0.914005 | 1 | 3764 | tags=38%, list=16%, signal=45% |
| 0.310948 | 0.828675 | 0.616105 | 0.915301 | 1 | 5644 | tags=41%, list=24%, signal=53% |
| 0.263054 | 0.827829 | 0.730129 | 0.916107 | 1 | 3280 | tags=24%, list=14%, signal=28% |
| 0.26935 | 0.827775 | 0.791111 | 0.915417 | 1 | 4816 | tags=35%, list=21%, signal=44% |
| 0.235233 | 0.827469 | 0.727586 | 0.915141 | 1 | 1485 | tags=11%, list=6%, signal=12% |
| 0.230519 | 0.827147 | 0.791379 | 0.91501 | 1 | 4519 | tags=28%, list=19%, signal=34% |
| 0.330082 | 0.827119 | 0.664336 | 0.914248 | 1 | 3375 | tags=29%, list=14%, signal=33% |
| 0.340583 | 0.825573 | 0.617702 | 0.91631 | 1 | 5547 | tags=40%, list=24%, signal=52% |
| 0.283489 | 0.824273 | 0.766423 | 0.917911 | 1 | 5673 | tags=42%, list=24%, signal=55% |
| 0.294825 | 0.823714 | 0.688925 | 0.918187 | 1 | 4559 | tags=39%, list=19%, signal=48% |
| 0.271271 | 0.823478 | 0.765958 | 0.917834 | 1 | 2192 | tags=22%, list=9%, signal=24% |
| 0.307187 | 0.822932 | 0.744373 | 0.918048 | 1 | 697 | tags=12%, list=3%, signal=12% |
| 0.240533 | 0.821839 | 0.739583 | 0.919208 | 1 | 4800 | tags=33%, list=20%, signal=41% |
| 0.270525 | 0.821738 | 0.819005 | 0.918584 | 1 | 4996 | tags=40%, list=21%, signal=51% |
| 0.289156 | 0.821695 | 0.711039 | 0.917847 | 1 | 4972 | tags=30%, list=21%, signal=38% |
| 0.269046 | 0.820748 | 0.790551 | 0.918717 | 1 | 6321 | tags=45%, list=27%, signal=62% |
| 0.467806 | 0.819889 | 0.699812 | 0.919506 | 1 | 7858 | tags=81%, list=34%, signal=122% |
| 0.282946 | 0.81855 | 0.700717 | 0.921031 | 1 | 7474 | tags=53%, list=32%, signal=78% |
| 0.319724 | 0.818293 | 0.736091 | 0.920692 | 1 | 3975 | tags=33%, list=17%, signal=40% |

| | | | | | | |
|---|---|---|---|---|---|---|
| 0.256524 | 0.816826 | 0.789292 | 0.922554 | 1 | 3704 | tags=29%, list=16%, signal=34% |
| 0.350431 | 0.816647 | 0.801399 | 0.922058 | 1 | 4404 | tags=47%, list=19%, signal=58% |
| 0.333846 | 0.81642 | 0.722936 | 0.921654 | 1 | 4227 | tags=44%, list=18%, signal=53% |
| 0.234198 | 0.816191 | 0.813665 | 0.921295 | 1 | 2966 | tags=23%, list=13%, signal=26% |
| 0.363888 | 0.814062 | 0.666667 | 0.92434 | 1 | 5846 | tags=59%, list=25%, signal=79% |
| 0.270551 | 0.814003 | 0.8 | 0.92365 | 1 | 5608 | tags=41%, list=24%, signal=54% |
| 0.338791 | 0.813431 | 0.627451 | 0.923818 | 1 | 7008 | tags=54%, list=30%, signal=77% |
| 0.364357 | 0.813075 | 0.708075 | 0.923628 | 1 | 2245 | tags=31%, list=10%, signal=35% |
| 0.261792 | 0.812665 | 0.793781 | 0.923531 | 1 | 5777 | tags=45%, list=25%, signal=59% |
| 0.33642 | 0.812399 | 0.722034 | 0.923227 | 1 | 7774 | tags=59%, list=33%, signal=88% |
| 0.258229 | 0.810715 | 0.747069 | 0.925528 | 1 | 4726 | tags=34%, list=20%, signal=43% |
| 0.229909 | 0.809822 | 0.841018 | 0.926333 | 1 | 3582 | tags=23%, list=15%, signal=27% |
| 0.276105 | 0.809701 | 0.822504 | 0.925758 | 1 | 3777 | tags=32%, list=16%, signal=38% |
| 0.274148 | 0.809612 | 0.77812 | 0.925152 | 1 | 3293 | tags=28%, list=14%, signal=32% |
| 0.261572 | 0.809161 | 0.863014 | 0.925156 | 1 | 2601 | tags=20%, list=11%, signal=22% |
| 0.254159 | 0.80901 | 0.748175 | 0.924634 | 1 | 5890 | tags=32%, list=25%, signal=42% |
| 0.254472 | 0.808097 | 0.759921 | 0.925521 | 1 | 3389 | tags=25%, list=14%, signal=29% |
| 0.236374 | 0.806699 | 0.874797 | 0.927077 | 1 | 7439 | tags=48%, list=32%, signal=70% |
| 0.240413 | 0.806546 | 0.825352 | 0.926556 | 1 | 3817 | tags=24%, list=16%, signal=28% |
| 0.297661 | 0.806177 | 0.788732 | 0.926479 | 1 | 2594 | tags=20%, list=11%, signal=22% |
| 0.263645 | 0.804148 | 0.887324 | 0.929381 | 1 | 6112 | tags=41%, list=26%, signal=55% |
| 0.35619 | 0.804122 | 0.702448 | 0.928646 | 1 | 7635 | tags=45%, list=33%, signal=66% |
| 0.291593 | 0.804018 | 0.80102 | 0.92806 | 1 | 4208 | tags=31%, list=18%, signal=38% |
| 0.280465 | 0.803727 | 0.639042 | 0.927813 | 1 | 5353 | tags=36%, list=23%, signal=46% |
| 0.254917 | 0.801218 | 0.666102 | 0.931369 | 1 | 3672 | tags=22%, list=16%, signal=26% |
| 0.278957 | 0.800319 | 0.748175 | 0.932102 | 1 | 3381 | tags=26%, list=14%, signal=30% |
| 0.245368 | 0.799698 | 0.832861 | 0.932321 | 1 | 5673 | tags=39%, list=24%, signal=52% |
| 0.310328 | 0.799506 | 0.794258 | 0.931889 | 1 | 926 | tags=13%, list=4%, signal=14% |
| 0.32298 | 0.799174 | 0.76507 | 0.931638 | 1 | 642 | tags=12%, list=3%, signal=12% |
| 0.308661 | 0.798818 | 0.802432 | 0.93144 | 1 | 2518 | tags=28%, list=11%, signal=31% |
| 0.402523 | 0.798597 | 0.714822 | 0.931055 | 1 | 5020 | tags=37%, list=21%, signal=46% |
| 0.286748 | 0.797892 | 0.816529 | 0.931551 | 1 | 5696 | tags=39%, list=24%, signal=52% |
| 0.248529 | 0.797749 | 0.784588 | 0.930983 | 1 | 6282 | tags=36%, list=27%, signal=49% |
| 0.359368 | 0.797684 | 0.648936 | 0.930331 | 1 | 7082 | tags=63%, list=30%, signal=90% |
| 0.322688 | 0.797082 | 0.763889 | 0.930563 | 1 | 2286 | tags=25%, list=10%, signal=28% |
| 0.281386 | 0.797024 | 0.721854 | 0.929877 | 1 | 4248 | tags=36%, list=18%, signal=44% |
| 0.401202 | 0.796905 | 0.75 | 0.929304 | 1 | 8282 | tags=67%, list=35%, signal=103% |
| 0.301454 | 0.796196 | 0.672535 | 0.929711 | 1 | 4900 | tags=39%, list=21%, signal=49% |
| 0.296383 | 0.795292 | 0.829755 | 0.9305 | 1 | 5270 | tags=42%, list=23%, signal=54% |
| 0.251841 | 0.794864 | 0.716607 | 0.930466 | 1 | 5939 | tags=38%, list=25%, signal=51% |
| 0.254292 | 0.794526 | 0.683616 | 0.930231 | 1 | 5349 | tags=32%, list=23%, signal=41% |
| 0.356034 | 0.793405 | 0.645098 | 0.931316 | 1 | 5428 | tags=43%, list=23%, signal=56% |
| 0.283078 | 0.792049 | 0.791878 | 0.932767 | 1 | 5777 | tags=28%, list=25%, signal=37% |
| 0.364913 | 0.792044 | 0.713761 | 0.931998 | 1 | 4797 | tags=29%, list=20%, signal=37% |
| 0.225751 | 0.790823 | 0.818038 | 0.933244 | 1 | 5857 | tags=39%, list=25%, signal=52% |
| 0.285105 | 0.790711 | 0.859568 | 0.932658 | 1 | 6655 | tags=56%, list=28%, signal=78% |
| 0.239823 | 0.790578 | 0.882726 | 0.932098 | 1 | 5786 | tags=35%, list=25%, signal=47% |
| 0.228653 | 0.790258 | 0.734993 | 0.93193 | 1 | 4708 | tags=31%, list=20%, signal=38% |
| 0.32033 | 0.790033 | 0.810594 | 0.931552 | 1 | 3398 | tags=26%, list=15%, signal=31% |
| 0.252185 | 0.789971 | 0.782759 | 0.930898 | 1 | 2054 | tags=19%, list=9%, signal=21% |

| | | | | | | |
|---|---|---|---|---|---|---|
| 0.255851 | 0.789257 | 0.798987 | 0.93128 | 1 | 7362 | tags=52%, list=31%, signal=75% |
| 0.324485 | 0.78819 | 0.721368 | 0.932249 | 1 | 1655 | tags=20%, list=7%, signal=22% |
| 0.234699 | 0.787445 | 0.877947 | 0.932652 | 1 | 6213 | tags=36%, list=27%, signal=49% |
| 0.251223 | 0.785172 | 0.75576 | 0.935581 | 1 | 5909 | tags=38%, list=25%, signal=51% |
| 0.288905 | 0.784834 | 0.802023 | 0.935369 | 1 | 5593 | tags=48%, list=24%, signal=63% |
| 0.21708 | 0.783098 | 0.87092 | 0.937368 | 1 | 3371 | tags=20%, list=14%, signal=24% |
| 0.225738 | 0.783093 | 0.86014 | 0.936621 | 1 | 5882 | tags=40%, list=25%, signal=53% |
| 0.319073 | 0.781273 | 0.750831 | 0.938705 | 1 | 5717 | tags=50%, list=24%, signal=66% |
| 0.263395 | 0.780888 | 0.827869 | 0.938511 | 1 | 4860 | tags=36%, list=21%, signal=46% |
| 0.346982 | 0.780428 | 0.7061 | 0.938481 | 1 | 4739 | tags=45%, list=20%, signal=56% |
| 0.292183 | 0.779929 | 0.720268 | 0.938552 | 1 | 1043 | tags=14%, list=4%, signal=15% |
| 0.229845 | 0.778742 | 0.790105 | 0.939656 | 1 | 6436 | tags=44%, list=27%, signal=60% |
| 0.265741 | 0.77723 | 0.845277 | 0.941305 | 1 | 3632 | tags=26%, list=16%, signal=31% |
| 0.30146 | 0.776954 | 0.77621 | 0.940933 | 1 | 2739 | tags=22%, list=12%, signal=25% |
| 0.231254 | 0.774845 | 0.883683 | 0.943445 | 1 | 3276 | tags=21%, list=14%, signal=25% |
| 0.242355 | 0.774451 | 0.798013 | 0.943238 | 1 | 5919 | tags=40%, list=25%, signal=53% |
| 0.262108 | 0.774111 | 0.741936 | 0.943018 | 1 | 6680 | tags=36%, list=29%, signal=50% |
| 0.372941 | 0.772633 | 0.808349 | 0.94461 | 1 | 5396 | tags=41%, list=23%, signal=54% |
| 0.300926 | 0.772433 | 0.773109 | 0.944124 | 1 | 7212 | tags=53%, list=31%, signal=77% |
| 0.237508 | 0.772026 | 0.777778 | 0.944003 | 1 | 4319 | tags=23%, list=18%, signal=28% |
| 0.287512 | 0.771741 | 0.790997 | 0.943668 | 1 | 4217 | tags=36%, list=18%, signal=43% |
| 0.221505 | 0.771725 | 0.791946 | 0.942927 | 1 | 4766 | tags=27%, list=20%, signal=34% |
| 0.338079 | 0.771367 | 0.722846 | 0.942694 | 1 | 5020 | tags=34%, list=21%, signal=44% |
| 0.350561 | 0.77041 | 0.725379 | 0.943447 | 1 | 5020 | tags=34%, list=21%, signal=43% |
| 0.249122 | 0.770209 | 0.862629 | 0.942989 | 1 | 2664 | tags=20%, list=11%, signal=23% |
| 0.280924 | 0.77006 | 0.834671 | 0.94246 | 1 | 7324 | tags=61%, list=31%, signal=89% |
| 0.249973 | 0.76937 | 0.696915 | 0.94276 | 1 | 5718 | tags=36%, list=24%, signal=46% |
| 0.271913 | 0.768154 | 0.785032 | 0.943894 | 1 | 4514 | tags=36%, list=19%, signal=45% |
| 0.308711 | 0.767477 | 0.78831 | 0.94419 | 1 | 3109 | tags=34%, list=13%, signal=39% |
| 0.283628 | 0.76736 | 0.831547 | 0.943595 | 1 | 466 | tags=11%, list=2%, signal=11% |
| 0.276486 | 0.766383 | 0.572954 | 0.944268 | 1 | 2114 | tags=21%, list=9%, signal=23% |
| 0.309636 | 0.766085 | 0.696462 | 0.94393 | 1 | 4665 | tags=44%, list=20%, signal=55% |
| 0.283683 | 0.76608 | 0.865478 | 0.943185 | 1 | 5533 | tags=37%, list=24%, signal=48% |
| 0.296308 | 0.765532 | 0.849722 | 0.943286 | 1 | 2577 | tags=25%, list=11%, signal=28% |
| 0.267847 | 0.765439 | 0.83913 | 0.942656 | 1 | 4478 | tags=27%, list=19%, signal=33% |
| 0.318072 | 0.765233 | 0.766304 | 0.942192 | 1 | 7718 | tags=43%, list=33%, signal=65% |
| 0.229595 | 0.764887 | 0.927497 | 0.94198 | 1 | 5768 | tags=32%, list=25%, signal=42% |
| 0.301698 | 0.764041 | 0.795737 | 0.942498 | 1 | 4673 | tags=40%, list=20%, signal=50% |
| 0.31153 | 0.763562 | 0.767176 | 0.942403 | 1 | 2470 | tags=30%, list=11%, signal=34% |
| 0.23484 | 0.763502 | 0.871404 | 0.941746 | 1 | 2966 | tags=19%, list=13%, signal=22% |
| 0.244707 | 0.762639 | 0.886115 | 0.942213 | 1 | 1984 | tags=18%, list=8%, signal=20% |
| 0.269943 | 0.762017 | 0.726957 | 0.94232 | 1 | 3125 | tags=22%, list=13%, signal=26% |
| 0.21831 | 0.761421 | 0.973793 | 0.942425 | 1 | 4767 | tags=28%, list=20%, signal=35% |
| 0.316865 | 0.761009 | 0.794682 | 0.942272 | 1 | 4847 | tags=38%, list=21%, signal=47% |
| 0.326763 | 0.76081 | 0.737037 | 0.941805 | 1 | 5857 | tags=59%, list=25%, signal=78% |
| 0.285693 | 0.760703 | 0.769912 | 0.94118 | 1 | 5133 | tags=41%, list=22%, signal=53% |
| 0.251174 | 0.760398 | 0.850407 | 0.940884 | 1 | 4065 | tags=26%, list=17%, signal=31% |
| 0.296545 | 0.759975 | 0.756055 | 0.940789 | 1 | 2662 | tags=27%, list=11%, signal=30% |
| 0.321213 | 0.759355 | 0.713208 | 0.940901 | 1 | 3764 | tags=35%, list=16%, signal=42% |
| 0.279827 | 0.75883 | 0.773124 | 0.940859 | 1 | 5595 | tags=37%, list=24%, signal=49% |

| | | | | | | |
|---|---|---|---|---|---|---|
| 0.288352 | 0.757162 | 0.789784 | 0.942405 | 1 | 3640 | tags=25%, list=16%, signal=30% |
| 0.260097 | 0.756919 | 0.861429 | 0.941963 | 1 | 2594 | tags=15%, list=11%, signal=17% |
| 0.30608 | 0.756258 | 0.723443 | 0.942093 | 1 | 3764 | tags=35%, list=16%, signal=41% |
| 0.28683 | 0.755266 | 0.704331 | 0.942763 | 1 | 5468 | tags=38%, list=23%, signal=49% |
| 0.276921 | 0.754113 | 0.756906 | 0.943516 | 1 | 2859 | tags=23%, list=12%, signal=27% |
| 0.28459 | 0.75405 | 0.817658 | 0.94285 | 1 | 3535 | tags=33%, list=15%, signal=39% |
| 0.214508 | 0.753677 | 0.917492 | 0.942571 | 1 | 4038 | tags=22%, list=17%, signal=27% |
| 0.236989 | 0.753663 | 0.764706 | 0.941848 | 1 | 6117 | tags=35%, list=26%, signal=48% |
| 0.363275 | 0.753349 | 0.742215 | 0.941568 | 1 | 5246 | tags=53%, list=22%, signal=68% |
| 0.265293 | 0.752553 | 0.870827 | 0.941919 | 1 | 4683 | tags=30%, list=20%, signal=37% |
| 0.262709 | 0.752028 | 0.887805 | 0.941845 | 1 | 4075 | tags=22%, list=17%, signal=27% |
| 0.287656 | 0.751533 | 0.829528 | 0.941758 | 1 | 5379 | tags=53%, list=23%, signal=69% |
| 0.266926 | 0.75146 | 0.874063 | 0.941107 | 1 | 3048 | tags=23%, list=13%, signal=26% |
| 0.240422 | 0.750741 | 0.953125 | 0.94136 | 1 | 5315 | tags=30%, list=23%, signal=39% |
| 0.24236 | 0.75061 | 0.821667 | 0.940776 | 1 | 3337 | tags=22%, list=14%, signal=26% |
| 0.239646 | 0.750199 | 0.871017 | 0.940593 | 1 | 5584 | tags=31%, list=24%, signal=41% |
| 0.315808 | 0.748235 | 0.768473 | 0.942438 | 1 | 2832 | tags=20%, list=12%, signal=23% |
| 0.282167 | 0.747968 | 0.791209 | 0.942094 | 1 | 8091 | tags=47%, list=35%, signal=71% |
| 0.230473 | 0.745574 | 0.910334 | 0.944554 | 1 | 3005 | tags=19%, list=13%, signal=21% |
| 0.231133 | 0.745392 | 0.897674 | 0.94402 | 1 | 5910 | tags=37%, list=25%, signal=49% |
| 0.285852 | 0.74535 | 0.731076 | 0.943351 | 1 | 6132 | tags=43%, list=26%, signal=58% |
| 0.278812 | 0.744461 | 0.822161 | 0.943845 | 1 | 4108 | tags=26%, list=18%, signal=32% |
| 0.212148 | 0.744186 | 0.938211 | 0.94349 | 1 | 6164 | tags=36%, list=26%, signal=48% |
| 0.285841 | 0.744011 | 0.824773 | 0.942971 | 1 | 6304 | tags=33%, list=27%, signal=46% |
| 0.266644 | 0.7429 | 0.889695 | 0.943545 | 1 | 7335 | tags=53%, list=31%, signal=77% |
| 0.307207 | 0.742548 | 0.733962 | 0.943248 | 1 | 3764 | tags=36%, list=16%, signal=42% |
| 0.209422 | 0.741903 | 0.947458 | 0.94333 | 1 | 6416 | tags=35%, list=27%, signal=48% |
| 0.257927 | 0.741767 | 0.85913 | 0.942738 | 1 | 3355 | tags=23%, list=14%, signal=27% |
| 0.218701 | 0.74124 | 0.9 | 0.942587 | 1 | 6670 | tags=38%, list=28%, signal=53% |
| 0.216649 | 0.741071 | 0.934426 | 0.942048 | 1 | 5595 | tags=26%, list=24%, signal=34% |
| 0.227509 | 0.740351 | 0.889923 | 0.9422 | 1 | 5234 | tags=32%, list=22%, signal=41% |
| 0.229915 | 0.739886 | 0.790441 | 0.941997 | 1 | 5361 | tags=33%, list=23%, signal=43% |
| 0.314243 | 0.739818 | 0.852649 | 0.941351 | 1 | 8403 | tags=41%, list=36%, signal=64% |
| 0.301212 | 0.739665 | 0.768382 | 0.940814 | 1 | 2654 | tags=26%, list=11%, signal=29% |
| 0.316574 | 0.738421 | 0.738095 | 0.941562 | 1 | 4345 | tags=39%, list=19%, signal=48% |
| 0.282764 | 0.738014 | 0.70674 | 0.941306 | 1 | 5777 | tags=44%, list=25%, signal=58% |
| 0.219044 | 0.737278 | 0.870432 | 0.941541 | 1 | 5859 | tags=32%, list=25%, signal=42% |
| 0.272555 | 0.735735 | 0.853333 | 0.94279 | 1 | 6866 | tags=35%, list=29%, signal=50% |
| 0.263726 | 0.733524 | 0.820809 | 0.944633 | 1 | 5715 | tags=36%, list=24%, signal=48% |
| 0.305218 | 0.733079 | 0.755459 | 0.944441 | 1 | 4958 | tags=33%, list=21%, signal=42% |
| 0.25343 | 0.732071 | 0.876068 | 0.944933 | 1 | 7759 | tags=46%, list=33%, signal=68% |
| 0.29387 | 0.731667 | 0.801105 | 0.944663 | 1 | 1855 | tags=17%, list=8%, signal=19% |
| 0.338645 | 0.730788 | 0.723776 | 0.944997 | 1 | 7082 | tags=53%, list=30%, signal=76% |
| 0.306738 | 0.730125 | 0.728782 | 0.945094 | 1 | 4802 | tags=40%, list=21%, signal=50% |
| 0.27246 | 0.729556 | 0.8646 | 0.945005 | 1 | 3220 | tags=23%, list=14%, signal=27% |
| 0.22329 | 0.728903 | 0.841818 | 0.945054 | 1 | 5547 | tags=34%, list=24%, signal=44% |
| 0.265397 | 0.726022 | 0.927808 | 0.947726 | 1 | 5593 | tags=38%, list=24%, signal=49% |
| 0.23269 | 0.725617 | 0.926563 | 0.947452 | 1 | 1442 | tags=16%, list=6%, signal=17% |
| 0.334555 | 0.725523 | 0.850556 | 0.946819 | 1 | 6983 | tags=40%, list=30%, signal=57% |
| 0.285915 | 0.725335 | 0.776062 | 0.946279 | 1 | 5869 | tags=48%, list=25%, signal=64% |

| | | | | | | |
|---|---|---|---|---|---|---|
| 0.299316 | 0.72432 | 0.822464 | 0.946757 | 1 | 8091 | tags=59%, list=35%, signal=90% |
| 0.312752 | 0.723953 | 0.833055 | 0.946446 | 1 | 1542 | tags=20%, list=7%, signal=21% |
| 0.211457 | 0.723518 | 0.926761 | 0.946292 | 1 | 4534 | tags=28%, list=19%, signal=34% |
| 0.255761 | 0.723003 | 0.929217 | 0.946134 | 1 | 5203 | tags=33%, list=22%, signal=43% |
| 0.284367 | 0.722958 | 0.855446 | 0.945447 | 1 | 3737 | tags=32%, list=16%, signal=38% |
| 0.278767 | 0.721888 | 0.831826 | 0.945966 | 1 | 6336 | tags=41%, list=27%, signal=56% |
| 0.220639 | 0.721735 | 0.825704 | 0.945405 | 1 | 5910 | tags=35%, list=25%, signal=46% |
| 0.277144 | 0.721115 | 0.867669 | 0.945315 | 1 | 5922 | tags=48%, list=25%, signal=64% |
| 0.249477 | 0.719436 | 0.838583 | 0.946406 | 1 | 4347 | tags=25%, list=19%, signal=31% |
| 0.296347 | 0.719042 | 0.795411 | 0.946139 | 1 | 3764 | tags=33%, list=16%, signal=39% |
| 0.234802 | 0.718859 | 0.865835 | 0.945613 | 1 | 4672 | tags=29%, list=20%, signal=36% |
| 0.324252 | 0.718642 | 0.9046 | 0.945149 | 1 | 6336 | tags=47%, list=27%, signal=64% |
| 0.293967 | 0.718263 | 0.749042 | 0.944847 | 1 | 3764 | tags=27%, list=16%, signal=32% |
| 0.285191 | 0.718051 | 0.827709 | 0.944414 | 1 | 6094 | tags=36%, list=26%, signal=49% |
| 0.274209 | 0.717877 | 0.867508 | 0.943902 | 1 | 7965 | tags=60%, list=34%, signal=91% |
| 0.334564 | 0.717368 | 0.83 | 0.943761 | 1 | 5847 | tags=63%, list=25%, signal=84% |
| 0.263126 | 0.715928 | 0.821218 | 0.944727 | 1 | 3897 | tags=25%, list=17%, signal=30% |
| 0.248477 | 0.715213 | 0.906143 | 0.944859 | 1 | 4190 | tags=30%, list=18%, signal=37% |
| 0.219524 | 0.711375 | 0.935024 | 0.948007 | 1 | 6381 | tags=38%, list=27%, signal=53% |
| 0.29447 | 0.709771 | 0.776557 | 0.948935 | 1 | 3764 | tags=33%, list=16%, signal=40% |
| 0.262598 | 0.707918 | 0.687037 | 0.950063 | 1 | 5834 | tags=39%, list=25%, signal=51% |
| 0.225552 | 0.705613 | 0.923211 | 0.951683 | 1 | 5911 | tags=33%, list=25%, signal=44% |
| 0.257963 | 0.705182 | 0.875385 | 0.951414 | 1 | 4929 | tags=44%, list=21%, signal=55% |
| 0.261966 | 0.703682 | 0.928685 | 0.952158 | 1 | 5131 | tags=33%, list=22%, signal=43% |
| 0.247848 | 0.703265 | 0.844794 | 0.951826 | 1 | 5857 | tags=43%, list=25%, signal=57% |
| 0.25064 | 0.70276 | 0.881557 | 0.951606 | 1 | 4352 | tags=26%, list=19%, signal=32% |
| 0.213015 | 0.701553 | 0.973134 | 0.952099 | 1 | 5671 | tags=33%, list=24%, signal=43% |
| 0.281876 | 0.701298 | 0.889621 | 0.951637 | 1 | 4008 | tags=30%, list=17%, signal=36% |
| 0.23239 | 0.701254 | 0.954167 | 0.950965 | 1 | 4217 | tags=28%, list=18%, signal=34% |
| 0.220569 | 0.699323 | 0.813528 | 0.952083 | 1 | 5760 | tags=32%, list=25%, signal=42% |
| 0.243889 | 0.699045 | 0.881024 | 0.951637 | 1 | 6316 | tags=42%, list=27%, signal=58% |
| 0.220526 | 0.69806 | 0.88547 | 0.951895 | 1 | 3560 | tags=25%, list=15%, signal=29% |
| 0.259852 | 0.694647 | 0.896552 | 0.954466 | 1 | 1948 | tags=19%, list=8%, signal=20% |
| 0.258527 | 0.6946 | 0.763986 | 0.953799 | 1 | 8033 | tags=58%, list=34%, signal=89% |
| 0.274698 | 0.694574 | 0.854938 | 0.953108 | 1 | 6304 | tags=40%, list=27%, signal=54% |
| 0.318688 | 0.694215 | 0.769674 | 0.952735 | 1 | 5020 | tags=31%, list=21%, signal=40% |
| 0.269958 | 0.692371 | 0.826241 | 0.953725 | 1 | 4145 | tags=35%, list=18%, signal=42% |
| 0.286484 | 0.691538 | 0.863139 | 0.953735 | 1 | 3632 | tags=27%, list=16%, signal=32% |
| 0.247719 | 0.691518 | 0.880783 | 0.953053 | 1 | 3871 | tags=22%, list=17%, signal=27% |
| 0.251356 | 0.688287 | 0.913997 | 0.955275 | 1 | 5787 | tags=38%, list=25%, signal=51% |
| 0.269189 | 0.688149 | 0.950213 | 0.954707 | 1 | 5379 | tags=35%, list=23%, signal=45% |
| 0.279914 | 0.688022 | 0.858716 | 0.954121 | 1 | 7107 | tags=50%, list=30%, signal=72% |
| 0.200507 | 0.687552 | 0.960227 | 0.953819 | 1 | 7206 | tags=43%, list=31%, signal=62% |
| 0.26486 | 0.687534 | 0.856867 | 0.953117 | 1 | 3368 | tags=25%, list=14%, signal=29% |
| 0.218262 | 0.687395 | 0.980029 | 0.952541 | 1 | 6761 | tags=35%, list=29%, signal=49% |
| 0.239242 | 0.685455 | 0.890388 | 0.95361 | 1 | 2470 | tags=20%, list=11%, signal=22% |
| 0.28893 | 0.68338 | 0.854962 | 0.954635 | 1 | 6440 | tags=41%, list=28%, signal=57% |
| 0.291366 | 0.681201 | 0.817308 | 0.955886 | 1 | 4802 | tags=39%, list=21%, signal=49% |
| 0.31204 | 0.676244 | 0.843985 | 0.959204 | 1 | 6273 | tags=44%, list=27%, signal=61% |
| 0.28007 | 0.675879 | 0.813264 | 0.958837 | 1 | 4959 | tags=39%, list=21%, signal=49% |

| | | | | | | |
|---|---|---|---|---|---|---|
| 0.24457 | 0.674935 | 0.838889 | 0.958927 | 1 | 4532 | tags=31%, list=19%, signal=38% |
| 0.197209 | 0.670271 | 0.970968 | 0.96184 | 1 | 5537 | tags=21%, list=24%, signal=28% |
| 0.221841 | 0.669228 | 0.89781 | 0.961874 | 1 | 3897 | tags=23%, list=17%, signal=28% |
| 0.285109 | 0.667501 | 0.898551 | 0.96244 | 1 | 6960 | tags=38%, list=30%, signal=54% |
| 0.317746 | 0.666361 | 0.845238 | 0.962637 | 1 | 5847 | tags=55%, list=25%, signal=72% |
| 0.302996 | 0.665577 | 0.849315 | 0.962472 | 1 | 5869 | tags=59%, list=25%, signal=78% |
| 0.262728 | 0.662065 | 0.893155 | 0.964385 | 1 | 3481 | tags=29%, list=15%, signal=35% |
| 0.253739 | 0.65598 | 0.871143 | 0.967835 | 1 | 4959 | tags=34%, list=21%, signal=43% |
| 0.217174 | 0.655955 | 0.983146 | 0.967144 | 1 | 7461 | tags=46%, list=32%, signal=68% |
| 0.309891 | 0.654958 | 0.806273 | 0.967076 | 1 | 2033 | tags=27%, list=9%, signal=30% |
| 0.245123 | 0.654366 | 0.894928 | 0.966794 | 1 | 7116 | tags=39%, list=30%, signal=56% |
| 0.285609 | 0.652619 | 0.777989 | 0.967231 | 1 | 5516 | tags=38%, list=24%, signal=49% |
| 0.327498 | 0.652534 | 0.839458 | 0.966578 | 1 | 4755 | tags=42%, list=20%, signal=53% |
| 0.283991 | 0.650749 | 0.824299 | 0.967106 | 1 | 4603 | tags=25%, list=20%, signal=31% |
| 0.231535 | 0.649568 | 0.954041 | 0.96712 | 1 | 2902 | tags=21%, list=12%, signal=23% |
| 0.297532 | 0.648073 | 0.770093 | 0.967386 | 1 | 7205 | tags=58%, list=31%, signal=84% |
| 0.26754 | 0.640017 | 0.744059 | 0.971625 | 1 | 8089 | tags=61%, list=35%, signal=93% |
| 0.24133 | 0.639389 | 0.940154 | 0.97131 | 1 | 5501 | tags=38%, list=23%, signal=49% |
| 0.208087 | 0.638152 | 0.985185 | 0.971317 | 1 | 6808 | tags=42%, list=29%, signal=59% |
| 0.261541 | 0.629276 | 0.849057 | 0.975621 | 1 | 8050 | tags=55%, list=34%, signal=84% |
| 0.239583 | 0.628051 | 0.932526 | 0.975586 | 1 | 7984 | tags=56%, list=34%, signal=85% |
| 0.212495 | 0.627006 | 0.961268 | 0.975462 | 1 | 8413 | tags=51%, list=36%, signal=80% |
| 0.243294 | 0.62334 | 0.955385 | 0.976711 | 1 | 7372 | tags=51%, list=31%, signal=74% |
| 0.252554 | 0.623 | 0.954466 | 0.976173 | 1 | 8692 | tags=47%, list=37%, signal=75% |
| 0.267767 | 0.622642 | 0.874396 | 0.975662 | 1 | 6053 | tags=38%, list=26%, signal=51% |
| 0.249957 | 0.618873 | 0.880309 | 0.976808 | 1 | 3435 | tags=23%, list=15%, signal=27% |
| 0.223936 | 0.618686 | 0.950368 | 0.976169 | 1 | 6612 | tags=32%, list=28%, signal=45% |
| 0.186 | 0.616864 | 0.963855 | 0.976346 | 1 | 5620 | tags=22%, list=24%, signal=29% |
| 0.258347 | 0.616837 | 0.884393 | 0.975648 | 1 | 5861 | tags=39%, list=25%, signal=52% |
| 0.232968 | 0.616501 | 0.83054 | 0.975106 | 1 | 5745 | tags=36%, list=25%, signal=48% |
| 0.309521 | 0.612859 | 0.794495 | 0.976068 | 1 | 8050 | tags=61%, list=34%, signal=92% |
| 0.256746 | 0.611423 | 0.878613 | 0.975978 | 1 | 5752 | tags=38%, list=25%, signal=51% |
| 0.204515 | 0.61017 | 0.983283 | 0.975866 | 1 | 5389 | tags=27%, list=23%, signal=35% |
| 0.224691 | 0.610042 | 0.967407 | 0.975219 | 1 | 6876 | tags=42%, list=29%, signal=59% |
| 0.238099 | 0.607946 | 0.960568 | 0.975405 | 1 | 6537 | tags=32%, list=28%, signal=44% |
| 0.20293 | 0.601727 | 0.850746 | 0.977338 | 1 | 5601 | tags=32%, list=24%, signal=42% |
| 0.230992 | 0.601306 | 0.863884 | 0.976811 | 1 | 6004 | tags=40%, list=26%, signal=54% |
| 0.233452 | 0.596636 | 0.858238 | 0.977901 | 1 | 5880 | tags=36%, list=25%, signal=48% |
| 0.285607 | 0.590302 | 0.842294 | 0.97965 | 1 | 3825 | tags=35%, list=16%, signal=42% |
| 0.231717 | 0.588781 | 0.969871 | 0.979457 | 1 | 7343 | tags=53%, list=31%, signal=77% |
| 0.248543 | 0.583106 | 0.8625 | 0.980669 | 1 | 7269 | tags=55%, list=31%, signal=79% |
| 0.211245 | 0.570882 | 0.89942 | 0.983594 | 1 | 4347 | tags=21%, list=19%, signal=26% |
| 0.270914 | 0.569559 | 0.927644 | 0.983266 | 1 | 4926 | tags=40%, list=21%, signal=51% |
| 0.232894 | 0.564349 | 0.961938 | 0.983973 | 1 | 4888 | tags=19%, list=21%, signal=24% |
| 0.192931 | 0.526211 | 0.928972 | 0.991542 | 1 | 4347 | tags=19%, list=19%, signal=24% |
| 0.289345 | 0.513048 | 0.899054 | 0.99288 | 1 | 7933 | tags=43%, list=34%, signal=66% |
| 0.200692 | 0.511421 | 0.95946 | 0.992394 | 1 | 8367 | tags=55%, list=36%, signal=86% |
| 0.180616 | 0.413967 | 0.984375 | 0.999549 | 1 | 7858 | tags=44%, list=34%, signal=67% |
| 0.17699 | 0.409574 | 0.99361 | 0.998987 | 1 | 9322 | tags=42%, list=40%, signal=69% |
| 0.179512 | 0.403636 | 0.992308 | 0.998444 | 1 | 7858 | tags=43%, list=34%, signal=64% |
