# Supplementary material for: Mutant p53-dependent mitochondrial metabolic alterations in a mesenchymal stem cell-based model of progressive malignancy
Source: Cell Death Differ. 2018 Nov 9;26(9):1566–81. doi: 10.1038/s41418-018-0227-z (PMC6748146; doi:10.1038/s41418-018-0227-z)
Supplement: Supplementary file 4 — Supplementary table 2 [file 41418_2018_227_MOESM4_ESM.pdf]

Annotation Cluster 1	Enrichment Score: 13.195268597156016
Category	Term
GOTERM_BP_DIRECT	GO:0009615~response to virus
GOTERM_BP_DIRECT	GO:0051607~defense response to virus
UP_KEYWORDS	Innate immunity
UP_KEYWORDS	Antiviral defense
UP_KEYWORDS	Immunity
GOTERM_BP_DIRECT	GO:0002376~immune system process
GOTERM_BP_DIRECT	GO:0045071~negative regulation of viral genome replication
GOTERM_BP_DIRECT	GO:0045087~innate immune response

Annotation Cluster 2	Enrichment Score: 6.807379517382686
Category	Term
UP_KEYWORDS	Metal-binding
UP_KEYWORDS	Zinc
UP_KEYWORDS	Zinc-finger
GOTERM_MF_DIRECT	GO:0046872~metal ion binding

Annotation Cluster 3	Enrichment Score: 5.371404391824246
Category	Term
GOTERM_MF_DIRECT	GO:0001730~2'-5'-oligoadenylate synthetase activity
GOTERM_MF_DIRECT	GO:0003725~double-stranded RNA binding
INTERPRO	IPR026774:2'-5'-oligoadenylate synthase
INTERPRO	IPR018952:2'-5'-oligoadenylate synthetase 1, domain 2/C-terminal
INTERPRO	IPR006117:2-5-oligoadenylate synthetase, conserved site
INTERPRO	IPR006116:2-5-oligoadenylate synthetase, N-terminal
INTERPRO	IPR002934:Nucleotidyl transferase domain
GOTERM_BP_DIRECT	GO:0006164~purine nucleotide biosynthetic process
GOTERM_MF_DIRECT	GO:0016779~nucleotidyltransferase activity
UP_KEYWORDS	Nucleotidyltransferase
GOTERM_BP_DIRECT	GO:0006955~immune response

Annotation Cluster 4	Enrichment Score: 5.077378188146548
Category	Term
GOTERM_BP_DIRECT	GO:0035458~cellular response to interferon-beta
INTERPRO	IPR007743:Interferon-inducible GTPase
GOTERM_MF_DIRECT	GO:0003924~GTPase activity
GOTERM_BP_DIRECT	GO:0006952~defense response

Annotation Cluster 5	Enrichment Score: 5.066681884158618
Category	Term
KEGG_PATHWAY	mmu05168:Herpes simplex infection
KEGG_PATHWAY	mmu05162:Measles
KEGG_PATHWAY	mmu05164:Influenza A
KEGG_PATHWAY	mmu05160:Hepatitis C

Annotation Cluster 6	Enrichment Score: 4.211708958414278
Category	Term
UP_KEYWORDS	Mitochondrion
UP_KEYWORDS	Transit peptide

UP_SEQ_FEATURE	transit peptide:Mitochondrion
Annotation Cluster 7	Enrichment Score: 3.4710239293625436
Category	Term
UP_KEYWORDS	Nucleus
UP_KEYWORDS	Transcription
UP_KEYWORDS	Transcription regulation
GOTERM_BP_DIRECT	GO:0006351~transcription, DNA-templated
GOTERM_MF_DIRECT	GO:0003700~transcription factor activity, sequence-specific DNA binding
UP_KEYWORDS	DNA-binding
GOTERM_MF_DIRECT	GO:0003677~DNA binding
GOTERM_BP_DIRECT	GO:0006355~regulation of transcription, DNA-templated
Annotation Cluster 8	Enrichment Score: 3.2779195928201856
Category	Term
INTERPRO	IPR027417:P-loop containing nucleoside triphosphate hydrolase
GOTERM_MF_DIRECT	GO:0005525~GTP binding
UP_KEYWORDS	GTP-binding
GOTERM_MF_DIRECT	GO:0003924~GTPase activity
UP_SEQ_FEATURE	nucleotide phosphate-binding region:GTP
Annotation Cluster 9	Enrichment Score: 2.5156353407135286
Category	Term
UP_KEYWORDS	Aminoacyl-tRNA synthetase
GOTERM_MF_DIRECT	GO:0004812~aminoacyl-tRNA ligase activity
GOTERM_BP_DIRECT	GO:0006418~tRNA aminoacylation for protein translation
KEGG_PATHWAY	mmu00970:Aminoacyl-tRNA biosynthesis
UP_KEYWORDS	Ligase
UP_SEQ_FEATURE	short sequence motif:"HIGH" region
UP_KEYWORDS	Protein biosynthesis
INTERPRO	IPR014729:Rossmann-like alpha/beta/alpha sandwich fold
GOTERM_MF_DIRECT	GO:0016874~ligase activity
INTERPRO	IPR001412:Aminoacyl-tRNA synthetase, class I, conserved site
UP_SEQ_FEATURE	short sequence motif:"KMSKS" region
INTERPRO	IPR006195:Aminoacyl-tRNA synthetase, class II
INTERPRO	IPR002314:Aminoacyl-tRNA synthetase, class II (G/ H/ P/ S), conserved do
GOTERM_BP_DIRECT	GO:0006412~translation
INTERPRO	IPR009080:Aminoacyl-tRNA synthetase, class 1a, anticodon-binding
Annotation Cluster 10	Enrichment Score: 2.5042715396802553
Category	Term
INTERPRO	IPR012317:Poly(ADP-ribose) polymerase, catalytic domain
UP_SEQ_FEATURE	domain:PARP catalytic
INTERPRO	IPR004170:WWE domain
GOTERM_MF_DIRECT	GO:0003950~NAD+ ADP-ribosyltransferase activity
UP_SEQ_FEATURE	domain:WWE
UP_KEYWORDS	NAD
INTERPRO	IPR000571:Zinc finger, CCCH-type
Annotation Cluster 11	Enrichment Score: 2.2128357554985434

Category	Term
UP_KEYWORDS	Endoplasmic reticulum
GOTERM_CC_DIRECT	GO:0005783~endoplasmic reticulum
GOTERM_CC_DIRECT	GO:0005789~endoplasmic reticulum membrane

Annotation Cluster 12	Enrichment Score: 2.0628407504549475
Category	Term
GOTERM_MF_DIRECT	GO:0000975~regulatory region DNA binding
INTERPRO	IPR019817:Interferon regulatory factor, conserved site
INTERPRO	IPR001346:Interferon regulatory factor DNA-binding domain
SMART	SM00348:IRF
UP_SEQ_FEATURE	DNA-binding region:Tryptophan pentad repeat
INTERPRO	IPR017855:SMAD domain-like
INTERPRO	IPR019471:Interferon regulatory factor-3
SMART	SM01243:SM01243
INTERPRO	IPR008984:SMAD/FHA domain
INTERPRO	IPR011991:Winged helix-turn-helix DNA-binding domain

Annotation Cluster 13	Enrichment Score: 2.0382869832033905
Category	Term
INTERPRO	IPR013083:Zinc finger, RING/FYVE/PHD-type
SMART	SM00184:RING
INTERPRO	IPR001841:Zinc finger, RING-type
INTERPRO	IPR017907:Zinc finger, RING-type, conserved site
UP_SEQ_FEATURE	zinc finger region:RING-type

Annotation Cluster 14	Enrichment Score: 1.7737746492511772
Category	Term
GOTERM_BP_DIRECT	GO:0045893~positive regulation of transcription, DNA-templated
GOTERM_BP_DIRECT	GO:0045944~positive regulation of transcription from RNA polymerase II
GOTERM_MF_DIRECT	GO:0003700~transcription factor activity, sequence-specific DNA binding
UP_KEYWORDS	Activator
GOTERM_MF_DIRECT	GO:0043565~sequence-specific DNA binding

Annotation Cluster 15	Enrichment Score: 1.7256241838300834
Category	Term
GOTERM_CC_DIRECT	GO:0020005~symbiont-containing vacuole membrane
INTERPRO	IPR003191:Guanylate-binding protein, C-terminal
GOTERM_BP_DIRECT	GO:0071346~cellular response to interferon-gamma
INTERPRO	IPR015894:Guanylate-binding protein, N-terminal
GOTERM_BP_DIRECT	GO:0042832~defense response to protozoan
GOTERM_BP_DIRECT	GO:0044406~adhesion of symbiont to host
GOTERM_BP_DIRECT	GO:0050830~defense response to Gram-positive bacterium

Annotation Cluster 16	Enrichment Score: 1.7076575626640753
Category	Term
UP_KEYWORDS	Nucleotide-binding
UP_KEYWORDS	Transferase
UP_KEYWORDS	ATP-binding
GOTERM_MF_DIRECT	GO:0016740~transferase activity

GOTERM_MF_DIRECT	GO:0005524~ATP binding
GOTERM_MF_DIRECT	GO:0000166~nucleotide binding
GOTERM_BP_DIRECT	GO:0046777~protein autophosphorylation
UP_SEQ_FEATURE	active site:Proton acceptor
UP_KEYWORDS	Kinase
UP_SEQ_FEATURE	nucleotide phosphate-binding region:ATP
GOTERM_MF_DIRECT	GO:0004672~protein kinase activity
GOTERM_BP_DIRECT	GO:0016310~phosphorylation
GOTERM_BP_DIRECT	GO:0006468~protein phosphorylation
INTERPRO	IPR000719:Protein kinase, catalytic domain
GOTERM_MF_DIRECT	GO:0016301~kinase activity
UP_SEQ_FEATURE	domain:Protein kinase
UP_SEQ_FEATURE	binding site:ATP
INTERPRO	IPR011009:Protein kinase-like domain
INTERPRO	IPR017441:Protein kinase, ATP binding site
UP_KEYWORDS	Serine/threonine-protein kinase
GOTERM_MF_DIRECT	GO:0004674~protein serine/threonine kinase activity
SMART	SM00220:S_TKc
INTERPRO	IPR008271:Serine/threonine-protein kinase, active site

Annotation Cluster 17	Enrichment Score: 1.664983051613404
Category	Term
INTERPRO	IPR003130:Dynamin GTPase effector
INTERPRO	IPR000375:Dynamin central domain
INTERPRO	IPR019762:Dynamin, GTPase region, conserved site
SMART	SM00302:GED
UP_SEQ_FEATURE	domain:GED
INTERPRO	IPR020850:GTPase effector domain, GED
INTERPRO	IPR001401:Dynamin, GTPase domain
SMART	SM00053:DYNc
INTERPRO	IPR022812:Dynamin

Annotation Cluster 18	Enrichment Score: 1.5845680420567625
Category	Term
UP_KEYWORDS	Endosome
GOTERM_CC_DIRECT	GO:0005768~endosome
GOTERM_CC_DIRECT	GO:0005769~early endosome

Annotation Cluster 19	Enrichment Score: 1.4840630619713144
Category	Term
UP_SEQ_FEATURE	domain:SAND
INTERPRO	IPR000770:SAND domain
SMART	SM00258:SAND
INTERPRO	IPR010919:SAND domain-like

Annotation Cluster 20	Enrichment Score: 1.4627125679080828
Category	Term
INTERPRO	IPR016192:APOBEC/CMP deaminase, zinc-binding
INTERPRO	IPR016193:Cytidine deaminase-like
INTERPRO	IPR002125:CMP/dCMP deaminase, zinc-binding

Annotation Cluster 21	Enrichment Score: 1.4090319959943285
Category	Term
UP_SEQ_FEATURE	zinc finger region:C2H2-type 2
UP_SEQ_FEATURE	zinc finger region:C2H2-type 1
UP_SEQ_FEATURE	zinc finger region:C2H2-type 3
INTERPRO	IPR015880:Zinc finger, C2H2-like
SMART	SM00355:ZnF_C2H2
INTERPRO	IPR007087:Zinc finger, C2H2
UP_SEQ_FEATURE	zinc finger region:C2H2-type 5
INTERPRO	IPR013087:Zinc finger C2H2-type/integrase DNA-binding domain
UP_SEQ_FEATURE	zinc finger region:C2H2-type 4
UP_SEQ_FEATURE	zinc finger region:C2H2-type 7
UP_SEQ_FEATURE	zinc finger region:C2H2-type 6
GOTERM_MF_DIRECT	GO:0003676~nucleic acid binding

Annotation Cluster 22	Enrichment Score: 1.3879374767230954
Category	Term
GOTERM_CC_DIRECT	GO:0016023~cytoplasmic, membrane-bounded vesicle
GOTERM_CC_DIRECT	GO:0031410~cytoplasmic vesicle
UP_KEYWORDS	Cytoplasmic vesicle

Annotation Cluster 23	Enrichment Score: 1.3678025249626984
Category	Term
UP_KEYWORDS	Protein transport
UP_KEYWORDS	Transport
GOTERM_BP_DIRECT	GO:0015031~protein transport
GOTERM_BP_DIRECT	GO:0006810~transport

Annotation Cluster 24	Enrichment Score: 1.321341282743224
Category	Term
INTERPRO	IPR021673:C-terminal domain of RIG-I
UP_KEYWORDS	Helicase
KEGG_PATHWAY	mmu04622:RIG-I-like receptor signaling pathway
GOTERM_MF_DIRECT	GO:0004386~helicase activity
INTERPRO	IPR006935:Helicase/UvrB domain
SMART	SM00490:HELICc
INTERPRO	IPR001650:Helicase, C-terminal
INTERPRO	IPR014001:Helicase, superfamily 1/2, ATP-binding domain
SMART	SM00487:DEXDc
UP_SEQ_FEATURE	domain:Helicase ATP-binding
GOTERM_MF_DIRECT	GO:0016817~hydrolase activity, acting on acid anhydrides
GOTERM_MF_DIRECT	GO:0003727~single-stranded RNA binding
GOTERM_MF_DIRECT	GO:0008026~ATP-dependent helicase activity
INTERPRO	IPR011545:DNA/RNA helicase, DEAD/DEAH box type, N-terminal
UP_SEQ_FEATURE	short sequence motif:DEAH box
UP_SEQ_FEATURE	domain:Helicase C-terminal
COG_ONTOLOGY	DNA replication, recombination, and repair
GOTERM_MF_DIRECT	GO:0004004~ATP-dependent RNA helicase activity

Annotation Cluster 25	Enrichment Score: 1.2070452742902662
Category	Term
INTERPRO	IPR011021:Arrestin-like, N-terminal
INTERPRO	IPR011022:Arrestin C-terminal-like domain
SMART	SM01017:SM01017
INTERPRO	IPR014756:Immunoglobulin E-set

Annotation Cluster 26	Enrichment Score: 1.1523106838753976
Category	Term
UP_KEYWORDS	Lipid degradation
KEGG_PATHWAY	mmu00565:Ether lipid metabolism
GOTERM_BP_DIRECT	GO:0016042~lipid catabolic process

Annotation Cluster 27	Enrichment Score: 1.1414124530916265
Category	Term
GOTERM_BP_DIRECT	GO:0050679~positive regulation of epithelial cell proliferation
GOTERM_BP_DIRECT	GO:0042733~embryonic digit morphogenesis
GOTERM_BP_DIRECT	GO:0035115~embryonic forelimb morphogenesis
GOTERM_BP_DIRECT	GO:0042476~odontogenesis
GOTERM_BP_DIRECT	GO:0060021~palate development
GOTERM_BP_DIRECT	GO:0035116~embryonic hindlimb morphogenesis

Annotation Cluster 28	Enrichment Score: 1.131731001752202
Category	Term
INTERPRO	IPR013026:Tetratricopeptide repeat-containing domain
SMART	SM00028:TPR
INTERPRO	IPR019734:Tetratricopeptide repeat
UP_SEQ_FEATURE	repeat:TPR 4
UP_SEQ_FEATURE	repeat:TPR 3
UP_KEYWORDS	TPR repeat
UP_SEQ_FEATURE	repeat:TPR 2
UP_SEQ_FEATURE	repeat:TPR 1
INTERPRO	IPR011990:Tetratricopeptide-like helical
UP_SEQ_FEATURE	repeat:TPR 5
UP_SEQ_FEATURE	repeat:TPR 7
UP_SEQ_FEATURE	repeat:TPR 6
UP_SEQ_FEATURE	repeat:TPR 8

Annotation Cluster 29	Enrichment Score: 1.1204498550026982
Category	Term
GOTERM_BP_DIRECT	GO:0042157~lipoprotein metabolic process
INTERPRO	IPR008405:Apolipoprotein L
GOTERM_BP_DIRECT	GO:0006869~lipid transport

Annotation Cluster 30	Enrichment Score: 1.0935200682405775
Category	Term
UP_KEYWORDS	Amino-acid biosynthesis
GOTERM_BP_DIRECT	GO:0008652~cellular amino acid biosynthetic process
KEGG_PATHWAY	mmu00270:Cysteine and methionine metabolism
UP_KEYWORDS	Pyridoxal phosphate

KEGG_PATHWAY	mmu01230:Biosynthesis of amino acids
INTERPRO	IPR015421:Pyridoxal phosphate-dependent transferase, major region, sub
INTERPRO	IPR015424:Pyridoxal phosphate-dependent transferase
Annotation Cluster 31	Enrichment Score: 1.0303308030013258
Category	Term
UP_KEYWORDS	Guanine-nucleotide releasing factor
GOTERM_MF_DIRECT	GO:0005085~guanyl-nucleotide exchange factor activity
GOTERM_MF_DIRECT	GO:0005089~Rho guanyl-nucleotide exchange factor activity
SMART	SM00325:RhoGEF
INTERPRO	IPR001331:Guanine-nucleotide dissociation stimulator, CDC24, conserved
INTERPRO	IPR000219:Dbl homology (DH) domain
GOTERM_BP_DIRECT	GO:0035023~regulation of Rho protein signal transduction
UP_SEQ_FEATURE	domain:DH
Annotation Cluster 32	Enrichment Score: 0.9933552816738535
Category	Term
UP_SEQ_FEATURE	repeat:ANK 11
UP_SEQ_FEATURE	repeat:ANK 19
UP_SEQ_FEATURE	repeat:ANK 20
UP_SEQ_FEATURE	repeat:ANK 21
UP_SEQ_FEATURE	repeat:ANK 17
UP_SEQ_FEATURE	repeat:ANK 18
UP_SEQ_FEATURE	repeat:ANK 15
UP_SEQ_FEATURE	repeat:ANK 16
UP_SEQ_FEATURE	repeat:ANK 13
UP_SEQ_FEATURE	repeat:ANK 14
UP_SEQ_FEATURE	repeat:ANK 12
UP_SEQ_FEATURE	repeat:ANK 8
UP_SEQ_FEATURE	repeat:ANK 10
UP_SEQ_FEATURE	repeat:ANK 7
UP_SEQ_FEATURE	repeat:ANK 9
UP_SEQ_FEATURE	repeat:ANK 6
UP_SEQ_FEATURE	repeat:ANK 5
UP_SEQ_FEATURE	repeat:ANK 4
UP_KEYWORDS	ANK repeat
SMART	SM00248:ANK
INTERPRO	IPR002110:Ankyrin repeat
INTERPRO	IPR020683:Ankyrin repeat-containing domain
UP_SEQ_FEATURE	repeat:ANK 3
UP_SEQ_FEATURE	repeat:ANK 2
UP_SEQ_FEATURE	repeat:ANK 1
Annotation Cluster 33	Enrichment Score: 0.977722014751383
Category	Term
GOTERM_MF_DIRECT	GO:0004602~glutathione peroxidase activity
KEGG_PATHWAY	mmu00480:Glutathione metabolism
GOTERM_MF_DIRECT	GO:0004364~glutathione transferase activity
KEGG_PATHWAY	mmu00980:Metabolism of xenobiotics by cytochrome P450
KEGG_PATHWAY	mmu00982:Drug metabolism - cytochrome P450

KEGG_PATHWAY	mmu05204:Chemical carcinogenesis
Annotation Cluster 34	Enrichment Score: 0.9710476564013425
Category	Term
GOTERM_BP_DIRECT	GO:0006974~cellular response to DNA damage stimulus
UP_KEYWORDS	DNA repair
UP_KEYWORDS	DNA damage
GOTERM_BP_DIRECT	GO:0006281~DNA repair
Annotation Cluster 35	Enrichment Score: 0.9661798554301865
Category	Term
UP_KEYWORDS	Sterol metabolism
GOTERM_BP_DIRECT	GO:0008202~steroid metabolic process
UP_KEYWORDS	Steroid metabolism
UP_KEYWORDS	Cholesterol metabolism
GOTERM_BP_DIRECT	GO:0008203~cholesterol metabolic process
GOTERM_BP_DIRECT	GO:0042632~cholesterol homeostasis
Annotation Cluster 36	Enrichment Score: 0.961467446403975
Category	Term
UP_KEYWORDS	Vitamin A
UP_KEYWORDS	Retinol-binding
GOTERM_MF_DIRECT	GO:0005501~retinoid binding
GOTERM_MF_DIRECT	GO:0016918~retinal binding
GOTERM_MF_DIRECT	GO:0019841~retinol binding
INTERPRO	IPR000463:Cytosolic fatty-acid binding
INTERPRO	IPR000566:Lipocalin/cytosolic fatty-acid binding protein domain
INTERPRO	IPR012674:Calycin
INTERPRO	IPR011038:Calycin-like
Annotation Cluster 37	Enrichment Score: 0.942074933444372
Category	Term
GOTERM_MF_DIRECT	GO:0005520~insulin-like growth factor binding
GOTERM_BP_DIRECT	GO:0001558~regulation of cell growth
GOTERM_MF_DIRECT	GO:0019838~growth factor binding
UP_KEYWORDS	Growth factor binding
UP_SEQ_FEATURE	domain:IGFBP N-terminal
SMART	SM00121:IB
INTERPRO	IPR000867:Insulin-like growth factor-binding protein, IGFBP
INTERPRO	IPR009030:Insulin-like growth factor binding protein, N-terminal
Annotation Cluster 38	Enrichment Score: 0.9334152272636511
Category	Term
COG_ONTOLOGY	Cell division and chromosome partitioning / Cytoskeleton
INTERPRO	IPR000408:Regulator of chromosome condensation, RCC1
INTERPRO	IPR009091:Regulator of chromosome condensation 1/beta-lactamase-inh
Annotation Cluster 39	Enrichment Score: 0.9302006657401664
Category	Term
UP_KEYWORDS	Mitochondrion inner membrane

UP_SEQ_FEATURE	repeat:Solcar 3
INTERPRO	IPR018108:Mitochondrial substrate/solute carrier
INTERPRO	IPR023395:Mitochondrial carrier domain
UP_SEQ_FEATURE	repeat:Solcar 2
UP_SEQ_FEATURE	repeat:Solcar 1
GOTERM_MF_DIRECT	GO:0003735~structural constituent of ribosome

Annotation Cluster 40	Enrichment Score: 0.9167914720556805
Category	Term
UP_KEYWORDS	Tyrosine-protein kinase
INTERPRO	IPR008266:Tyrosine-protein kinase, active site
GOTERM_BP_DIRECT	GO:0030155~regulation of cell adhesion
GOTERM_MF_DIRECT	GO:0004713~protein tyrosine kinase activity
GOTERM_MF_DIRECT	GO:0004715~non-membrane spanning protein tyrosine kinase activity
INTERPRO	IPR001245:Serine-threonine/tyrosine-protein kinase catalytic domain
GOTERM_MF_DIRECT	GO:0004714~transmembrane receptor protein tyrosine kinase activity
INTERPRO	IPR020635:Tyrosine-protein kinase, catalytic domain
SMART	SM00219:TyrKc
GOTERM_BP_DIRECT	GO:0007169~transmembrane receptor protein tyrosine kinase signaling p
GOTERM_BP_DIRECT	GO:0018108~peptidyl-tyrosine phosphorylation
GOTERM_CC_DIRECT	GO:0031234~extrinsic component of cytoplasmic side of plasma membrai
GOTERM_BP_DIRECT	GO:0038083~peptidyl-tyrosine autophosphorylation

Annotation Cluster 41	Enrichment Score: 0.9101077886957015
Category	Term
KEGG_PATHWAY	mmu00240:Pyrimidine metabolism
UP_KEYWORDS	DNA-directed RNA polymerase
KEGG_PATHWAY	mmu03020:RNA polymerase
GOTERM_MF_DIRECT	GO:0003899~DNA-directed RNA polymerase activity
GOTERM_MF_DIRECT	GO:0001056~RNA polymerase III activity
GOTERM_CC_DIRECT	GO:0005666~DNA-directed RNA polymerase III complex
GOTERM_BP_DIRECT	GO:0006383~transcription from RNA polymerase III promoter
KEGG_PATHWAY	mmu00230:Purine metabolism

Annotation Cluster 42	Enrichment Score: 0.909295919223381
Category	Term
INTERPRO	IPR008979:Galactose-binding domain-like
SMART	SM00231:FA58C
INTERPRO	IPR000421:Coagulation factor 5/8 C-terminal type domain

Annotation Cluster 43	Enrichment Score: 0.8590557989265755
Category	Term
UP_SEQ_FEATURE	domain:WW 2
UP_SEQ_FEATURE	domain:WW 1
SMART	SM00456:WW
INTERPRO	IPR001202:WW domain

Annotation Cluster 44	Enrichment Score: 0.8522780787139118
Category	Term
GOTERM_MF_DIRECT	GO:0001077~transcriptional activator activity, RNA polymerase II core prc

UP_KEYWORDS	Activator
GOTERM_MF_DIRECT	GO:0000978~RNA polymerase II core promoter proximal region sequence

Annotation Cluster 45	Enrichment Score: 0.8131931393363744
Category	Term
GOTERM_MF_DIRECT	GO:0005089~Rho guanyl-nucleotide exchange factor activity
INTERPRO	IPR011993:Pleckstrin homology-like domain
SMART	SM00233:PH
INTERPRO	IPR001849:Pleckstrin homology domain
UP_SEQ_FEATURE	domain:PH

Annotation Cluster 46	Enrichment Score: 0.8102154502628406
Category	Term
UP_SEQ_FEATURE	binding site:FAD
UP_KEYWORDS	Flavoprotein
UP_KEYWORDS	FAD
GOTERM_MF_DIRECT	GO:0050660~flavin adenine dinucleotide binding
UP_SEQ_FEATURE	nucleotide phosphate-binding region:FAD
INTERPRO	IPR023753:Pyridine nucleotide-disulphide oxidoreductase, FAD/NAD(P)-bi

Annotation Cluster 47	Enrichment Score: 0.785894588288057
Category	Term
UP_SEQ_FEATURE	domain:Thyroglobulin type-1
INTERPRO	IPR000716:Thyroglobulin type-1
SMART	SM00211:TY
INTERPRO	IPR009030:Insulin-like growth factor binding protein, N-terminal

Annotation Cluster 48	Enrichment Score: 0.7721122140927985
Category	Term
UP_SEQ_FEATURE	repeat:II
UP_SEQ_FEATURE	repeat:I
UP_SEQ_FEATURE	repeat:IV
UP_SEQ_FEATURE	repeat:III

Annotation Cluster 49	Enrichment Score: 0.7660004143384734
Category	Term
INTERPRO	IPR011011:Zinc finger, FYVE/PHD-type
INTERPRO	IPR017455:Zinc finger, FYVE-related
INTERPRO	IPR000306:Zinc finger, FYVE-type
SMART	SM00064:FYVE

Annotation Cluster 50	Enrichment Score: 0.7531957895348913
Category	Term
UP_SEQ_FEATURE	zinc finger region:B box-type
INTERPRO	IPR017907:Zinc finger, RING-type, conserved site
INTERPRO	IPR027370:RING-type zinc-finger, LisH dimerisation motif
INTERPRO	IPR013320:Concanavalin A-like lectin/glucanase, subgroup
INTERPRO	IPR003879:Butyrophylin-like
UP_SEQ_FEATURE	domain:B30.2/SPRY
SMART	SM00336:BBOX

INTERPRO	IPR000315:Zinc finger, B-box
SMART	SM00449:SPRY
INTERPRO	IPR003877:SPla/RYanodine receptor SPRY
INTERPRO	IPR001870:B30.2/SPRY domain

Annotation Cluster 51	Enrichment Score: 0.7043878201278949
Category	Term
UP_SEQ_FEATURE	zinc finger region:C3H1-type 3
INTERPRO	IPR000571:Zinc finger, CCCH-type
UP_SEQ_FEATURE	zinc finger region:C3H1-type 1
UP_SEQ_FEATURE	zinc finger region:C3H1-type 2

Annotation Cluster 52	Enrichment Score: 0.6786425511652497
Category	Term
GOTERM_BP_DIRECT	GO:0030513~positive regulation of BMP signaling pathway
GOTERM_BP_DIRECT	GO:0060045~positive regulation of cardiac muscle cell proliferation
GOTERM_BP_DIRECT	GO:0003151~outflow tract morphogenesis

Annotation Cluster 53	Enrichment Score: 0.6406602843562897
Category	Term
KEGG_PATHWAY	mmu05213:Endometrial cancer
KEGG_PATHWAY	mmu05222:Small cell lung cancer
KEGG_PATHWAY	mmu04722:Neurotrophin signaling pathway
KEGG_PATHWAY	mmu05221:Acute myeloid leukemia
KEGG_PATHWAY	mmu04917:Prolactin signaling pathway
KEGG_PATHWAY	mmu05230:Central carbon metabolism in cancer
KEGG_PATHWAY	mmu04012:ErbB signaling pathway
KEGG_PATHWAY	mmu04630:Jak-STAT signaling pathway
KEGG_PATHWAY	mmu05223:Non-small cell lung cancer
KEGG_PATHWAY	mmu04919:Thyroid hormone signaling pathway
KEGG_PATHWAY	mmu05205:Proteoglycans in cancer
KEGG_PATHWAY	mmu05216:Thyroid cancer
KEGG_PATHWAY	mmu05161:Hepatitis B
KEGG_PATHWAY	mmu04662:B cell receptor signaling pathway
KEGG_PATHWAY	mmu04068:FoxO signaling pathway
KEGG_PATHWAY	mmu05220:Chronic myeloid leukemia
KEGG_PATHWAY	mmu04210:Apoptosis
KEGG_PATHWAY	mmu04152:AMPK signaling pathway
KEGG_PATHWAY	mmu05219:Bladder cancer
KEGG_PATHWAY	mmu05210:Colorectal cancer
KEGG_PATHWAY	mmu05212:Pancreatic cancer
KEGG_PATHWAY	mmu05214:Glioma
KEGG_PATHWAY	mmu04151:PI3K-Akt signaling pathway
KEGG_PATHWAY	mmu05211:Renal cell carcinoma
KEGG_PATHWAY	mmu04930:Type II diabetes mellitus
KEGG_PATHWAY	mmu05200:Pathways in cancer
KEGG_PATHWAY	mmu05231:Choline metabolism in cancer
KEGG_PATHWAY	mmu04660:T cell receptor signaling pathway
KEGG_PATHWAY	mmu04921:Oxytocin signaling pathway
KEGG_PATHWAY	mmu04370:VEGF signaling pathway

KEGG_PATHWAY	mmu04931:Insulin resistance
KEGG_PATHWAY	mmu05215:Prostate cancer
KEGG_PATHWAY	mmu05166:HTLV-I infection
KEGG_PATHWAY	mmu04973:Carbohydrate digestion and absorption
KEGG_PATHWAY	mmu04664:Fc epsilon RI signaling pathway
KEGG_PATHWAY	mmu05218:Melanoma
KEGG_PATHWAY	mmu04915:Estrogen signaling pathway
KEGG_PATHWAY	mmu04650:Natural killer cell mediated cytotoxicity
KEGG_PATHWAY	mmu04666:Fc gamma R-mediated phagocytosis
GOTERM_BP_DIRECT	GO:0045471~response to ethanol
KEGG_PATHWAY	mmu04150:mTOR signaling pathway
KEGG_PATHWAY	mmu04810:Regulation of actin cytoskeleton
KEGG_PATHWAY	mmu04932:Non-alcoholic fatty liver disease (NAFLD)
KEGG_PATHWAY	mmu04910:Insulin signaling pathway
KEGG_PATHWAY	mmu04725:Cholinergic synapse
KEGG_PATHWAY	mmu04110:Cell cycle
KEGG_PATHWAY	mmu04750:Inflammatory mediator regulation of TRP channels
KEGG_PATHWAY	mmu04022:cGMP-PKG signaling pathway
KEGG_PATHWAY	mmu05206:MicroRNAs in cancer
KEGG_PATHWAY	mmu04261:Adrenergic signaling in cardiomyocytes
KEGG_PATHWAY	mmu04071:Sphingolipid signaling pathway
KEGG_PATHWAY	mmu04611:Platelet activation
KEGG_PATHWAY	mmu04024:cAMP signaling pathway

Annotation Cluster 54	Enrichment Score: 0.6385417752083253
Category	Term
UP_SEQ_FEATURE	zinc finger region:C2H2-type 5
UP_SEQ_FEATURE	zinc finger region:C2H2-type 4
UP_SEQ_FEATURE	zinc finger region:C2H2-type 7
UP_SEQ_FEATURE	domain:KRAB
UP_SEQ_FEATURE	zinc finger region:C2H2-type 11
UP_SEQ_FEATURE	zinc finger region:C2H2-type 6
UP_SEQ_FEATURE	zinc finger region:C2H2-type 10
UP_SEQ_FEATURE	zinc finger region:C2H2-type 8
UP_SEQ_FEATURE	zinc finger region:C2H2-type 12
UP_SEQ_FEATURE	zinc finger region:C2H2-type 9

Annotation Cluster 55	Enrichment Score: 0.6332217310048168
Category	Term
GOTERM_BP_DIRECT	GO:0030488~tRNA methylation
UP_KEYWORDS	Methyltransferase
UP_KEYWORDS	S-adenosyl-L-methionine
GOTERM_BP_DIRECT	GO:0032259~methylation
GOTERM_MF_DIRECT	GO:0008168~methyltransferase activity

Annotation Cluster 56	Enrichment Score: 0.6284255908370221
Category	Term
INTERPRO	IPR000418:Ets domain
UP_SEQ_FEATURE	DNA-binding region:ETS
SMART	SM00413:ETS

Annotation Cluster 57	Enrichment Score: 0.6232862313951621
Category	Term
UP_KEYWORDS	Thiol protease
GOTERM_MF_DIRECT	GO:0008234~cysteine-type peptidase activity
UP_KEYWORDS	Protease
GOTERM_MF_DIRECT	GO:0008233~peptidase activity
GOTERM_BP_DIRECT	GO:0006508~proteolysis

Annotation Cluster 58	Enrichment Score: 0.6148810020775494
Category	Term
UP_KEYWORDS	Protein phosphatase
GOTERM_BP_DIRECT	GO:0006470~protein dephosphorylation
GOTERM_MF_DIRECT	GO:0004721~phosphoprotein phosphatase activity
GOTERM_BP_DIRECT	GO:0016311~dephosphorylation
GOTERM_MF_DIRECT	GO:0016791~phosphatase activity
GOTERM_MF_DIRECT	GO:0004725~protein tyrosine phosphatase activity
INTERPRO	IPR000387:Protein-tyrosine/Dual specificity phosphatase
UP_SEQ_FEATURE	active site:Phosphocysteine intermediate
INTERPRO	IPR003595:Protein-tyrosine phosphatase, catalytic
SMART	SM00404:PTPc_motif
UP_SEQ_FEATURE	domain:Tyrosine-protein phosphatase
SMART	SM00194:PTPc
INTERPRO	IPR000242:Protein-tyrosine phosphatase, receptor/non-receptor type
INTERPRO	IPR016130:Protein-tyrosine phosphatase, active site

Annotation Cluster 59	Enrichment Score: 0.6144837198312858
Category	Term
GOTERM_CC_DIRECT	GO:0005770~late endosome
GOTERM_CC_DIRECT	GO:0005764~lysosome
UP_KEYWORDS	Lysosome

Annotation Cluster 60	Enrichment Score: 0.6135038683522933
Category	Term
UP_KEYWORDS	Thiol protease
GOTERM_MF_DIRECT	GO:0008234~cysteine-type peptidase activity
GOTERM_MF_DIRECT	GO:0036459~thiol-dependent ubiquitinyl hydrolase activity
GOTERM_BP_DIRECT	GO:0071108~protein K48-linked deubiquitination
GOTERM_BP_DIRECT	GO:0016579~protein deubiquitination
GOTERM_MF_DIRECT	GO:0004843~thiol-dependent ubiquitin-specific protease activity
GOTERM_BP_DIRECT	GO:0006511~ubiquitin-dependent protein catabolic process

Annotation Cluster 61	Enrichment Score: 0.5878897039358573
Category	Term
KEGG_PATHWAY	mmu00910:Nitrogen metabolism
UP_KEYWORDS	Lyase
GOTERM_MF_DIRECT	GO:0016829~lyase activity

Annotation Cluster 62	Enrichment Score: 0.5838541340280756
Category	Term

GOTERM_MF_DIRECT	GO:0004519~endonuclease activity
INTERPRO	IPR001279:Beta-lactamase-like
UP_KEYWORDS	Endonuclease
UP_KEYWORDS	Nuclease
GOTERM_MF_DIRECT	GO:0004518~nuclease activity
Annotation Cluster 63	Enrichment Score: 0.5594431285648914
Category	Term
INTERPRO	IPR020846:Major facilitator superfamily domain
INTERPRO	IPR011701:Major facilitator superfamily
GOTERM_BP_DIRECT	GO:0055085~transmembrane transport
Annotation Cluster 64	Enrichment Score: 0.5279269881069204
Category	Term
GOTERM_MF_DIRECT	GO:0004896~cytokine receptor activity
UP_SEQ_FEATURE	short sequence motif:WSXWS motif
INTERPRO	IPR003961:Fibronectin, type III
Annotation Cluster 65	Enrichment Score: 0.5189531100740667
Category	Term
UP_KEYWORDS	Prenylation
UP_SEQ_FEATURE	lipid moiety-binding region:S-geranylgeranyl cysteine
INTERPRO	IPR005225:Small GTP-binding protein domain
UP_SEQ_FEATURE	propeptide:Removed in mature form
GOTERM_BP_DIRECT	GO:0007264~small GTPase mediated signal transduction
UP_SEQ_FEATURE	short sequence motif:Effector region
INTERPRO	IPR001806:Small GTPase superfamily
Annotation Cluster 66	Enrichment Score: 0.5016849133043755
Category	Term
INTERPRO	IPR013087:Zinc finger C2H2-type/integrase DNA-binding domain
UP_SEQ_FEATURE	domain:KRAB
SMART	SM00349:KRAB
INTERPRO	IPR001909:Krueppel-associated box
Annotation Cluster 67	Enrichment Score: 0.48627507285377
Category	Term
UP_KEYWORDS	Oxidoreductase
GOTERM_MF_DIRECT	GO:0016491~oxidoreductase activity
GOTERM_BP_DIRECT	GO:0055114~oxidation-reduction process
Annotation Cluster 68	Enrichment Score: 0.46453256967911033
Category	Term
INTERPRO	IPR013761:Sterile alpha motif/pointed domain
INTERPRO	IPR001660:Sterile alpha motif domain
SMART	SM00454:SAM
UP_SEQ_FEATURE	domain:SAM
Annotation Cluster 69	Enrichment Score: 0.45758300230633847
Category	Term

GOTERM_MF_DIRECT	GO:0030881~beta-2-microglobulin binding
GOTERM_CC_DIRECT	GO:0042612~MHC class I protein complex
GOTERM_MF_DIRECT	GO:0042605~peptide antigen binding
INTERPRO	IPR011161:MHC class I-like antigen recognition
GOTERM_BP_DIRECT	GO:0002474~antigen processing and presentation of peptide antigen via I
INTERPRO	IPR011162:MHC classes I/II-like antigen recognition protein
INTERPRO	IPR001039:MHC class I, alpha chain, alpha1/alpha2
INTERPRO	IPR003006:Immunoglobulin/major histocompatibility complex, conserved
SMART	SM00407:IGc1
INTERPRO	IPR003597:Immunoglobulin C1-set
Annotation Cluster 70	Enrichment Score: 0.4514343275886803
Category	Term
UP_SEQ_FEATURE	domain:SANT
INTERPRO	IPR017884:SANT domain
INTERPRO	IPR001005:SANT/Myb domain
SMART	SM00717:SANT
INTERPRO	IPR009057:Homeodomain-like
Annotation Cluster 71	Enrichment Score: 0.4489217265794778
Category	Term
INTERPRO	IPR000301:Tetraspanin
INTERPRO	IPR008952:Tetraspanin, EC2 domain
INTERPRO	IPR018499:Tetraspanin/Peripherin
PIR_SUPERFAMILY	PIRSF002419:tetraspanin
GOTERM_BP_DIRECT	GO:0007166~cell surface receptor signaling pathway
Annotation Cluster 72	Enrichment Score: 0.4448921835253668
Category	Term
INTERPRO	IPR018980:FERM, C-terminal PH-like domain
SMART	SM01196:SM01196
INTERPRO	IPR018979:FERM, N-terminal
INTERPRO	IPR014352:FERM/acyl-CoA-binding protein, 3-helical bundle
UP_SEQ_FEATURE	domain:FERM
INTERPRO	IPR000299:FERM domain
INTERPRO	IPR019748:FERM central domain
INTERPRO	IPR019749:Band 4.1 domain
SMART	SM00295:B41
Annotation Cluster 73	Enrichment Score: 0.44206365319982555
Category	Term
GOTERM_CC_DIRECT	GO:0005761~mitochondrial ribosome
GOTERM_CC_DIRECT	GO:0030529~intracellular ribonucleoprotein complex
UP_KEYWORDS	Ribonucleoprotein
UP_KEYWORDS	Ribosomal protein
GOTERM_MF_DIRECT	GO:0003735~structural constituent of ribosome
GOTERM_CC_DIRECT	GO:0005840~ribosome
KEGG_PATHWAY	mmu03010:Ribosome
Annotation Cluster 74	Enrichment Score: 0.4362364757988361

Category	Term
SMART	SM00228:PDZ
INTERPRO	IPR001478:PDZ domain
UP_SEQ_FEATURE	domain:PDZ

Annotation Cluster 75	Enrichment Score: 0.41532921637339754
Category	Term
UP_KEYWORDS	Iron-sulfur
GOTERM_MF_DIRECT	GO:0051536~iron-sulfur cluster binding
GOTERM_MF_DIRECT	GO:0051539~4 iron, 4 sulfur cluster binding

Annotation Cluster 76	Enrichment Score: 0.40239885396406344
Category	Term
GOTERM_MF_DIRECT	GO:0042605~peptide antigen binding
GOTERM_CC_DIRECT	GO:0030670~phagocytic vesicle membrane
KEGG_PATHWAY	mmu04612:Antigen processing and presentation
KEGG_PATHWAY	mmu04145:Phagosome

Annotation Cluster 77	Enrichment Score: 0.3996273979904003
Category	Term
INTERPRO	IPR011011:Zinc finger, FYVE/PHD-type
INTERPRO	IPR001965:Zinc finger, PHD-type
SMART	SM00249:PHD

Annotation Cluster 78	Enrichment Score: 0.3938020712528611
Category	Term
INTERPRO	IPR004046:Glutathione S-transferase, C-terminal
INTERPRO	IPR010987:Glutathione S-transferase, C-terminal-like
INTERPRO	IPR012336:Thioredoxin-like fold

Annotation Cluster 79	Enrichment Score: 0.3873086630728798
Category	Term
KEGG_PATHWAY	mmu04668:TNF signaling pathway
KEGG_PATHWAY	mmu04060:Cytokine-cytokine receptor interaction
UP_KEYWORDS	Cytokine
GOTERM_MF_DIRECT	GO:0005125~cytokine activity

Annotation Cluster 80	Enrichment Score: 0.38061934520077645
Category	Term
KEGG_PATHWAY	mmu04152:AMPK signaling pathway
KEGG_PATHWAY	mmu04920:Adipocytokine signaling pathway
KEGG_PATHWAY	mmu04922:Glucagon signaling pathway

Annotation Cluster 81	Enrichment Score: 0.37153751789139633
Category	Term
UP_KEYWORDS	Cilium biogenesis/degradation
UP_KEYWORDS	Cilium
GOTERM_CC_DIRECT	GO:0005929~cilium
GOTERM_BP_DIRECT	GO:0035058~nonmotile primary cilium assembly
GOTERM_CC_DIRECT	GO:0036064~ciliary basal body

GOTERM_BP_DIRECT	GO:0030030~cell projection organization
GOTERM_BP_DIRECT	GO:0060271~cilium morphogenesis
GOTERM_BP_DIRECT	GO:0042384~cilium assembly
GOTERM_CC_DIRECT	GO:0005813~centrosome
GOTERM_CC_DIRECT	GO:0005814~centriole

Annotation Cluster 82	Enrichment Score: 0.3705070915495205
Category	Term
UP_SEQ_FEATURE	repeat:HEAT 5
UP_SEQ_FEATURE	repeat:HEAT 4
UP_SEQ_FEATURE	repeat:HEAT 3
UP_SEQ_FEATURE	repeat:HEAT 2
UP_SEQ_FEATURE	repeat:HEAT 1

Annotation Cluster 83	Enrichment Score: 0.3598759140924123
Category	Term
KEGG_PATHWAY	mmu04146:Peroxisome
UP_KEYWORDS	Peroxisome
GOTERM_CC_DIRECT	GO:0005777~peroxisome

Annotation Cluster 84	Enrichment Score: 0.35442010473239455
Category	Term
UP_KEYWORDS	Fatty acid metabolism
UP_KEYWORDS	Lipid biosynthesis
GOTERM_BP_DIRECT	GO:0006631~fatty acid metabolic process
UP_KEYWORDS	Fatty acid biosynthesis
GOTERM_BP_DIRECT	GO:0006633~fatty acid biosynthetic process

Annotation Cluster 85	Enrichment Score: 0.34553165307304123
Category	Term
INTERPRO	IPR001791:Laminin G domain
SMART	SM00282:LamG
INTERPRO	IPR000742:Epidermal growth factor-like domain

Annotation Cluster 86	Enrichment Score: 0.33383052249262757
Category	Term
UP_KEYWORDS	WD repeat
INTERPRO	IPR015943:WD40/YVTN repeat-like-containing domain
INTERPRO	IPR001680:WD40 repeat
SMART	SM00320:WD40
INTERPRO	IPR017986:WD40-repeat-containing domain
INTERPRO	IPR020472:G-protein beta WD-40 repeat
UP_SEQ_FEATURE	repeat:WD 3
UP_SEQ_FEATURE	repeat:WD 2
UP_SEQ_FEATURE	repeat:WD 1
INTERPRO	IPR019775:WD40 repeat, conserved site
UP_SEQ_FEATURE	repeat:WD 4
UP_SEQ_FEATURE	repeat:WD 5
UP_SEQ_FEATURE	repeat:WD 6
UP_SEQ_FEATURE	repeat:WD 7

Annotation Cluster 87	Enrichment Score: 0.30948291087418495
Category	Term
GOTERM_BP_DIRECT	GO:0051017~actin filament bundle assembly
UP_SEQ_FEATURE	domain:SH3
UP_KEYWORDS	SH3 domain
INTERPRO	IPR001452:Src homology-3 domain
SMART	SM00326:SH3
Annotation Cluster 88	Enrichment Score: 0.289429360398106
Category	Term
KEGG_PATHWAY	mmu04510:Focal adhesion
KEGG_PATHWAY	mmu05146:Amoebiasis
KEGG_PATHWAY	mmu04512:ECM-receptor interaction
GOTERM_CC_DIRECT	GO:0005604~basement membrane
Annotation Cluster 89	Enrichment Score: 0.28825240509910094
Category	Term
GOTERM_MF_DIRECT	GO:0070888~E-box binding
UP_SEQ_FEATURE	DNA-binding region:Basic motif
SMART	SM00353:HLH
INTERPRO	IPR011598:Myc-type, basic helix-loop-helix (bHLH) domain
UP_SEQ_FEATURE	domain:Helix-loop-helix motif
GOTERM_MF_DIRECT	GO:0046983~protein dimerization activity
Annotation Cluster 90	Enrichment Score: 0.246599578011054
Category	Term
UP_SEQ_FEATURE	domain:SH2
INTERPRO	IPR000980:SH2 domain
UP_KEYWORDS	SH2 domain
SMART	SM00252:SH2
Annotation Cluster 91	Enrichment Score: 0.2274488224850573
Category	Term
GOTERM_MF_DIRECT	GO:0061630~ubiquitin protein ligase activity
UP_KEYWORDS	Ubl conjugation pathway
GOTERM_BP_DIRECT	GO:0016567~protein ubiquitination
GOTERM_MF_DIRECT	GO:0004842~ubiquitin-protein transferase activity
Annotation Cluster 92	Enrichment Score: 0.22614544450373625
Category	Term
INTERPRO	IPR008160:Collagen triple helix repeat
UP_KEYWORDS	Collagen
UP_KEYWORDS	Extracellular matrix
GOTERM_CC_DIRECT	GO:0005581~collagen trimer
Annotation Cluster 93	Enrichment Score: 0.2163118368490164
Category	Term
GOTERM_CC_DIRECT	GO:0005913~cell-cell adherens junction
GOTERM_MF_DIRECT	GO:0098641~cadherin binding involved in cell-cell adhesion

GOTERM_BP_DIRECT	GO:0098609~cell-cell adhesion
Annotation Cluster 94	Enrichment Score: 0.21534295115873656
Category	Term
UP_SEQ_FEATURE	domain:C2
INTERPRO	IPR000008:C2 calcium-dependent membrane targeting
SMART	SM00239:C2
Annotation Cluster 95	Enrichment Score: 0.2091659143714945
Category	Term
INTERPRO	IPR017871:ABC transporter, conserved site
KEGG_PATHWAY	mmu02010:ABC transporters
GOTERM_MF_DIRECT	GO:0042626~ATPase activity, coupled to transmembrane movement of su
INTERPRO	IPR003439:ABC transporter-like
INTERPRO	IPR003593:AAA+ ATPase domain
SMART	SM00382:AAA
GOTERM_MF_DIRECT	GO:0016887~ATPase activity
Annotation Cluster 96	Enrichment Score: 0.1942465727085632
Category	Term
INTERPRO	IPR008936:Rho GTPase activation protein
SMART	SM00324:RhoGAP
UP_SEQ_FEATURE	domain:Rho-GAP
INTERPRO	IPR000198:Rho GTPase-activating protein domain
Annotation Cluster 97	Enrichment Score: 0.18936787762871923
Category	Term
GOTERM_CC_DIRECT	GO:0016020~membrane
UP_SEQ_FEATURE	transmembrane region
UP_KEYWORDS	Membrane
UP_SEQ_FEATURE	topological domain:Extracellular
UP_SEQ_FEATURE	topological domain:Cytoplasmic
UP_SEQ_FEATURE	glycosylation site:N-linked (GlcNAc...)
UP_KEYWORDS	Transmembrane helix
UP_KEYWORDS	Transmembrane
GOTERM_CC_DIRECT	GO:0016021~integral component of membrane
Annotation Cluster 98	Enrichment Score: 0.15511653346524382
Category	Term
GOTERM_CC_DIRECT	GO:0014069~postsynaptic density
UP_KEYWORDS	Synapse
GOTERM_CC_DIRECT	GO:0045202~synapse
UP_KEYWORDS	Cell junction
GOTERM_CC_DIRECT	GO:0030054~cell junction
Annotation Cluster 99	Enrichment Score: 0.15156651398096185
Category	Term
KEGG_PATHWAY	mmu05410:Hypertrophic cardiomyopathy (HCM)
KEGG_PATHWAY	mmu05412:Arrhythmogenic right ventricular cardiomyopathy (ARVC)
KEGG_PATHWAY	mmu05414:Dilated cardiomyopathy

Annotation Cluster 100	Enrichment Score: 0.14655563940347935
Category	Term
UP_KEYWORDS	Biological rhythms
GOTERM_BP_DIRECT	GO:0048511~rhythmic process
GOTERM_BP_DIRECT	GO:0007623~circadian rhythm
Annotation Cluster 101	Enrichment Score: 0.13499607529702456
Category	Term
UP_SEQ_FEATURE	domain:Ig-like C2-type
INTERPRO	IPR013098:Immunoglobulin I-set
UP_KEYWORDS	Immunoglobulin domain
INTERPRO	IPR003598:Immunoglobulin subtype 2
SMART	SM00408:IGc2
INTERPRO	IPR003599:Immunoglobulin subtype
SMART	SM00409:IG
INTERPRO	IPR013783:Immunoglobulin-like fold
INTERPRO	IPR007110:Immunoglobulin-like domain
Annotation Cluster 102	Enrichment Score: 0.09372770012503656
Category	Term
UP_SEQ_FEATURE	domain:BTB
SMART	SM00225:BTB
INTERPRO	IPR000210:BTB/POZ-like
INTERPRO	IPR011333:BTB/POZ fold
Annotation Cluster 103	Enrichment Score: 0.0922369706912232
Category	Term
INTERPRO	IPR000742:Epidermal growth factor-like domain
UP_SEQ_FEATURE	domain:EGF-like
UP_KEYWORDS	EGF-like domain
UP_SEQ_FEATURE	domain:EGF-like 2
INTERPRO	IPR013032:EGF-like, conserved site
UP_SEQ_FEATURE	domain:EGF-like 1
SMART	SM00181:EGF
Annotation Cluster 104	Enrichment Score: 0.08555796835887602
Category	Term
INTERPRO	IPR013164:Cadherin, N-terminal
INTERPRO	IPR002126:Cadherin
SMART	SM00112:CA
INTERPRO	IPR015919:Cadherin-like
INTERPRO	IPR020894:Cadherin conserved site
GOTERM_BP_DIRECT	GO:0007156~homophilic cell adhesion via plasma membrane adhesion m[illegible]
Annotation Cluster 105	Enrichment Score: 0.08421116676238453
Category	Term
UP_SEQ_FEATURE	repeat:LRR 10
UP_SEQ_FEATURE	repeat:LRR 11
UP_SEQ_FEATURE	repeat:LRR 9

UP_SEQ_FEATURE	repeat:LRR 13
UP_SEQ_FEATURE	repeat:LRR 8
UP_SEQ_FEATURE	repeat:LRR 12
UP_SEQ_FEATURE	repeat:LRR 7
UP_SEQ_FEATURE	repeat:LRR 6
INTERPRO	IPR003591:Leucine-rich repeat, typical subtype
UP_SEQ_FEATURE	repeat:LRR 5
UP_KEYWORDS	Leucine-rich repeat
SMART	SM00369:LRR_TYP
UP_SEQ_FEATURE	repeat:LRR 4
INTERPRO	IPR001611:Leucine-rich repeat
UP_SEQ_FEATURE	repeat:LRR 3
UP_SEQ_FEATURE	repeat:LRR 2
UP_SEQ_FEATURE	repeat:LRR 1

Annotation Cluster 106	Enrichment Score: 0.07513508155008054
Category	Term
GOTERM_BP_DIRECT	GO:0034765~regulation of ion transmembrane transport
UP_KEYWORDS	Voltage-gated channel
GOTERM_MF_DIRECT	GO:0005244~voltage-gated ion channel activity
UP_KEYWORDS	Ion channel

Annotation Cluster 107	Enrichment Score: 0.05142447157418296
Category	Term
GOTERM_MF_DIRECT	GO:0008236~serine-type peptidase activity
GOTERM_MF_DIRECT	GO:0004252~serine-type endopeptidase activity
UP_KEYWORDS	Serine protease

Annotation Cluster 108	Enrichment Score: 0.03904906240784728
Category	Term
UP_KEYWORDS	Ion transport
GOTERM_BP_DIRECT	GO:0006811~ion transport
UP_KEYWORDS	Ion channel

Annotation Cluster 109	Enrichment Score: 0.03650113345805451
Category	Term
UP_SEQ_FEATURE	repeat:4
UP_SEQ_FEATURE	repeat:3
UP_SEQ_FEATURE	repeat:5
UP_SEQ_FEATURE	repeat:1
UP_SEQ_FEATURE	repeat:2

Annotation Cluster 110	Enrichment Score: 0.035104293344696426
Category	Term
INTERPRO	IPR011992:EF-hand-like domain
SMART	SM00054:EFh
UP_SEQ_FEATURE	calcium-binding region:2
UP_SEQ_FEATURE	calcium-binding region:1
UP_SEQ_FEATURE	domain:EF-hand 2
INTERPRO	IPR002048:EF-hand domain

UP_SEQ_FEATURE	domain:EF-hand 1
INTERPRO	IPR018247:EF-Hand 1, calcium-binding site

Annotation Cluster 111	Enrichment Score: 0.032814098359388214
Category	Term
UP_KEYWORDS	mRNA processing
KEGG_PATHWAY	mmu03015:mRNA surveillance pathway
GOTERM_BP_DIRECT	GO:0006397~mRNA processing
UP_KEYWORDS	mRNA splicing
GOTERM_BP_DIRECT	GO:0008380~RNA splicing

Annotation Cluster 112	Enrichment Score: 0.016729313217965877
Category	Term
UP_KEYWORDS	Cell cycle
UP_KEYWORDS	Cell division
GOTERM_BP_DIRECT	GO:0007049~cell cycle
GOTERM_BP_DIRECT	GO:0007067~mitotic nuclear division
GOTERM_BP_DIRECT	GO:0051301~cell division
UP_KEYWORDS	Mitosis

Annotation Cluster 113	Enrichment Score: 0.015185607101453584
Category	Term
GOTERM_CC_DIRECT	GO:0005615~extracellular space
UP_KEYWORDS	Secreted
GOTERM_CC_DIRECT	GO:0005576~extracellular region
UP_SEQ_FEATURE	signal peptide

Annotation Cluster 114	Enrichment Score: 0.004370800534753207
Category	Term
INTERPRO	IPR009057:Homeodomain-like
INTERPRO	IPR017970:Homeobox, conserved site
UP_SEQ_FEATURE	DNA-binding region:Homeobox
UP_KEYWORDS	Homeobox
SMART	SM00389:HOX
INTERPRO	IPR001356:Homeodomain

Annotation Cluster 115	Enrichment Score: 0.003898878823604014
Category	Term
UP_KEYWORDS	Glycoprotein
UP_KEYWORDS	Disulfide bond
UP_SEQ_FEATURE	topological domain:Extracellular
UP_SEQ_FEATURE	topological domain:Cytoplasmic
UP_SEQ_FEATURE	signal peptide
UP_SEQ_FEATURE	disulfide bond
UP_SEQ_FEATURE	glycosylation site:N-linked (GlcNAc...)
UP_KEYWORDS	Signal

Count	%	PValue	Genes	List Total	Pop Hits	Pop Total	Fold Enrich	Bonferroni
29	3.877005	6.57E-20	IFIH1, IFITM	661	84	18082	9.444168	1.88E-16
35	4.679144	1.75E-16	IFIH1, APOI	661	167	18082	5.733193	6.35E-13
39	5.213904	4.52E-16	IFIH1, ZC3H	728	241	22680	5.041494	1.50E-13
25	3.342246	7.69E-15	IFIH1, POLF	728	100	22680	7.788462	2.59E-12
47	6.283422	7.42E-14	IFIH1, ZC3H	728	401	22680	3.651448	2.51E-11
44	5.882353	5.98E-11	IFIH1, ZC3H	661	383	18082	3.142671	1.71E-07
13	1.737968	5.85E-10	BST2, ZC3H	661	32	18082	11.11318	1.67E-06
42	5.614973	2.63E-09	IFIH1, ZC3H	661	400	18082	2.87233	7.53E-06

Count	%	PValue	Genes	List Total	Pop Hits	Pop Total	Fold Enrich	Bonferroni
169	22.59358	2.45E-09	S100A6, AF	728	3395	22680	1.55081	8.27E-07
117	15.64171	3.66E-09	S100A5, AF	728	2099	22680	1.736541	1.24E-06
84	11.22995	4.18E-06	ZFP13, ZC3	728	1565	22680	1.672155	0.001413
167	22.3262	1.57E-05	S100A6, AF	642	3355	17446	1.352648	0.013605

Count	%	PValue	Genes	List Total	Pop Hits	Pop Total	Fold Enrich	Bonferroni
8	1.069519	2.54E-08	OASL2, OAS	642	11	17446	19.76324	2.21E-05
16	2.139037	2.82E-08	IFIH1, ADAI	642	70	17446	6.211304	2.46E-05
8	1.069519	3.51E-08	OASL2, OAS	702	12	20594	19.55745	4.63E-05
8	1.069519	3.51E-08	OASL2, OAS	702	12	20594	19.55745	4.63E-05
7	0.935829	4.02E-08	OASL2, OAS	702	8	20594	25.66916	5.31E-05
8	1.069519	1.43E-07	OASL2, OAS	702	14	20594	16.76353	1.89E-04
6	0.802139	6.98E-05	OASL2, OAS	702	14	20594	12.57265	0.088118
6	0.802139	6.27E-04	ADSS, OASI	661	20	18082	8.206657	0.833433
11	1.470588	0.001094	POLI, REV1	642	85	17446	3.516694	0.614501
9	1.203209	0.002967	POLI, REV1	728	76	22680	3.689271	0.633737
19	2.540107	0.011386	OAS3, H2-D	661	272	18082	1.910864	1

Count	%	PValue	Genes	List Total	Pop Hits	Pop Total	Fold Enrich	Bonferroni
15	2.005348	9.20E-11	IRGM1, IRG	661	41	18082	10.00812	2.63E-07
8	1.069519	1.18E-06	IRGM1, IRG	702	18	20594	13.0383	0.001555
19	2.540107	7.29E-04	GBP7, RAB:	642	209	17446	2.470405	0.469985
9	1.203209	0.062091	IRGM1, IRG	661	116	18082	2.122411	1

Count	%	PValue	Genes	List Total	Pop Hits	Pop Total	Fold Enrich	Bonferroni
24	3.208556	2.61E-06	TRP53, TRA	289	208	7720	3.082246	6.49E-04
19	2.540107	2.63E-06	TRP53, IFIH	289	136	7720	3.731936	6.54E-04
21	2.807487	5.12E-06	IFIH1, AGF(	289	171	7720	3.28052	0.001273
16	2.139037	1.55E-04	TRP53, PIK:	289	136	7720	3.142683	0.03776

Count	%	PValue	Genes	List Total	Pop Hits	Pop Total	Fold Enrich	Bonferroni
68	9.090909	8.63E-08	NDUFAF6,	728	1058	22680	2.002327	2.92E-05
33	4.411765	2.58E-04	ME3, NDUI	728	510	22680	2.015837	0.083587

29	3.877005	0.010398	ME3, NDUI	638	496	18012	1.65066	1

Count	%	PValue	Genes	List Total	Pop Hits	Pop Total	Fold Enrich	Bonferroni
215	28.74332	4.37E-10	S100A6, FA	728	4534	22680	1.4773	1.48E-07
92	12.29947	3.27E-05	AKNA, ELF1	728	1859	22680	1.541772	0.011004
86	11.49733	1.92E-04	AKNA, ELF1	728	1799	22680	1.489289	0.062964
92	12.29947	0.003254	AKNA, ELF1	661	1885	18082	1.335124	0.99991
49	6.550802	0.004395	DEAF1, ZFP	642	883	17446	1.507982	0.978435
71	9.491979	0.005283	AKNA, ELF1	728	1604	22680	1.379004	0.833089
85	11.36364	0.023084	AKNA, ELF1	642	1847	17446	1.250584	1
100	13.36898	0.035546	AKNA, CDK	661	2279	18082	1.20033	1

Count	%	PValue	Genes	List Total	Pop Hits	Pop Total	Fold Enrich	Bonferroni
57	7.620321	1.36E-05	SEPT3, SLFI	702	909	20594	1.839563	0.017792
29	3.877005	4.50E-04	ADSS, RAB3	642	383	17446	2.057596	0.324465
24	3.208556	4.78E-04	RAP2B, GBI	728	332	22680	2.252085	0.149278
19	2.540107	7.29E-04	GBP7, RAB3	642	209	17446	2.470405	0.469985
20	2.673797	0.019127	RAP2B, ADS	638	319	18012	1.77003	1

Count	%	PValue	Genes	List Total	Pop Hits	Pop Total	Fold Enrich	Bonferroni
9	1.203209	2.24E-05	WARS, DAF	728	38	22680	7.378543	0.00753
9	1.203209	7.13E-05	WARS, DAF	642	39	17446	6.271028	0.060208
8	1.069519	2.77E-04	WARS, DAF	661	36	18082	6.079005	0.547179
10	1.336898	7.05E-04	WARS, DAF	289	66	7720	4.047394	0.161016
23	3.074866	0.00105	DTX4, ADSS	728	330	22680	2.171329	0.298772
5	0.668449	0.002553	WARS, RAR	638	17	18012	8.303522	0.984056
13	1.737968	0.002803	WARS, EIF4	728	147	22680	2.755102	0.612727
7	0.935829	0.00357	WARS, TRN	702	44	20594	4.66712	0.991128
24	3.208556	0.00809	DTX4, ADSS	642	362	17446	1.801621	0.999154
4	0.534759	0.013159	WARS, RAR	702	15	20594	7.822982	1
4	0.534759	0.014579	WARS, EPR	638	15	18012	7.528527	1
4	0.534759	0.018701	DARS, SARS	702	17	20594	6.902631	1
3	0.40107	0.028268	SARS, EPRS	702	8	20594	11.00107	1
23	3.074866	0.038206	DARS, SARS	661	401	18082	1.56902	1
3	0.40107	0.070421	RARS, MAR	702	13	20594	6.769888	1

Count	%	PValue	Genes	List Total	Pop Hits	Pop Total	Fold Enrich	Bonferroni
6	0.802139	1.44E-04	PARP9, ZC3	702	16	20594	11.00107	0.173185
5	0.668449	4.19E-04	PARP9, ZC3	638	11	18012	12.83272	0.492824
5	0.668449	5.30E-04	DTX4, ZC3H	702	12	20594	12.22341	0.503282
6	0.802139	8.22E-04	PARP9, ZC3	642	21	17446	7.76413	0.511369
3	0.40107	0.023301	ZC3HAV1, I	638	7	18012	12.09942	1
12	1.604278	0.034217	XDH, CYB5I	728	183	22680	2.042875	0.999992
5	0.668449	0.140954	ZC3H3, ZC3	702	59	20594	2.486117	1

Count	%	PValue	Genes	List Total	Pop Hits	Pop Total	Fold Enrich	Bonferroni
51	6.818182	0.001252	JPH4, HPS6	728	997	22680	1.593627	0.345243
64	8.55615	0.009397	JPH4, SLC2	695	1323	19662	1.368557	0.988393
37	4.946524	0.019537	JPH4, ERMI	695	710	19662	1.474301	0.99991

Count	%	PValue	Genes	List Total	Pop Hits	Pop Total	Fold Enrich	Bonferroni
6	0.802139	2.05E-04	IRF9, IRF5,	642	16	17446	10.19042	0.163889
4	0.534759	0.002829	IRF9, IRF5,	702	9	20594	13.0383	0.976296
4	0.534759	0.002829	IRF9, IRF5,	702	9	20594	13.0383	0.976296
4	0.534759	0.002914	IRF9, IRF5,	360	9	10425	12.87037	0.56974
4	0.534759	0.003152	IRF9, IRF5,	638	9	18012	12.54754	0.993974
4	0.534759	0.013159	IRF9, SMAD	702	15	20594	7.822982	1
3	0.40107	0.021685	IRF9, IRF5,	702	7	20594	12.57265	1
3	0.40107	0.022142	IRF9, IRF5,	360	7	10425	12.41071	0.998452
5	0.668449	0.100302	IRF9, SMAD	702	52	20594	2.820787	1
11	1.470588	0.245804	IRF9, ELF1,	702	227	20594	1.421577	1

Count	%	PValue	Genes	List Total	Pop Hits	Pop Total	Fold Enrich	Bonferroni
28	3.743316	0.002877	CBLL1, RNF	702	445	20594	1.845872	0.977749
18	2.406417	0.003012	DTX4, DTX3	360	234	10425	2.227564	0.581747
19	2.540107	0.009404	DTX4, DTX3	702	286	20594	1.948907	0.999996
12	1.604278	0.023917	TRIM65, TF	702	163	20594	2.159719	1
13	1.737968	0.033028	DTX3L, TRII	638	186	18012	1.973203	1

Count	%	PValue	Genes	List Total	Pop Hits	Pop Total	Fold Enrich	Bonferroni
36	4.812834	0.002316	DEAF1, ELF	661	576	18082	1.70972	0.998678
54	7.219251	0.003713	AKNA, NAN	661	995	18082	1.484621	0.999976
49	6.550802	0.004395	DEAF1, ZFP	642	883	17446	1.507982	0.978435
26	3.475936	0.150326	AKNA, TRP!	728	624	22680	1.298077	1
28	3.743316	0.238046	ELF1, ZFP1	642	633	17446	1.20203	1

Count	%	PValue	Genes	List Total	Pop Hits	Pop Total	Fold Enrich	Bonferroni
4	0.534759	0.002147	GBP7, IIGP	695	8	19662	14.14532	0.637351
4	0.534759	0.010797	GBP7, GBP	702	14	20594	8.381766	0.999999
8	1.069519	0.011807	GBP7, GBP	661	68	18082	3.218297	1
4	0.534759	0.013159	GBP7, GBP	702	15	20594	7.822982	1
5	0.668449	0.022694	GBP7, IRGN	661	30	18082	4.559254	1
3	0.40107	0.040392	GBP7, GBP	661	9	18082	9.118507	1
6	0.802139	0.252358	GBP7, P2R)	661	93	18082	1.764872	1

Count	%	PValue	Genes	List Total	Pop Hits	Pop Total	Fold Enrich	Bonferroni
94	12.56684	1.03E-06	SLC27A1, S	728	1754	22680	1.66959	3.49E-04
85	11.36364	1.83E-05	NAMPT, SE	728	1654	22680	1.601014	0.006153
69	9.224599	1.99E-04	SEPHS2, PA	728	1363	22680	1.577121	0.065028
81	10.82888	2.43E-04	NAMPT, SE	642	1472	17446	1.495333	0.191094

81	10.82888	5.00E-04	SEPHS2, PA	642	1507	17446	1.460604	0.353357
91	12.16578	0.01128	SLC27A1, S	642	1936	17446	1.277312	0.999949
14	1.871658	0.016972	HTATIP2, F	661	183	18082	2.092772	1
37	4.946524	0.019522	XDH, ME3,	638	710	18012	1.471244	1
34	4.545455	0.020712	SEPHS2, PF	728	707	22680	1.498205	0.999153
46	6.149733	0.033853	SEPHS2, TD	638	963	18012	1.348568	1
28	3.743316	0.056138	FASTKD2, F	642	531	17446	1.432928	1
30	4.010695	0.092757	SEPHS2, PA	661	612	18082	1.340957	1
28	3.743316	0.110617	PASK, MAP	661	576	18082	1.329782	1
24	3.208556	0.112533	FLT1, FLT3,	702	515	20594	1.367123	1
32	4.278075	0.116792	SEPHS2, PA	642	674	17446	1.290182	1
24	3.208556	0.124571	FLT1, FLT3,	638	502	18012	1.349736	1
27	3.609626	0.132074	PASK, MAP	638	583	18012	1.307484	1
25	3.342246	0.137763	PASK, MAP	702	556	20594	1.319073	1
17	2.272727	0.270025	FLT1, FLT3,	702	394	20594	1.265774	1
15	2.005348	0.424379	STK24, PNC	728	405	22680	1.153846	1
17	2.272727	0.509161	HTATIP2, S	642	428	17446	1.079359	1
14	1.871658	0.550561	STK24, PNC	360	380	10425	1.066886	1
10	1.336898	0.803244	MAP3K15,	702	333	20594	0.880966	1

Count	%	PValue	Genes	List Total	Pop Hits	Pop Total	Fold Enrich	Bonferroni
3	0.40107	0.015844	MX1, MX2,	702	6	20594	14.66809	1
3	0.40107	0.015844	MX1, MX2,	702	6	20594	14.66809	1
3	0.40107	0.015844	MX1, MX2,	702	6	20594	14.66809	1
3	0.40107	0.016181	MX1, MX2,	360	6	10425	14.47917	0.991036
3	0.40107	0.017039	MX1, MX2,	638	6	18012	14.11599	1
3	0.40107	0.021685	MX1, MX2,	702	7	20594	12.57265	1
3	0.40107	0.021685	MX1, MX2,	702	7	20594	12.57265	1
3	0.40107	0.022142	MX1, MX2,	360	7	10425	12.41071	0.998452
3	0.40107	0.090696	MX1, MX2,	702	15	20594	5.867236	1

Count	%	PValue	Genes	List Total	Pop Hits	Pop Total	Fold Enrich	Bonferroni
25	3.342246	0.009083	RAP2B, DEI	728	443	22680	1.758118	0.954226
30	4.010695	0.018554	SNX19, CCI	695	544	19662	1.560146	0.999855
13	1.737968	0.104627	SH3GL3, AN	695	226	19662	1.627338	1

Count	%	PValue	Genes	List Total	Pop Hits	Pop Total	Fold Enrich	Bonferroni
3	0.40107	0.023301	DEAF1, SP1	638	7	18012	12.09942	1
3	0.40107	0.028268	DEAF1, SP1	702	8	20594	11.00107	1
3	0.40107	0.028858	DEAF1, SP1	360	8	10425	10.85938	0.999789
3	0.40107	0.060925	DEAF1, SP1	702	12	20594	7.334046	1

Count	%	PValue	Genes	List Total	Pop Hits	Pop Total	Fold Enrich	Bonferroni
3	0.40107	0.021685	APOBEC1, A	702	7	20594	12.57265	1
3	0.40107	0.043436	APOBEC1, A	702	10	20594	8.800855	1
3	0.40107	0.043436	APOBEC1, A	702	10	20594	8.800855	1

Count	%	PValue	Genes	List Total	Pop Hits	Pop Total	Fold Enrich	Bonferroni
21	2.807487	0.001416	ZFP710, ZF	638	268	18012	2.212207	0.899157
20	2.673797	0.002636	ZFP710, ZF	638	263	18012	2.146918	0.986073
18	2.406417	0.011753	ZFP710, ZF	638	261	18012	1.947033	1
34	4.545455	0.032992	ZFP358, ZF	702	693	20594	1.439293	1
34	4.545455	0.03607	ZFP358, ZF	360	693	10425	1.420755	0.999975
35	4.679144	0.041343	ZFP358, ZF	702	731	20594	1.404605	1
13	1.737968	0.043339	ZFP710, ZF	638	194	18012	1.891833	1
31	4.144385	0.058659	ZFP358, ZF	702	652	20594	1.394818	1
13	1.737968	0.080804	ZFP710, ZF	638	215	18012	1.70705	1
8	1.069519	0.172872	ZFP710, ZF	638	129	18012	1.75082	1
8	1.069519	0.292308	ZFP710, ZF	638	152	18012	1.485893	1
46	6.149733	0.551106	ZFP358, ZF	642	1237	17446	1.010529	1

Count	%	PValue	Genes	List Total	Pop Hits	Pop Total	Fold Enrich	Bonferroni
12	1.604278	0.018287	PLA2G4A, A	695	151	19662	2.248263	0.999835
32	4.278075	0.053122	SNX19, SLC	695	646	19662	1.401394	1
23	3.074866	0.070594	SNX19, FM	728	489	22680	1.465314	1

Count	%	PValue	Genes	List Total	Pop Hits	Pop Total	Fold Enrich	Bonferroni
30	4.010695	0.011856	SNX19, AFT	728	577	22680	1.619784	0.982249
76	10.16043	0.035296	SLC27A1, S	728	1901	22680	1.245498	0.999995
31	4.144385	0.04486	SNX19, AFT	661	592	18082	1.432468	1
75	10.02674	0.179994	SLC27A1, C	661	1822	18082	1.126051	1

Count	%	PValue	Genes	List Total	Pop Hits	Pop Total	Fold Enrich	Bonferroni
3	0.40107	0.003393	DDX58, IFIH	702	3	20594	29.33618	0.988771
11	1.470588	0.009148	DDX58, REC	728	130	22680	2.636095	0.955224
8	1.069519	0.012956	DDX58, IFIH	289	68	7720	3.142683	0.96111
11	1.470588	0.020642	DDX58, REC	642	129	17446	2.317202	1
3	0.40107	0.028268	DDX58, IFIH	702	8	20594	11.00107	1
9	1.203209	0.032672	DDX58, REC	360	108	10425	2.413194	0.999932
9	1.203209	0.03268	DDX58, REC	702	109	20594	2.422254	1
9	1.203209	0.035845	DDX58, REC	702	111	20594	2.378609	1
9	1.203209	0.035876	DDX58, REC	360	110	10425	2.369318	0.999974
8	1.069519	0.058746	DDX58, REC	638	98	18012	2.304651	1
3	0.40107	0.059514	DDX58, IFIH	642	11	17446	7.411215	1
5	0.668449	0.067351	DDX58, IFIH	642	42	17446	3.235054	1
4	0.534759	0.082025	RECQL4, DH	642	28	17446	3.882065	1
6	0.802139	0.085723	DDX58, REC	702	69	20594	2.550972	1
4	0.534759	0.11769	RECQL4, TD	638	34	18012	3.321409	1
7	0.935829	0.120176	DDX58, REC	638	95	18012	2.080251	1
5	0.668449	0.321078	DDX58, IFIH	88	70	2126	1.725649	0.999938
3	0.40107	0.678687	DDX18, TDI	642	63	17446	1.294022	1

Count	%	PValue	Genes	List Total	Pop Hits	Pop Total	Fold Enrich	Bonferroni
3	0.40107	0.043436	TXNIP, ARR	702	10	20594	8.800855	1
3	0.40107	0.043436	TXNIP, ARR	702	10	20594	8.800855	1
3	0.40107	0.044324	TXNIP, ARR	360	10	10425	8.6875	0.999998
7	0.935829	0.177612	TXNIP, ARR	702	111	20594	1.85003	1

Count	%	PValue	Genes	List Total	Pop Hits	Pop Total	Fold Enrich	Bonferroni
8	1.069519	0.041822	PLA2G4A, I	728	100	22680	2.492308	0.999999
5	0.668449	0.080347	PLA2G4A, (	289	44	7720	3.035546	1
8	1.069519	0.103919	PLA2G4A, I	661	109	18082	2.007745	1

Count	%	PValue	Genes	List Total	Pop Hits	Pop Total	Fold Enrich	Bonferroni
8	1.069519	0.020793	GLUL, OSR2	661	76	18082	2.879529	1
7	0.935829	0.03304	MKS1, OSR	661	66	18082	2.901343	1
5	0.668449	0.041152	ZFP358, OS	661	36	18082	3.799378	1
4	0.534759	0.074046	OSR2, OSR1	661	27	18082	4.05267	1
6	0.802139	0.198921	OSR2, OSR1	661	85	18082	1.930978	1
3	0.40107	0.34041	OSR2, OSR1	661	33	18082	2.486866	1

Count	%	PValue	Genes	List Total	Pop Hits	Pop Total	Fold Enrich	Bonferroni
10	1.336898	0.021493	IFIT3, TRAP	702	120	20594	2.444682	1
10	1.336898	0.021538	IFIT3, TRAP	360	119	10425	2.433473	0.99815
10	1.336898	0.028374	IFIT3, TRAP	702	126	20594	2.328268	1
8	1.069519	0.032516	IFIT3, TRAP	638	86	18012	2.62623	1
10	1.336898	0.033406	IFIT3, TRAP	638	125	18012	2.258558	1
10	1.336898	0.042574	IFIT3, TRAP	728	144	22680	2.163462	1
10	1.336898	0.054399	IFIT3, TRAP	638	137	18012	2.060728	1
10	1.336898	0.054399	IFIT3, TRAP	638	137	18012	2.060728	1
11	1.470588	0.159247	IFIT3, TRAP	702	204	20594	1.581853	1
5	0.668449	0.190256	RPAP3, IFIT	638	64	18012	2.205623	1
4	0.534759	0.269274	RPAP3, IFIT	638	51	18012	2.214273	1
4	0.534759	0.327646	RPAP3, IFIT	638	57	18012	1.981191	1
3	0.40107	0.403475	IFIT1, TTC3	638	39	18012	2.17169	1

Count	%	PValue	Genes	List Total	Pop Hits	Pop Total	Fold Enrich	Bonferroni
5	0.668449	0.028132	APOBEC1, I	661	32	18082	4.2743	1
3	0.40107	0.070421	APOL9A, AI	702	13	20594	6.769888	1
7	0.935829	0.219655	SLC27A1, S	661	111	18082	1.725123	1

Count	%	PValue	Genes	List Total	Pop Hits	Pop Total	Fold Enrich	Bonferroni
5	0.668449	0.007747	ADI1, PYCR	728	25	22680	6.230769	0.927831
5	0.668449	0.012084	ADI1, PYCR	661	25	18082	5.471104	1
5	0.668449	0.060594	ADI1, TST, (	289	40	7720	3.3391	1
5	0.668449	0.125974	ODC1, CTH	728	60	22680	2.596154	1

6	0.802139	0.165461	RPE, PYCR2	289	78	7720	2.054831	1
3	0.40107	0.432814	CTH, GOT1,	702	43	20594	2.04671	1
3	0.40107	0.432814	CTH, GOT1,	702	43	20594	2.04671	1

Count	%	PValue	Genes	List Total	Pop Hits	Pop Total	Fold Enrich	Bonferroni
11	1.470588	0.010648	DENND6B,	728	133	22680	2.576634	0.973172
13	1.737968	0.01261	DENND6B,	642	156	17446	2.264538	0.999984
7	0.935829	0.054393	ARHGEF3, S	642	74	17446	2.570557	1
5	0.668449	0.185036	ARHGEF3, S	360	65	10425	2.227564	1
3	0.40107	0.196064	ARHGEF3, I	702	24	20594	3.667023	1
5	0.668449	0.200925	ARHGEF3, S	702	68	20594	2.157072	1
5	0.668449	0.309477	ARHGEF3, S	661	77	18082	1.776333	1
4	0.534759	0.347156	ARHGEF3, S	638	59	18012	1.914032	1

Count	%	PValue	Genes	List Total	Pop Hits	Pop Total	Fold Enrich	Bonferroni
4	0.534759	0.01748	ANKRD27,	638	16	18012	7.057994	1
3	0.40107	0.023301	ANKRD28,	638	7	18012	12.09942	1
3	0.40107	0.023301	ANKRD28,	638	7	18012	12.09942	1
3	0.40107	0.023301	ANKRD28,	638	7	18012	12.09942	1
3	0.40107	0.023301	ANKRD28,	638	7	18012	12.09942	1
3	0.40107	0.023301	ANKRD28,	638	7	18012	12.09942	1
3	0.40107	0.030349	ANKRD28,	638	8	18012	10.58699	1
3	0.40107	0.030349	ANKRD28,	638	8	18012	10.58699	1
3	0.40107	0.038119	ANKRD28,	638	9	18012	9.410658	1
3	0.40107	0.038119	ANKRD28,	638	9	18012	9.410658	1
3	0.40107	0.046553	ANKRD28,	638	10	18012	8.469592	1
5	0.668449	0.048	ANKRD27,	638	39	18012	3.619484	1
4	0.534759	0.051341	ANKRD27,	638	24	18012	4.705329	1
5	0.668449	0.117202	ANKRD27,	638	53	18012	2.663394	1
4	0.534759	0.11769	ANKRD27,	638	34	18012	3.321409	1
6	0.802139	0.142034	ANKRD27,	638	78	18012	2.17169	1
6	0.802139	0.33515	ANKRD27,	638	108	18012	1.568443	1
6	0.802139	0.468251	ANKRD27,	638	127	18012	1.333794	1
9	1.203209	0.504308	ANKRD27,	728	240	22680	1.168269	1
9	1.203209	0.526569	ANKRD27,	360	225	10425	1.158333	1
9	1.203209	0.533569	ANKRD27,	702	232	20594	1.138042	1
9	1.203209	0.583544	ANKRD27,	702	242	20594	1.091015	1
6	0.802139	0.672181	ANKRD27,	638	160	18012	1.058699	1
7	0.935829	0.682175	ANKRD27,	638	193	18012	1.023958	1
7	0.935829	0.682175	ANKRD27,	638	193	18012	1.023958	1

Count	%	PValue	Genes	List Total	Pop Hits	Pop Total	Fold Enrich	Bonferroni
5	0.668449	0.005469	MGST3, PR	642	20	17446	6.793614	0.991577
7	0.935829	0.015995	MGST3, OD	289	55	7720	3.399811	0.981955
4	0.534759	0.119775	MGST3, GS	642	33	17446	3.293873	1
4	0.534759	0.429347	MGST3, GS	289	64	7720	1.66955	1
4	0.534759	0.449193	MGST3, GS	289	66	7720	1.618958	1

4	0.534759	0.673214	MGST3, GS	289	92	7720	1.161426	1

Count	%	PValue	Genes	List Total	Pop Hits	Pop Total	Fold Enrich	Bonferroni
23	3.074866	0.057658	TRP53, RBE	661	420	18082	1.49804	1
15	2.005348	0.066514	REV1, POLI	728	279	22680	1.674938	1
16	2.139037	0.124371	POLI, REV1	728	336	22680	1.483516	1
15	2.005348	0.273729	REV1, POLI	661	318	18082	1.290355	1

Count	%	PValue	Genes	List Total	Pop Hits	Pop Total	Fold Enrich	Bonferroni
6	0.802139	0.048363	SOAT1, SO/	728	62	22680	3.014888	1
7	0.935829	0.086444	SOAT1, SO/	661	84	18082	2.279627	1
6	0.802139	0.088782	SOAT1, SO/	728	74	22680	2.525988	1
5	0.668449	0.09941	SOAT1, SO/	728	55	22680	2.832168	1
7	0.935829	0.106663	CEBPA, SO/	661	89	18082	2.151558	1
4	0.534759	0.405422	SOAT1, SO/	661	63	18082	1.736859	1

Count	%	PValue	Genes	List Total	Pop Hits	Pop Total	Fold Enrich	Bonferroni
3	0.40107	0.01936	RBP1, CRAI	728	7	22680	13.35165	0.99865
3	0.40107	0.02527	RBP1, CRAI	728	8	22680	11.68269	0.999825
3	0.40107	0.032577	RBP1, CRAI	642	8	17446	10.19042	1
3	0.40107	0.059514	RBP1, CRAI	642	11	17446	7.411215	1
3	0.40107	0.069722	RBP1, CRAI	642	12	17446	6.793614	1
3	0.40107	0.101397	RBP1, CRAI	702	16	20594	5.500534	1
3	0.40107	0.654012	RBP1, CRAI	702	65	20594	1.353978	1
3	0.40107	0.708287	RBP1, CRAI	702	72	20594	1.222341	1
3	0.40107	0.715431	RBP1, CRAI	702	73	20594	1.205597	1

Count	%	PValue	Genes	List Total	Pop Hits	Pop Total	Fold Enrich	Bonferroni
4	0.534759	0.05065	HTRA1, IGF	642	23	17446	4.725992	1
6	0.802139	0.059991	HSD3B7, H	661	58	18082	2.829882	1
5	0.668449	0.067351	FLT1, HTRA	642	42	17446	3.235054	1
3	0.40107	0.081805	HTRA1, IGF	728	15	22680	6.230769	1
3	0.40107	0.131738	HTRA1, IGF	638	18	18012	4.705329	1
3	0.40107	0.137832	HTRA1, IGF	360	19	10425	4.572368	1
3	0.40107	0.14712	HTRA1, IGF	702	20	20594	4.400427	1
5	0.668449	0.64995	HTRA1, IGF	702	130	20594	1.128315	1

Count	%	PValue	Genes	List Total	Pop Hits	Pop Total	Fold Enrich	Bonferroni
4	0.534759	0.079567	RPGR, HER	88	25	2126	3.865455	0.874163
3	0.40107	0.135316	RPGR, HER	702	19	20594	4.632029	1
3	0.40107	0.14712	RPGR, HER	702	20	20594	4.400427	1

Count	%	PValue	Genes	List Total	Pop Hits	Pop Total	Fold Enrich	Bonferroni
14	1.871658	0.066455	NDUFAF6,	728	254	22680	1.717141	1

5	0.668449	0.083825	SLC25A33,	638	47	18012	3.003402	1
5	0.668449	0.100302	SLC25A33,	702	52	20594	2.820787	1
5	0.668449	0.100302	SLC25A33,	702	52	20594	2.820787	1
5	0.668449	0.105503	SLC25A33,	638	51	18012	2.767841	1
5	0.668449	0.105503	SLC25A33,	638	51	18012	2.767841	1
11	1.470588	0.493793	MRPL24, R	642	264	17446	1.132269	1

Count	%	PValue	Genes	List Total	Pop Hits	Pop Total	Fold Enrich	Bonferroni
9	1.203209	0.029034	STYK1, FLT	728	113	22680	2.48128	0.999953
8	1.069519	0.05211	STYK1, FLT	702	99	20594	2.370601	1
5	0.668449	0.066073	LAMA3, EP	661	42	18082	3.25661	1
9	1.203209	0.07766	STYK1, FLT	642	121	17446	2.02124	1
5	0.668449	0.082419	STYK1, RIP	642	45	17446	3.019384	1
9	1.203209	0.102485	STYK1, FLT	702	139	20594	1.899465	1
5	0.668449	0.136265	FLT1, EPHA	642	54	17446	2.516153	1
6	0.802139	0.142002	FLT1, EPHA	702	81	20594	2.173051	1
6	0.802139	0.146586	FLT1, EPHA	360	81	10425	2.145062	1
7	0.935829	0.158732	STYK1, FLT	661	100	18082	1.914887	1
5	0.668449	0.212831	FLT1, DYRK	661	65	18082	2.104271	1
4	0.534759	0.432372	S100A6, ST	695	68	19662	1.664156	1
3	0.40107	0.444275	STYK1, FES,	661	41	18082	2.001624	1

Count	%	PValue	Genes	List Total	Pop Hits	Pop Total	Fold Enrich	Bonferroni
9	1.203209	0.038104	POLR3H, P	289	103	7720	2.334128	0.999937
4	0.534759	0.094404	POLR3H, P	728	34	22680	3.665158	1
4	0.534759	0.099625	POLR3H, P	289	30	7720	3.561707	1
4	0.534759	0.136377	POLR3H, P	642	35	17446	3.105652	1
3	0.40107	0.140178	POLR3H, P	642	18	17446	4.529076	1
3	0.40107	0.1435	POLR3H, P	695	19	19662	4.466944	1
3	0.40107	0.151436	POLR3H, P	661	19	18082	4.319293	1
9	1.203209	0.351808	XDH, ADSS,	289	179	7720	1.343102	1

Count	%	PValue	Genes	List Total	Pop Hits	Pop Total	Fold Enrich	Bonferroni
7	0.935829	0.072907	NRP2, EPH	702	86	20594	2.387829	1
3	0.40107	0.149826	NRP2, CNT	360	20	10425	4.34375	1
3	0.40107	0.171298	NRP2, CNT	702	22	20594	4.000389	1

Count	%	PValue	Genes	List Total	Pop Hits	Pop Total	Fold Enrich	Bonferroni
4	0.534759	0.081559	SAV1, ARH	638	29	18012	3.894066	1
4	0.534759	0.081559	SAV1, ARH	638	29	18012	3.894066	1
4	0.534759	0.219406	SAV1, ARH	360	47	10425	2.464539	1
4	0.534759	0.250951	SAV1, ARH	702	51	20594	2.300877	1

Count	%	PValue	Genes	List Total	Pop Hits	Pop Total	Fold Enrich	Bonferroni
17	2.272727	0.041623	TRP53, CE	642	270	17446	1.710984	1

26	3.475936	0.150326	AKNA, TRP!	728	624	22680	1.298077	1
15	2.005348	0.443404	CEBPA, AKI	642	359	17446	1.135423	1

Count	%	PValue	Genes	List Total	Pop Hits	Pop Total	Fold Enrich	Bonferroni
7	0.935829	0.054393	ARHGEF3, !	642	74	17446	2.570557	1
21	2.807487	0.067744	ARHGEF3, I	702	409	20594	1.506259	1
12	1.604278	0.261381	ARHGEF3, I	360	253	10425	1.373518	1
12	1.604278	0.282081	ARHGEF3, I	702	262	20594	1.343642	1
10	1.336898	0.316208	ARHGEF3, I	638	208	18012	1.357306	1

Count	%	PValue	Genes	List Total	Pop Hits	Pop Total	Fold Enrich	Bonferroni
4	0.534759	0.046095	XDH, DLD, I	638	23	18012	4.909909	1
9	1.203209	0.058087	XDH, CYB5I	728	130	22680	2.156805	1
8	1.069519	0.085369	XDH, CYB5I	728	118	22680	2.112125	1
5	0.668449	0.272237	XDH, DLD, I	642	72	17446	1.887115	1
4	0.534759	0.347156	XDH, CYB5I	638	59	18012	1.914032	1
3	0.40107	0.637095	L2HGDH, D	702	63	20594	1.396961	1

Count	%	PValue	Genes	List Total	Pop Hits	Pop Total	Fold Enrich	Bonferroni
3	0.40107	0.085833	IGFBP6, NII	638	14	18012	6.049709	1
3	0.40107	0.112423	IGFBP6, NII	702	17	20594	5.176973	1
3	0.40107	0.114559	IGFBP6, NII	360	17	10425	5.110294	1
5	0.668449	0.64995	HTRA1, IGF	702	130	20594	1.128315	1

Count	%	PValue	Genes	List Total	Pop Hits	Pop Total	Fold Enrich	Bonferroni
3	0.40107	0.169	ITGB7, CAC	638	21	18012	4.033139	1
3	0.40107	0.169	ITGB7, CAC	638	21	18012	4.033139	1
3	0.40107	0.169	ITGB7, CAC	638	21	18012	4.033139	1
3	0.40107	0.169	ITGB7, CAC	638	21	18012	4.033139	1

Count	%	PValue	Genes	List Total	Pop Hits	Pop Total	Fold Enrich	Bonferroni
9	1.203209	0.09329	WDFY1, SP	702	136	20594	1.941365	1
4	0.534759	0.122901	WDFY1, ZF'	702	36	20594	3.259576	1
3	0.40107	0.272125	WDFY1, ZF'	702	30	20594	2.933618	1
3	0.40107	0.276591	WDFY1, ZF'	360	30	10425	2.895833	1

Count	%	PValue	Genes	List Total	Pop Hits	Pop Total	Fold Enrich	Bonferroni
6	0.802139	0.01573	TRIM65, TF	638	42	18012	4.033139	1
12	1.604278	0.023917	TRIM65, TF	702	163	20594	2.159719	1
5	0.668449	0.036166	TRIM30A, T	702	37	20594	3.964349	1
11	1.470588	0.183651	NRP2, TRIN	702	211	20594	1.529374	1
4	0.534759	0.306803	TRIM65, TF	702	57	20594	2.058679	1
4	0.534759	0.347156	TRIM65, TF	638	59	18012	1.914032	1
4	0.534759	0.351044	TRIM30A, T	360	61	10425	1.898907	1

4	0.534759	0.436715	TRIM30A, T	702	71	20594	1.652743	1
4	0.534759	0.480307	TRIM65, TF	360	75	10425	1.544444	1
4	0.534759	0.489796	TRIM65, TF	702	77	20594	1.523958	1
4	0.534759	0.540311	TRIM65, TF	702	83	20594	1.413792	1

Count	%	PValue	Genes	List Total	Pop Hits	Pop Total	Fold Enrich	Bonferroni
3	0.40107	0.119786	ZC3H3, ZC3	638	17	18012	4.982113	1
5	0.668449	0.140954	ZC3H3, ZC3	702	59	20594	2.486117	1
3	0.40107	0.300249	ZC3H3, ZC3	638	31	18012	2.732127	1
3	0.40107	0.300249	ZC3H3, ZC3	638	31	18012	2.732127	1

Count	%	PValue	Genes	List Total	Pop Hits	Pop Total	Fold Enrich	Bonferroni
4	0.534759	0.142937	RNF165, GA	661	36	18082	3.039502	1
3	0.40107	0.204286	GATA4, TGI	661	23	18082	3.568112	1
4	0.534759	0.315274	SEC24B, GA	661	54	18082	2.026335	1

Count	%	PValue	Genes	List Total	Pop Hits	Pop Total	Fold Enrich	Bonferroni
7	0.935829	0.012321	TRP53, CCN	289	52	7720	3.595954	0.954356
9	1.203209	0.012736	TRAF1, TRP	289	84	7720	2.862086	0.958899
11	1.470588	0.015515	TRP53, IRA	289	122	7720	2.408531	0.979629
7	0.935829	0.017371	CEBPA, CCN	289	56	7720	3.3391	0.987266
8	1.069519	0.018619	CCND1, SO	289	73	7720	2.92743	0.990719
7	0.935829	0.031392	TRP53, SLC	289	64	7720	2.921713	0.999644
8	1.069519	0.043201	EREG, PAK3	289	87	7720	2.45635	0.999983
11	1.470588	0.044293	IRF9, CCND	289	145	7720	2.026488	0.999987
6	0.802139	0.056802	TRP53, CCN	289	56	7720	2.862086	1
9	1.203209	0.059958	TRP53, CCN	289	113	7720	2.127568	1
13	1.737968	0.076238	TRP53, CTS	289	203	7720	1.710672	1
4	0.534759	0.092084	TRP53, CCN	289	29	7720	3.684525	1
10	1.336898	0.095115	DDX58, TRI	289	146	7720	1.829644	1
6	0.802139	0.119475	NFKBIB, SO	289	70	7720	2.289669	1
9	1.203209	0.127402	TNFSF10, C	289	134	7720	1.794143	1
6	0.802139	0.130345	TRP53, CCN	289	72	7720	2.226067	1
5	0.668449	0.184669	TRP53, TNF	289	60	7720	2.226067	1
8	1.069519	0.195725	CPT1C, CCN	289	127	7720	1.682696	1
4	0.534759	0.195928	TRP53, CCN	289	41	7720	2.606127	1
5	0.668449	0.215631	TRP53, CCN	289	64	7720	2.086938	1
5	0.668449	0.223587	TRP53, CCN	289	65	7720	2.054831	1
5	0.668449	0.223587	TRP53, CCN	289	65	7720	2.054831	1
17	2.272727	0.235891	TRP53, FLT	289	351	7720	1.293782	1
5	0.668449	0.239719	PAK3, SOS2	289	67	7720	1.993493	1
4	0.534759	0.286031	PIK3CD, PIK	289	50	7720	2.137024	1
18	2.406417	0.316373	TRP53, TRA	289	397	7720	1.21116	1
6	0.802139	0.324675	PLA2G4A, I	289	101	7720	1.586899	1
6	0.802139	0.347003	PAK3, NFKE	289	104	7720	1.541123	1
8	1.069519	0.376131	PLA2G4A, (	289	158	7720	1.352547	1
4	0.534759	0.388954	PLA2G4A, F	289	60	7720	1.780854	1

6	0.802139	0.391856	SLC27A1, P	289	110	7720	1.457062	1
5	0.668449	0.417375	TRP53, CCN	289	88	7720	1.517773	1
12	1.604278	0.458844	TRP53, CCN	289	278	7720	1.153071	1
3	0.40107	0.468295	PIK3CD, PIK	289	42	7720	1.908057	1
4	0.534759	0.468756	PLA2G4A, S	289	68	7720	1.571341	1
4	0.534759	0.497503	TRP53, CCN	289	71	7720	1.504947	1
5	0.668449	0.500089	SOS2, PIK3	289	98	7720	1.362898	1
5	0.668449	0.554873	TNFSF10, S	289	105	7720	1.272038	1
4	0.534759	0.612003	PIK3CD, AR	289	84	7720	1.272038	1
5	0.668449	0.622557	TRP53, CCN	661	117	18082	1.169039	1
3	0.40107	0.652346	PIK3CD, PIK	289	59	7720	1.358278	1
8	1.069519	0.692635	EZR, PAK3,	289	214	7720	0.998609	1
6	0.802139	0.702957	CEBPA, PIK	289	157	7720	1.020871	1
5	0.668449	0.772844	SOS2, PIK3	289	140	7720	0.954029	1
4	0.534759	0.799426	KCNQ5, PIK	289	113	7720	0.945586	1
4	0.534759	0.84748	TRP53, CCN	289	124	7720	0.861703	1
4	0.534759	0.855058	PLA2G4A, F	289	126	7720	0.848025	1
5	0.668449	0.886952	GATA4, PIK	289	171	7720	0.781076	1
8	1.069519	0.896241	TRP53, CCN	289	276	7720	0.774284	1
4	0.534759	0.923272	CACNG8, P	289	150	7720	0.712341	1
3	0.40107	0.949234	TRP53, PIK	289	124	7720	0.646277	1
3	0.40107	0.959425	PLA2G4A, F	289	131	7720	0.611744	1
4	0.534759	0.980097	PTGER3, PI	289	197	7720	0.542392	1

Count	%	PValue	Genes	List Total	Pop Hits	Pop Total	Fold Enrich	Bonferroni
13	1.737968	0.043339	ZFP710, ZF	638	194	18012	1.891833	1
13	1.737968	0.080804	ZFP710, ZF	638	215	18012	1.70705	1
8	1.069519	0.172872	ZFP710, ZF	638	129	18012	1.75082	1
4	0.534759	0.250054	ZFP13, ZFP	638	49	18012	2.304651	1
5	0.668449	0.273853	ZFP710, ZF	638	75	18012	1.882132	1
8	1.069519	0.292308	ZFP710, ZF	638	152	18012	1.485893	1
5	0.668449	0.394468	ZFP710, ZF	638	90	18012	1.568443	1
6	0.802139	0.412649	ZFP710, ZF	638	119	18012	1.423461	1
4	0.534759	0.433636	ZFP93, PRD	638	68	18012	1.660704	1
5	0.668449	0.481322	ZFP710, ZF	638	101	18012	1.397623	1

Count	%	PValue	Genes	List Total	Pop Hits	Pop Total	Fold Enrich	Bonferroni
3	0.40107	0.177551	LCMT2, TR	661	21	18082	3.907932	1
9	1.203209	0.204762	METTL13, T	728	176	22680	1.593094	1
8	1.069519	0.244533	TRMT1L, LC	728	158	22680	1.57741	1
9	1.203209	0.276729	METTL13, T	661	169	18082	1.456803	1
9	1.203209	0.277284	METTL13, T	642	168	17446	1.455774	1

Count	%	PValue	Genes	List Total	Pop Hits	Pop Total	Fold Enrich	Bonferroni
3	0.40107	0.23391	ELF1, ETS1,	702	27	20594	3.259576	1
3	0.40107	0.234049	ELF1, ETS1,	638	26	18012	3.257536	1
3	0.40107	0.237885	ELF1, ETS1,	360	27	10425	3.217593	1

Count	%	PValue	Genes	List Total	Pop Hits	Pop Total	Fold Enrich	Bonferroni
8	1.069519	0.139713	CTSL, USP1	728	134	22680	1.859931	1
9	1.203209	0.148221	CTSL, USP1	642	141	17446	1.73454	1
21	2.807487	0.288312	AMZ1, ERM	728	542	22680	1.207068	1
22	2.941176	0.346263	AMZ1, ERM	642	516	17446	1.158601	1
24	3.208556	0.369958	AMZ1, ERM	661	582	18082	1.128063	1

Count	%	PValue	Genes	List Total	Pop Hits	Pop Total	Fold Enrich	Bonferroni
9	1.203209	0.058087	PTPRE, DUS	728	130	22680	2.156805	1
10	1.336898	0.066124	PTPRE, DUS	661	138	18082	1.982284	1
10	1.336898	0.070759	PTPRE, DUS	642	139	17446	1.954997	1
7	0.935829	0.208025	PTPRE, DUS	661	109	18082	1.756777	1
7	0.935829	0.266739	PTPRE, DUS	642	118	17446	1.612044	1
6	0.802139	0.284837	PTPRE, DUS	642	97	17446	1.680894	1
5	0.668449	0.305161	PTPRE, DUS	702	82	20594	1.788792	1
5	0.668449	0.346022	PTPRE, DUS	638	84	18012	1.680475	1
4	0.534759	0.36306	PTPRE, DUS	702	63	20594	1.862615	1
4	0.534759	0.369987	PTPRE, DUS	360	63	10425	1.838624	1
4	0.534759	0.376301	DUSP2, PTI	638	62	18012	1.821418	1
3	0.40107	0.378295	PTPRE, PTP	360	38	10425	2.286184	1
3	0.40107	0.397113	PTPRE, PTP	702	40	20594	2.200214	1
3	0.40107	0.715431	PTPRE, DUS	702	73	20594	1.205597	1

Count	%	PValue	Genes	List Total	Pop Hits	Pop Total	Fold Enrich	Bonferroni
9	1.203209	0.092668	ANKRD27,	695	131	19662	1.943632	1
14	1.871658	0.386352	IRGM1, ZC	695	331	19662	1.196583	1
10	1.336898	0.400532	ANKRD27,	728	249	22680	1.251158	1

Count	%	PValue	Genes	List Total	Pop Hits	Pop Total	Fold Enrich	Bonferroni
8	1.069519	0.139713	CTSL, USP1	728	134	22680	1.859931	1
9	1.203209	0.148221	CTSL, USP1	642	141	17446	1.73454	1
6	0.802139	0.152964	USP18, ATX	642	77	17446	2.11749	1
3	0.40107	0.217815	ATXN3, FAI	661	24	18082	3.41944	1
5	0.668449	0.317781	USP18, ATX	661	78	18082	1.753559	1
5	0.668449	0.347253	ATXN3, CO	642	81	17446	1.677435	1
6	0.802139	0.666613	USP18, ATX	661	154	18082	1.0658	1

Count	%	PValue	Genes	List Total	Pop Hits	Pop Total	Fold Enrich	Bonferroni
3	0.40107	0.13071	GLUL, CAR	289	17	7720	4.714024	1
7	0.935829	0.326954	ODC1, CTH	728	146	22680	1.493678	1
7	0.935829	0.403214	ODC1, CTH	642	139	17446	1.368498	1

Count	%	PValue	Genes	List Total	Pop Hits	Pop Total	Fold Enrich	Bonferroni

7	0.935829	0.084695	RAD1, ELA(	642	83	17446	2.291821	1
3	0.40107	0.159123	ELAC1, CPS	702	21	20594	4.190883	1
5	0.668449	0.254518	RAD1, ELA(	728	80	22680	1.947115	1
5	0.668449	0.571601	RAD1, ELA(	728	125	22680	1.246154	1
5	0.668449	0.614226	RAD1, ELA(	642	115	17446	1.181498	1

Count	%	PValue	Genes	List Total	Pop Hits	Pop Total	Fold Enrich	Bonferroni
9	1.203209	0.12582	SLC16A1, S	702	146	20594	1.808395	1
5	0.668449	0.19391	SLC16A1, S	702	67	20594	2.189267	1
11	1.470588	0.859647	KCNQ5, SL(	661	364	18082	0.826678	1

Count	%	PValue	Genes	List Total	Pop Hits	Pop Total	Fold Enrich	Bonferroni
5	0.668449	0.082419	FLT3, F3, IL	642	45	17446	3.019384	1
3	0.40107	0.339601	IL11RA1, IL	638	34	18012	2.491057	1
5	0.668449	0.931585	EPHA8, F3,	702	211	20594	0.69517	1

Count	%	PValue	Genes	List Total	Pop Hits	Pop Total	Fold Enrich	Bonferroni
9	1.203209	0.13265	RAP2B, RAI	728	157	22680	1.785889	1
7	0.935829	0.194338	RAP2B, RAI	638	110	18012	1.79658	1
8	1.069519	0.330997	RAP2B, DN.	702	165	20594	1.42236	1
11	1.470588	0.33246	RAP2B, RAI	638	238	18012	1.304839	1
11	1.470588	0.36005	RAP2B, DN.	661	236	18082	1.275046	1
5	0.668449	0.473604	RAP2B, RAI	638	100	18012	1.411599	1
6	0.802139	0.481637	RAP2B, DN.	702	134	20594	1.31356	1

Count	%	PValue	Genes	List Total	Pop Hits	Pop Total	Fold Enrich	Bonferroni
31	4.144385	0.058659	ZFP358, ZFI	702	652	20594	1.394818	1
4	0.534759	0.250054	ZFP13, ZFP!	638	49	18012	2.304651	1
11	1.470588	0.818374	ZFP13, ZFP!	360	367	10425	0.867961	1
11	1.470588	0.820241	ZFP13, ZFP!	702	373	20594	0.865142	1

Count	%	PValue	Genes	List Total	Pop Hits	Pop Total	Fold Enrich	Bonferroni
25	3.342246	0.235365	XDH, CYB5I	728	639	22680	1.218852	1
26	3.475936	0.292033	XDH, CYB5I	642	604	17446	1.169761	1
26	3.475936	0.505826	XDH, CYB5I	661	676	18082	1.052135	1

Count	%	PValue	Genes	List Total	Pop Hits	Pop Total	Fold Enrich	Bonferroni
8	1.069519	0.094408	SASH1, MA	702	114	20594	2.058679	1
5	0.668449	0.375004	SASH1, EPH	702	91	20594	1.611878	1
4	0.534759	0.548622	SASH1, EPH	360	83	10425	1.395582	1
3	0.40107	0.713762	EPHA8, BIC	638	70	18012	1.209942	1

Count	%	PValue	Genes	List Total	Pop Hits	Pop Total	Fold Enrich	Bonferroni

3	0.40107	0.08045	H2-Q4, H2-	642	13	17446	6.271028	1
3	0.40107	0.107779	H2-Q4, H2-	695	16	19662	5.304496	1
4	0.534759	0.228644	H2-Q4, TAF	642	45	17446	2.415507	1
4	0.534759	0.316186	H2-Q4, PR(	702	58	20594	2.023185	1
3	0.40107	0.380281	H2-Q4, H2-	661	36	18082	2.279627	1
4	0.534759	0.418553	H2-Q4, PR(	702	69	20594	1.700648	1
3	0.40107	0.455976	H2-Q4, H2-	702	45	20594	1.955745	1
3	0.40107	0.790189	H2-Q4, H2-	702	85	20594	1.035395	1
3	0.40107	0.856308	H2-Q4, H2-	360	98	10425	0.88648	1
3	0.40107	0.862678	H2-Q4, H2-	702	101	20594	0.871372	1

Count	%	PValue	Genes	List Total	Pop Hits	Pop Total	Fold Enrich	Bonferroni
3	0.40107	0.143945	TADA2A, TI	638	19	18012	4.45768	1
3	0.40107	0.196064	TADA2A, TI	702	24	20594	3.667023	1
3	0.40107	0.444463	TADA2A, TI	702	44	20594	2.000194	1
3	0.40107	0.450598	TADA2A, TI	360	44	10425	1.974432	1
7	0.935829	0.978608	HOXC8, TA	702	345	20594	0.595227	1

Count	%	PValue	Genes	List Total	Pop Hits	Pop Total	Fold Enrich	Bonferroni
3	0.40107	0.246631	TSPAN11, T	702	28	20594	3.143162	1
3	0.40107	0.272125	TSPAN11, T	702	30	20594	2.933618	1
3	0.40107	0.284867	TSPAN11, T	702	31	20594	2.838985	1
3	0.40107	0.365437	TSPAN11, T	86	27	1807	2.334625	1
7	0.935829	0.814927	TSPAN11, H	661	219	18082	0.874377	1

Count	%	PValue	Genes	List Total	Pop Hits	Pop Total	Fold Enrich	Bonferroni
3	0.40107	0.221229	EZR, PTPN3	702	26	20594	3.384944	1
3	0.40107	0.225032	EZR, PTPN3	360	26	10425	3.341346	1
3	0.40107	0.246631	EZR, PTPN3	702	28	20594	3.143162	1
4	0.534759	0.260175	EZR, PTPN3	702	52	20594	2.256629	1
3	0.40107	0.487538	EZR, PTPN3	638	46	18012	1.841216	1
3	0.40107	0.500605	EZR, PTPN3	702	49	20594	1.796093	1
3	0.40107	0.500605	EZR, PTPN3	702	49	20594	1.796093	1
3	0.40107	0.500605	EZR, PTPN3	702	49	20594	1.796093	1
3	0.40107	0.507077	EZR, PTPN3	360	49	10425	1.772959	1

Count	%	PValue	Genes	List Total	Pop Hits	Pop Total	Fold Enrich	Bonferroni
4	0.534759	0.056604	MTG1, MRI	695	25	19662	4.526504	1
14	1.871658	0.340473	MPHOSPH1	695	320	19662	1.237716	1
12	1.604278	0.391644	MRPL24, R	728	308	22680	1.213786	1
8	1.069519	0.476233	MRPL24, R	728	203	22680	1.227738	1
11	1.470588	0.493793	MRPL24, R	642	264	17446	1.132269	1
8	1.069519	0.496968	MRPL24, R	695	188	19662	1.203857	1
4	0.534759	0.912097	MRPL24, R	289	145	7720	0.736905	1

Count	%	PValue	Genes	List Total	Pop Hits	Pop Total	Fold Enrich	Bonferroni
8	1.069519	0.259185	PDZD7, PTI	360	150	10425	1.544444	1
8	1.069519	0.271496	PDZD7, PTI	702	154	20594	1.523958	1
4	0.534759	0.6981	PTPN3, SHI	638	101	18012	1.118098	1

Count	%	PValue	Genes	List Total	Pop Hits	Pop Total	Fold Enrich	Bonferroni
4	0.534759	0.310985	XDH, NUBP	728	61	22680	2.042875	1
4	0.534759	0.419442	XDH, NUBP	642	64	17446	1.698403	1
3	0.40107	0.435112	NUBPL, RS/	642	40	17446	2.038084	1

Count	%	PValue	Genes	List Total	Pop Hits	Pop Total	Fold Enrich	Bonferroni
4	0.534759	0.228644	H2-Q4, TAI	642	45	17446	2.415507	1
4	0.534759	0.230205	IRGM1, TAI	695	47	19662	2.407715	1
4	0.534759	0.595559	CTSL, TAP1	289	82	7720	1.303064	1
6	0.802139	0.783801	CTSL, TAP1	289	174	7720	0.921131	1

Count	%	PValue	Genes	List Total	Pop Hits	Pop Total	Fold Enrich	Bonferroni
9	1.203209	0.09329	WDFY1, SP	702	136	20594	1.941365	1
3	0.40107	0.820645	PHF19, PHI	702	91	20594	0.967127	1
3	0.40107	0.82628	PHF19, PHI	360	91	10425	0.95467	1

Count	%	PValue	Genes	List Total	Pop Hits	Pop Total	Fold Enrich	Bonferroni
3	0.40107	0.284867	EPRS, GSTT	702	31	20594	2.838985	1
3	0.40107	0.467351	EPRS, GSTT	702	46	20594	1.913229	1
6	0.802139	0.494662	PRDX6, EPI	702	136	20594	1.294243	1

Count	%	PValue	Genes	List Total	Pop Hits	Pop Total	Fold Enrich	Bonferroni
7	0.935829	0.22134	TRAF1, CSF	289	109	7720	1.715501	1
12	1.604278	0.303515	TNFSF10, F	289	245	7720	1.308382	1
7	0.935829	0.622216	NAMPT, TN	728	200	22680	1.090385	1
8	1.069519	0.675437	NAMPT, TN	642	214	17446	1.015867	1

Count	%	PValue	Genes	List Total	Pop Hits	Pop Total	Fold Enrich	Bonferroni
8	1.069519	0.195725	CPT1C, CCN	289	127	7720	1.682696	1
4	0.534759	0.506912	CPT1C, NFK	289	72	7720	1.484045	1
4	0.534759	0.727049	CPT1C, PPI	289	100	7720	1.068512	1

Count	%	PValue	Genes	List Total	Pop Hits	Pop Total	Fold Enrich	Bonferroni
8	1.069519	0.163585	MKS1, RPG	728	140	22680	1.78022	1
9	1.203209	0.27046	MKS1, SOR	728	191	22680	1.467982	1
12	1.604278	0.298801	PDZD7, MK	695	257	19662	1.320964	1
3	0.40107	0.313384	MKS1, FAM	661	31	18082	2.647309	1
6	0.802139	0.340699	MKS1, RPG	695	109	19662	1.557283	1

7	0.935829	0.475034	MKS1, RPG	661	151	18082	1.268137	1
7	0.935829	0.591018	MKS1, RPG	661	170	18082	1.126404	1
5	0.668449	0.697596	MKS1, RPG	661	129	18082	1.060292	1
14	1.871658	0.768194	RPGR, POLI	695	435	19662	0.910504	1
3	0.40107	0.906878	MKS1, IFT8	695	111	19662	0.764612	1

Count	%	PValue	Genes	List Total	Pop Hits	Pop Total	Fold Enrich	Bonferroni
3	0.40107	0.300249	EIF4G3, IPC	638	31	18012	2.732127	1
3	0.40107	0.390917	EIF4G3, IPC	638	38	18012	2.22884	1
3	0.40107	0.440371	EIF4G3, IPC	638	42	18012	2.01657	1
3	0.40107	0.521242	EIF4G3, IPC	638	49	18012	1.728488	1
3	0.40107	0.521242	EIF4G3, IPC	638	49	18012	1.728488	1

Count	%	PValue	Genes	List Total	Pop Hits	Pop Total	Fold Enrich	Bonferroni
5	0.668449	0.374807	XDH, PEX11	289	83	7720	1.609205	1
5	0.668449	0.449885	XDH, PEX11	728	107	22680	1.455787	1
6	0.802139	0.493699	XDH, PEX11	695	131	19662	1.295755	1

Count	%	PValue	Genes	List Total	Pop Hits	Pop Total	Fold Enrich	Bonferroni
7	0.935829	0.207858	ECI1, CPT1C	728	124	22680	1.758685	1
7	0.935829	0.407034	CEPT1, CYB	728	160	22680	1.362981	1
7	0.935829	0.506673	ECI1, CPT1C	661	156	18082	1.227491	1
3	0.40107	0.520217	PTGES, PRK	728	54	22680	1.730769	1
3	0.40107	0.757863	PTGES, PRK	661	74	18082	1.109008	1

Count	%	PValue	Genes	List Total	Pop Hits	Pop Total	Fold Enrich	Bonferroni
4	0.534759	0.316186	LAMA3, CN	702	58	20594	2.023185	1
3	0.40107	0.415003	LAMA3, CN	360	41	10425	2.118902	1
8	1.069519	0.700506	LAMA3, ER	702	237	20594	0.990251	1

Count	%	PValue	Genes	List Total	Pop Hits	Pop Total	Fold Enrich	Bonferroni
12	1.604278	0.200666	WDR75, W	728	256	22680	1.460337	1
15	2.005348	0.263443	WDFY1, RB	702	340	20594	1.294243	1
12	1.604278	0.283777	WDR75, W	702	263	20594	1.338533	1
12	1.604278	0.299345	WDR75, W	360	262	10425	1.326336	1
13	1.737968	0.35305	WDR75, W	702	307	20594	1.242249	1
5	0.668449	0.375004	WDFY1, WI	702	91	20594	1.611878	1
10	1.336898	0.486387	RBBP5, WD	638	243	18012	1.16181	1
10	1.336898	0.514367	RBBP5, WD	638	248	18012	1.138386	1
10	1.336898	0.514367	RBBP5, WD	638	248	18012	1.138386	1
6	0.802139	0.644327	WDFY1, WI	702	161	20594	1.093274	1
8	1.069519	0.70887	RBBP5, WD	638	230	18012	0.981982	1
7	0.935829	0.760892	RBBP5, WD	638	211	18012	0.936606	1
5	0.668449	0.846318	RBBP5, NLE	638	167	18012	0.845269	1
3	0.40107	0.942178	NLE1, WDR	638	127	18012	0.666897	1

Count	%	PValue	Genes	List Total	Pop Hits	Pop Total	Fold Enrich	Bonferroni
4	0.534759	0.134455	EZR, EPS8,	661	35	18082	3.126345	1
7	0.935829	0.536544	SH3GL3, SA	638	166	18012	1.190505	1
7	0.935829	0.659679	SH3GL3, SA	728	208	22680	1.048447	1
7	0.935829	0.732021	SH3GL3, SA	702	212	20594	0.968648	1
6	0.802139	0.813854	SH3GL3, SA	360	197	10425	0.88198	1

Count	%	PValue	Genes	List Total	Pop Hits	Pop Total	Fold Enrich	Bonferroni
10	1.336898	0.363202	CCND1, LA	289	207	7720	1.290474	1
6	0.802139	0.443864	LAMA3, PIK	289	117	7720	1.369887	1
4	0.534759	0.64353	LAMA3, ITG	289	88	7720	1.214218	1
4	0.534759	0.670373	LAMA3, NI	695	97	19662	1.166625	1

Count	%	PValue	Genes	List Total	Pop Hits	Pop Total	Fold Enrich	Bonferroni
3	0.40107	0.356814	ARNTL2, M	642	34	17446	2.397746	1
7	0.935829	0.475945	CEBPA, ARI	638	156	18012	1.266819	1
5	0.668449	0.527729	ARNTL2, M	360	110	10425	1.316288	1
5	0.668449	0.539396	ARNTL2, M	702	113	20594	1.298061	1
5	0.668449	0.54854	ARNTL2, M	638	110	18012	1.283272	1
7	0.935829	0.703006	ANO1, ARN	642	190	17446	1.001164	1

Count	%	PValue	Genes	List Total	Pop Hits	Pop Total	Fold Enrich	Bonferroni
5	0.668449	0.418511	RIN1, STAT	638	93	18012	1.517848	1
5	0.668449	0.539396	RIN1, STAT	702	113	20594	1.298061	1
4	0.534759	0.658571	RIN1, STAT	728	105	22680	1.186813	1
4	0.534759	0.694039	RIN1, FES,	360	103	10425	1.124595	1

Count	%	PValue	Genes	List Total	Pop Hits	Pop Total	Fold Enrich	Bonferroni
10	1.336898	0.31	RNF165, HI	642	199	17446	1.36555	1
20	2.673797	0.643067	DTX4, USP	728	631	22680	0.987444	1
13	1.737968	0.674345	DTX4, NUB	661	362	18082	0.982381	1
9	1.203209	0.915598	DTX3L, HEF	642	326	17446	0.750215	1

Count	%	PValue	Genes	List Total	Pop Hits	Pop Total	Fold Enrich	Bonferroni
4	0.534759	0.481113	C1QL1, CO	702	76	20594	1.54401	1
4	0.534759	0.51559	C1QL1, CO	728	85	22680	1.466063	1
8	1.069519	0.630798	LGALS3BP,	728	235	22680	1.060556	1
3	0.40107	0.796115	C1QL1, CO	695	83	19662	1.022554	1

Count	%	PValue	Genes	List Total	Pop Hits	Pop Total	Fold Enrich	Bonferroni
12	1.604278	0.561857	BZW2, KRT	695	316	19662	1.074328	1
11	1.470588	0.577126	BZW2, KRT	642	279	17446	1.071394	1

7	0.935829	0.692098	BZW2, ZC3	661	189	18082	1.013167	1

Count	%	PValue	Genes	List Total	Pop Hits	Pop Total	Fold Enrich	Bonferroni
4	0.534759	0.452332	PLA2G4A, F	638	70	18012	1.613256	1
6	0.802139	0.616663	PLA2G4A, F	702	156	20594	1.128315	1
4	0.534759	0.809964	PLA2G4A, F	360	125	10425	0.926667	1

Count	%	PValue	Genes	List Total	Pop Hits	Pop Total	Fold Enrich	Bonferroni
3	0.40107	0.489669	ABCB1A, TA	702	48	20594	1.833511	1
3	0.40107	0.516566	ABCB1A, TA	289	46	7720	1.742139	1
3	0.40107	0.542053	ABCB1A, TA	642	49	17446	1.663742	1
3	0.40107	0.542809	ABCB1A, TA	702	53	20594	1.660539	1
5	0.668449	0.72666	NVL, ABCB1	702	144	20594	1.018617	1
5	0.668449	0.735972	NVL, ABCB1	360	144	10425	1.005498	1
6	0.802139	0.862837	TOR3A, AB(	642	200	17446	0.815234	1

Count	%	PValue	Genes	List Total	Pop Hits	Pop Total	Fold Enrich	Bonferroni
4	0.534759	0.587826	HMHA1, AF	702	89	20594	1.31848	1
3	0.40107	0.652404	HMHA1, AF	360	64	10425	1.357422	1
3	0.40107	0.658018	HMHA1, AF	638	63	18012	1.34438	1
3	0.40107	0.662233	HMHA1, AF	702	66	20594	1.333463	1

Count	%	PValue	Genes	List Total	Pop Hits	Pop Total	Fold Enrich	Bonferroni
274	36.63102	0.020393	S100A6, CN	695	6998	19662	1.107693	0.99994
132	17.64706	0.982442	TSPAN5, CN	638	4312	18012	0.864244	1
251	33.55615	0.987727	S100A6, CN	728	8683	22680	0.900566	1
57	7.620321	0.998994	NRP2, SLC2	638	2256	18012	0.713308	1
75	10.02674	0.999398	NRP2, JPH4	638	2880	18012	0.735208	1
92	12.29947	0.999903	NRP2, ANO	638	3563	18012	0.728976	1
168	22.45989	0.999999	FAM189B,	728	6938	22680	0.754374	1
168	22.45989	0.999999	FAM189B,	728	6955	22680	0.75253	1
169	22.59358	1	FAM189B,	695	6878	19662	0.695132	1

Count	%	PValue	Genes	List Total	Pop Hits	Pop Total	Fold Enrich	Bonferroni
10	1.336898	0.462736	NETO2, EPS	695	239	19662	1.183709	1
12	1.604278	0.595847	CPT1C, PRF	728	357	22680	1.047188	1
17	2.272727	0.706468	NETO2, CPT	695	505	19662	0.952358	1
17	2.272727	0.903251	CPT1C, SEP	728	661	22680	0.801234	1
19	2.540107	0.952902	CPT1C, NAI	695	718	19662	0.748638	1

Count	%	PValue	Genes	List Total	Pop Hits	Pop Total	Fold Enrich	Bonferroni
4	0.534759	0.570062	CACNG8, IT	289	79	7720	1.352547	1
3	0.40107	0.749141	CACNG8, IT	289	71	7720	1.12871	1
3	0.40107	0.821892	CACNG8, IT	289	83	7720	0.965523	1

Count	%	PValue	Genes	List Total	Pop Hits	Pop Total	Fold Enrich	Bonferroni
5	0.668449	0.57792	TRP53, NAI	728	126	22680	1.236264	1
5	0.668449	0.691775	TRP53, NAI	661	128	18082	1.068575	1
3	0.40107	0.908867	TRP53, NAI	661	108	18082	0.759876	1

Count	%	PValue	Genes	List Total	Pop Hits	Pop Total	Fold Enrich	Bonferroni
8	1.069519	0.072665	LINGO1, FL	638	103	18012	2.192775	1
4	0.534759	0.880881	LINGO1, FL	702	147	20594	0.798263	1
10	1.336898	0.97302	LINGO1, FL	728	481	22680	0.647689	1
4	0.534759	0.989825	LINGO1, FL	702	242	20594	0.484896	1
4	0.534759	0.990877	LINGO1, FL	360	242	10425	0.47865	1
8	1.069519	0.998948	LINGO1, FL	702	518	20594	0.453068	1
8	1.069519	0.999187	LINGO1, FL	360	518	10425	0.447233	1
20	2.673797	0.999792	H2-Q4, FLT	702	1099	20594	0.53387	1
13	1.737968	0.999985	LINGO1, H2	702	920	20594	0.414533	1

Count	%	PValue	Genes	List Total	Pop Hits	Pop Total	Fold Enrich	Bonferroni
6	0.802139	0.610094	LGALS3BP,	638	149	18012	1.136858	1
6	0.802139	0.875534	LGALS3BP,	360	218	10425	0.797018	1
6	0.802139	0.8777	LGALS3BP,	702	222	20594	0.79287	1
6	0.802139	0.899649	LGALS3BP,	702	232	20594	0.758694	1

Count	%	PValue	Genes	List Total	Pop Hits	Pop Total	Fold Enrich	Bonferroni
8	1.069519	0.700506	LAMA3, ER	702	237	20594	0.990251	1
3	0.40107	0.706293	EREG, HBE(	638	69	18012	1.227477	1
7	0.935829	0.727324	EREG, HBE(	728	224	22680	0.973558	1
3	0.40107	0.802495	NID1, CNTN	638	84	18012	1.008285	1
5	0.668449	0.906517	EREG, HBE(	702	197	20594	0.744573	1
3	0.40107	0.907522	NID1, CNTN	638	111	18012	0.763026	1
4	0.534759	0.951791	LAMA3, NII	360	181	10425	0.639963	1

Count	%	PValue	Genes	List Total	Pop Hits	Pop Total	Fold Enrich	Bonferroni
3	0.40107	0.685952	PCDH7, PCI	702	69	20594	1.275486	1
4	0.534759	0.802784	CLSTN1, PC	702	125	20594	0.938758	1
4	0.534759	0.805625	CLSTN1, PC	360	124	10425	0.93414	1
4	0.534759	0.811348	CLSTN1, PC	702	127	20594	0.923974	1
3	0.40107	0.906467	PCDH7, PCI	702	115	20594	0.765292	1
4	0.534759	0.93987	CLSTN1, PC	661	163	18082	0.671301	1

Count	%	PValue	Genes	List Total	Pop Hits	Pop Total	Fold Enrich	Bonferroni
5	0.668449	0.496622	LINGO1, PF	638	103	18012	1.370484	1
4	0.534759	0.606358	LINGO1, TL	638	88	18012	1.283272	1
5	0.668449	0.636875	LINGO1, PF	638	123	18012	1.147641	1

3	0.40107	0.674779	LINGO1, TL	638	65	18012	1.303014	1
5	0.668449	0.702261	LINGO1, PF	638	134	18012	1.053432	1
3	0.40107	0.774036	LINGO1, TL	638	79	18012	1.0721	1
5	0.668449	0.814321	LINGO1, PF	638	158	18012	0.893417	1
5	0.668449	0.909688	LINGO1, PF	638	191	18012	0.739057	1
4	0.534759	0.939924	LINGO1, PF	702	175	20594	0.670541	1
5	0.668449	0.942144	LINGO1, PF	638	210	18012	0.67219	1
6	0.802139	0.942416	LINGO1, PF	728	275	22680	0.67972	1
4	0.534759	0.94368	LINGO1, PF	360	175	10425	0.661905	1
5	0.668449	0.962699	LINGO1, PF	638	228	18012	0.619122	1
5	0.668449	0.978092	LINGO1, PF	702	259	20594	0.566336	1
5	0.668449	0.980202	LINGO1, PF	638	253	18012	0.557944	1
5	0.668449	0.988585	LINGO1, PF	638	274	18012	0.515182	1
5	0.668449	0.988585	LINGO1, PF	638	274	18012	0.515182	1

Count	%	PValue	Genes	List Total	Pop Hits	Pop Total	Fold Enrich	Bonferroni
5	0.668449	0.730864	KCNQ5, CA	661	135	18082	1.013167	1
4	0.534759	0.815483	KCNQ5, CA	728	136	22680	0.91629	1
4	0.534759	0.874502	KCNQ5, CA	642	134	17446	0.811178	1
7	0.935829	0.960389	KCNQ5, P2	728	336	22680	0.649038	1

Count	%	PValue	Genes	List Total	Pop Hits	Pop Total	Fold Enrich	Bonferroni
5	0.668449	0.85403	IMMP2L, H	642	163	17446	0.833572	1
6	0.802139	0.893553	IMMP2L, H	642	212	17446	0.769088	1
4	0.534759	0.918614	HTRA1, PCS	728	173	22680	0.72032	1

Count	%	PValue	Genes	List Total	Pop Hits	Pop Total	Fold Enrich	Bonferroni
17	2.272727	0.842946	CACNG8, A	728	619	22680	0.855598	1
16	2.139037	0.943204	CACNG8, A	661	584	18082	0.749466	1
7	0.935829	0.960389	KCNQ5, P2	728	336	22680	0.649038	1

Count	%	PValue	Genes	List Total	Pop Hits	Pop Total	Fold Enrich	Bonferroni
4	0.534759	0.839255	RPL7, IGF2I	638	129	18012	0.87541	1
4	0.534759	0.903835	RPL7, IGF2I	638	150	18012	0.752853	1
3	0.40107	0.910163	RPL7, IGF2I	638	112	18012	0.756214	1
4	0.534759	0.974411	RPL7, IGF2I	638	200	18012	0.564639	1
4	0.534759	0.97645	RPL7, IGF2I	638	203	18012	0.556295	1

Count	%	PValue	Genes	List Total	Pop Hits	Pop Total	Fold Enrich	Bonferroni
8	1.069519	0.825065	S100A6, EF	702	273	20594	0.859668	1
4	0.534759	0.880913	S100A6, EF	360	145	10425	0.798851	1
3	0.40107	0.915234	S100A6, PL	638	114	18012	0.742947	1
3	0.40107	0.940434	S100A6, PL	638	126	18012	0.67219	1
4	0.534759	0.946894	S100A6, S1	638	173	18012	0.652762	1
5	0.668449	0.948053	S100A6, EF	702	223	20594	0.657762	1

4	0.534759	0.94828	S100A6, S1	638	174	18012	0.649011	1
3	0.40107	0.983588	S100A6, S1	702	175	20594	0.502906	1

Count	%	PValue	Genes	List Total	Pop Hits	Pop Total	Fold Enrich	Bonferroni
8	1.069519	0.865406	APOBEC1, /	728	307	22680	0.811827	1
3	0.40107	0.878878	MAGOHB, (	289	96	7720	0.834775	1
9	1.203209	0.905387	APOBEC1, /	661	322	18082	0.764595	1
3	0.40107	0.996518	C1QBP, MA	728	240	22680	0.389423	1
3	0.40107	0.998762	C1QBP, MA	661	241	18082	0.340525	1

Count	%	PValue	Genes	List Total	Pop Hits	Pop Total	Fold Enrich	Bonferroni
16	2.139037	0.904551	TRP53, TXN	728	626	22680	0.796264	1
8	1.069519	0.955969	SEPT3, CCN	728	372	22680	0.669975	1
16	2.139037	0.964863	TRP53, TXN	661	614	18082	0.712847	1
6	0.802139	0.975516	TADA2A, H	661	277	18082	0.592538	1
8	1.069519	0.984661	SEPT3, CCN	661	374	18082	0.585145	1
4	0.534759	0.990285	HMGA2, TF	728	259	22680	0.48114	1

Count	%	PValue	Genes	List Total	Pop Hits	Pop Total	Fold Enrich	Bonferroni
46	6.149733	0.896285	XDH, NAMI	695	1504	19662	0.865272	1
42	5.614973	0.977415	XDH, NAMI	728	1685	22680	0.776535	1
46	6.149733	0.992809	XDH, NAMI	695	1753	19662	0.742367	1
81	10.82888	0.999692	NRP2, IGFB	638	3124	18012	0.732007	1

Count	%	PValue	Genes	List Total	Pop Hits	Pop Total	Fold Enrich	Bonferroni
7	0.935829	0.978608	HOXC8, TA	702	345	20594	0.595227	1
3	0.40107	0.987481	HOXC8, HC	702	184	20594	0.478307	1
3	0.40107	0.988633	HOXC8, HC	638	180	18012	0.470533	1
4	0.534759	0.994404	HOXC8, ZH	728	280	22680	0.445055	1
4	0.534759	0.995418	HOXC8, ZH	360	266	10425	0.435464	1
4	0.534759	0.995484	HOXC8, ZH	702	271	20594	0.433006	1

Count	%	PValue	Genes	List Total	Pop Hits	Pop Total	Fold Enrich	Bonferroni
108	14.4385	0.946132	FAM189B,	728	3815	22680	0.881944	1
82	10.96257	0.986019	NRP2, IGFB	728	3124	22680	0.817739	1
57	7.620321	0.998994	NRP2, SLC2	638	2256	18012	0.713308	1
75	10.02674	0.999398	NRP2, JPH4	638	2880	18012	0.735208	1
81	10.82888	0.999692	NRP2, IGFB	638	3124	18012	0.732007	1
62	8.28877	0.999714	NRP2, IGFB	638	2510	18012	0.697364	1
92	12.29947	0.999903	NRP2, ANO	638	3563	18012	0.728976	1
108	14.4385	0.999931	RAB3GAP2,	728	4543	22680	0.740615	1

Benjamini	FDR
1.88E-16	1.18E-16
3.17E-13	4.00E-13
7.51E-14	6.00E-13
8.63E-13	1.04E-11
6.27E-12	1.01E-10
5.70E-08	1.07E-07
3.35E-07	1.05E-06
1.26E-06	4.72E-06

Benjamini	FDR
1.18E-07	3.33E-06
1.55E-07	4.98E-06
1.18E-04	0.005693
0.004556	0.024408

Benjamini	FDR
2.21E-05	3.94E-05
1.23E-05	4.38E-05
4.63E-05	5.74E-05
4.63E-05	5.74E-05
2.66E-05	6.58E-05
6.30E-05	2.34E-04
0.015257	0.114176
0.138744	1.116542
0.076362	1.684312
0.032926	3.962477
0.716119	18.54298

Benjamini	FDR
6.57E-08	1.65E-07
3.89E-04	0.001928
0.061512	1.124944
0.921557	68.2754

Benjamini	FDR
6.49E-04	0.003384
3.27E-04	0.003409
4.25E-04	0.006641
0.009577	0.200489

Benjamini	FDR
3.24E-06	1.17E-04
0.004584	0.350753

0.847444	16.0769

Benjamini	FDR
2.46E-08	5.94E-07
6.91E-04	0.044532
0.003818	0.261443
0.421981	5.670465
0.202033	6.608607
0.05441	6.953095
0.492405	30.40677
0.884187	47.70314

Benjamini	FDR
0.003584	0.02223
0.047849	0.696563
0.007669	0.648674
0.061512	1.124944
0.875621	27.66343

Benjamini	FDR
5.40E-04	0.030423
0.015404	0.110597
0.069491	0.49508
0.028837	0.911206
0.014097	1.418545
0.644654	4.196029
0.032182	3.7466
0.32547	5.683602
0.275011	11.84569
0.688572	19.48397
0.884848	21.82849
0.769366	26.57019
0.835334	37.44542
0.887386	50.22691
0.941409	69.71913

Benjamini	FDR
0.026802	0.235244
0.492824	0.7007
0.083751	0.862839
0.06303	1.268086
0.89411	32.65594
0.221494	37.7306
0.980462	91.67256

Benjamini	FDR
0.016156	1.690274
0.359571	12.61557
0.485833	24.5596

Benjamini	FDR
0.029392	0.318464
0.340182	4.528674
0.340182	4.528674
0.56974	3.803195
0.57341	5.156622
0.688572	19.48397
0.782218	30.13929
0.725881	25.73312
0.966809	82.25706
0.996159	99.00995

Benjamini	FDR
0.316506	4.603432
0.353276	3.928294
0.617149	14.32215
0.797886	32.70184
0.915548	43.06068

Benjamini	FDR
0.357206	4.067674
0.446137	6.44526
0.202033	6.608607
0.524824	89.10027
0.959236	98.52984

Benjamini	FDR
0.119081	3.02291
0.640945	16.27164
0.715671	19.16146
0.688572	19.48397
0.822197	33.71011
0.891836	52.21432
0.995637	99.4532

Benjamini	FDR
3.17E-05	0.001403
4.75E-04	0.024843
0.003729	0.270297
0.029842	0.377207

0.047286	0.773886
0.326466	16.14422
0.773054	26.40453
0.863976	28.1506
0.155009	24.78031
0.902043	43.87045
0.743356	59.21112
0.953034	82.50864
0.962589	87.74892
0.969941	85.81686
0.880093	85.45136
0.98875	89.25714
0.989813	90.7012
0.980081	91.15169
0.996895	99.41959
0.81396	99.94549
0.993522	99.9984
0.996906	99.99757
1	100

Benjamini	FDR
0.732481	22.99318
0.732481	22.99318
0.732481	22.99318
0.792271	19.48716
0.882386	25.03823
0.782218	30.13929
0.782218	30.13929
0.725881	25.73312
0.963306	78.89035

Benjamini	FDR
0.084345	11.67483
0.493365	23.47117
0.844789	79.37289

Benjamini	FDR
0.89411	32.65594
0.835334	37.44542
0.755969	32.23192
0.925351	64.24174

Benjamini	FDR
0.782218	30.13929
0.867722	51.64049
0.867722	51.64049

Benjamini	FDR
0.53454	2.348192
0.574624	4.330146
0.852513	17.98299
0.842221	42.2385
0.692612	38.62152
0.863577	49.88023
0.936639	52.42966
0.923918	62.80336
0.974947	75.65566
0.996622	95.85239
0.999765	99.69656
0.99552	99.9996

Benjamini	FDR
0.516136	23.1739
0.742311	54.14441
0.352166	63.06759

Benjamini	FDR
0.100653	14.97932
0.223562	38.67104
0.899954	56.04632
0.987275	97.13944

Benjamini	FDR
0.335098	5.408011
0.082663	11.75316
0.302868	15.57433
0.465512	27.65616
0.835334	37.44542
0.746256	35.68414
0.851669	41.93283
0.843488	44.96399
0.732825	38.45764
0.94871	63.76788
0.754976	61.41748
0.789277	66.11671
0.816253	73.51004
0.959228	76.92017
0.988945	87.75016
0.987849	88.31622
0.960327	95.67968
0.999055	100

Benjamini	FDR
0.867722	51.64049
0.867722	51.64049
0.730243	45.24957
0.989239	95.91994

Benjamini	FDR
0.255239	44.08089
0.525196	66.29144
0.95928	85.98704

Benjamini	FDR
0.802812	31.36225
0.876092	45.21498
0.891916	52.88724
0.938216	74.78781
0.990558	98.1172
0.998603	99.94204

Benjamini	FDR
0.796994	29.91428
0.792606	25.12141
0.822425	37.55645
0.9218	42.55335
0.908521	43.43285
0.254805	44.67546
0.946134	60.85751
0.946134	60.85751
0.984488	94.1442
0.997995	97.0953
0.999642	99.4808
0.999867	99.87146
0.99994	99.98272

Benjamini	FDR
0.856651	40.01532
0.941409	69.71913
0.993219	98.82285

Benjamini	FDR
0.076569	10.04112
0.711013	19.56686
0.463437	55.58158
0.482924	83.99001

0.632431	90.44654
0.999779	99.99065
0.999779	99.99065

Benjamini	FDR
0.093163	13.5541
0.346303	17.87814
0.741573	58.02438
0.975172	93.4036
0.990437	97.18527
0.990934	97.45116
0.997878	99.86828
0.999872	99.92155

Benjamini	FDR
0.869875	25.5991
0.89411	32.65594
0.89411	32.65594
0.89411	32.65594
0.89411	32.65594
0.89411	32.65594
0.917486	40.3571
0.917486	40.3571
0.91929	47.8849
0.91929	47.8849
0.936481	55.03951
0.935828	56.1705
0.941828	58.67938
0.989815	87.63596
0.988945	87.75016
0.992273	92.33742
0.999868	99.89351
0.999979	99.99749
0.861479	99.99287
0.997528	99.99516
0.999949	99.99962
0.999978	99.99994
1	100
1	100
1	100

Benjamini	FDR
0.212459	8.160154
0.305797	18.8877
0.877127	86.19572
0.817945	99.93129
0.82213	99.9566

0.913094	99.99995

Benjamini	FDR
0.92067	65.48
0.339944	60.79924
0.48323	83.58589
0.996704	99.67473

Benjamini	FDR
0.28002	49.05679
0.948752	80.1957
0.407704	71.77542
0.419849	75.93961
0.961507	86.73592
0.999363	99.99096

Benjamini	FDR
0.148848	23.35515
0.178494	29.40716
0.594027	40.19384
0.754976	61.41748
0.792727	67.4295
0.965358	82.60698
0.999995	100
0.999999	100
0.999999	100

Benjamini	FDR
0.725692	55.37029
0.920084	66.97918
0.789277	66.11671
0.391864	68.6888
0.990603	90.64051
0.963004	86.06153
0.981059	92.59832
0.999995	100

Benjamini	FDR
0.874163	48.96573
0.980152	90.73191
0.981059	92.59832

Benjamini	FDR
0.344659	60.76578

0.9736	76.96321
0.966809	82.25706
0.966809	82.25706
0.986402	84.58154
0.986402	84.58154
0.992856	99.99743

Benjamini	FDR
0.194662	33.02676
0.905253	58.3362
0.928708	70.60309
0.81297	71.48583
0.810783	73.68608
0.96391	82.94843
0.905845	89.70715
0.979569	91.83709
0.962073	87.82889
0.984277	95.47557
0.992457	98.62419
0.983627	99.9693
0.999638	99.99731

Benjamini	FDR
0.433918	39.61087
0.422737	74.05493
0.536321	74.39713
0.901915	89.72801
0.90454	90.40764
0.890907	89.05793
0.981914	94.71891
0.767508	99.64076

Benjamini	FDR
0.942571	71.01742
0.956162	88.42877
0.988113	95.37617

Benjamini	FDR
0.973344	75.98852
0.973344	75.98852
0.976894	96.27893
0.996042	99.1149

Benjamini	FDR
0.663482	48.30851

0.524824	89.10027
0.987715	99.98877

Benjamini	FDR
0.741573	58.02438
0.939678	68.26089
0.977784	98.21467
0.997082	99.55801
0.999848	99.82944

Benjamini	FDR
0.94097	54.67599
0.322249	55.70088
0.40023	70.30268
0.964377	99.27909
0.999872	99.92155
0.999994	99.99999

Benjamini	FDR
0.973545	77.79537
0.972139	85.78802
0.959099	80.14294
0.999995	100

Benjamini	FDR
0.9965	95.51457
0.9965	95.51457
0.9965	95.51457
0.9965	95.51457

Benjamini	FDR
0.963744	79.85408
0.976853	88.29747
0.996552	99.44632
0.976315	98.6459

Benjamini	FDR
0.882234	23.34559
0.797886	32.70184
0.835074	45.2636
0.990162	96.38339
0.997644	99.75087
0.999872	99.92155
0.990227	99.68016

0.999781	99.99165
0.996759	99.98328
0.999885	99.99835
0.999948	99.9997

Benjamini	FDR
0.988786	88.22906
0.980462	91.67256
0.999797	99.74887
0.999797	99.74887

Benjamini	FDR
0.979106	93.68687
0.991509	98.33068
0.998017	99.88674

Benjamini	FDR
0.356596	14.8664
0.328986	15.33054
0.322511	18.37314
0.304849	20.34869
0.302311	21.65136
0.41108	33.90597
0.439403	43.6362
0.431094	44.46608
0.469058	53.19702
0.473497	55.19003
0.518731	64.28298
0.528432	71.46883
0.529589	72.68117
0.575258	80.8313
0.590572	82.95472
0.59003	83.68627
0.660954	92.93897
0.669401	94.08588
0.662426	94.10524
0.687453	95.72847
0.688681	96.25801
0.688681	96.25801
0.691275	96.9589
0.691675	97.15088
0.735951	98.74006
0.751602	99.28303
0.757471	99.38821
0.770965	99.6046
0.778244	99.78135
0.788297	99.83303

0.787324	99.84304
0.809984	99.91003
0.827515	99.9655
0.82581	99.97256
0.822854	99.97286
0.838445	99.98682
0.837527	99.98767
0.866733	99.99727
0.89409	99.99954
0.999977	100
0.902523	99.99989
0.920527	99.99998
0.924482	99.99999
0.950232	100
0.958201	100
0.971965	100
0.973109	100
0.980976	100
0.983229	100
0.98856	100
0.994014	100
0.995769	100
0.998626	100

Benjamini	FDR
0.936639	52.42966
0.974947	75.65566
0.996622	95.85239
0.999386	99.19756
0.99961	99.53275
0.999765	99.69656
0.999929	99.97778
0.999944	99.98667
0.999964	99.99276
0.999981	99.99834

Benjamini	FDR
0.987727	96.98295
0.611086	95.5723
0.667831	97.79697
0.996712	99.69797
0.965515	99.35291

Benjamini	FDR
0.995552	98.72113
0.999156	98.85661
0.976211	97.29344

Benjamini	FDR
0.506615	87.09478
0.902595	91.70964
0.709491	99.02215
0.976236	99.86361
0.999007	99.97449

Benjamini	FDR
0.322249	55.70088
0.926308	70.63219
0.789661	67.98861
0.991746	98.46573
0.964427	99.18975
0.96647	99.45026
0.99773	99.74104
0.999882	99.91924
0.99917	99.93762
0.991508	99.78424
0.99992	99.96353
0.991231	99.81912
0.999579	99.97461
0.999999	100

Benjamini	FDR
0.852291	75.06851
0.975709	99.90652
0.798401	99.90531

Benjamini	FDR
0.506615	87.09478
0.902595	91.70964
0.90656	92.39796
0.993134	98.77213
0.998065	99.89394
0.975654	99.86678
0.99999	100

Benjamini	FDR
0.58188	83.77478
0.748339	99.54249
0.985034	99.96686

Benjamini	FDR

0.812819	74.68086
0.985588	94.13015
0.672246	98.16176
0.891511	99.99902
0.997953	99.99996

Benjamini	FDR
0.975294	88.91861
0.99061	97.05935
1	100

Benjamini	FDR
0.810783	73.68608
0.999871	99.90486
1	100

Benjamini	FDR
0.497001	85.57611
0.998064	97.33133
0.998459	99.86068
0.999872	99.88605
0.998832	99.96627
0.99998	99.99788
0.999882	99.99785

Benjamini	FDR
0.923918	62.80336
0.999386	99.19756
0.999972	100
1	100

Benjamini	FDR
0.655999	97.40403
0.968493	99.53013
0.999843	99.99967

Benjamini	FDR
0.962182	80.25651
0.999327	99.95423
0.997245	99.99743
1	100

Benjamini	FDR

0.81711	72.79574
0.833749	80.38626
0.958611	98.2216
0.997967	99.80066
0.999069	99.98103
0.999708	99.98596
0.99984	99.99527
1	100
0.999989	100
1	100

Benjamini	FDR
0.992085	92.61871
0.990437	97.18527
0.999803	99.99334
0.995524	99.96501
1	100

Benjamini	FDR
0.99592	99.02757
0.996552	99.44632
0.996824	99.58526
1	99.23305
1	100

Benjamini	FDR
0.994264	98.32709
0.974873	96.61995
0.99592	99.02757
0.996609	99.27729
0.99998	99.99865
0.999896	99.99883
0.999896	99.99883
0.999896	99.99883
0.997094	99.99172

Benjamini	FDR
0.747199	56.49522
0.971901	99.7382
0.795002	99.88431
0.845609	99.98491
0.992856	99.99743
0.989619	99.99453
0.98593	100

Benjamini	FDR
0.98057	98.14283
0.99676	99.43843
1	100

Benjamini	FDR
0.737993	99.37054
0.986508	99.9784
0.987268	99.98587

Benjamini	FDR
0.958611	98.2216
0.954372	97.61758
0.885522	99.99921
0.953832	100

Benjamini	FDR
0.963744	79.85408
1	100
0.999973	100

Benjamini	FDR
0.996824	99.58526
0.99987	99.99665
0.999889	99.99859

Benjamini	FDR
0.691298	96.11497
0.744524	99.08681
0.911013	99.99982
0.999042	100

Benjamini	FDR
0.669401	94.08588
0.84022	99.98969
0.930889	100

Benjamini	FDR
0.548163	91.19995
0.686054	98.63015
0.967263	99.37188
0.997998	99.88101
0.970137	99.73947

0.999742	99.99903
0.999964	99.99999
0.999995	100
0.998846	100
0.999925	100

Benjamini	FDR
0.999797	99.74887
0.999929	99.97549
0.999963	99.99408
0.99999	99.99957
0.99999	99.99957

Benjamini	FDR
0.781044	99.77525
0.824719	99.97058
0.989841	99.994

Benjamini	FDR
0.612834	95.80112
0.799284	99.91836
0.999841	99.99968
0.867101	99.99543
0.999999	100

Benjamini	FDR
0.997967	99.80066
0.993251	99.91943
0.999999	100

Benjamini	FDR
0.607265	95.25174
0.996617	99.32778
0.996976	99.57479
0.980826	99.11442
0.999023	99.91949
0.999327	99.95423
0.999981	99.99859
0.999988	99.99945
0.999988	99.99945
0.999994	100
1	100
1	100
1	100
1	100

Benjamini	FDR
0.975746	92.46912
0.999992	99.99975
0.924515	99.99996
0.999999	100
0.999974	100

Benjamini	FDR
0.772046	99.71463
0.82072	99.95082
0.899063	99.99985
0.997401	99.99999

Benjamini	FDR
0.976054	99.89405
0.999979	99.99803
0.997148	99.99531
0.999952	99.99969
0.999994	99.99984
0.999385	100

Benjamini	FDR
0.999948	99.98874
0.999952	99.99969
0.925312	99.99996
0.999711	99.99999

Benjamini	FDR
0.971321	99.68471
0.919802	99.99992
0.999992	100
0.999998	100

Benjamini	FDR
0.999891	99.99782
0.865759	99.99479
0.91442	99.99987
0.999159	100

Benjamini	FDR
0.994823	99.99924
0.996729	99.99984

0.999995	100

Benjamini	FDR
0.999969	99.99588
0.999989	99.99998
0.999977	100

Benjamini	FDR
0.999895	99.99834
0.845229	99.99202
0.99508	99.99946
0.999947	99.99973
0.999999	100
0.99987	100
0.999982	100

Benjamini	FDR
0.999978	99.99995
0.999418	99.99992
1	100
0.999996	100

Benjamini	FDR
0.477088	25.49488
1	100
0.999765	100
1	100
1	100
1	100
1	100
1	100
1	100

Benjamini	FDR
0.986598	99.986
0.898245	99.99956
0.998013	100
0.992579	100
0.99999	100

Benjamini	FDR
0.872627	99.99826
0.940537	100
0.96422	100

Benjamini	FDR
0.890154	99.9992
0.999995	100
1	100

Benjamini	FDR
0.966383	71.77733
1	100
0.999067	100
1	100
1	100
1	100
1	100
1	100
1	100

Benjamini	FDR
0.999999	99.99999
0.999994	100
1	100
1	100

Benjamini	FDR
0.999999	100
1	100
0.950628	100
1	100
1	100
1	100
1	100

Benjamini	FDR
0.999998	100
1	100
0.999979	100
1	100
1	100
1	100

Benjamini	FDR
0.999981	99.999
0.999999	99.99998
1	100

1	100
1	100
1	100
1	100
1	100
1	100
1	100
0.996684	100
1	100
1	100
1	100
1	100
1	100
1	100

Benjamini	FDR
0.999998	100
0.975504	100
0.999988	100
0.998178	100

Benjamini	FDR
0.999978	100
0.999994	100
0.994316	100

Benjamini	FDR
0.981413	100
1	100
0.998178	100

Benjamini	FDR
1	100
1	100
1	100
1	100
1	100

Benjamini	FDR
1	100
0.999994	100
1	100
1	100
1	100
1	100

1	100
1	100

Benjamini	FDR
0.986298	100
0.979035	100
1	100
0.999974	100
1	100

Benjamini	FDR
0.992563	100
0.997838	100
1	100
1	100
1	100
0.999842	100

Benjamini	FDR
0.999901	100
0.99931	100
1	100
1	100

Benjamini	FDR
1	100
1	100
1	100
0.999941	100
1	100
1	100

Benjamini	FDR
0.996997	100
0.999712	100
1	100
1	100
1	100
1	100
1	100
1	100
