# Supplementary material for: Mutant p53-dependent mitochondrial metabolic alterations in a mesenchymal stem cell-based model of progressive malignancy
Source: Cell Death Differ. 2018 Nov 9;26(9):1566–81. doi: 10.1038/s41418-018-0227-z (PMC6748146; doi:10.1038/s41418-018-0227-z)
Supplement: Supplementary file 5 — Supplementary table 3 [file 41418_2018_227_MOESM5_ESM.pdf]

Table S1. Related to Material and Methods

Primers Used in this Study, Related to Experimental Procedures

Primers for cDNA relative to mouse cells

Glut1 F	CAGTTCGGCTATAACACTGGTG
Glut1 R	GCCCCCGACAGAGAAGATG
Glut3 F	ATGGGGACAACGAAGGTGAC
Glut3 R	GTCTCAGGTGCATTGATGACTC
Hexokinase 2 F	TGATCGCCTGCTTATTCACGG
Hexokinase 2 R	AACCGCCTAGAAATCTCCAGA
PDK1 F	GGACTTCGGGTCAGTGAATGC
PDK1 R	TCCTGAGAAGATTGTCGGGGA
MCT1 F	ACGCCGGAGTCTTTGGATTT
MCT1 R	TGAGGCGGCCTAAAAGTGG
Slc1A5 F	CATCAACGACTCTGTTGTAGACC
Slc1A5 R	CGCTGGATACAGGATTGCGG
POLG2 F	GAGCAACTCCATTCAAAGTACGA
POLG2 R	CATCGTGGTGTCTCTGCTCC
Sox2 F	AAAAAGCAGGCTTGTATAACATGATGGAGACGG
Sox2 R	AGAAAGCTGGGTTTCACATGTGCGACAGGGGCAGT
CD44 F	CACCATTGCCTCAACTGTGC
CD44 R	TTGTGGGCTCCTGAGTCTGA
MGST2 F	TGCCTGTCAGCAAAGTTATTTCG
MGST2 R	CTGGGGGTGCAATCTTGTGT
mGSTπ1 F	TACCCCTCTGTCTACGCAGC
mGSTπ1 R	GCCCATACAGACAAGTGGGC

Primers for cDNA relative to human cells

Glut1 F	TCTGGCATCAACGCTGTCTT
Glut1 R	AGCCAATGGTGGCATACACA
Hexokinase 2 F	ATTGCCGAATGCCTGGCTAA
Hexokinase 2 R	CATGAGACCAGGAAACTCTCGT
LDH-A F	AGCTGTTCCACTTAAGGCCC
LDH-A R	TGGAACCAAAAGGAATCGGGA
PDK1 F	TCCTGGACTTCGGATCAGTG
PDK1 R	TGCAACCATGTTCTTCTAGGC

Primers for internal controls

HPRT F	TCAGTCAACGGGGGACATAAA
HPRT R	GGGGCTGTACTGCTTAACCAG

Primers for mtDNA quantification

Mitoch. mouse mtND1 F	CTCTTATCCACGC TTCCGTTACG

Mitoch. mouse mtND1 R	GATGGTGGTACTCCCGCTGTA
Mitoch. mouse mtCO-I F	ACTTGCAACCCTACACGGAGGTAA
Mitoch. mouse mtCO-I R	TCGTGAAGCACGATGTCAAGGGAT
Nucl. mouse H19 F	GTACCCACCTGTCGTCC
Nucl. mouse H19 R	GTCCACGAGACCAATGACTG
Nucl. Mouse Rp18S F	ACCTGTCTTGATAACTGCCCGTGT
Nucl. Mouse Rp18S R	TAATGGCAGTGATGGCGAAGGCTA

Table S1. Primer sequences used for qPCR. F, forward; R, reverse
